# 5th Pediatric Allergy and Asthma Meeting (PAAM)

**DOI:** 10.1186/s13601-018-0204-0

**Published:** 2018-06-25

## O1

### Evolution of immunologic parameters in children subjected to food oral immunotherapy with cow's milk during a 10-year follow-up

#### Sara Bellón*, Laura Sánchez, Loreto González, Ana Moreira, Teresa Bracamonte, Sergio Quevedo, Luis Á. Echeverría

##### Pediatric Allergy Unit, Severo Ochoa University Hospital, Leganés, Spain

**Correspondence:** Sara Bellón - sara.bellon.alonso@gmail.com

*Clinical and Translational Allergy* 2018, **8(Suppl 2):**O1

**Introduction**

Food oral immunotherapy (OIT) has recently become an alternative to elimination diets in patients with cow’s milk IgE-mediated allergy. Here we report the evolution of skin prick test and immuno CAP through a 10-year period and the results of treatment during long-term follow-up.

**Methods**

In the period 2007–2017, one hundred and eight children with persistent cow’s milk allergy, confirmed by open oral food challenge, have been included in our OIT protocol. During the induction phase, they gradually received increasing doses of this food until a minimum of 3600 mg of protein per day.

We subsequently monitored these children annually performing skin and blood test to determine evolution of immunologic parameters and carrying out strict control of new symptoms and possible adverse reactions.

**Results**

In the analysed period, 108 patients underwent cow’s milk OIT with an initial median age of 6.38 years (2–16 years). A 53.7% (58) were males.

Follow-up time varies among our patients depending on the beginning date of OIT and reaches 10 years in the oldest cases.

In our series, the success rate of patients who have undergone cow’s milk OIT is 91.6%. Treatment failure was found in three patients during the initial induction phase and in six during the maintenance one. The most common reason of failed therapy was serious adverse reactions (44%) followed by development of eosinophilic esophagitis (22%). Two patients refused to continue OIT treatment because they dislike cow’s milk and another one discontinued OIT because of the coexistence of complicated kidney disease. Assessment of permanent tolerance was not proved in any of the patient.

Evolution of skin prick test and inmuno CAP is shown in Figs. 1, 2 and 3 respectively. The most significant decrease, both in prick and CAP values, is reached in the first 3 years of follow-up, as shown in the following charts. Regarding casein CAP levels specifically, we found an average decrease of 71.6% between last control titres and initial ones.

**Fig. 1:**
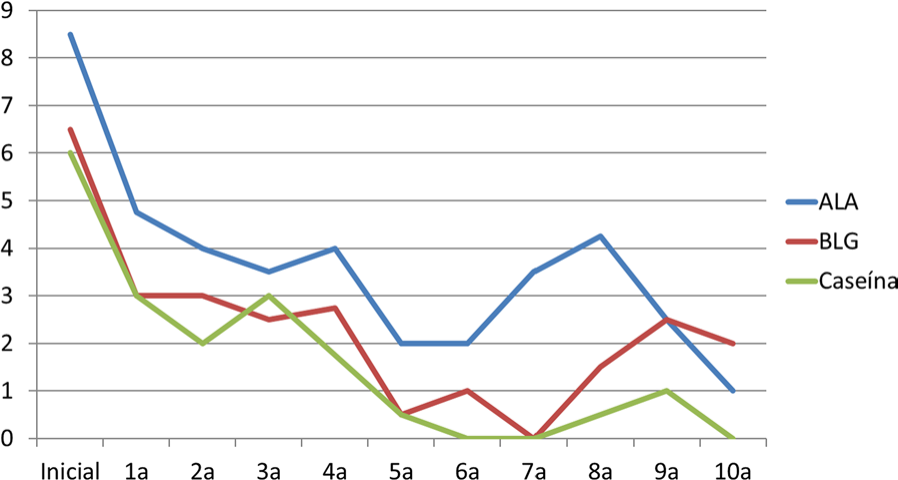
Skin prick test evolution

**Fig. 2:**
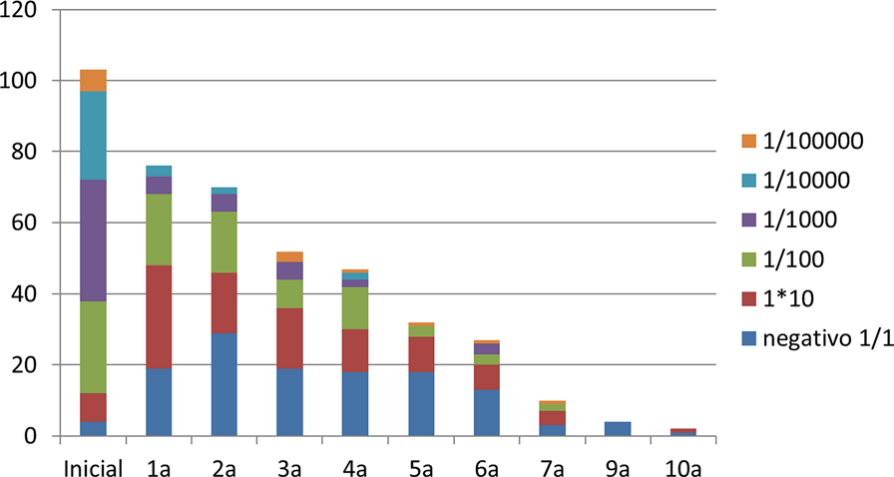
Dilution (cow’s milk) prick test evolution

**Fig. 3:**
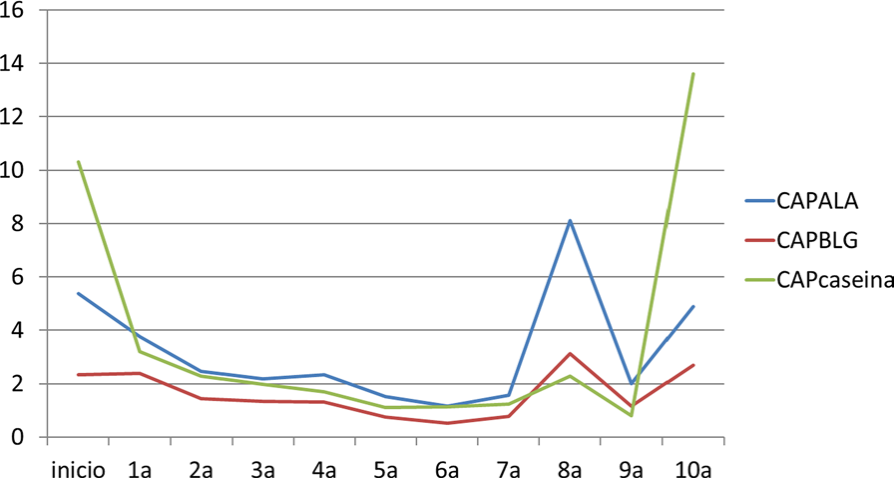
Immuno CAP evolution

**Conclusion**

OIT is a novel form of treatment that has already proven to be effective, reaching success rates greater than 90% in our experience. Decrease in prick and inmuno CAP levels is mostly seen in the first two or 3 years of follow-up and accurately predict tolerance in most of the patients.

## O2

### Cow's milk protein consumption and risk of adverse reactions in children subjected to food oral immunotherapy with cow's milk during a long-term follow-up

#### Sara Bellón*, Laura Sánchez, Loreto González, Ana Moreira, Teresa Bracamonte, Sergio Quevedo, Luis Á. Echeverría

##### Pediatric Allergy Unit, Severo Ochoa University Hospital, Leganés, Spain

**Correspondence:** Sara Bellón - sara.bellon.alonso@gmail.com

*Clinical and Translational Allergy* 2018, **8(Suppl 2):**O2

**Introduction**

OIT (oral immunotherapy) has proven efficacy in achieving cow’s milk tolerance. However, there is still low data about tolerance persistence rate and adverse effects (AE), not only at the escalation dose period, but also in maintenance period.

**Methods**

A 10-year clinical follow up study conducted in patients who went under cow’s milk IOT. During this period, we analysed: (1) Current cow’s milk protein consumption (2) Rate and severity of AE related to long-term follow-up. (3) Allergy test: SPT (skin prick test) and sIgE (specific IgE) to cow’s milk (CM) and casein at the beginning of IOT.

**Results**

108 children between 2 and 16 years old were included. 90.3% of them are currently consuming more than 200 ml of raw cow’s milk/day (group A). Group B was defined as children who are consuming less than 200 ml of raw cow’s milk/day. 3.2% of children are taking cow’s protein contained in dairy and 2.1% are able to tolerate only traces.

Significant statistically differences were detected in both groups respect SPT to cow’s milk (8.34 mm ± 3.40 vs. 12.25 mm ± 4.11, p < 0.029) and casein SPT (6.09 mm ± 2.99 vs. 10.25 mm ± 4.87, p < 0.010). Specific IgE levels to casein were higher in group B, but failed to reach statistical significance (36.80 KU/L ± 83.89 vs. 118.02 KU/L ± 91.55, p < 0.063).

Regarding adverse effects during the follow-up, 47.1% of our patients presented adverse reaction in some form, and 35.4% were defined as severe in accordance with Clark classification of allergic adverse reactions. We also found statistically significant difference in the initial casein SPT (7.09 mm ± 3.17 vs. 5.58 mm ± 2.81, p < 0.012), the initial specific IgE levels to beta-lactoglobulin (23.03 KU/L ± 33.51 vs. 7.59 KU/L ± 18.19, p < 0.005) and to casein (66.95 KU/L ± 105.41 vs. 19.65 KU/L ± 47.44, p < 0.004) between the group of children with adverse reactions and the one without them.

**Conclusion**

Cow's milk OIT is a safe and effective treatment in the medium term for those children who don’t acquire spontaneus tolerance. Adverse reactions are usually mild and are more likely to appear in patients with initial bigger SPT and high levels of specific IgE. Allergy test could be a good biomarker of prediction of final cow's milk protein consumption.

## O3

### Improvement of safety during the induction and maintenance phase of oral immunotherapy with cow’s milk and egg when assisted with omalizumab

#### Cristina Blasco-Valero, Paula Galván-Blasco*, Teresa Garriga-Baraut, Blanca Vilá-Indurain

##### Hospital Vall D’hebron, Barcelona, Spain

**Correspondence:** Paula Galván-Blasco - pauligalvan@gmail.com

*Clinical and Translational Allergy* 2018, **8(Suppl 2):**O3

**Introduction**

Oral immunotherapy (OIT) is a promising therapeutic alternative to food allergen avoidance in allergic patients. Nevertheless, it is associated with frequent adverse reactions that sometimes can be life-threatening and the need of adrenaline administration is not infrequent. Results from previous trials suggest that the use of omalizumab (OMZ) could potentially lead to safer and more efficient OIT protocols, by reducing the number and severity of reactions, and increasing allergen tolerance threshold. Currently, there is no consensus about the length of OMZ therapy. We here report our experience with a protocol of OMZ-assisted OIT in patients with egg and cow’s milk allergy.

**Methods**

We performed a retrospective observational study of nineteen pediatric patients under an OMZ-assisted OIT with cow’s milk (15 patients) or raw egg white (4 patients). Patients selected showed elevated levels of specific IgE (sIgE) to casein or ovomucoid and grade 2–3 anaphylactic symptoms. All patients/parents provided informed consent. OMZ was administered a minimum of 16 weeks before the start of the induction phase. Levels of total IgE, sIgE and skin prick test (SPT) were assessed, as well as the number and severity of adverse reactions during the induction and maintenance phase.

**Results**

The induction phase lasted a mean of 5 months; only2 anaphylactic reactions that required the use of adrenaline occurred. The dose of OMZ ranged from 300 to 1200 mg per month depending of total IgE values and weight. Nowadays, 12 patients have reached the maintenance phase. At this point, we decrease the dose of Omalizumab in 10 patients and in two cases was successfully discontinued (Fig. 1). All of these patients are currently tolerating the full maintenance dose, referring mild symptoms only 4 patients of the cow’s milk group. The maximumfollow-up extends to 5 years and only 3 severe adverse reactions have been reported, all associated to cofactors. In both groups, SPT diminished after the induction phase and over time. sIgE to cow’s milk and egg fractions increased during the induction phase, decreasing afterwards during the maintenance phase.

**Conclusions**

The present report describes the optimized safety in high risk patients undergoing OMZ-assisted OIT. The reduction or discontinuation of OMZ may be achieved without losing tolerance or increasing the number of severe reactions.

## O4

### Cow’s milk allergy from infancy to school age

#### Emma Goksör*, Natascha Lougheide-Camejo, Bernt Alm, Göran Wennergren

##### Department of Pediatrics, Institution of Clinical Sciences, University of Gothenburg, Gothenburg, Sweden

**Correspondence:** Emma Goksör - emma.goksor@vgregion.se

*Clinical and Translational Allergy* 2018, **8(Suppl 2):**O4

**Introduction**

Allergy towards cow’s milk protein is the most common food allergy during infancy. The prognosis is considered to be good, but reports of prevalence and prognosis are varying.

The aim is to analyse the prevalence of cow’s milk allergy during infancy, and the prognosis and comorbidity until 12 years.

**Methods**

Data were obtained from a longitudinal study of children born in western Sweden in 2003. Parents answered postal questionnaires when the children were 6 months and 1, 4, 8 and 12 years. Questions about doctor-diagnosed food allergy, triggering allergens and symptoms were asked at age 1, 4, 8 and 12 years. At 4, 8 and 12 years, questions about allergy testing were included. The response rate at 12 months was 88% (4987/5654) and at 12 years 76% (3637/4777), that is 64% of the 5654 originally included.

**Results**

The prevalence of reported doctor-diagnosed cow’s milk allergy in infancy was 3.5% (173/4944) and it was the most common reported food allergy with 72%. Most children with doctor-diagnosed cow’s milk allergy in infancy reported symptoms from milk only (55%), while another 30% had symptoms also from eggs; 64% had eczema during infancy.

At 12 years, 36% reported no allergic disease at all. However, 15% still reported symptoms from cow’s milk and 24% from other foods. 12% reported persistent symptoms at all follow-ups (4, 8, and 12 years). Most of the children who grew out of symptoms did so between 4 and 8 years.

Over 90% of the children with persistent food-allergy at 12 years reported other allergic manifestations. In addition, 51% of the children who had become symptom-free from food-allergy at 12 years reported having other allergic manifestations, like asthma, rhinitis and eczema instead.

Having other food allergies than cow’s milk during infancy increased the risk of doctor-diagnosed food allergy at 12 years (OR 4.3; 95% CI 1.8–10.1), compared to those with only cow’s milk allergy. In addition, eczema during infancy (OR 2.9; 1.2–7.5), more severe symptoms (OR 3.7; 1.6–8.6) and parental asthma (OR 2.4; 1.01–5.9) increased the risk.

**Conclusion**

Of children with doctor-diagnosed cow’s milk allergy during infancy 85% became symptom-free before 12 years of age. Multiple food allergy in infancy, more severe disease, eczema and parental asthma increased the risk of persistent food-allergy at 12 years of age.

## O5

### Children with Asian-born parents living in Melbourne have more allergy in the first 6 years of life

#### Noor H. A. Suaini^1,2^*, Jennifer J. Koplin^2^, Justine A. Ellis^1,2,3^, David J. Martino^1,2^, Shyamali C. Dharmage^4^, Adrian J. Lowe^4^, Mimi L. K. Tang^1,2,5^, Anne-Louise Ponsonby^1,4^, Lyle C. Gurrin^4^, Melissa Wake^1,2,6^, Katrina J. Allen^1,2,5,7^, for the HealthNuts study group

##### ^1^Department of Pediatrics, University of Melbourne, Parkville, Victoria, Australia; ^2^Murdoch Children’s Research Institute, Parkville, Victoria, Australia; ^3^Centre for Social and Early Emotional Development, Faculty of Health, Deakin University, Burwood, Victoria, Australia; ^4^The School of Population and Global Health, University of Melbourne, Carlton, Australia; ^5^Department of Allergy and Clinical Immunology, Royal Children’s Hospital, Parkville, Victoria, Australia; ^6^The Department of Pediatrics and the Liggins Institute, The University of Auckland, Auckland, Australia; ^7^Institute of Inflammation and Repair, University of Manchester, Manchester, United Kingdom

**Correspondence:** Noor H. A. Suaini - noor.suaini@mcri.edu.au

*Clinical and Translational Allergy* 2018, **8(Suppl 2):**O5

**Introduction**

We previously found that Melbourne infants with Asian-born parents are three times more likely to have food allergy than infants with Australian-born (predominantly Caucasian) parents. It is not known if this increased likelihood translates to higher rates of allergic comorbidities later in childhood. Using data from a longitudinal population-based study of children in Melbourne, Australia, we aim to assess whether the evolution of infantile food allergy is associated with an increased risk of other allergic diseases in later childhood and whether there is a differential pattern in this evolution amongst infants with Asian-born compared to non-Asian born parents.

**Methods**

Participants were recruited at 12-months from community immunisation centres (N = 5276) and followed up at 6 years old (N = 4411). Of those followed up, 3131 had a parent born in Australia, UK or Europe (non-Asians) and 425 in East Asia (Asians), while others were excluded from analyses. Food allergy status at age one and 6 years was determined using skin prick tests (SPT) and oral food challenge. SPT to aeroallergens were also undertaken. Data on asthma, hayfever and eczema were obtained from the International Study of Asthma and Allergies in Childhood questionnaires. Logistic regression was used to estimate odds ratios and 95% confidence intervals.

**Results**

At age six, hayfever and aeroallergen sensitisation were more common in children of Asian parents (28.3%, 95% CI 24.1–32.9; 69%, 95% CI 63.4–74.2, respectively) than children of non-Asian parents (17.6%, 95% CI, 16.3–19.0; 36.9%, 95% CI 34.9–39 respectively; both p < 0.001). However, asthma prevalence was similar in Asians (12.9%, 95% CI 10.0–16.6) and non-Asians (14.5%, 95% CI 13.3–15.8; p = 0.387).

Children with food allergy and eczema at age one were 5 times more likely to be diagnosed with asthma at 6 years, irrespective of parental country of birth (4.8, 95% CI 3.4–6.8 for non-Asians; 5.6, 95% CI 2.4–12.9 for Asians; p < 0.001 for both). They were also 3 times more likely to have hayfever (non-Asians OR 3.3, 95% CI 2.3–4.7, p < 0.001; Asians OR 2.5, 95% CI 1.3–4.9, p = 0.006).

**Conclusion**

Children of Asian-born parents in Australia not only have more food allergy and eczema in infancy but also go on to have more aeroallergen sensitisation and hayfever by age 6 years. Interestingly however, rates of asthma at age 6 years old are not higher in children of Asian-born. Having food allergy and eczema in infancy is strongly associated with asthma and hayfever later in childhood, irrespective of parental country of birth.

## O6

### Improving safety of egg oral immunotherapy with a boiled-egg protocol

#### Adrianna Machinena*, Montserrat Alvaro, Jaime Lozano, Carmen Riggioni, Mónica Piquer, Olga Dominguez, Rosa Jimenez, Maria Teresa Giner, Ana Maria Plaza

##### Pediatric Allergy and Clinical Immunology Department, Hospital Sant Joan de Déu, Barcelona, Spain

**Correspondence:** Adrianna Machinena - amachinena@hsjdbcn.org

*Clinical and Translational Allergy* 2018, **8(Suppl 2):**O6

**Introduction**

Our group has previously published data regarding safety in a raw egg-OIT protocol, reporting adverse reactions in 7.6% of doses. Safety of egg oral immunotherapy (OIT) is a source of concern. Many studies report early discontinuation in egg-OIT due to severe adverse events. However, one of the aims of OIT protocols is to prevent severe reactions after accidental exposure in children with more persistent/severe egg-allergy.

To evaluate the safety of boiled egg (BE)-OIT up-dosing phase protocol in egg allergic children.

**Methods**

Prospective study of egg allergic children following the protocol from January 2015. Data are collected for demographics, adverse events at oral food challenge (OFC) and during up-dosing phase. Specific-IgE (s-IgE) and skin prick test (SPT) are expressed with median. Written informed consent has been signed. Open OFC (PRACTALL consensus) with BE was performed, confirming allergy. Anaphylaxis was defined following EAACI position paper criteria. A BE-OIT protocol was set up reaching a total dose of 1 cooked egg in 11 weeks.

**Results**

27 patients have been enrolled, 74.1% (20/27) boys, median age 9 years (IQ range, 7–12). 59.3% (16/27) had previous anaphylaxis history and all underwent an open OFC previous to start OIT-protocol except 2 patients because of recent reaction. At OFC: Median Total IgE 1065.5 KU/L (416.5–1914.2) and s-IgE: ovalbumin (OVA)-sIgE 3.19 KU/L (0.74–13.3), ovomucoid (OVM)-sIgE 4.47 KU/L (1.91–20.4), egg white (EW)-sIgE 6.6 KU/L (2.31–26.6). Median SPT for: OVA 7.7 mm (6.2–10.5), OVM 9.7 mm (7.6–11.6), EW 10.4 mm (7.7–12.2). 51.9% (14/27) presented anaphylaxis. OIT up-dosing phase: 74.1% (20/27) patients completed it in an average of 13 weeks, reaching 7.5 gr of BE-protein. 70.3% of patients (19/27) had adverse events: 48.1% gastrointestinal, 41% anaphylaxis, 14.8% respiratory and 14.8% cutaneous. 11 (41%) patients had anaphylaxis, 6 at hospital during up-dosing and 5 at home. Adrenaline was administered in 3 patients (11.1%), 2 at home and 1 at hospital; they withdrew the protocol. In all, 7 patients dropped out: 4 (14.8%) due to anaphylaxis and 3 for family reasons. 2554 doses were administrated and 61 reactions occurred (2.4% of doses). The decrease of ovomucoid-sIgE and egg white-sIgE from initial time and after 1 year of treatment has statistical significance (p 0.007–0.008 respectively).

**Conclusion**

BE-OIT up-dosing phase protocol could be completed in most children (70.4%) almost within the expected time. The most frequent adverse events were gastrointestinal, followed by anaphylaxis. 14.8% of the patients who withdrew the protocol had anaphylaxis and needed adrenaline. The rate of adverse events per doses was 2.4%. Children were able to eat 1 cooked egg (7.5 gr of protein) which improves children’s diet by including baked and usual amounts of fully cooked egg.

## O7

### Allergic rhinitis in children with asthma is under recognised and undertreated

#### Miranda Crealey*, Ursula Caulfield, Angela Mernagh, Miriam Kennedy, Michael Williamson, Fiona Healy

##### Department of Pediatric Respiratory Medicine, Temple St Children’s University Hospital, Dublin, Ireland

**Correspondence:** Miranda Crealey - mirandac@eircom.net

*Clinical and Translational Allergy* 2018, **8(Suppl 2):**O7

**Introduction**

Allergic rhinitis (AR) is a disorder of the nose induced after allergen exposure by an immunoglobulin E (IgE) mediated inflammation of the membranes lining the nose [1]. Typical symptoms include nasal discharge, sneezing, rhinorrhoea and nasal blockage and it is frequently associated with conjunctivitis. Over 80% of asthmatics have rhinitis and 10–40% of patients with rhinitis have asthma suggesting the concept of “one airway, one disease” [2]. AR has a significant impact on quality of life impairing sleep, concentration and exam results [3].

**Methods**

Data was collected prospectively on successive asthmatic patients with AR attending the respiratory clinic in Temple St Children’s hospital (TSH) using a questionnaire modified from the Allergic rhinitis in Asthma (ARIA) questionnaire between March and May 2017. SPSS™ software package was used and Chi square test looked for associations between categorical variables.

**Results**

Data was collected on 89 consecutive patients with AR and asthma. Table 1 reports the clinical characteristics.

**Table 1:** Clinical features of children with allergic rhinitis and asthma (N = 89)

Median age (upper quartile, lower quartile)	7.5 years (5.5, 9)
Symptoms	
Nasal discharge	57 (72%)
Sneezing	83 (93%)
Nasal obstruction	70 (78%)
Nasal itching	56 (63%)
Watery itchy eyes	
Diagnosis: aeroallergen sensitisation	
SPT done	68 (76%)
1 or more positive SPT	60 (88%)
Specific IgE done	45 (50%)
SPT or specific IgE done	76 (85%)
*Classification of AR*	
Intermittent	29 (33%)
Mild	20 (69%)
Moderate-severe	9 (31%)
Persistent	60 (67%)
Mild	28 (47%)
Moderate-severe	32 (53%)
Asthma management (GINA stage)	
Stage 1	3 (4%)
Stage 2	24 (27%)
Stage 3	43 (48%)
Stage 4/5	18 (20%)
Leukotriene receptor antagonist	65 (73%)
AR treatment	
No treatment	20 (23%)
Intermittent treatment	55 (61%)
Regular treatment	14 (16%)

*HDM* house dust mite, *SPT* skin prick test, *IgE* immunoglobulin E, *AR* allergic rhinitis

44 (49%) did not have a diagnosis of AR in their medical notes but met the criteria for diagnosis on completion of the questionnaire. 73% were commenced on new AR medication after completion of the questionnaire (15 antihistamine, 22 intranasal corticosteroid, 60 nasal douching).

**Conclusion**

Many children with asthma and AR have persistent AR symptoms and 23% are not taking any AR medication. Almost half of this cohort did not previously have a diagnosis of AR and 73% were prescribed new medication after completion of the audit questionnaire. This highlights a lack of awareness of AR among both patients and healthcare professionals. Since this audit was completed, we have designed an algorithm for the management AR in the clinic and will re-audit later in 2017. We recommend regularly looking for AR in asthmatic patients, especially in those with uncontrolled asthma symptoms.

**References**

1. Holgate ST. Allergy: Edinburgh :Saunders, 2012. 4th ed.; 2012.
2. Brozek JL, Bousquet J, Baena-Cagnani CE, Bonini S, Canonica GW, Casale TB, et al. Allergic Rhinitis and its Impact on Asthma (ARIA) guidelines: 2010 revision. J Allergy Clin Immunol. 2010;126(3):466–76.
3. Valls-Mateus M, Marino-Sanchez F, Ruiz-Echevarría K, Cardenas-Escalante P, Jiménez-Feijoo R, Blasco-Lozano J et al. Nasal obstructive disorders impair health-related quality of life in adolescents with persistent allergic rhinitis: a real-life study. Pediatr Allergy Immunol. 2017 Apr 19. 10.1111/pai.12724.

## O8

### Efficacy of allergen blocker mechanical barrier gel on symptom and quality of life in patients with allergic rhinitis

#### Sirin Kose Seda, Atakul Gizem*, Asilsoy Suna, Karaman Ozkan, Uzuner Nevin, Anal Ozden

##### Department of Immunology and Allergy, Faculty of Medicine, Dokuz Eylul University, Izmir, Turkey

**Correspondence:** Atakul Gizem - drgizematakul@gmail.com

*Clinical and Translational Allergy* 2018, **8(Suppl 2):**O8

**Introduction**

Control of environmental and triggering factors is important in the treatment of allergic rhinitis. In recent years, physical barrier methods have been used to prevent contact between the allergen agent and the mucous membrane. The aim of this study was to evaluate the efficacy of allergen blocker mechanical barrier gel by symptom scores and quality of life in patients with allergic rhinitis.

**Methods**

A total of 45 patients with allergic rhinitis, between 6 and 18 years of age were included. Allergen blocker gel was recommended at least twice a day in patients with allergic rhinitis who had symptoms during examination. Participants were assessed by nasal sypmtom score (NSS), ocular symptom score (OSS), total symptom score (TSS), visual analogue scale (VAS) and quality of life using questionnaires, at pretreatment, 1st week and 1st month.

**Results**

The study consisted of 45 patients, 42.2% (19) girls and 57.8% (26) boys. Family history of allergic disease was found in 38 (84.4%) patients. Nineteen (42%) patients were allergic to house dust mite, 6 (13%)to pollen and 3 (17.5%) to cat dander, and 17 (38%) had mix allergies. Thirty (66.7%) patients were receiving at least one treatment (24.4% antihistamines, 42.2% nasal corticosteroids, 40% montelukast). Symptom scores decreased significantly in both participants, either using or not using medications (P < 0.001). Statistically significant decrease was observed in NSS, OSS, TSS and VAS after treatment with allergen blocker gel in all patients (p < 0.001). In the quality of life scale, the post-treatment values of the patients significantly improved compared to the pre-treatment values.

**Conclusion**

Allergen blocker mechanical barrier gel therapy is seen as an effective treatment option in patients with allergic rhinitis when evaluated by symptom scores and quality of life scale.

## O10

### Whole-genome shotgun sequencing for nasopharyngeal microbiome in preschool children with asthma exacerbation

#### Leung Ting Fan^1^*, Kwok Jaime^2^, Song Yuping^1^, Tang Man Fung^1^, Tung Christine^2^, Chan Renee Wan-Yi^1^, Leung Agnes Sze-Yin^1^, Tao Kin Pong^1^, Wong Gary Wing-Kin^1^, Tsui Stephen Kwok-Wing^2^

##### ^1^Department of Pediatrics, the Chinese University of Hong Kong, Hong Kong, China; ^2^School of Biomedical Sciences, the Chinese University of Hong Kong, Hong Kong, China

**Correspondence:** Leung Ting Fan - leungtf@netvigator.com

*Clinical and Translational Allergy* 2018, **8(Suppl 2):**O10

**Introduction**

Several studies reported upper airway microbiota in children with bronchiolitis and recurrent wheeze using 16S rRNA-based sequencing approach. However, there has not been any metagenomic study that delineates the high-resolution composition of airway microbiota in these patients. This study characterised nasopharyngeal (NP) microbiome of Chinese children with asthma exacerbation.

**Methods**

NP secretions were collected in Jan-Apr 2015 from 76 preschool children, which consisted of 24 cases Hospitalised for human rhinovirus (HRV)-associated asthma exacerbations, 14 inpatient controls with upper respiratory tract infection (RTI) who were virus-negative, and 38 community controls without any RTI for at least 4 weeks. Genomic DNA extracted by PowerSoil DNA Isolation Kit (MO BIO Laboratories) was sequenced using Illumina HiSeq X Ten. High-quality reads were mapped to human reference genome Ensembl version GRCh38.87 using Burrows-Wheeler Aligner version 0.7.15-r1140. Following removal of human sequences, these reads underwent microbial taxonomic classification using MetaPh1An2 version 2.6.0. Heatmaps of relative abundances for bacteria and viruses and cladogram were generated using R version 3.3.1 and MetaPh1An2’s utility scripts respectively. Linear Discriminant Analysis (LDA) scores for microbial abundances were calculated by LDA Effect Size programme.

**Results**

The mean (SD) age of cases, inpatient controls and community controls was 3.5 (1.0), 3.5 (1.0) and 4.9 (0.7) years respectively. All groups had low but similar NP biomass (5–7% of all sequence reads). NP biodiversity as represented by Shannon diversity index was lower in cases with asthma exacerbations than community controls median [IQR]: 3.49 [2.81–3.97] vs. 4.17 [3.24–5.84], *P *= 0.029) but similar to inpatient controls (3.44 [2.30–4.46], *P *= 0.674). Detailed microbiome analyses revealed patients with asthma exacerbation to have higher levels of viruses (log LDA score 5.20, *P *= 0.0031) and lower levels of bacteria (log LDA score 5.20, *P *= 0.0032). Bacilli (taxonomic level: class) was lower in cases compared to inpatient and community controls (log LDA score 4.82, *P *= 0.0055), which was accounted for by lower Lactobacillales (order). The class Actinobacteria was higher in community controls than cases and inpatient controls (LDA score 4.47, *P *= 0.00076). Specifically, this difference was due to Bifidobacterium (genus) (log LDA score 4.24, *P *= 0.021). Community controls also had higher abundances of *Clostridium hathewayi*, *Enterobacter cloaceae* and *Akkermansia muciniphila*, but these were at rare abundance levels.

**Conclusion**

NP biodiversity is lower in children with asthma exacerbations than community controls. Our results also show high abundance of virus-matched reads in metagenome of most NP samples.

**Acknowledgements**

Funded by Research Committee’s One-off Fund for Research (3132910) of CUHK

## O12

### A precision medicine trial to study heterogeneity in pediatric asthma: design of the PUFFIN trial

#### Elise M. A. Slob^1^*, Susanne J. H. Vijverberg^1^, Mariëlle W. Pijnenburg^2^, Gerard H. Koppelman^3^, Anke-Hilse Maitland - van der Zee^1^

##### Academic Medical Center, Amsterdam, The Netherlands; ^2^ Erasmus Medical Center, Rotterdam, The Netherlands; ^3^ University Medical Center Groningen, Groningen, The Netherlands

**Correspondence:** Elise M. A. Slob - e.m.slob@amc.nl

*Clinical and Translational Allergy* 2018, **8(Suppl 2):**O12

**Introduction**

There is large heterogeneity in treatment response to asthma medication and a one-size fits all approach based on current guidelines might not fit all children with asthma. It is expected that children with one or more variant alleles (Arg16Arg and Arg16Gly) within the beta2 adrenergic receptor (*ADRB2)* gene coding for the beta2-receptor have a higher risk to poorly respond to long-acting beta2-agonists (LABA) comparing to the Gly16Gly wildtype [1, 2].

**The aim is to** study whether *ADRB2* genotype-guided treatment will lead to improvement in asthma control in children with uncontrolled asthma on inhaled corticosteroids compared with usual care.

**Methods**

A multicentre, double-blind, precision medicine, randomized trial will be carried out within 15 Dutch hospitals. 310 asthmatic children (6–17 years of age) not well controlled on a low dose of inhaled corticosteroids (ICS) will be included and randomized over a genotype-guided and a non-genotype-guided (control) arm. In the genotype-guided arm children with Arg16Arg and Arg16Gly will be treated with double dosages of ICS and with the Gly16Gly wildtype with add on LABA. In the control arm children will be randomized over both treatment options (Fig. 1). Lung function measurements, questionnaires focussing on asthma control (ACT/c-ACT) and quality of life, will be obtained in three visits within 6 months.

**Fig. 1:**
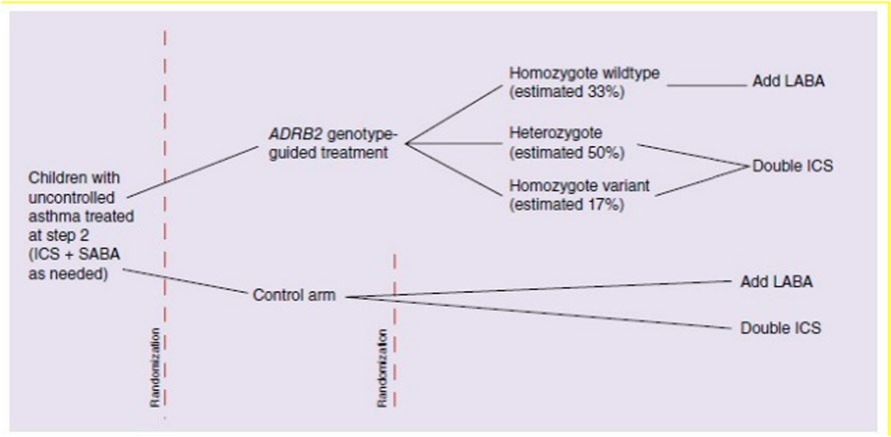
.

**Results**

The primary outcome will be improvement in asthma control based on repeated measurement analysis of c-ACT or ACT scores in the first 3 months of the trial. Additional cost effectiveness studies will be performed [3].

**Conclusion**

Currently, pharmacogenetics is not used in pediatric asthmas. This trial may pave the way to implement promising results for genotype-guided treatment in pediatric asthma in clinical practice.

**References**

1. Lipworth BJ, Basu K, Donald HP et al. Tailored second-line therapy in asthmatic children with the Arg (16) genotype. *Clin. Sci. (Lond)* 124(8), 521–528 (2013).
2. Turner S, Francis B, Vijverberg S et al. Childhood asthma exacerbations and the Arg16 beta2-receptor polymorphism: a meta-analysis stratified by treatment. *J Allergy Clin*. Immunol. 138(1), 107.e105–113.e105 (2016).
3. Vijverberg SJH, Pijnenburg MW, Hövels AM, Koppelman GH, Maitland-van der Zee AH. The need for precision medicine clinical trials in childhood asthma: rationale and design of the PUFFIN trial. *Pharmacogenomics.* 18(4), 393–401 (2017).
4. The PUFFIN trial is funded by the Lung Foundation Netherlands (projectnr: 5.1.16.094).

## O13

### Treatment of chronic urticaria in children—A cross-sectional analysis of specialized dermatological care in pediatric patients

#### Petra Staubach*, Sebastian Zimmer, Berenice Lang, Dennis Maeck, Anja Weber, Tom Gilfert, Max Jaeger, Adriane Peveling-Oberhag

##### Department of Dermatology, University Medical Center of Mainz, Johannes Gutenberg-University Mainz, Mainz, Germany

**Correspondence:** Petra Staubach - petra.staubach@unimedizin-mainz.de

*Clinical and Translational Allergy* 2018, **8(Suppl 2):**O13

**Introduction**

Chronic urticaria (CU) is a common disease occuring in all ages. But CU in children has been devoted less attention so far. International management guidelines for pharmacotherapy in children derive largely from evidence in the adult.

**Objective**

To examine the clinical presentation, disease burden and pharmacological treatments in childhood to develop strategies for effective management of children with CU.

**Methods**

200 children (0–17 years, 57% females) with CU were included in a standardized extended diagnostic program in the specialized urticaria outpatient clinic, Department of Dermatology, University Medical Center Mainz, Germany from 2012 to 2015.

**Results**

The disease duration at time of presentation ranged from 2 months to 9 years. 62.5% presented with chronic spontaneous urticaria (CsU), 28% with chronic inducible urticaria, and 9.5% showed a combination. 15% of patients had not received any treatment. The majority (73%) were treated with second generation antihistamines (single dose) as monotherapy. Only 4 patients received an updosing (doubled recommended daily dose) as suggested by guidelines. 53% showed persisting symptoms despite therapy. Of these insufficiently treated patients 60% received single-dose second generation antihistamines without updosing. All eight patients (4%) on first generation antihistamines reported ongoing symptoms andalso all patients with additional montelukast treatment did not have full symptom control. Eleven patients (8.3%) received steroid pulse therapy in addition to antihistamines with consecutive symptom control in all cases.

**Conclusion**

The current data suggest a significant pharmacological undertreatment in children with CU. Although more than half of the patients were symptomatic under therapy with single-dose second generation antihistamines, no updosing or change of medication was performed. Treating physicians should be alerted to existing options of treatment escalation in pediatric CU and further investigations are urgently necessary to optimize the management of CU in children.

## O14

### Airborne spore allergens, air pollutants and socioeconomic status as risk factors for childhood allergic diseases in West Bengal, India

#### Partha Karak*, Kashinath Bhattacharya

##### Department of Botany, Environmental Biology Lab., Visva-Bharati (A Central University) Santiniketan - 731235, West Bengal, India

**Correspondence:** Partha Karak - parthakarak@rediffmail.com

*Clinical and Translational Allergy* 2018, **8(Suppl 2):**O14

**Introduction**

Airborne allergens load vary from one climatic region to another. Moreover, synergistic effect of persistent exposure to aeroallergens in a particular set of climatic condition along with other parameters like socio-economic status, age group, air pollutants, etc., are major risk factors for childhood allergy. Hence an attempt has been made to find out such relationship among the childhood allergic diseases in West Bengal, India.

**Methods**

A total of 1536 pediatric subjects diagnosed as allergic patients at different sub- divisional Hospitals at Durgapur and Bolpur, West Bengal were thoroughly studied in presence of clinician with the help of ISAAC questionnaires. The prior ethical approval and written consent were taken from the patient’s guardians. A Burkard 7-day volumetric and an Andersen two-stage sampler were concurrently used for monitoring and assessment of airborne fungal spores. Skin prick tests were performed with 11 dominant fungal species as per EAACI guideline. Multiple logistic regression analysis was performed to estimate the association between air pollutants, allergen exposure and the risk of allergic diseases with adjustments for potential confounders.

**Results**

Children with age group of 1–5 years showed higher prevalence in atopic dermatitis (15.79%), idiopathic urticaria (3.51%), cold and heat urticaria (3.75%), dermatographism (3.65%), chronic urticaria (14.4%), allergic contact dermatitis (3.51%), food allergy (3.51%), insect bite allergy (3.51%). While children among 6–19 years age group showed higher prevalence in allergic acute urticaria (23.19%), cough (60.14%), asthma (37.68%), allergic rhinitis (27.90%), rhinosinitis (2.17%), chronic allergic conjunctivitis (20.29%), mite allergy (2.54%), etc. The synergistic effects of pollution, fungal spore load and meteorological factors showed an increase in asthma severity 2.441(1.60–3.70), allergic conjunctivitis 0.277(0.145–0.529), rhinosinitis 3.453(1.44–8.28) and chronic urticaria 0.267(0.129–0.55). A clinically significant association of fungal spores with allergic symptoms were observed such as *Penicillium oxalicum* with asthma, *Aspergillus flavus* with allergic urticaria, and *Aspergillus tenuis* with rhinitis.

**Conclusion**

Our findings are in line with the perception of increase in the occurrence of atopic dermatitis, cough, asthma or conjunctivitis among children in the study area. Children below 10 years age were severely affected with at least one or more allergic symptoms. In general, girl child showed more sensitivity than boys. The lower economy class people are more vulnerable to atopic dermatitis perhaps due to their ignorance about sanity and poor nourishment.

## O15

### Effects of a swimming training session on skin barrier function in elite athletes

#### Ana Rodolfo^1^*, Inês Paciência^2^, Tiago Rama^1^, Leonor Leão^1^, Diana Silva^3^, João Rufo^2^, Francisca Mendes^4^, Patrícia Padrão^5^, Eduardo de Oliveira Fernandes^6^, Pedro Moreira^7^, Luís Delgado^8^, André Moreira^8^

##### ^1^Centro Hospitalar de São João, Porto, Portugal; ^2^Faculdade de Medicina; Instituto de Saúde Pública, Inegi, Porto, Portugal; ^3^Centro Hospitalar de São João; Serviço e Laboratório de Imunologia, Faculdade de, Porto, Portugal; ^4^Serviço e Laboratório de Imunologia, Faculdade de Medicina, Porto, Portugal; ^5^Instituto de Saúde Pública; Faculdade de Ciências da Nutrição e Alimenta, Porto, Portugal; ^6^Institute of Science and Innovation in Mechanical Engineering and Industrial Management (Inegi), Porto, Portugal; ^7^Faculdade de Ciências da Nutrição e Alimentação da Universidade do Porto, Porto, Portugal; ^8^Centro Hospitalar de São João; Faculdade de Medicina; Instituto de Saúde Pública, Porto, Portugal

**Correspondence:** Ana Rodolfo - aipre@hotmail.com

*Clinical and Translational Allergy* 2018, **8(Suppl 2):**O15

**Introduction**

The benefits of swimming on asthma have been extensively accessed. However, exposure to chlorine by-products and other potentially irritating chemicals in swimming pools have been correlated with perceived health problems in swimmers, namely skin irritation that may lead to the interruption of this practice. This study aimed to evaluate, in elite swimmers, whether skin barrier function, as measured by transepidermal water loss (TEWL), is affected by a training session.

**Methods**

Elite swimmers enrolled in the SWAN trial—Swimming Pool Environment Impact on the Human Respiratory Health (ClinicalTrials.gov Identifier: NCT03017976) were invited to participate. Due to the lack of prior information it was not possible to calculate the sample size and all athletes that provided informed consent were included in the analysis (n = 33, 23 females, aged 12–21 years). None of the participants used emollients. TEWL was measured using the Tewameter^®^ TM 300 (Courage and Khazaka, Germany) in an environmentally controlled room, before, immediately after, and 30 min after a 2 h training session. The probe was held on the dorsum of hand, the volar forearm and the antecubital flexure for approximately 60 s each, or until a steady TEWL value was achieved. The average of two consecutive measurements was recorded. Non-parametric statistics were used as appropriate. Ethical approval was obtained from the University Clinical Research Ethics Committee.

**Results**

TEWL was independent of gender and allergic sensitization according with the Mann–Whitney U Test findings. A Friedman test revealed a significant effect of swimming on TEWL of dorsum of hand (X^2^(1) = 31.6; p < 0.001), volar forearm (X^2^(1) = 43.3.6; p < 0.001) and antecubital flexure (X^2^ 1) = 41.9; p < 0.001). Pairwise analysis for volar forearm and antecubital flexure showed a significant increase of the TEWL (all p < 0.001) in both measurements after training in comparison to baseline and between immediately and 30` after swimming. For the dorsum of hand, although significantly increased from baseline (p < 0.001), no significant differences between immediately and 30’ after swimming were seen.

**Conclusion**

In conclusion, TEWL increases significantly after a single swimming training session in elite swimmers. The clinical significance of this is findings and potential long term effect in skin barrier function is unclear and requires further investigation.

**Aknowledgements**

Authors gratefully acknowledge the funding of Project NORTE-01-0145-FEDER-000010–Health, Comfort and Energy in the Built Environment (HEBE), cofinanced by Programa Operacional Regional do Norte (NORTE2020), through Fundo Europeu de Desenvolvimento Regional (FEDER).

## O16

### Pediatric chronic urticaria: epidemiology of utility of basophil activation test

#### Carmen Riggioni^1^*, Yadira Gordon^1^, Montserrat Alvaro-Lozano^1^, Mariona Pascal^2^ Monica Piquer^1^, Jaime Lozano^1^, Olga Dominguez^1^, Maria Teresa Giner^1^, Rosa Jimenez-Feijoo^1^, Adriana Machinena^1^, Mar Folque^1^, Marcia Dias^1^, Ana Maria Plaza^1^

##### ^1^Hospital Sant Joan De Deu, Barcelona, Spain; ^2^Hospital Clinic, Barcelona, Spain

**Correspondence:** Carmen Riggioni - criggioni@hotmail.es

*Clinical and Translational Allergy* 2018, **8(Suppl 2):**O16

**Introduction**

Chronic urticaria (CU) is a mast-cell and basophil driven disease, but cell activating signals are ill-defined and heterogeneous. There is limited evidence of CU in children. Objective: To evaluate the clinical features, possible causes, associated findings and laboratory results of CU in a pediatric population.

**Methods**

Prospective observational study in the Pediatric Allergy and Clinical Immunology Department at Sant Joan de Déu Hospital, Barcelona, Spain. From April 2015 to 2017. The following data were recorded: age of onset, sex, duration and severity of symptoms, triggering factors, comorbidities, personal/family history, diagnostic tests and laboratory including basophil activation test (BAT) with autologous serum.

**Results**

A total of 52 patients were diagnosed with CU, females 59.6%, median (*M*) age 8 years (1–14). Personal and family history of atopy in21.6 and 36.5% respectively. Personal and family history of autoimmune disease were both 9.8%. The duration of urticaria was *M* 24 months (range 3–144). A*M* of 97 days with active disease per year was recorded after initial diagnosis, ranging from 3 months to daily throughout the year. According to EAACI guidelines, treatment was on step-1 (single dose antihistamines) in 59.6%, on step-2 (increased dose of antihistamines) 26.9%, and on step-3 (omalizumab or cyclosporine) 13.5%.

With regards to the subtypes of CU, spontaneous (73.1%) was more common than inducible (26.9%). Causes of induced CU were: symptomatic dermographism 11.5%, cold 9.6%, exercise 1.9% and heat 3.9%. After induced causes were excluded, laboratory tests were performed confirming infectious etiology in 11.5%. and autoinmune disease in 9.6%.

No possible cause was identified in 50% of patients. To these, BAT was performed in the 21 patients with active disease, 42.9% were positive. There were no significant statistical differences between BAT results regarding sex, age of onset, duration or treatment used. However, patients with positive BAT had a significantly higher number of active symptoms in days/year (p = 0.001). Patients with positive TAB had a*M* of 190 symptomatic days/year (100–365) vs. 102 (47–132) in the negative TAB group.

**Conclusions**

Chronic urticaria in our pediatric population was found to have a diverse etiology. In most cases of non-inducible urticaria, an infectious etiology could be established.

The exclusion of inducible urticaria, combining a thorough clinical history with according diagnostic tests, prevents the need to perform unnecessary tests in these children.

Children with chronic spontaneous urticaria who had a positive basophil activation test, had more symptomatic days/year therefore could need a more continuous clinical follow-up.

**Figure: CU**


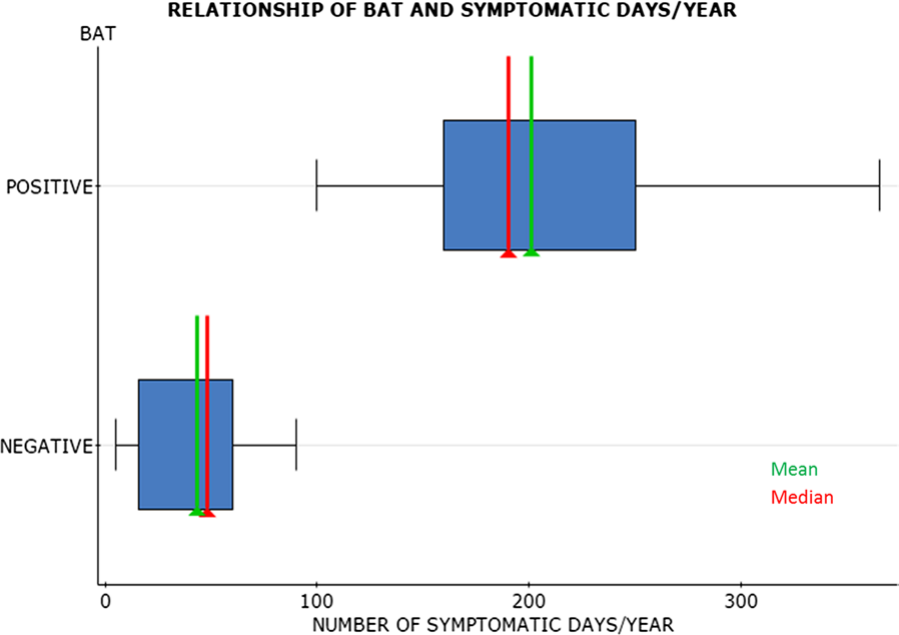


## O18

### Innate immune activation in food reactions in infants with Food Protein Induced Enterocolitis Syndrome

#### Sam Mehr^1^, Eric Lee^1,2^, Peter Hsu^1,2^, Denise Anderson^3^, Emma de Jong^3^, Anthony Bosco^3^, Dianne E. Campbell^1,2^*

##### ^1^Department of Allergy and Immunology, Children’s Hospital at Westmead, Sydney, Australia; ^2^Child and Adolescent Health, University of Sydney, Sydney, Australia; ^3^Centre for Biostatistics, Telethon Kids Institute, Perth, Australia

**Correspondence:** E. Campbell - diannec3@chw.edu.au

*Clinical and Translational Allergy* 2018, **8(Suppl 2):**O18

**Introduction**

FPIES is a non-IgE-mediated food allergy, with a characteristic clinical presentation in early infancy, and a tendency for remission in early childhood. The immune-pathophysiology of the disorder is unclear. Recent reports from Japan and the US have variably reported elevated C-reactive protein, neutrophilia, T cell activation and evidence of innate immune activation.

**Methods**

Infants with a history of FPIES, who met international criteria for diagnosis (1), were invited to undergo an observed oral food challenge to the trigger food or foods from 6 months following the last known clinical episode. Blood samples were collected immediately prior to the challenge and at the onset of a reaction, or at the end of a non-reactive challenge. Each subject included in the study had a paired pre and post challenge sample obtained. Samples were collected prior to any treatment (such as Ondansetron and IV fluids)—or as soon as possible after treatment. Infants were deemed reactive on challenge according to published criteria (2).

Total RNA was isolated from whole blood, and gene expression patterns were profiled by RNA-Seq. Gene network patterns induced by the challenge were compared and contrasted in reactors and non-reactors, and upstream regulator analysis was employed to unveil network driver genes.

**Results**

Paired samples from 36 infants who met inclusion criteria undergoing food challenge were obtained (median age 18 months). 26 infants were tolerant to the food challenge and 10 infants reacted on challenge. Trigger foods included rice (n = 12), cow’s milk (n = 11), egg and meats (n = 9), soy (n = 1), fish (n = 1). Other grains (n = 1) and fruit (n = 1).

198 differentially expressed genes were identified between samples from reactors and non-reactors on food challenge (with a fold change > 1.5, FDR < 0.05). Biological pathways upregulated in food reaction responses included granulocyte adhesion and diapedesis and TREM1 signalling. Network analysis revealed that an innate inflammatory module was upregulated in the responses from reactors, and upstream regulator analysis suggested that the responses were driven by TNF, CFS2 and 3, IL-13, CEBPA, IL-1B, IL-6, TGF-B1 and IFNG.

**Conclusion**

Our results support the proposition that FPIES and food reactions in FPIES are underpinned by activation of the innate immune system. Further scrutiny of the molecular drivers of this response and a more detailed analysis of the response dynamics is underway.

**References**

1. Nowak-Węgrzyn A, et al. International Consensus Guidelines for the Diagnosis and Management of Food Protein-Induced Enterocolitis Syndrome. JACI 2017; In Press.
Lee E et al. Resolution of acute food protein-induced enterocolitis syndrome in children. JACI-IP. 2017;5(2):486–8.

## O19

### Predictive factors of severe allergy to cashew nut in children

#### Clémence Delahaye^1^, Fabien Pelletier^2^, Amandine Luc^3^, Pascale Dumond^2^, Cyril Schweitzer^4^, Etienne Beaudouin^5^, Amandine Chauveau^1^*

##### ^1^Pediatric Allergy Department, Children’s Hospital, University Hospital of Nancy, Vandoeuvre les Nancy, France; ^2^Dermatology department, University Hospital of Besançon, Besançon, France; ^3^ESPRI-BIOBASE Unit-Parc, University Hospital of Nancy, Vandoeuvre les Nancy, France; ^4^Department of Pediatric Lung Function Testing, Children’s Hospital, University Hospital of Nancy; EA 3450 DevAH-Department of Physiology, Faculty of Medicine, University of Lorraine, Vandoeuvre les Nancy, France; ^5^Allergy Department, Regional Hospital Emile Durkheim, Epinal, France

**Correspondence:** Amandine Chauveau - a.chauveau@chru-nancy.fr

*Clinical and Translational Allergy* 2018, **8(Suppl 2):**O19

**Introduction**

The prevalence of cashew nut allergy seems to increase and affects young children. Cashew nut consumption by allergic patients can cause severe reactions, maybe even more severe than peanut consumption does. However, there are no studies on severity criteria for this allergy. The aim of our study is to identify predictive factors of severe allergy to cashew nut in children.

**Methods**

All children who underwent an oral food challenge (OFC) to cashew nut with a positive outcome at the Pediatric Allergy Department of the University Hospital of Nancy between November 2013 and October 2016 were included. The severity of allergic reactions to OFC was analyzed according to 3 criteria: clinical symptoms according to the Astier’s classification, threshold dose and a score combining clinical symptoms and threshold dose. Predictive factors of severe allergy were analyzed by multivariate regression.

**Results**

Among the 94 children with a positive OFC to cashew nut, 31.9% had a history of allergic reaction to cashew nut, 47.9% were asthmatic and 63.8% had at least one other food allergy. The absence of previous allergic reaction to cashew nut (that is to say a sensitization to cashew nut discovered during an allergy testing for another food allergy) and the presence of asthma were associated to anaphylaxis during the OFC (respectively OR = 2.8 [1.1–7.3] and 2.8 [1.2–6.6]). Female gender and size of the skin prick-test to raw cashew nut superior or equal to 8 mm were associated to a low threshold dose (respectively OR = 7.5 [2.0–27.9] and 11.6 [2.6–52.2]). According to the score combining symptoms and threshold dose, only the size of the skin prick-test to raw cashew nut (≥ 8 mm) was a predictive factor of severe allergic reaction (OR = 2, 8 [1, 1–7, 3]).

**Conclusion**

Absence of previous allergic reaction to cashew nut, asthma, femalegender and a skin prick-test to raw cashew nut superior or equal to 8 mm seem to be predictive factors of severe allergy to cashew nut in children.

## O20

### High prevalence of primary eosinophilic gastrointestinal disorders in children who have received food oral immunotherapy

#### Sara Bellón^1^*, Laura Sánchez^1^, Cristina Muñoz^2^, Teresa Bracamonte^1^, Sonia Fernández^3^, Sergio Quevedo^1^, Ana Rayo^2^, Luis Á. Echeverría^1^

##### ^1^Pediatric Allergy Unit, Severo Ochoa University Hospital, Leganés, Spain; ^2^Pediatric Allergy Unit, Villalba General Hospital, Villalba, Spain; ^3^Pediatric Gastroenterology Unit, Severo Ochoa University Hospital, Leganés, Spain

**Correspondence:** Sara Bellón - sara.bellon.alonso@gmail.com

*Clinical and Translational Allergy* 2018, **8(Suppl 2):**O20

**Introduction**

Recently, the relationship between *Primary eosinophilic gastrointestinal disorders* (PEGDs) and *Food oral immunotherapy* (OIT) has been described but large prospective studies are needed to assess the true incidence of PEGDs in this sensitive group of patients.

**Methods**

This is a prospective study in children with milk and/or egg allergy who has been subjected to OIT with these foods during the last 10 years (2006–2016). In all these patients, strict monitoring of new gastrointestinal signs was carried out during both the initial dose escalation phase and the maintenance one.

When patients presented persistent gastrointestinal symptoms during the OIT they were evaluated by our Pediatric Gastroenterology Unit and a digestive endoscopy with biopsy specimens was performed if PEGDs was suspected.

**Results**

In the period 2006–2016, the OIT protocol was applied in 108 cases with cow's milk and 39 cases with egg in our Pediatric Allergy Unit.

In that period, PEGDs were diagnosed in eleven of the 147 global cases of OIT representing 6.8% of the sample (case 8 is not included in prevalence rate because OIT was performed in other centre). A 72% of the patients were males between 3 and 14 years of age.

Eosinophilic oesophagitis (EoE) was diagnosed in nine patients with exclusively oesophageal involvement, while the other two patients were diagnosed with Eosinophilic Gastroenteritis (EoGE) (one with oesophageal and duodenal disease and the other with oesophageal and colon involvement).

Eight of the affected patients had undergone OIT with cow’s milk, two with egg, and one with both foods.

The median time of development of PEGDs after OIT in our patients were 25.7 months (0 months–5 years). The most common initial symptom was abdominal pain (in seven of the eleven cases), followed by vomiting (in six cases) and dysphagia (in five cases).

Eight patients had a good clinical response to the treatment with proton pump inhibitors with or without swallowed corticosteroids, and only three required OIT discontinuation to control the symptoms.

All these results are more specifically showed in Table 1.

**Table 1:**
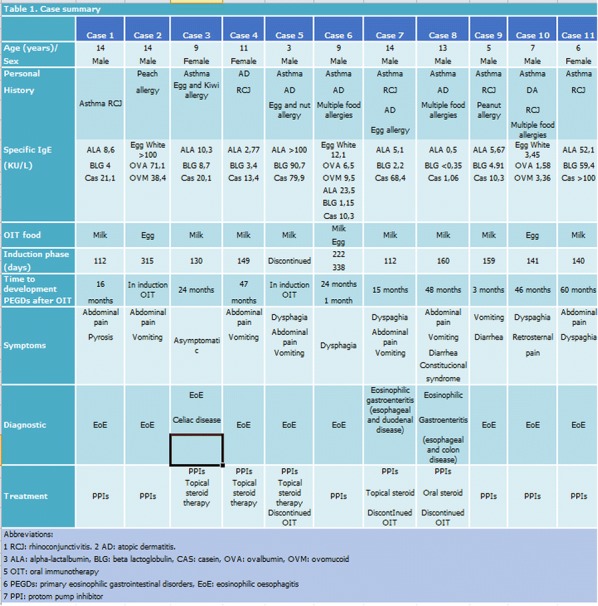
Case summary

**Conclusion:** In our series, the prevalence of PEGDs in patients who have undergone food OIT is 6.8%, that is considerably higher than other studies published to the date. These may be due to the correct and prolonged patient gastrointestinal follow-up.

In our experience, the decision to eliminate the food from OIT should be made on an individual basis, depending on the severity of the condition, the symptoms, evolution and response to other possible treatment alternatives.

## O21

### Caesarean delivery, preterm birth and association with food allergy in children—Swedish nationwide cohort study of 1 million children

#### Niki Mitselou^1^*, Olof Stephansson^3^, Jenny Hallberg^4,5,6^, Erik Melén^4,5^, Jonas F Ludvigsson^1,7^

##### ^1^Department of Pediatrics, Örebro University Hospital, Örebro, Sweden; ^2^Department of Medicine, Solna, Clinical Epidemiology Unit, Karolinska Institutet, Stockholm, Sweden; ^3^Department of Women’s and Children’s Health, Karolinska Institutet, Stockholm, Sweden; ^4^Institute of Environmental Medicine, Karolinska Institutet, Stockholm, Sweden; ^5^Sachs’ Children’s Hospital, Södersjukhuset, Stockholm, Sweden; ^6^Department of Clinical Science and Education, Karolinska Institutet, Stockholm, Sweden; ^7^Department of Medical Epidemiology and Biostatistics, Karolinska Institutet, Stockholm, Sweden

**Correspondence:** Niki Mitselou - nikimitselou@gmail.com

*Clinical and Translational Allergy* 2018, **8(Suppl 2):**O21

**Introduction**

With the exception of heredity and concurrent atopic disease, there are few known risk factors for food allergy in children. We examined the influence of preterm birth, caesarean delivery, low birth weight and intrauterine growth restriction on the risk of food allergy in the offspring.

**Methods**

A nationwide Swedish longitudinal cohort study. We examined 1,086,378 children born in Sweden between 2001 and 2012 using prospectively recorded data from the Swedish Medical Birth Registry and the Patient Registry.

Cox regression estimated Hazard ratios (HRs) for food allergy adjusting for sex, birth weight, gestational age, caesarean delivery, and maternal factors such as country of birth, body mass index, age, smoking, asthma/pulmonary disease, and parity.

**Results**

During follow-up, 26,732 children developed food allergy; of these, 14,534 had at least two Hospital-based diagnoses of food allergy (54.4%). Food allergy was positively associated with caesarean delivery (HR = 1.21; 95% CI = 1.18–1.25) but negatively associated with very preterm birth (HR = 0.74; 95% CI = 0.56–0.98). None of the other exposures could be linked to food allergy. Risk estimates were similar when we restricted our outcome to two diagnoses with food allergy, and did not change with follow-up time (5 years or later: HR for food allergy in children with caesarean delivery = 1.21; 95% CI = 1.12–1.30, and HR for food allergy after very preterm birth = 0.74; 95% CI = 0.38–1.43). There was no difference between boys and girls.

In 100,000 children undergoing caesarean delivery, an extra 78 developed later food allergy compared to the reference group.

**Conclusions**

This study suggests that caesarean delivery increases the risk of food allergy, while very preterm birth is associated with decreased risk of later food allergy.

## O22

### A prospective cross sectional audit of growth parameters of pediatric patients attending food allergy dietetic clinics

#### Carol Fudge^1^, Kate E. Grimshaw^1,2^*, Mark Alderton^3^, Emma Grainger-Allen^3^, Mich Erlewyn-Lajeunesse^3^, Luise V. Marino^1^

##### ^1^Department of Nutrition, Dietetics and Speech and Language Therapy, Southampton Children’s Hospital, Southampton, United Kingdom; ^2^Clinical and Experimental Sciences, Southampton University, Southampton, United Kingdom; ^3^Pediatric Allergy Team, Southampton Children’s Hospital, Southampton, United Kingdom

**Correspondence:** Kate E. Grimshaw - kate.grimshaw@uhs.nhs.uk

*Clinical and Translational Allergy* 2018, **8(Suppl 2):**O22

**Introduction**

The management of allergic reactions involves the exclusion of foods from the diet, predisposing allergic infants/children to inadequate nutritional intake, poor growth and malnutrition. This in turn increases the risk of associated poor developmental and socioeconomic outcomes (e.g. neuro developmental outcomes, increased all-cause mortality in adulthood, reduced scholastic ability, work productivity and lost future earnings). Poor growth and malnutrition has been previously described in food allergic children in the UK [1].

This audit aimed to describe the growth and allergic profile of children seen in the pediatric allergy clinics at Southampton Children’s Hospital.

**Methods**

Between 1st April and 30th September 2016 prospective, cross sectional anthropometric and clinical data were collected from food allergic children attending 4 of the 5 pediatric allergy dietetic clinics held at Southampton Children’s Hospital. These patients represented referrals from primary, secondary and tertiary services. Serial data was not collected for patients seen more than once within the 6 months period. Data was entered into an access database designed for the purpose and were analysed using SPSS version 17.

**Results**

236 children with IgE and non IgE mediated allergies were included. More females were seen than males with ages ranging from 3 to 177 months (mean 25.0) (Table 1).

**Table 1:** Characteristics of children included in Audit

	All	IgE mediated	Non IgE mediated	New referral	Review
Mean age in months	25.04	28.2	21.24	22.63	27.6
Male gender	106	34	76	54	51
New referral	121	42	84	–	–
Three or more food allergies	53	38	32	22	31
Mean weight Z	0.2012	0.1674	0.2076	0.1849	0.21
Mean height Z	− 0.0872	− 0.1670	− 0.0730	− 0.1514	− 0.0247

The numbers of diagnosed food allergy ranged from 1 (n = 123) to 7 (n = 3), the most common being Cow’s milk (91.5%)followed by hens egg, soya, peanuts, tree nuts, wheat, fish, sesame, oats, lupin, corn, kiwi and legumes.

1.3% of children seen had wasting (weight for height ≤ − 2 z scores) and 5.9% had stunting (height for age ≤ − 2 z scores). The rate for low height for age z scores (HAZ) was higher for new referrals (8.3%) than for follow ups (3.5%) (p = 0.097 (ns)), indicating a significant improvement in HAZ score following dietetic intervention. There was a trend for HAZ < − 2 scores to be associated with having 4 or more food allergies (p = 0.079 (ns)). Children with Non IgE milk allergy (new/review) had the highest rates of low HAZ score at 7.7%.

**Conclusion**

Although growth problems are not a major issue in our food allergy population, the cross sectional prevalence of stunting for new referrals to the dietetic allergy service was slightly higher than would be expected within a population standard of around 5% [2] and was highest amongst children with Non IgE mediated milk allergy. Growth status improved upon dietetic intervention.

**References**

1. Meyer R, De KC, Dziubak R, Venter C, Dominguez-Ortega G, Cutts R, et al. Malnutrition in children with food allergies in the UK. J Hum Nutr Diet. 2013.
2. World Health Organisation. Global database on Child growth and malnutritioni: Child growth indicators and their interpretation. World Health Organisation 2012

## O23

### Food allergy at 12 years of age in western Sweden—Risk factors and protective factors

#### Ivar Orn Clausen^1^*, Emma Goksor^2^, Bernt Alm^2^, Goran Wennergren^2^

##### ^1^Department of Medicine, University of Iceland, Reykjavík, Iceland; ^2^Department of Pediatrics, University of Gothenburg, Queen Silvia Children’s Hospital, Gothenburg, Sweden

**Correspondence:** Ivar Orn Clausen - ioc1@hi.is

*Clinical and Translational Allergy* 2018, **8(Suppl 2):**O23

**Introduction**

The prevalence of allergy has increased in the western world over the last decades. Multiple factors have been identified as increasing and reducing the risk. Rural living has been associated with less risk of allergic disease, in line with the hygiene hypothesis.

The goal of this study was to estimate possible risk factors and protective factors for food allergy in 12-year-old children living in western Sweden.

**Methods**

The data was obtained from a prospective, longitudinal cohort study in western Sweden. Questionnaires were sent out when the children were 6 months, 1, 4.5, 8 and 12 years of age. Additional information was received from the Swedish Medical Birth Register. Children with possible food allergy and probable food allergy were identified and allocated into two groups for further examination. Possible food allergy was defined as those who reported both doctor diagnosed food allergy and symptoms of food allergy in the last 12 months. Probable food allergy was defined as those with reported doctor diagnosed food allergy, symptoms of food allergy as well as reported sensitization to the food they reported symptoms from. Early risk and protective factors were identified and evaluated.

**Results**

A total of 3637 families answered the questionnaires distributed at 12 years and thus the response rate was 76.1% (3637/4777). Of these, 52.4% (1895) were boys. The prevalence of possible food allergy was 6.4% (n = 230) and of probable food allergy was 3.0% (n = 110). In a multivariate analysis, heredity for atopic diseases (adjusted OR (aOR) 1.8; 95% confidence interval (CI) 1.1–2.8) and early eczema (aOR 4.0; 2.7–6.1) were independent risk factors for probable food allergy.

Rural living at 6 months of age (aOR 0.45; 0.24–0.83) and fish consumption more than once a month at 1 year (aOR 0.47; 0.25–0.87) decreased the risk of probable food allergy at 12 years, independently of heredity and socioeconomic factors.

**Conclusion**

In our study, we show that rural living at 6 months of age and fish consumption more than once a month at 1 year of age, reduce the risk of having food allergy at 12 year of age and that heredity and early eczema are independent risk factors.

## O24

### Using clinical decision support to promote prevention of peanut allergy guideline adherence: a US pilot project

#### Lucy Bilaver^1^*, Monika Martusiewicz^2^, James Shea^2^, Lauren Kao^1^, Matthew Davis^1,2^, Ruchi Gupta^1,2^

##### ^1^Department of Pediatrics, Northwestern University, Chicago, IL, United States of America; ^2^Lurie Children’s Hospital, Chicago, IL, United States of America

**Correspondence:** Lucy Bilaver - l-bilaver@northwestern.edu

*Clinical and Translational Allergy* 2018, **8(Suppl 2):**O24

**Introduction**

The groundbreaking Learning Early About Peanut allergy (LEAP) trial demonstrated that early introduction of peanut products into the diet of high risk infants 4–11 months old reduced the subsequent incidence of PA in early childhood by 81% [1]. Consequently, in 2017 the National Institute of Allergy and Infectious Disease coordinated the publication of *The Addendum guidelines for the prevention of peanut allergy in the United States* [2] [“Prevention of Peanut Allergy (PPA) guidelines”], recommending dietary introduction of peanut products during infancy. The new PPA guidelines present an immediate clinical challenge for pediatricians to assess risk by identifying infants with severe eczema and/or egg allergy and subsequently test, refer, and/or counsel caregivers. Clinical decision support (CDS) tools integrated within electronic health record (EHR) systems is an evidenced-based approach to facilitate adherence to clinical guidelines. The objective of this project was to assess adherence to PPA guidelines using a CDS recently implemented in one large, US pediatric residency clinic and to validate the pediatrician’s interpretation of high risk infants.

**Methods**

We analyzed data for 51 infants seen for 4, 6, and 9 month well-child visits between June 7, 2017 and August 21, 2017. Data was pulled from an EHR that had implemented a CDS to support PPA guideline adherence. The CDS included best practice advisory, order sets, and additions to the note template around evaluation of risk status and anticipatory guidance. Chart review was conducted by trained pediatric residents to validate risk categorization.

**Results**

Of the 51 infants seen for well-child visits during the observation period the best practice advisory was triggered for 11 infants (21.6%). Pediatricians identified two infants with severe eczema, 38 with neither severe eczema no egg allergy, and 11 were left unidentified. Adherence to the guidelines was 66% among infants with no risk factors and 100% among those with severe eczema. Chart review revealed that the two infants (3.9%) identified with severe eczema were prescribed topical corticosteroids and calcineurin inhibitors; however, another eight infants (15.7%) were also prescribed topical corticosteroids or calcineurin inhibitors but were identified as having mild or moderate eczema by the pediatrician.

**Conclusion**

Clinical decision support was an effective method to promote PPA guideline adherence. Clinical judgement was an important factor in identifying infants at high risk for peanut allergy as use of prescription medications alone did not discriminate between those with severe versus mild to moderate eczema.

**References**

1. Du Toit G, Roberts G, Sayre PH, Bahnson HT, Radulovic S, Santos AF, et al. Randomized trial of peanut consumption in infants at risk for peanut allergy. N Engl J Med. 2015;372(9):803–1310.1056/NEJMoa1414850.
2. Togias A, Cooper SF, Acebal ML, Assa’ad A, Baker JR, Jr., Beck LA, et al. Addendum guidelines for the prevention of peanut allergy in the United States: Report of the National Institute of Allergy and Infectious Diseases-sponsored expert panel. J Allergy Clin Immunol. 2017;139(1):29–4410.1016/j.jaci.2016.10.010.

## D2

### Quality of life improves in children with persistent egg allergy after oral immunotherapy with egg

#### Sonia Vázquez-Cortés*, Guadalupe Marco-Martín, Inmaculada Cerecedo, Mónica Rodríguez-Álvarez, Victoria Fuentes-Aparicio, Ximena Larco-Rojas, Montserrat Fernández-Rivas

##### Allergy Department, Hospital Clinico San Carlos IdISSC, Madrid, Spain

**Correspondence:** Sonia Vázquez-Cortés - sonia.vazquez.cortes@gmail.com

*Clinical and Translational Allergy* 2018, **8(Suppl 2):**D2

**Introduction**

Food allergy compromises patient's quality of life (QoL). Oral immunotherapy (OIT) is effective in patients with persistent food allergy.

The objective of this study is to evaluate if OIT modifies QoL in children from 8 to 12 years of age with persistent egg allergy.

**Methods**

We performed a prospective cohort study in patients with persistent egg allergy included in our protocol of egg oral immunotherapy. We analyzed data of 18 patients aged between 8 and 12 years that completed the Food Allergy Quality of Life Questionnaire (FAQLQ) and the Food Allergy Independent Measure (FAIM) before starting the OIT (v1), after 6 months (v2) and 12 months after (v3) finishing the OIT.

Statistical analyses were carried out with nonparametric matched-pair methods (Wilcoxon and Kendall) using Stata.

**Results**

50% of patients were female and 61% suffered from other food allergies, most of them to tree nuts (81.8%).

We found an improvement trend in FAQLQ score statistically significant since v6 onward (p = 0.004). We observed a decrease in FAQLQ score in v2 and v3, statistically significant (v2: 0.956 points p = 0.03; v3: 1.58 points, p = 0.02). In the domain analysis, we observed a score decrease, all of them clinically relevant (>=0.5 points) and statistically significant at 1 year (p < 0.05), except in the Dietary Restrictions domain, where we did not find this statistical significance, although the decrease was clinically relevant.

Regarding FAIM, we found a slight increase in v6 (0.17 points, p > 0.05). At v12 we observed a decrease in score statistically significant (0.417 points, p = 0.03).

**Conclusion**

OIT improves QoL in children between 8 and 12 years of age with egg allergy, even those suffering from allergy to other food.

## D3

### “Are we there yet?”: choosing the best time to offer baked milk and egg challenges to milk and egg allergic children

#### Nikita Ohayon*, Shalini Thiruvarudchelvam, Jason Ohayon

##### Department of Pediatrics, McMaster University, Hamilton, Ontario, Canada

**Correspondence:** Nikita Ohayon - nsohayon@gmail.com

*Clinical and Translational Allergy* 2018, **8(Suppl 2):**D3

**Introduction**

The option of baked milk (BM) and baked egg (BE) challenges allow for desensitization in milk and egg allergic children, respectively. Serum specific IgE (sIgE) measurements to milk (< 10 kU/L) and egg (< 5kU/L) have been suggested as guides in selecting children for desensitization.

**Methods**

Retrospective chart review was performed in a community Allergy clinic on children who participated in BM and BE oral challenges. Data on demographics, allergic measurements and outcome of oral challenges was collected. Data on milk and egg allergic children who were not selected for oral challenge was collected for comparison.

**Results**

Forty five children with milk allergy were identified over a 40 month period (Jan 2014 to Apr 2017). Average age at diagnosis was 15 months. Thirty-two of 45 children were male (71%). Average skin (STz) size at diagnosis was 5.8 mm to milk. BM challenges were offered to 25/45 (56%). Average time from diagnosis of milk allergy to BM challenge was 38 months. At challenge date, average STz was 6.7 mm with average sIgE of 3.29 kU/l for milk and 2.52 kU/L for casein. Twenty-one of 22 (95%) children passed their BM challenge. Twenty of 45 (44%) were not challenged, based on their average STz of 9.2 mm and sIgE to milk 29.6 kU/L and casein 38.16.

Fifty-three children with egg allergy were identified over the same time period. Average age at diagnosis was 13 months. Average STz at diagnosis was 6.9 mm for egg white (EW). Twenty-five of 53 children (47%) were offered BE oral challenge. Average time from diagnosis to BE challenge was 37 months. At challenge date, average STz was 5 mm for EW and sIgE was 0.7 kU/L. Twenty-three of 25 (92%) children passed. Twenty-eight of 53 (53%) were not challenged based on average STz of 8.1 mm and sIgE 27.9 to EW.

**Conclusions**

BM and BE challenges were safe in milk and egg allergic children respectively after an average of 2.5 years from time of diagnosis. Children chosen for challenge were within suggested sIgE cutoff levels. STz in the “passed” milk challenge group was not different to non-challenged group. The high rate of successful challenges to both milk and egg, suggests that the sIgE levels chosen may be too low. Children in the non-selected group may therefore be included sooner for BM/BE challenges.

## D4

### Pediatric anaphylaxis in a food allergy consultation

#### Rosa-Anita Fernandes*, Emília Faria, Celso Pereira, Ana Todo-Bom, Isabel Carrapatoso

##### Coimbra University Hospital, Coimbra, Portugal

**Correspondence:** Rosa-Anita Fernandes - rosa.anita.fernandes@gmail.com

*Clinical and Translational Allergy* 2018, **8(Suppl 2):**D4

**Introduction**

Anaphylaxis is a potentially fatal allergic reaction and its prevalence has been increasing. Food allergy is the commonest cause of anaphylaxis in children, especially in preschool age. The aim of this study was to characterize the pediatric population with food induced anaphylaxis followed in our Food Allergy outpatient department (FAOD) over a 16 months period.

**Methods**

Retrospective analyses of medical charts and electronic records of all children and adolescents with food-related anaphylaxis observed in our FAOD from January 2016 to April 2017. Patients were categorize into clusters according to the culprit food in the first anaphylactic episode.

**Results**

35 patients were included, 20 male and 15 female. The mean age was 5.8, the earliest anaphylaxis was at 14 days old (cow’s milk) and the majority of reactions were with cow’s milk (n = 9), egg (n = 6) and fish (n = 6). Reactions to milk were more common in infants (88%). Egg allergy was more frequent in pre-school and school-age children. In adolescents, no specific allergy pattern was found. Peanut was the culprit food in only 1 patient that also reported symptoms with fresh fruits and was sensitized to Pru p 3. Multiple combinations of symptoms were observed, the most frequent were urticaria (82.9%), angioedema (57%) and respiratory symptoms (62.9%). Thirty-one patients were observed in the emergency department during anaphylaxis, but few (n = 6) were treated with epinephrine. Most of the patients were atopic (86%) and asthma and/or rhinitis were the more frequent comorbidities. Only 3 patients had symptoms with other foods. The presence of cofactors was observed in only 1 patient, a 16-year-old male, sensitized to Pru p 3, with exercise-induced anaphylaxis to apple.

**Conclusion**

In our pediatric population, the main triggering agent of food-dependent anaphylaxis in infants was milk and egg, as reported in previous studies. Differing from other studies, the prevalence of reactions due to peanut was low. Atopy was present in almost all patients. Epinephrine is underused, as reported by others, which represents an important risk of more severe reaction in this age group.

## D5

### Review of oral food challenge tests in children

#### Belén García Avilés^1^*, Nuria Marco Lozano^2^, Teresa Toral Pérez^3^, Luis Moral Gil^3^, Cristina Montahud Posada ^4,^ María Caballero Caballero^4^, Patricia Martínez Rovira^1^, Teresa Atienza Almarcha^1^, Mª José Forniés Arnáu^5^, Cristina González Toro^5^, Jesús Garde Garde^6^

##### ^1^Hospital Clínico Universitario de San Juan de Alicante, Spain; ^2^Hospital Vega Baja de Orihuela, Spain; ^3^Hospital General Universitario de Alicante, Spain; ^4^Hospital Universitario del Vinalopó de Elche, Spain; ^5^Hospital General Universitario de Elda, Spain; ^6^Hospital General Universitario de Elche, Spain

**Correspondence:** Belén García Avilés - bgarciaviles@gmail.com

*Clinical and Translational Allergy* 2018, **8(Suppl 2):**D5

**Introduction**

The objective of our study, is to analyze the results of oral food challenge tests (OFCT) in children

**Methods**

Retrospective review of OFCT performed in pediatric patients in 6 Hospitals during the years 2014–2015. Patient-related data, clinical history, and test results were collected. Data were analyzed using the SPSS 17.0 program.

**Results**

756 OFCT performed in 548 patients (61%males, median age 4 years, mode 1 year). Foods most frequently tested: egg (34%), nuts (23%), cow’s milk (17%) and fish (9%). OFCT were positive (onset of allergy symptoms) in 167 patients (22%), negative in 576 (76%) and inconclusive in 13 (2%). Tests were more frequently positive (p < 0.05) for egg, walnut, and in patients with higher levels of specific IgE, with a history of gastrointestinal allergy symptoms or with atopic dermatitis and approached significance for milk (p = 0.063) or in patients reporting a history of anaphylaxis (P = 0.094). OFCT were significantly less positive for almonds or with foods not previously consumed because sensitization was detected prior to introduction. Most frequent signs and symptoms in positive tests were cutaneous (68%), gastrointestinal (48%) and oral pruritus (31%). No treatment was required in 32% of the positive tests and adrenaline was administered in 10%; no one required Hospitalization.

Twenty patients presented anaphylaxis during the test, more frequently in those who had previous anaphylaxis (p = 0.01) and with walnuts (p = 0.068). A total of 183 OFCT were performed due to sensitization detected prior to the introduction of the food, always being negative with almond (p = 0.028) and positive more commonly with higher specific IgE (p = 0.008), atopic dermatitis (p = 0.076) or with egg (p = 0.082).

**Conclusions**

OFCT have an acceptable safety profile in our population, which reaffirms its irreplaceable usefulness in the study of pediatric patients with suspected or known food allergy. The high rate of positve tests should not be an impediment to its performance.

## D6

### Impact of diagnostic testing for food allergy on quality of life and health-related costs: a systematic review

#### Kansen Hannah M^1^*, Le Thuy-My^2^, Meijer Yolanda^1^, Knulst André C^2^, Van der Ent Cornelis^1^, K van Erp Francine^1^

##### ^1^Wilhelmina Children’s Hospital, University Medical Center, Utrecht, Netherlands; ^2^University Medical Center, Utrecht, Netherlands

**Correspondence:** Kansen Hannah M - h.m.kansen-2@umcutrecht.nl

*Clinical and Translational Allergy* 2018, **8(Suppl 2):**D6

**Introduction**

There is increasing interest in using alternative diagnostic strategies for food allergy, thereby limiting the burden compared to double-blind placebo-controlled food challenges (DBPCFC). However, uncertainty remains regarding the impact of these tests on quality of life (QoL) and health-related costs. The current study aims to systematically review literature regarding this matter.

**Methods**

Electronic databases (MEDLINE, EMBASE, Cochrane Library, NIHR) were searched until January 2017. Studies were included if diagnostic testing was performed in patients with suspected food allergy and changes in either QoL or costs were reported. Validity was assessed using validated checklists for critical appraisal. Study selection and validity assessment were performed independently by two researchers.

**Results**

A total of 1.239 original references on QoL and 1.994 on costs were identified.

In all seven identified references on QoL, the diagnostic test under study was an oral food challenge. Two studies reported no substantial improvement in food-allergy related QoL following a food challenge. However, five references found significantly improved QoL in challenged patients compared to unchallenged irrespective of the outcome.

Four of seven identified studies on costs used a Markov model to investigate cost-effectiveness of diagnostics in patients with suspected peanut allergy. These models estimated that using sIgE to peanut components results in increased QoL and reduced costs compared to DBPCFC. Another study reported a 63% decrease in unnecessary elimination diets when sIgE to allergen components would be used in primary care. A prospective study reported that indirect socioeconomic costs decreased following DBPCFC in all patients, while direct costs increased in food allergic patients. Finally, one study constructed a model to assess budget impact of diagnosis and treatment of cow’s milk allergy in the Netherlands from the perspective of the health care insurers. Total annual costs of managing all new CMA sufferers expected in 1 year (n = 4382) were estimated at €11.3 million. Costs would increase substantially (by ~ €1.9 million) if DBPCFC would be performed in all patients.

**Conclusion**

sIgE to allergen components has the potential to be more cost-effective compared to DBPCFC, but valid data on the impact on QoL are lacking. The impact of food challenges on QoL varies across studies, probably explained by study heterogeneity. The present review indicates the need for a prospective study on the impact of (alternative) diagnostic testing on quality of life and costs in patients with food allergy, as all studies identified were considered of low to moderate validity.

## D7

### IgE-mediated allergy to baked and regular cow’s milk and egg: learning from oral food challenges

#### Natalia Cartledge*, Sophia Lazenby, Rachel De Boer, Susan Chan, Alexandra Santos

##### Children’s Allergy Service, Guy’s and St Thomas’ Hospital, London, United Kingdom

**Correspondence:** Natalia Cartledge - Natalia.cartledge@gmail.com

*Clinical and Translational Allergy* 2018, **8(Suppl 2):**D7

**Introduction**

The majority of children with IgE-mediated cow’s milk and egg allergies tolerate baked milk and egg and outgrow their allergy over time. The primary aim of this study was to determine optimal identifiers of children who may be able to tolerate (baked and regular) milk or egg.

**Methods**

Medical records of oral food challenges (OFC) to baked and regular cow’s milk and hen’s egg performed over a 2-year period (Jan 2014-Dec 2015) in the Pediatric Allergy Department were reviewed. Demographic and clinical characteristics, results of skin prick test (SPT), specific IgE (sIgE) to milk and egg and OFC were recorded. Statistical analyses were performed using IBM SPSS v21.

**Results**

Following assessment in the Pediatric Allergy clinic, 126 children underwent OFC to milk (79 (73%) to baked and 47 (37%) to regular milk) and 177 children underwent OFC to egg (124 (70%) to baked and 53 (30%) to regular egg). Overall, 22 patients (15%) failed OFC to milk and 46 (26%) to egg. The majority of the reactions were mild; only 2 (1.6%)children who reacted to milk and 3 (1.7%) children who reacted to egg were treated with intramuscular adrenaline. There was no significant difference in the wheal size of SPT to fresh milk (p = 0.374, area under the ROC curve (AUC) = 0.419) between children who passed or failed OFC to baked or regular milk. Children who reacted to baked milk had a higher SPT to milk extract (p = 0.043, AUC = 0.698) and higher sIgE to milk (p = 0.015, AUC = 0.733). To predict reactivity to regular milk, sIgE (p = 0.012, AUC = 0.782) performed better than SPT to milk extract (p = 0.642, AUC = 0.434). Children who reacted to baked egg had higher SPT to egg extract (p = 0.044, AUC = 0.613) and to raw egg (p = 0.007, AUC = 0.659) and higher sIgE to egg white (p = 0.002, AUC = 0.741) than children who passed the OFC. To predict reactivity to regular egg, SPT to raw egg (p = 0.003, AUC = 0.883) performed better than SPT to egg extract (p = 0.223, AUC = 0.673) and than sIgE to egg white (p = 0.149, AUC = 0.761).

**Conclusion**

The majority of patients were referred for OFC to baked rather than regular milk and egg. Specific IgE was a better predictor of clinical reactivity to baked and regular milk and to baked egg than SPT. SPT to raw egg was the best predictor of reactivity to regular egg.

## D8

### Amino-acid based formula including synbiotics effectively modulates gut microbiota of non-IgE mediated Cow’s Milk Allergic infants

#### Harm Wopereis^1^*, Marleen T. J. van Ampting^1^, David C. A. Candy^2^, Diego Peroni^3^, Yvan Vandenplas^4^, Adam T. Fox^5^, Neil Shah^6^, Aysun C. Yavuz^1^, Lucien F. Harthoorn^1^, Louise J. Michaelis^7^, Jan Knol^1^, Christina E. West^8^

##### ^1^Nutricia Research, Utrecht, The Netherlands; ^2^Royal Alexandra Children’s Hospital, Brighton, United Kingdom; ^3^University Hospital Verona, Verona, Italy; ^4^Vrije Universiteit Brussel, Brussels, Belgium; ^5^Guy’s and St Thomas’ Hospitals NHS Foundation Trust, London, United Kingdom; ^6^Great Ormond Street Hospital, London, United Kingdom; ^7^Great North Children’s Hospital, Newcastle upon, United Kingdom; ^8^Umeå University, Umeå, Sweden

**Correspondence:** Harm Wopereis - harm.wopereis@danone.com

*Clinical and Translational Allergy* 2018, **8(Suppl 2):**D8

**Introduction**

A healthy breastfed infant is characterized by optimal immune maturation and specific gut microbiota development [1]. Infants who suffer from severe cow’s milk allergy (CMA) often rely on cow’s milk protein avoidance and, when breastfeeding is not possible, on specialised infant formulas such as amino-acid based formulas (AAF). In this study, we investigated the gut microbiota development of infants with CMA receiving an AAF including specific prebiotic and probiotic ingredients (synbiotics).

**Methods**

In a prospective, randomized, double-blind controlled study (registered as NTR3979), full-term infants with suspected non-IgE mediated CMA received an AAF (control; n = 36) or an AAF including synbiotics specifically designed for dietary management of CMA (oligofructose, inulin, *Bifidobacterium breve* M-16 V) (test; n = 35) for 8 weeks (8w). Healthy breastfed infants, age-matched to CMA infants at 8w, were used as reference group. After 8w, CMA infants continued to use study product if advised by clinician. Microbial composition of faecal samples collected at baseline, 8w, 12w and 26w were analysed by 16S ribosomal-DNA sequencing and fluorescent in situ hybridization (FISH). Statistical analysis of sequencing data involved mixed models and the Principal Response Curves (PRC) technique to assess time-dependent treatment effects [2].

**Results**

At inclusion the mean age (± SD) of CMA infants (n = 71) was 6.00 ± 2.98 months. At 8w, there was no difference in gut microbial diversity between test and control group. In both groups diversity increased over time (from baseline until 26w) characterised by a more gradual increment in test compared to control (Shannon index, difference = − 0.026, P = 0.005). PRC analysis revealed that the gut microbial composition changed significantly over time in test vs. control (Monte Carlo Permutation Test, 1000 permutations, P = 0.001), which was driven by significantly increased relative abundances of *Bifidobacterium* spp. and *Veillonella* sp., and significantly decreased relative abundances of *Alistipes* sp. and several species within the family of *Lachnospiraceae*.

**Conclusion**

We showed that the AAF including specific synbiotics leads to a selective enhancement of *Bifidobacterium* spp. and decreased levels of species of *Lachnospiraceae*. These results confirm previously reported results by FISH quantification of both bacterial groups, which were shown for test to approximate the levels observed in the healthy breastfed reference group at the 8w primary endpoint [3]. Overall, these results show that the AAF including specific synbiotics effectively modulates the gut microbiota development of non-IgE mediated CMA infants to a more healthy profile.

**Acknowledgements**

We would like to thank the ASSIGN study group, and all participants and their families.

**References**

1. Wopereis, H., et al., *The first thousand days*—*intestinal microbiology of early life: establishing a symbiosis.* Pediatr Allergy Immunol, 2014. **25**(5): p. 428–38.
2. van den Brink, P. J., et al., *Principal response curves technique for the analysis of multivariate biomonitoring time series.* Environ Monit Assess, 2009. **152**(1–4): p. 271–81.
3. Wopereis, H., et al., *Gut Microbiota Composition of Non*-*IgE Mediated Cow’s Milk Allergic Infants before and after Dietary Management with a Synbiotics*-*Supplemented Amino Acid*-*Based Formula.* Journal of Allergy and Clinical Immunology, 2017. **139**(2): p. Ab53-Ab53.

## D9

### Participatory action research to disseminate EAACI food allergy and anaphylaxis guidelines and raise whole school food allergy awareness via development of a practical online ‘self-service’ process toolkit for UK secondary schools

#### Jennette Higgs1*, Kathryn Styles^1^, Sarah Bowyer^1^, Amena Warner^2^, Carla Jones^2^

##### ^1^Nutrition and Dietetic Consultancy, Food To Fit Limited, Northamptonshire, United Kingdom; ^2^Allergy UK, Sidcup, Kent, United Kingdom

**Correspondence:** Jennette Higgs - jennette@foodtofit.com

*Clinical and Translational Allergy* 2018, **8(Suppl 2):**D9

**Introduction**

Recent deaths in UK schools have reinforced the urgency for embedding whole school allergy awareness to minimise risk [1]. Whilst essential, training for emergency AAI administration is not an adequate safeguard in isolation for the school environment [2]. Participatory action research [3–4] is a powerful symbiotic method employing a series of iterative loops of diagnosing, planning, taking action and evaluating to gradually create a contextually appropriate solution to a problem [5]. This analytical framework helps schools *immediately* begin to improve their whole school practices, whilst working towards implementation of school-specific action plans and a robust allergy policy, embracing best practice [2, 6–7].

Focusing on secondary schools recognises the high risk of this age group [8–10].

**Methods**

A key stakeholder cross-disciplinary workshop in 2015 initiated a participatory action research programme to develop resources, enabling hands-on, school-led, iterative evaluation of support materials at every stage of the process towards developing whole school allergy awareness. User feedback justified modification, ready for re-testing in three different case study schools. This co-productive approach resonates with school systems. Self-service piloting by new schools, with remote support and monitoring by us will proof-test the toolkit ready for release in downloadable format.

**Results**

1. Refined process toolkit now available for schools to work through the consecutive stages ‘in-house’[11].
2. Toolkit comprises template meeting agendas, activities and resources.
3. Staged process drives whole school risk assessment via a **S**chool **A**llergy **A**ction **G**roup, enabling education of ALL staff, catering, parents and pupils; creation of bespoke action plans, designed to address identified priority issues for their school; and development of best practice policy.
4. Continuous review process is instilled into toolkit for risk reduction.
5. Figure 1 illustrates example outcomes.

**Fig. 1:**
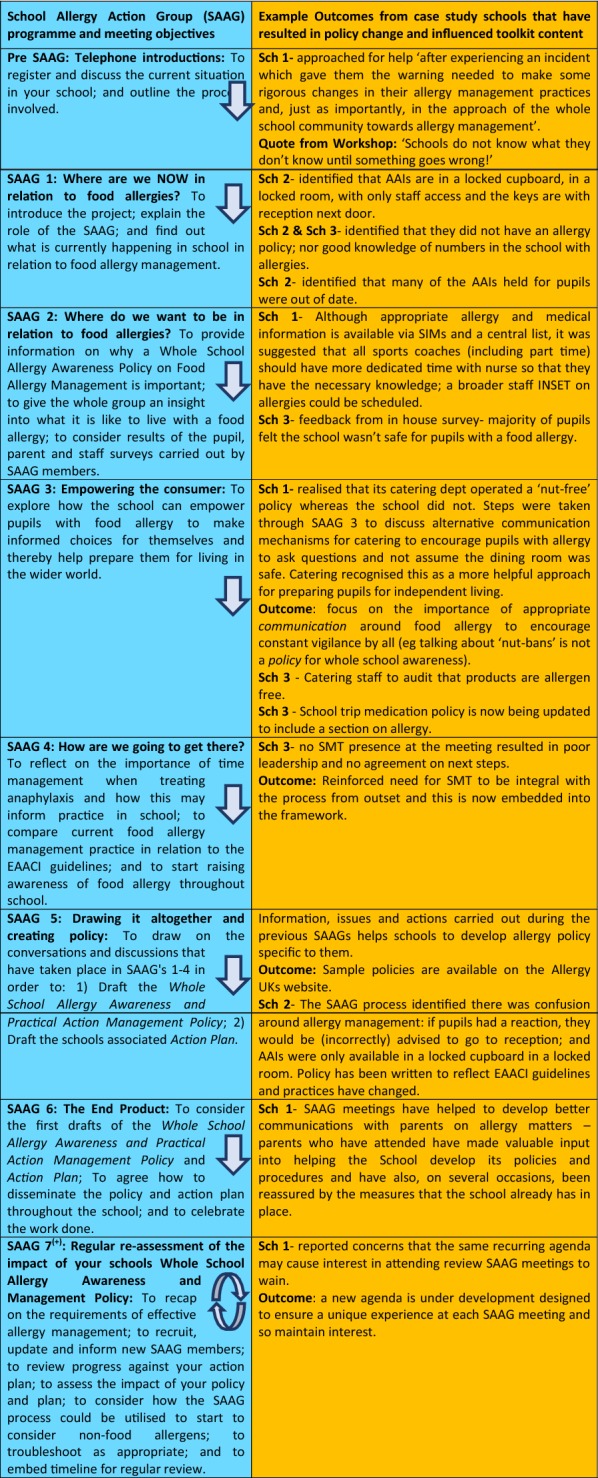
Whole school allergy awareness process stages with outcomes

**Conclusion**

Live testing in school, with concurrent evaluation and modification of materials has enabled faster development of effective tools for wide scale dissemination. Following the remote piloting and evaluation it will be made available as a free download on https://www.allergyuk.org/. As an online process toolkit, continued evolution based on best practice guidance is automatically picked up by schools, since regular review has been built in.

Improving awareness of the whole school community has the potential to reduce risk of allergic reactions and empower secondary school pupils with allergies, to live independent lives.

**References**

1. BBC. 12th May 2017. *Bow pupil died from allergic reaction.* Available from: http://www.bbc.co.uk/news/uk-england-39896499 [Accessed 17th August 2017].
2. Muraro A, Agache I, Clark A, Sheikh A, Roberts G, Akdis CA, et al. EAACI Food Allergy and Anaphylaxis Guidelines: managing patients with food allergy in the community. *Allergy.* 2014;69: 1046–1057.
3. Reason P, Bradbury H. (eds) *The Sage Handbook of Action Research: Participative Inquiry and Practice.* London: Sage; 2008.
4. Brydon-Miller M, Kral M, Maguire P, Noffke S, Sabhlok A. Jazz and the Banyan Tree: Roots and Riffs on Participatory Action Research. In: Denzin NK, Lincoln YS. (eds.) *The Sage Handbook of Qualitative Research* 4th edition. Thousand Oaks, California: Sage; 2011. p. 387–401.
5. Lewin, K. Action research and minority problems. *Journal of Social Issues*. 1946; 2(4): 34–46.
6. REGULATION (EU) No 1169/2011 OF THE EUROPEAN PARLIAMENT AND OF THE COUNCIL of 25 October 2011 on the provision of food information to consumers, amending Regulations (EC) No 1924/2006 and (EC) No 1925/2006 of the European Parliament and of the Council, and repealing Commission Directive 87/250/EEC, Council Directive 90/496/EEC, Commission Directive 1999/10/EC, Directive 2000/13/EC of the European Parliament and of the Council, Commission Directives 2002/67/EC and 2008/5/EC and Commission Regulation (EC) No 608/2004.
7. Department for Education. Supporting pupils at school with medical conditions. Statutory guidance for governing bodies of maintained schools and proprietors of academies in England. Available from: https://www.gov.uk/government/uploads/system/uploads/attachment_data/file/484418/supporting-pupils-at-school-with-medical-conditions.pdf [Accessed 17th August 2017].
8. Branum A, Lukacs S. Food allergy among US children: Trends in prevalence and hospitalizations. NCHS data brief, no 10. Hyattsville, MD: National Center for Health Statistics; 2008.
9. Marrs T and Lack G. Why do few food‐allergic adolescents treat anaphylaxis with adrenaline?–reviewing a pressing issue. Pediatric Allergy and Immunology, 2013; 24(3), pp. 222–229.
10. Pereira B, Venter C, Grundy J, Clayton C, Arshad SH, Dean T. Prevalence of sensitization to food allergens, reported adverse reaction to foods, food avoidance, and food hypersensitivity among teenagers. J Allergy Clin Immunol. 2005;116: 884–892.
11. Allergy UK. *For schools.* Available from: https://www.allergyuk.org/information-and-advice/for-schools [Accessed 17th August 2017].

## D10

### Adrenaline auto-injectors prescribing habits in the UK

#### Brenda DeWitt, Donald Hodge*

##### Pediatric Allergy Department, Leeds Children’s Hospital, Leeds, United Kingdom

**Correspondence:** Donald Hodge - donaldhodge@nhs.net

*Clinical and Translational Allergy* 2018, **8(Suppl 2):**D10

**Introduction**

In 2014 the Medicines and Healthcare Products Regulatory Agency (MHRA) published a drug safety update recommending that people who have been prescribed an adrenaline autoinjector (AAI) should carry two at all times. In 2016 the British Society of Allergy and Clinical Immunology (BSACI) published a guideline ‘‘Prescribing an adrenaline auto-injector’ recommending that the number of AAI’s anyone should carry should be based on an individual risk assessment [1].

If the BSACI guideline is followed and the individual risk assessment is that only one AAI is required, it is essential to ensure that the correct dose AAI is prescribed.

The current BSACI recommended doses of adrenaline available for self-administration are shown in Table 1.

**Table 1:** Recommended doses of adrenaline available for self-administration [1]

Adult or child > 12 years–0.5 mg [0.3 mg more appropriate for a smaller child > 12 years]
Adult, adolescent or child > 30 kg–0.3 mg
Children 15–30 kg–0.15 mg [0.3 mg may be more appropriate for some children, for example over 25 kg]
Children < 15 kg (unlicensed)–0.15 mg
[EAACI Anaphylaxis guideline recommends > 25 kg : 0.3 mg (2)]

The British National Formulary (BNF) and the Resuscitation Council UK dosages of adrenaline to be administered intramuscularly by healthcare professionals are shown in Table 2.

**Table 2:** The British National Formulary (BNF) and the Resuscitation Council UK dosages of adrenaline to be administered intramuscularly by healthcare professionals*

Adult or child > 12 years 0.5 mg (= 0.5 mL) [*0.3 mg if child small or pre-pubertal]
Children aged 6–12 years 0.3 mg (= 0.3 mL)
Children < 6 years 0.15 mg (= 0.15 mL)
*Using syringe, needle and vial of adrenaline 1 in 1000 strength

This study examines AAI prescribing habits across the UK.

**Methods**

Dispensing data was obtained from Lloyds Pharmacy

**Results**

Between November 2015 and November 2016, 30426 patients were dispensed AAI’s. Of this 30426, 7496 patients (25%) were < 12 years old and 22930 (75%) were > 12 years old.

Of those patients < 12 years old, 76.2% were prescribed 150 mcg products, 23.7% 300 mcg products and 0.1% 500 mcg products.

Of those patients aged 6–12 years old, 65% are prescribed 150 mcg products.

Of those patients > 12 year olds: 2% were prescribed 150 mcg products, 95% 300 mcg products and 3% 500 mcg products.

The majority of CCG’s in England do not appear to be demonstrating any change in dispensing behaviour since the BSACI’s guideline. Scottish Area Teams have shown the greatest change in behaviour over this period with 8 out of 11 areas switching to dispense more single item scripts for AAI. The Grampian area team now dispenses 73% of scripts with 1 item compared to 32% last year.

**Conclusion:** Recognising that some children > 12 years old will be small (< 50 kg) and not appropriate for a 0.5 mg dose of adrenaline as outlined in Table 1, this data demonstrates that only 3% of > 12 year olds had the appropriate 500 mcg product prescribed. There are therefore a large number of patients who do not have the correct dose AAI prescribed.

If the BSACI guideline is adopted there will be a large number of children of all ages who will be prescribed only 1 AAP after risk assessment. It is therefore essential to ensure that this one AAP is of the correct dose.

**References**

1. Ewan P et al. BSACI guideline: prescribing an adrenaline auto-injector. Clinical and Experimental Allergy, 2016 (46) 1258–1280
2. Muraro A et al. Anaphylaxis: guidelines from the European Academy of Allergy and Clinical Immunology. Allergy 2014; 69: 1026–1045.

## D11

### Improved standardisation of the whole blood basophil activation test to peanut

#### Matthew Kwok^1,2^*, Gideon Lack^1,2,3^, Alexandra F. Santos^1,2,3^

##### ^1^Department of Pediatric Allergy, Division of Asthma, Allergy and Lung Biology, King’s College London, London, United Kingdom; ^2^MRC and Asthma UK Centre in Allergic Mechanisms of Asthma, London, United Kingdom; ^3^Children’s Allergy Service, Guy’s and St Thomas’ Hospital, London, United Kingdom

**Correspondence:** Matthew Kwok - matthew.kwok1@gmail.com

*Clinical and Translational Allergy* 2018, **8(Suppl 2):**D11

**Introduction**

The basophil activation test (BAT) is a valuable tool to diagnose peanut allergy; however, there is currently no standardised methodology for this test. We performed the BAT within 4 h of blood collection or 24 h later, using liquid or lyophilised antibodies and following a two-step or one-step simplified procedure.

**Methods**

Whole blood from patients with suspected peanut allergy was collected in lithium heparin tubes, stored at room temperature and tested within 4 or 24 h of blood collection. Blood was stained with liquid or lyophilised antibodies using a BAT method of incubating blood with peanut extract (PE) or controls (RPMI, anti-IgE and fMLP) before staining with fluorochrome-conjugated antibodies in two steps or a one-step simplified incubation procedure. Flow cytometry was performed using FACS Canto II with FACSDiva Software. The results were analysed with FlowJo v10. Statistical analyses were performed using IBM SPSS Statistics v21.

**Results**

Within 4 h of blood collection, basophil activation detected with lyophilised antibodies was not significantly different from basophil activation detected with liquid antibodies when stimulation and staining were performed using the two-step procedure (p = 0.123 for stimulation with PE; p = 0.161 for stimulation with anti-IgE). The percentage of CD63 + basophils using the simplified one-step procedure was higher with lyophilised antibodies compared to liquid antibodies (p = 0.012 for PE, p = 0.012 for anti-IgE). Within 24 h of blood collection, a decrease in the percentage of CD63 + basophils stained with liquid antibodies and using the two-step method was observed in response to PE (p = 0.028) and anti-IgE (p = 0.008) compared to when BAT was performed within 4 h of blood collection. A decrease in basophil CD63 + expression at 24 h was also observed with lyophilised antibodies using the two-step method (p = 0.017 for PE, p = 0.025 for anti-IgE) and with lyophilised antibodies using the one-step procedure (p = 0.012 for PE, p = 0.012 for anti-IgE).

**Conclusion**

Using a two-step incubation procedure, the results of BAT with lyophilised antibodies were comparable to the results of BAT with liquid antibodies and allowed improved standardisation of the BAT methodology. Using a one-step incubation procedure, lyophilised antibodies induced enhanced basophil activation that was independent of allergen stimulation and therefore not desirable. Performing BAT on the same day of blood collection using blood stored at room temperature ensured higher level of basophil reactivity.

## D12

### Current management and use of oral immunotherapy (OIT) for peanut allergy in pediatric patients in France, Germany, Italy, Spain, Switzerland and UK (EU6)

#### Andrea Vereda^1^*, Katharina Blümchen^2^, George Du Toit^3^, Frederic de Blay^4^, Nicholas Georgitseas^5^, Marie Cassese^6^, Aditya Venugopal^6^, Ellen Zigmont^7^

##### ^1^Aimmune Therapeutics, London, United Kingdom; ^2^University Hospital Frankfurt, Frankfurt, Germany; ^3^Guy’s and St Thomas’ Hospitals, London, United Kingdom; ^4^University Hospital Strasbourg, Strasburg, France; ^5^Navigant, London, United Kingdom; ^6^Navigant, New York, New York, United States of America; ^7^Aimmune Therapeutics, San Francisco, California, United States of America

**Correspondence:** Andrea Vereda - avereda@aimmune.com

*Clinical and Translational Allergy* 2018, **8(Suppl 2)**:D12

**Introduction**

Peanut allergy is a major health burden in Europe; avoidance and acute management are the major therapeutic approaches. Experimental peanut OIT has been evaluated in several small studies in Europe, yet remains unapproved and is not recommended by EAACI. The objective is to detail current practice protocols for experimental peanut OIT for children in the EU6 and understand how practice differs between clinics.

**Methods**

We conducted qualitative, in-depth, telephonic interviews with seventy-five allergists and fifteen nurse food allergy specialists across the EU6 between September 2016 and February 2017. Eligibility criteria included managing > 100 peanut allergy patients per year and offering immunotherapy in their clinic (food or environmental).

**Results**

72/90 clinicians interviewed treat pediatric (either exclusively or both pediatric and adult) peanut allergic patients. All patients receive a full diagnostic workup; in select cases, a food-challenge is performed and moderate/severe patients receive an autoinjector, however, this varies by country. Experimental peanut OIT in children varies significantly across practices, particularly with regards to patient selection, protocols, and peanut material used.

Patient selection: Some physicians view disease severity as a primary selection criteria whereas others weigh family environment as equally important

Peanut material: Includes whole peanuts (Spain), peanut candy (France, Switzerland), pharmacy-compounded peanut flour capsules (Germany, Italy, UK), peanut administered in liquid solution (Switzerland)

Starting dose: Can be patient-tailored, e.g. in France and Switzerland, patients initiated at 10% of the reactive food challenge dose or fixed; in Germany, and Italy, dose ranges from 0.1 to 10 mg whole peanut in different practices

End-dose: 500 mg - ~ 1 gm whole peanut

Up-dosing interval: Every few days, up to 1 month

Clinician oversight during up-dosing: Ranges from no oversight (patients updose themselves at home with the assistance of a parent or caregiver) to intensive monitoring (patient observed for 3–4 h following peanut OIT updosing administration, monitored by physician or nurse)

Frequency of OIT use: Ranges from being offered throughout the country in allergy centers (France) to only being offered in clinical trial settings (Germany)

Amongst those physicians not offering peanut OIT, major barriers include lack of an EMA approved therapy for children, no standardized protocols, and the absence of a recommendation in national guidelines.

**Conclusions**

Substantial variability in the approach to experimental peanut OIT exists within and across European countries. Physicians indicate a significant unmet need for a standardized, EMA approved OIT protocol to treat peanut allergies as well as tailored protocols to treat children.

## D13

### Childhood mactocytosis and anaphylaxis after oral amoxicillin: beyond drug allergy

#### Isis Monteiro^1^*, António J. Cabral^1^, Ricardo Fernandes^1^, Joana Fermeiro^1^, Cristina Tapadinhas^2^, Anabela Lopes^3^, Ana Margarda Neves^3^

##### ^1^Pediatric Allergy Unit, Hospital de Santa Maria, Lisbon, Portugal; ^2^Dermatology Department, Hospital de Santa Maria, Lisbon, Portugal; ^3^Immunoallergology Department, Hospital de Santa Maria, Lisbon, Portugal

**Correspondence:** Isis Monteiro - isis.sm@gmail.com

*Clinical and Translational Allergy* 2018, **8(Suppl 2):**D13

**Introduction:** Mastocytosis is a rare group of disorders characterized by clonal proliferation and excessive accumulation of mast cells in tissues. Cutaneous mactocytosis (CM) is the predominant form in children, usually benign, though anaphylaxis can occur due to facilitated release of mast cell activation mediators.

The incidence of anaphylaxis in children with mastocytosis is higher than in general pediatric population. Specific triggers may be implicated (foods, hymenoptera venom), but its cause is most frequently idiopathic or unidentified.

**Case report:** Male child with diagnosis of CM at 3 years of age, manifested by cutaneous lesions on the trunk since birth with positive Darier’s sign, confirmed by skin biopsy and normal serum tryptase. Clinical course was stable under oral antihistamine. At age 7, the patient was diagnosed with bacterial tonsillitis and prescribed oral amoxicillin. Fifteen minutes after first intake, he developed generalized urticaria and angioedema of the face and tongue, requiring emergency treatment with adrenaline and antihistamine. The child was first evaluated in Pediatric Drug Allergy consult at age 8, presenting frequent nasal pruritus, dry cough and occasional wheezing and shortness of breath. The laboratory workup showed eosinophilia (1170/mm3), normal serum tryptase (5.1 μg/L), elevated total IgE (785 U/mL), negative IgE for beta-lactams (penicillin G and V, ampicillin, amoxicillin and cefaclor) and positive IgE specific for airborne allergens (*D. pteronyssinus *> 100 kU/L, *D. farinae* 59.0 kU/L, *D. glomerata* 23.0 kU/L, *A. alternata* 0.75 kU/L). Genetic study was negative for KIT D816 V mutation. Sequential in vivo tests were performed (skin prick and intradermal tests) with PPL, MDM, penicillin, amoxicillin, clavulanate, cefuroxime and ceftriaxone, with no immediate or late reaction. He later underwent a challenge test with oral cefuroxime, also negative. Currently aged 9, the child carries an adrenaline autoinjector device; he remains stable and no new anaphylactic events were reported.

**Conclusion:** Anaphylaxis in patients with mastocytosis requires a detailed, careful approach to identify the causal agent and its implication in the reaction (which is not always possible). In the presented case, the accute infection may have played a facilitating role and the investigation ruled out specific hypersensitivity to beta-lactams. It also provided a safe alternative to amoxicillin within a first-line antibacterial drug class, avoiding potential life-threatning events in the future.

**Consent to publish**

The parents of the patient have provided written consent to publish.

## D16

### Cutaneous exposure to clinically-relevant pigeon pea (*Cajanus cajan*) proteins promote TH2-dependent sensitization and IgE-mediated anaphylaxis in BALB/c mice

#### Rinkesh Kumar Gupta^1,28*^, Kriti Gupta^1^*, Premendra Dhar Dwivedi^1^

##### ^1^Food Toxicology Laboratory, Food, Drug and Chemical Toxicology Group, CSIR-Indian Institute of Toxicology Research (CSIR-IITR), Lucknow UP, India; ^2^Department of Biosciences, Integral University, Dasauli UP, India

**Correspondence:** Kriti Gupta - rinkesh.gupta9@gmail.com

*Clinical and Translational Allergy* 2018, **8(Suppl 2):**D16

**Introduction**

Epicutaneous (EC) sensitization to food allergens may occur when the skin has been lightly damaged. The study here tested whether cutaneous exposure to pigeon pea protein(s) may cause allergic sensitization. BALB/c mice were either orally gavaged or epicutaneously sensitized by repeated application of pigeon pea crude protein extract (CPE) on undamaged areas of skin without any adjuvant; afterwards, both groups were orally challenged with the pigeon pea CPE. The experimental results support the hypothesis that in addition to oral exposure, skin exposure to food allergens can promote Th2-dependent sensitization, IgE-mediated anaphylaxis and intestinal changes after oral challenge. Based on this, an avoidance of cutaneous exposures to allergens might prevent development of food anaphylaxis.

**Methods**

1. Epicutaneous and oral treatment
2. Analysis of signs and symptoms of anaphylaxis
3. Type 1 skin test
4. Measures of specific IgE and IgG1 and of MCPT-1 and TSLP
5. Histopathology of skin and intestine
6. Expression of cytokines and TFs
7. Isolation of skin and intestinal proteins and Western blotting
8. Mast cell staining

**Results**

In the epicutaneously-sensitized mice, elevated levels of specific IgE and IgG1, as well as of MCPT-1, TSLP, TH2 cytokines and TFs, higher anaphylactic scores and histological changes in the skin and intestine were indicative of sensitization ability via both routes in the pigeon pea CPE-treated hosts. Elevated levels of mast cells were observed in both the skin and intestine. Decreased levels of filaggrin in skin may have played a key role in the skin barrier dysfunction, increasing the chances of sensitization.

**Conclusions**

Little is known regarding the prevention of food allergy development via the EC exposure. The current study identified an IgE-mediated anaphylaxis following oral challenge and induction of TH2-biased adaptive immune responses when mice were exposed to pigeon pea proteins on their healthy intact skin. An additional interesting finding was that EC sensitization also yielded intestinal changes with reference to mast cells. The immune response caused by IL-4 and IL-13 contributes to the impairment in filaggrin, therefore neutralization of IL-4 and IL-13 that may improve skin barrier dysfunction. These findings support the hypothesis that cutaneous exposure to food allergens may be a risk factor for the allergic sensitization and development of food allergy.

**Acknowledgements**

The authors are grateful to the Director of the Institute for the keen interest in this study. RKG is thankful to the Indian Council of Medical Research (ICMR), New Delhi for award of his Senior Research Fellowship. KG is thankful to the Department of Science and Technology (DST), New Delhi for award of her Women Scientist. This is CSIR-IITR manuscript number 3379.

**References**

1. Sicherer SH, Leung DY. 2015. Advances in allergic skin disease, anaphylaxis, and hypersensitivity reactions to foods, drugs, and insects in 2014. J Allergy Clin Immunol. 135:357–367.
2. Sicherer SH, Sampson HA. 2010. Food allergy. J Allergy Clin Immunol. 125:S116–S125.

## D17

### The prevalence of cashew nut sensitisation and allergy in children presenting to an Irish tertiary allergy clinic

#### Susan Keogh^1,2^, Aideen Byrne^1,2^*, Cathryn O’Carroll^1,2^

##### ^1^Allergy Department, Our Lady’s Children’s Hospital, Dublin, Ireland; ^2^Dept of Pediatrics, The National Children’s Hospital Tallaght, Dublin, Ireland

**Correspondence:** Aideen Byrne - aideen.byrne@olchc.i.e.

*Clinical and Translational Allergy* 2018, **8(Suppl 2):**D17

**Introduction**

Cashew nut allergy, associated with a high risk of anaphylaxis, is increasing worldwide [1]. Food allergy prevention is a growing reality and thus it is important that the specific food allergy risks of different populations are identified. In the past 10 years, cashew consumption in Europe, including Ireland, has grown by 30%. A recent UK study revealed a differential prevalence in cashew allergy between Caucasian and Asian populations [2]. This study explored the current prevalence of cashew sensitisation in moderate to severe eczematous Irish children and any identifiable ethnical differences.

**Method**

A retrospective review was performed on a database of patients 6 months to 17 years attending the tertiary allergy clinic. Details including clinical history and results of skin prick testing (SPT) had been collected over a 8mth period between June 2015–April 2016. SPT of ≥ 3 mm was considered a positive result. SPT of ≥ 8 mm was considered “likely allergic”. Ethnicity had not originally been recorded. Ethical approval was obtained to contact families to establish ethnicity, as per Irish census criteria, and to confirm tolerance or reactions to cashew.

**Results**

The database contained 306 patients. 123(40.1%) were sensitised to cashew by SPT. 88 0f 123 had moderate to severe eczema. 65(52.8%) had a history of asthma. 96(78%) were co-sensitised ± clinically allergic to peanut. 93(75%) were co-sensitised to egg. 15% of sensitised patients were confirmed to tolerate cashew. In contrast, 16.8% of sensitised patients had had confirmed allergic reactions to cashew. There was a significant association between wheals > 8 mm and a previous reaction; Relative Risk (RR) 2.5094(95% CI: 1.0996–5.7266 p = 0.0288). The data shows that 16% of the total cohort were either sensitised SPT > 8 mm or had had an allergic reaction to cashew.

Ethnicity was confirmed in 113 of 123. White Irish: 76 Other white:15 Black Irish/Black African/Other Black:0 Chinese:5 Other Asian:17. Chinese and Other Asian account for 19.5% of the cohort. These 2 ethnicity groups together compared with White ethnicity groups collectively were more likely to have wheals > 8 mm RR1.7727 (95% CI:1.0856–2.8948 p 0.0221).

**Conclusion**

This data shows cashew sensitisation and cashew allergy as a common finding in atopic Irish children. Thus cashew allergy poses a significant health risk and prevention strategise should be considered. Furthermore, our cashew sensitised/allergic cohort appears to be over represented by Chinese/Asian children. In the 2016 Irish census only 2.1% of the population identified themselves as Chinese or Other Asian. Thus preventative strategies in this group should be a priority.

**References**

1. Van der Valk JPM, Dubois AEJ, Gerth van Wijk R, Wichers HJ, De Jong NW. Systematic review on cashew nut allergy. Allergy 2014; 69: 692–698.
2. Luyt DK, Vaughan D, Oyewole E, Stiefel G. Ethnic differences in prevalence of cashew nut, pistachio nut and almond allergy, Pediatr Allergy Immunol 2016; 27: 645–659.

## D18

### Patterns of tree nut sensitisation among those with challenge confirmed food allergy at 12 months in the HealthNuts study

#### Vicki McWilliam^1,2,3^*, Rachel Peters^1^, Mimi L. K. Tang^1,2,3^, Shyamali Dharmage^4^, Jennifer Koplin^1^, Katrina J. Allen^1,2,3,5^

##### ^1^Murdoch Children’s Research Institute, Royal Children’s Hospital, Melbourne, Australia; ^2^Department of Pediatrics, the University of Melbourne, Royal Children’s Hospital, Melbourne, Australia; ^3^Department of Allergy and Immunology, Royal Children’s Hospital, Melbourne, Australia; ^4^Allergy and Lung Health Unit Centre for Epidemiology and Biostatistics, the University of Melbourne, Melbourne, Australia; ^5^Institute of Inflammation and Repair, University of Manchester, Manchester, United Kingdom

**Correspondence:** Vicki McWilliam - vicki.mcwilliam@rch.org.au

*Clinical and Translational Allergy* 2018, **8(Suppl 2):**D18

**Introduction**

The LEAP study findings^1^ have resulted in updated infant feeding advice recommending peanut introduction before 12 months of age. However, advice regarding the introduction of other nuts is at present unclear and the usefulness of sensitisation status to guide introduction recommendations is debated. We aimed to determine the rate of tree nut sensitisation among those with challenge confirmed allergy to egg, peanut or sesame at age 1 year.

**Methods**

Within the HealthNuts study, a population-representative longitudinal cohort study of 5276 infants, skin prick testing (SPT) was performed to egg, peanut and sesame at age 1 recruitment. Those with a SPT > 1 mm had an oral food challenge (OFC) to test for food allergy. For those attending OFC clinic appointment additional SPT were performed for tree nuts (almond, cashew, and hazelnut). Tree nut sensitisation was defined as > 3 mm wheal. Tree nuts with negative SPT were instructed to be introduced to the diet and those tree nuts with positive SPT avoided.

**Results**

Of 493 12 month olds with challenge confirmed food allergy, 143 (29%, 95% CI 25.0, 33.2) were sensitised to one or more tree nuts at age 1. Those with single food allergies had the lowest rates of tree nut sensitisation with 31% (95% CI 19.1, 46.0) of single peanut allergic infants (n = 51) sensitised to one or more tree nuts and 23% (95% CI 18.8, 27.8) of those with single egg allergy (n = 364). Those with two or more food allergies (n = 104) had higher rates of tree nut sensitisation at 48% (95% CI 38.2, 58.1). Cashew was the most common tree nut sensitisation at 23%, followed by almond (13%) then hazelnut (11%).

**Conclusion**

Tree nut sensitisation is common among those with all forms of food allergy at 12 months of age. Sensitisation rates are highest for those with multiple food allergies and similar for those with peanut and egg allergy.

**Reference**

1. DuToit, G et al. Randomised trial of peanut consumption in infants at risk of peanut allergy. N Eng J Med 2015;372:803–813.

## D20

### The quality of management offered by the general pediatrician, ENT and primary care health professionals for children with allergic rhinitis including the patient and parents understanding of the correct technique for administration of topical nasal steroid spray (TSNS)

#### Tushar Banerjee*, Antima Banerjee

##### County Durham and Darlington Foundation Trust, Darlington, United Kingdom

**Correspondence:** Tushar Banerjee - bnrjt07@gmail.com

*Clinical and Translational Allergy* 2018, **8(Suppl 2):**D20

**Introduction**

The objective is to explore the quality of management of allergic rhinitis in children with moderate to severe symptoms managed by the primary care, general pediatric and ENT clinics in a District General Hospital (DGH) and to assess the user technique for TSNS administration.

**Methods**

In this study, 46 patients (5–15 years) with moderate to severe seasonal and perennial allergic rhinitis were included. The majority (n = 28) were referred from primary care, 12 patients from general pediatric and 6 cases from ENT clinic. These patientswere referred for multisystem allergy management in the specialist pediatric allergy clinic in a DGH in the North East of England. During the consultation, the user technique of TSNS administration was observed and compared to the ideal technique mentioned in the ‘Standard Operating Procedure’ (SOP) published by British Society of Pediatric Allergy and Immunology (BSACI) in 2013. Further data on clinical management was gathered from structured clinical history during the clinic consultation.

**Results**

8/46 children with allergic rhinitis were misdiagnosed as ‘recurrent viral upper respiratory tract infection’ in primary care whereas 38 children had a diagnosis and treatment of allergic rhinitis. 5/38 patients received symptomatic treatment with chlorpheniramine alone whereas 15/38 cases received regular second generation antihistamines. 20/38 children were advised to use TSNS of which 12/20 cases (all general pediatric referral) did not receive any advice on technique for the use of TSNS, whereas all 6 cases referred from general ENT clinic and 2 children referred from the primary care received verbal advice on the technique. 10/20 cases used TSNS regularly. None of the 20 children including the 8 cases who received verbal advice could manage to follow all the correct steps as mentioned in BSACI-SOP. During the demonstration, none of the children cleared nose before using TSNS, 5/20 children shook the bottle before use, and all children actively sniffed while activating the spray. All patients choose to use standing position; only 6/20 had the correct position of the head and 5/20 used the correct hand during the administration.

**Conclusion**

The standard of care is inconsistent and fragmented for the management of pediatric allergic rhinitis in both primary and secondary care. The regular basic allergy updates for primary and secondary care health professionals and the use of audiovisual training for patient education on TSNS use is strongly recommended for improving the quality of care of allergic rhinitis in children.

## D21

### Clinical relevance of the SQ HDM SLIT-tablet in adolescents with moderate-to-severe house dust mite allergic rhinitis

#### Tomokazu Matsuoka^1^, David I. Bernstein^2^, Keisuke Masuyama^1^, Hendrik Nolte^3^, Kazuhiro Okamiya ^4^, Hanne Villesen^3^, Harold S. Nelson^5^

##### ^1^University of Yamanashi, Yamanashi, Japan; ^2^University of Cincinnati, Cincinnati, Ohio, United States of America; ^3^ALK, Hørsholm, Denmark; ^4^Torii Pharmaceutical Co. Ltd, Tokyo, Japan; ^5^Department of Medicine National Jewish Health, Denver, Colorado, United States of America

**Correspondence:** Riis Bente - berdk@alk.net

*Clinical and Translational Allergy* 2018, **8(Suppl 2):**D21

**Introduction**

House dust mite (HDM) respiratory allergy is a common and burdensome disease in children and adolescents. The SQ HDM sublingual immunotherapy (SLIT) tablet has shown to be efficacious in adults and adolescents with HDM allergic rhinitis. This abstract presents findings illustrating the clinical relevance of the efficacy from pooled data from adolescents (12–17 years) included in 2 phase III trials.

**Methods**

In 2 DBPC trials conducted in North America (clinicaltrials.gov identifier NCT01700192) and Japan (JapicCTI number 121848), subjects aged 12 + years with moderate-to-severe HDM allergic rhinitis were randomised to up to 1 year of treatment. The primary endpoint was the average total combined rhinitis score (TCRS) during the last 8 weeks of treatment in the active group compared to placebo. Data from subjects aged 12–17 years were pooled.

Post-hoc analyses concerning rhinitis exacerbation days and mild days were done for placebo versus 12 SQ-HDM. A rhinitis exacerbation day was defined as a day with an allergic rhinitis symptom score of 6, or 5 with one individual symptom scored 3 (i.e. implying a symptom that is hard to tolerate and causes interference with activities of daily living and/or sleeping). A mild day was defined as a day with no individual symptom scored higher than 1 (i.e. the symptom was clearly present but caused minimal awareness and was easily tolerated) and no antihistamine use.

**Results**

In the pooled adolescent subpopulation (N = 395), the average TCRS improved 22% with 12 SQ-HDM versus placebo (absolute difference of 1.04;p = 0.002). The estimated probability of experiencing a rhinitis exacerbation was 22.6% for placebo and 9.3% with 12 SQ-HDM (OR = 0.35; 95% CI [0.14; 0.88]; p = 0.026). The estimated probability for experiencing a mild rhinitis day was 28.5% with placebo and 47.1% with 12 SQ-HDM (OR = 2.23; 95% CI [1.18; 4.24]; p = 0.014).

Extrapolated to a full year, this corresponds to 82 days with rhinitis exacerbation and 3½ months of mild days in the placebo group, compared with 34 days with rhinitis exacerbation and almost 6 months of mild days in the 12 SQ-HDM group.

**Conclusion**

Treatment with 12 SQ-HDM significantly improved the TCRS in adolescents with moderate-to-severe HDM allergic rhinitis. Furthermore, the treatment reduced the patient’s probability for having rhinitis exacerbation days and increased the probability for having mild days with no more than minimal awareness of symptoms. Taken together these findings illustrate the clinical relevance of the SQ HDM SLIT-tablet seen in adolescents in moderate-to-severe HDM allergic rhinitis.

## D23

### The association between nasal eosinophil and aeroallergen sensitization in children and adolescents in Seoul, Korea

#### Lee Hye Jin*

##### Seoul St. Mary’s Hospital, College of Medicine, The Catholic University of Korea, Seoul, Korea Republic

**Correspondence:** Lee Hye Jin - kchyejin@gmail.com

*Clinical and Translational Allergy* 2018, **8(Suppl 2):**D23

**Introduction**

Local infiltration of eosinophils is the hallmark of allergic inflammation in the nasal tissues. Nasal eosinophil examination is useful in the diagnosis of nasal allergic inflammation and allergic rhinitis. Nasal exposure to sensitized aeroallergens increases nasal eosinophil and nasal allergenic symptoms.

**Methods**

To identify the correlation between nasal eosinophil and aeroallergen sensitization in children and adolescents. We recruited patients less than 18 years of age, with a history of chronic and acute rhinitis, and those who had nasal eosinophil examinations and MAST to aeroallergens between March 2013 and February 2016 at the Allergy Clinic in Seoul St. Mary’s Hospital in Seoul, Korea. Patients’ medical records were reviewed retrospectively. We analyzed data using *T* test, Mann–Whitney test and ANOVA to determine the association between nasal eosinophil and 18 aeroallergens (i.e. Birch-Alder Mix, oak white, Bermuda grass, orchard grass, timothy grass, sweet vernal grass, rye, mugwort, short ragweed, Japanese hop, *Alternaria alternata*, *Aspergillus*, *Cladosporium*, cats, dogs, cockroaches, *Dermatophagoides farinae*, and *Dermatophagoides pteronyssinus*).

**Results**

Of the 245 patients enrolled, 156 (63.7%) were males and mean age (± standard deviation [SD]) was 7.9 years (± 3.8). In total, 175 (71.4%) patients were sensitized to at least one of the 18 aeroallergens tested. Additionally, 118 (48.2%) and 69 (28.2%) patients had at least 1 and 5% prevalence rates of nasal eosinophils, respectively. None of the patients had severe lower respiratory infection (e.g. pneumonia) or were immunocompromised. There were significant differences in the percentage of nasal eosinophils between the groups sensitized and non- sensitized to aeroallergens (*P *< 0.001). Among the 18 aeroallergens, 18 (8.3%) patients who were sensitized to *Alternaria alternata* showed the greatest mean percentage (± SD) of nasal eosinophil (27.9% [± 32.4]) and the greatest significant difference in the proportion of nasal eosinophil when compared to the non-sensitized group (7.8% [± 17.1]) (*P *= 0.001).

**Conclusion**

Nasal eosinophil was significantly associated with sensitization to aeroallergens. Data suggest that nasal eosinophil is the most common among patients sensitized to *Alternariaalternata*.

## D24

### Clinical profile, aeroallergen sensitivity and assessment of pulmonary function in pediatric chronic rhinosinusitis

#### Anamika Anamika^1^*, Arunabha Chakravarti^1^, Raj Kumar^2^

##### ^1^Department of ENT and Head and Neck Surgery, Lady Hardinge Medical College and Associated Hospitals, New Delhi, India; ^2^National Centre of Respiratory Allergy, Asthma and Immunology, Department of Respiratory Allergy and Applied Immunology, Vallabhbhai Patel Chest Institute, University of Delhi, Delhi, India

**Correspondence:** Anamika Anamika - anamikakoro@yahoo.com

*Clinical and Translational Allergy* 2018, **8(Suppl 2):**D24

**Introduction**

The role of atopy has been suggested in development of CRS as allergic rhinitis commonly co-exists in pediatric and adult patients with CRS [1]. Allergic disorders are known to affect quality of life of both parents and children with the disease [2]. And if co-exist with CRS these may add to the impairment in quality of life. There is paucity of literature on pulmonary function test and quality of life assessment in children with CRS and allergy. Theknowledge of aeroallergen sensitivities in children with CRS and allergic rhinitis is important as it will help in improving both diagnostic and treatment strategies [3].

**Methods**

This descriptive, observational, cross sectional study included 110 children, aged 7–18 years, fulfilling the requisite inclusion criteria and diagnosed with CRS at pediatric ENT clinic of a tertiary referral centre from November 2015 to March 2017. Clinical grading of symptoms was done with the help of Global Assessment of Rhinosinusitis Symptom Severity Score [4] and each patient underwent diagnostic nasal endoscopy and Lund Mackay endoscopic score [5] was calculated. These patients underwent skin prick testing [6] for 65 common aeroallergens, absolute eosinophil counts, serum total IgE level and pulmonary function test [7]. The quality of life was assessed with the help of SN-5 quality of life survey [8].

**Results**

Positive skin prick test to at least one of the common aeroallergens was present in 52.7% patients. Aeroallergen sensitivity for single aeroallergen was present in 28.2% and for multiple aeroallergens was present in 24.5%. Most common aeroallergen positivity was seen with insects (43.6%). 8.1% of children with CRS had evidence of lower airway obstruction on pulmonary function test. SPT positive patients had significantly high mean SN-5 score than SPT negative patients (P value-0.000).

**Conclusion**

There is a high prevalence of allergy in pediatric patients with CRS. There is a trend of lower airway obstruction in this group of patients. The quality of life of allergic children with CRS is poorer as compared to non allergic children with CRS.

**References**

1. Krause HF. Allergy and chronic rhinosinusitis. Otolaryngol Head Neck Surg 2003;128:14–16.
2. Juniper EF. Quality of life in adults and children with asthma and rhinitis. Allergy. 1997;52(10):971–7.
3. Sedaghat AR, Phipatanakul W, Cunningham MJ. Characterization ofaeroallergen sensitivities in children with allergic rhinitis and chronic rhinosinusitis. allergy rhinol (providence). 2014;5(3):e143-e145.
4. D.J. Kay, R.M. Rosenfeld, Quality of life for children with persistent sinonasal symptoms, Otolaryngol.—Head Neck Surg. 128 (2003) 17–26.
5. Lund VJ, Mackay IS.Staging in rhinosinusitus.Rhinology. 1993;31(4):183–4.
6. Dreborg S. The safety of skin tests and the information obtained from using different methods and concentrations of allergen. Allergy 1993;48:473–5
7. Lung function testing: selection of reference values and interpretative strategies. American Thoracic Society. Am Rev Respir Dis. 1991;144(5):1202–18.
8. Kay DJ, Rosenfeld RM. Quality of life for children with persistent sinonasal symptoms, Otolaryngol Head Neck Surg. 2003; 128:17–26.

## D25

### Genome-wide association study of asthma exacerbations in European children treated with inhaled corticosteroids

#### Susanne J. Vijverberg^1,2±^*, Natalia Hernandez-Pacheco^3±^, Niloufar Farzan^1,2^, Ben Francis^4^, Carlos Flores^3,5^, Maximilian Schieck^6,7^, Patricia Soares^8^, Leila Karimi^9^, Roger Tavendale^10^, Sommath Mukhopadhyay^8,11^, Katia M.C. Verhamme^9^, Munir Pirmohamed^12^, Colin N. Palmer,^11^, Steve Turner^13^, Daniel B Hawcutt^4,14^, Michael Kabesch^6,7^, Maria Pino-Yanes^3,5±^, Anke H. Maitland-van der Zee^1,2±^ on behalf of the PiCA and SysPharmPedia consortia

##### ^1^Department of Respiratory Medicine, Academic Medical Center (AMC). University of Amsterdam, Amsterdam, The Netherlands; ^2^Division of Pharmacoepidemiology and Clinical Pharmacology, Faculty of Science, Utrecht University, Utrecht, The Netherlands; ^3^Research Unit, Hospital Universitario N.S. de Candelaria, Universidad de La Laguna, Santa Cruz de Tenerife, Spain; ^4^Department of Women’s and Children’s Health, University of Liverpool, Liverpool, United Kingdom; ^5^CIBER de Enfermedades Respiratorias, Instituto de Salud Carlos III, Madrid, Spain; ^6^Deptartment of Pediatric Pneumology and Allergy, University Children’s Hospital Regensburg (KUNO), Regensburg, Germany; ^7^Deptartment of Pediatric Pneumology, Allergy and Neonatology, Hannover Medical School, Hannover, Germany; Member of the German Lung Research Center (DZL); ^8^Academic Department of Pediatrics, Brighton and Sussex Medical School, Royal Alexandra Children’s Hospital, Brighton, United Kingdom; ^9^Deptartment of Medical Informatics, Erasmus University Medical Center, Rotterdam, The Netherlands; ^10^Division of Molecular and Clinical Medicine, School of Medicine, University of Dundee, Dundee, United Kingdom; ^11^Population Pharmacogenetics Group, Biomedical Research Institute, Ninewells Hospital and Medical School, University of Dundee, Dundee, United Kingdom; ^12^Department of Molecular and Clinical Pharmacology, Institute of Translational Medicine, University of Liverpool, Liverpool, United Kingdom; ^13^Child Health, University of Aberdeen, Aberdeen, United Kingdom; ^14^Alder Hey Children’s Hospital, Liverpool, United Kingdom; ^±^ These authors contributed equally to this work

**Correspondence:** Susanne J. Vijverberg - s.j.vijverberg@amc.uva.nl

*Clinical and Translational Allergy* 2018, **8(Suppl 2):**D25

**Introduction**

Inhaled corticosteroids (ICS) are the most commonly prescribed medication to control persistent asthma. However, a high proportion of patients does not respond to this medication and suffer exacerbations. Genetic variation has shown to influence treatment response to ICS. In this study we aim to identify genetic variants associated with asthma exacerbations despite ICS use in European children.

**Methods**

Within the Pharmacogenomics in Childhood Asthma (PiCA) consortium we performed a Genome-Wide Association Study (GWAS) of asthma exacerbations in 3 European cohorts; PACMAN (NL), PASS (UK), and followMAGICS (GER). In total, 1204 asthmatic children treated with ICS were analysed. Imputation of genetic variants was performed using the Haplotype Reference Consortium as reference panel by means of the Michigan Imputation Server. Association testing of 7.5 million genetic variants with minor allele frequency ≥ 1% was performed using logistic regression models with EPACTS. Subsequently, results were meta-analyzed using METASOFT.

**Results**

A total of 74 genetic variants were suggestively associated with asthma exacerbations despite the use of ICS (*p *≤ 5 × 10^−6^). The most significant variants were located in 9 different loci (minimum *p*-value = 2.3x10^−7^), including one gene previously identified as associated with ICS response in Asian populations (*ALLC*). Additionally, novel associations were revealed in biologically plausible genes with drug metabolism functions and in genes belonging to the Wnt/β-catenin signaling pathway.

**Conclusion**

This is the first GWAS of ICS response in European children with asthma. We identified several novel genes suggestively associated with asthma exacerbations despite the use of ICS. These results will be validated with further independent studies.

This work was supported by Instituto de Salud Carlos III (AC15/00015) and the ERACoSysMed 1st Joint Transnational Call (SysPharmPedia, ID:99) from the European Union, under the Horizon 2020.

## D27

### Pediatrics living with severe asthma in the year 2016: results of a global survey

#### Paraskevi Katsaounou^1^*, Marcela Gavornikova^2^, Michael E. Hyland^3^, Xavier Jaumont^2^, Lorena Garcia Conde^2^, Matthias Gasser^4^, Mikaela Odemyr^5^, Ismail Kasujee^2^

##### ^1^Respiratory Medicine National and Kapodistrian, University of Athens, Athens, Greece; ^2^Novartis Pharma AG, Basel, Switzerland; ^3^School of Psychology, University of Plymouth, Plymouth, United Kingdom; ^4^GFK Switzerland AG, Basel, Switzerland; ^5^European Federation of Allergy and Airways Diseases Patients’ Associations (EFA), Brussels, Belgium

**Correspondence:** Paraskevi Katsaounou - paraskevikatsaounou@gmail.com

*Clinical and Translational Allergy* 2018, **8(Suppl 2):**D27

**Introduction**

We present findings from a global survey assessing the impact of severe asthma on the daily lives of patients, conducted to evaluate what had changed 10 years after the EFA survey ‘Still Fighting for Breath’.

**Methods**

Patients aged 6–17 years with severe persistent asthma were included (through their caregivers who completed a questionnaire on asthma control, daily/physical activity, psychological aspects and treatment). Data were collected using an online survey conducted by GfK Health on behalf of Novartis between 12 July and 31 October 2016 in seven countries.

**Results**

Caregivers (N = 152) of children and adolescents diagnosed with severe asthma were included (country-wise distribution and patient demographics shown in Table 1). Of the total population, 55% of adolescents and 67% of children were diagnosed with allergic asthma (Fig. 1).

**Table 1:** Sample distribution and patient demographics

	Adolescents*	Children*	Total
Country			
United Kingdom	18	11	29
France	15	15	30
Germany	14	10	24
Brazil	10	20	30
Canada	3	6	9
Spain	4	16	20
Italy	5	5	10
Total	69	83	152
Male/Female, %	52/48	54/46	N/A
Age, years			
Male, mean ± SD	14 ± 1.5	8 ± 1.5	N/A
Female, mean ± SD	14 ± 1.8	8 ± 1.8	N/A
Diagnosis by, %			
Pediatricians	20	31	N/A
Respiratory physicians	23	27	N/A
Allergists	23	24	N/A
General practioners	30	18	N/A
Mean disease duration, years	7	3	N/A

*Data captured through caregivers. Patients were aged between 6 and 17 years

**Fig. 1:**
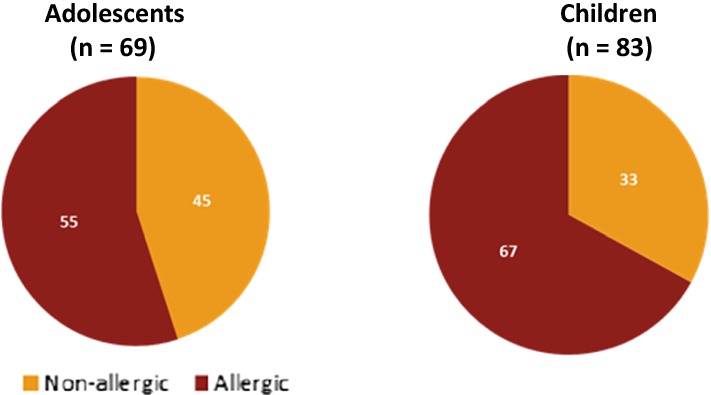
Proportion (%) of adolescents and children diagnosed with allergic or non-allergic asthma assessed as per individual patient’s knowledge

A large discrepancy was observed between patient-reported perceived asthma control (54%) and asthma control as per GINA guidelines (12%; figures for adolescents and children reported in Fig. 2).

**Fig. 2:**
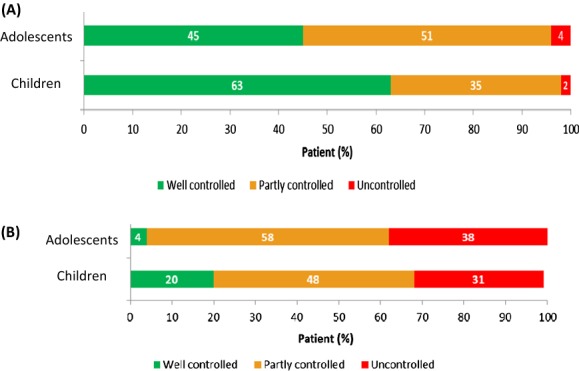
Level of control **a** as perceived by patients, **b** as per GINA control questionnaire

The majority of patients took both controller and reliever medications (Fig. 3). In the previous year, 86% of adolescents and 72% of children experienced exacerbations (most common symptoms were cough, wheezing, persistent shortness of breath, breathlessness even while lying down, fatigue) that required intervention by HCPs (50% of adolescents and 39% of children experienced ≥ 2). The majority of patients physically recovered within 24 h after treatment; however, emotional/psychological recovery took longer (17% adolescents and 25% children took ≥ 1 week). On average, they received 20 days of OCS in the last 6 months.

**Fig. 3:**
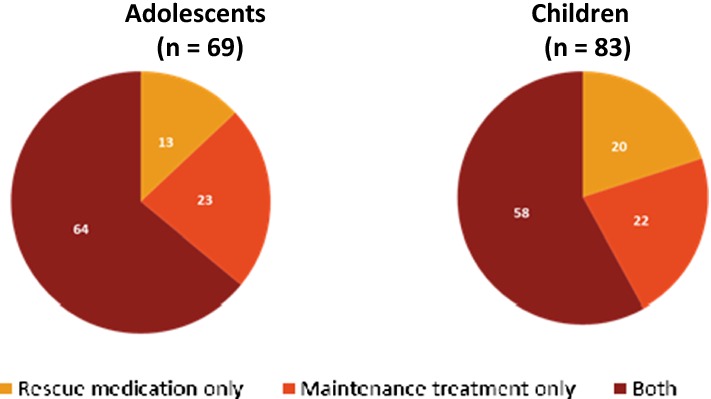
Proportion (%) of adolescents and children with severe persistent asthma on maintenance treatment, as needed reliever medication or on both

Anxiety and depression related to asthma was diagnosed in 39 and 13% of adolescents and 18 and 10% of children, respectively. Of caregivers, 36% felt their professional lives were negatively affected by their child’s disease. A large proportion of adolescents (84%) and children (67%) reported to have disruption in daily living activities with lack of exercise tolerance most often mentioned (25% adolescents and 21% children). Physical activities (90% adolescents; 89% children) and social activities (59% adolescents; 46% children) were disrupted and 90% of respondents reported disturbed sleep.

**Conclusion**

There is a strong disconnect between GINA-defined (12%) and patient-perceived asthma control (54%) in patients with severe persistent asthma. This study indicates that the majority of children and adolescent patients remain uncontrolled, have serious restrictions in their daily activities and poor psychological state. Patients with severe persistent asthma need improved disease management (support and strategies) to achieve better asthma control and live normal, unrestrained lives.

**Acknowledgements**

This study was funded by Novartis Pharma AG, Basel, Switzerland

## D29

### The impact of food allergy on asthma morbidity amongst schoolchildren

#### Idun Holmdahl*, Björn Nordlund, Anna Asanoj, Gunilla Hedlin, Jon Konradsen

##### The Department of Women's and Children's Health, Karolinska Institutet, Stockholm, Sweden

**Correspondence:** Idun Holmdahl - idun_hp@hotmail.com

*Clinical and Translational Allergy* 2018, **8(Suppl 2):**D29

**Introduction**

Food allergy and asthma often coexist in children. The aim of this study was to explore the relationship between food allergy and asthma morbidity in children with asthma of different severity.

**Methods**

The study included school-age children with severe asthma (def.: persistent symptoms and/or exacerbations despite high doses of inhaled corticosteroids (≥800 μg Budesonide/24 h)) (n = 57) and aged-matched children with controlled asthma (def.: low doses of inhaled corticosteroids (≤400 μg Budesonide/24 h) and no symptoms) (n = 39). The protocol included a questionnaire, asthma control test (ACT), exhaled NO (FENO), metacholine provocation (dose response slope, DRS), and blood sampling for IgE antibodies (Phadiatop and Fx5) and blood eosinophils.

**Results**

The mean age of the participants were 13.4 years and 58% were boys. The majority were sensitized to at least one allergen (83%) and had a family history of asthma/allergy (91%). 33% of the participants had suffered an anaphylactic reaction and 66% had IgE antibodies against ≥ 1 food allergen.

Children with severe asthma had a lower score at ACT (17.1 vs. 22.9) (p < 0.001) and more asthma symptoms during the night (49 vs. 5) (p < 0.001). There were no difference between the two groups regarding family history (p = 0.39) and anaphylaxis (p = 0.38)

The children in the study were divided into three groups based on Phadiatop and Fx5; Group 1: only sensitized to airborne allergens (n = 35), Group 2: sensitized to food and airborne allergens (n = 40) and Group 3: non-atopic (n = 21). Group 2 had a higher level of FENO (median 25 compared to group 1: 17 and group 3: 9) (p = 0.004) and increased bronchial hyperresponsiveness (DRS 18 compared to group 1: 11 and group 3: 2.5) (p = 0.023). Group 2 had a higher prevalence of anaphylaxis compared to group 1 and group 3 (p = 0.001). There was no significant difference in ACT between the three groups (p = 0.26) or eosinophils (p = 0.053).

**Conclusion**

Children with asthma who are sensitized to both airborne allergen and food have an increased bronchial hyperresponsiveness and signs of a more pronounced airway inflammation compared to children with only airway allergy. The results suggest that more intense asthma treatment is needed for these children.

## D30

### Improving medication adherence during the Diagnostic-Therapeutic–Educational Pathway IOEASMA: a real life study of 1164 children

#### Sebastiano Guarnaccia*, Valeria Gretter, Ada Pluda, Malica Frassine, Emanuele D’Agata, Sara Griffini, Cristina Quecchia

##### “Io e l’Asma” Center, University Hospital Brescia, Brescia, Italy

**Correspondence:** Sebastiano Guarnaccia - Guarnaccia.s@gmail.com

*Clinical and Translational Allergy* 2018, **8(Suppl 2):**D30

**Introduction**

GINA asthma guidelines highlight the importance of optimizing medication adherence with a view to improving clinical outcomes. Although medication adherence is the key for optimal benefit of all pharmacological treatments.

**Methods**

Consecutive children, aged from 0 to 17 years old, were enrolled following the Diagnostic Therapeutic Education Pathway IOEASMA, that including three clinical visits at 8–12 weeks intervals and 2 follow-up visits at 6-month intervals, with a primary care evaluation between visits.

After the first visit the patient and their parents received an educational course managed by a healthcare assistant. The period of the study is from 1 June 2016 to 30 March 2017.

Before each clinical visit we checked adherence with a specific questionnaire/interview.

**Results**

1164 children were divided in three ages 0–5 (256 patients), 6–11 (605 patients), 12–17 (303 patients) years old. The nationality of patients was: Italians n° 960 (82.5%), other nationalities n° 204 (17.5%).

At first visit 274 patients answered the following: they had their medication upon them in 74 patients (26.6%) and their spacer devices in 60 (21.9%), 111 (40.5%) had daily therapy, and the correct usage of medication and devices in this group was 56 patients (50.5%).

At third visit 212 patients answered as following: they had their medication upon them in 110 patients (51.9%) and their spacer devices in 93 (43.9%), 156 (73.6%) had daily therapy, and the correct usage of medication and devices in this group was 148 patients (94.9%).

At fourth–fifth visit 215 patients, with 318 records, answered as following: they had their medication upon them in 149 patients (46.9%) and their spacer devices in 120 (37.7%), 255 (80.2%) had daily therapy, and the correct usage of medication and devices in this group was 228 patients (89.4%).

After the sixth visit 448 patients, with 580 records, answered as following: they had their medication upon them in 265 patients (45.7%) and their spacer devices in 215 (37%), 470 (81%) had daily therapy, and the correct usage of medication and devices in this group was 419 patients (89.1%).

**Conclusion**

This real life study demonstrated the difference in medication adherence during the IOEASMA pathway. The data suggest how the educational intervention, after the first visit, and following the path, enhanced the adherence from 50.5% at first visit to 94.9% at third visit. We noticed that this improvement remains stable over time at 89.4–89.1% after 6 months and longer.

## D31

### Evaluating the impact of staff education on asthma first-aid knowledge and first-aid skills performance in primary schools

#### Kate Luckie^1^*, Bandana Saini^1^, Yien Yien Soo^1,2^, Vicky Kritikos^1,3^, Jack Collins^1^, Rebekah Jane Moles^1^

##### ^1^Faculty of Pharmacy, University of Sydney, Australia; ^2^National Prescribing Service, Sydney, Australia; ^3^Woolcock Institute of Medical Research, Sydney, Australia

**Correspondence:** Kate Luckie - kate.luckie@sydney.edu.au

*Clinical and Translational Allergy* 2018, **8(Suppl 2):**D31

**Introduction**

Improvements in the knowledge and skills associated with Asthma First-Aid (AFA) could reduce the morbidity and mortality associated with asthma. Research has shown that 45%of people did not receive medical assistance in their final fatal asthma attack [1]. To assess AFA training the skills required to treat an acute exacerbation of asthma should be assessed alongside AFA knowledge. Our research explored AFA education in primary school staff by comparing the effectiveness of AFA skills-based scenario training to conventional asthma education.

**Methods**

Nineteen primary schools (204 participants) were allocated to one of three arms to compare AFA knowledge and AFA skills. The scenario-based skills assessment required the participant to sit with the educator and describe how they would manage a child having a severe exacerbation of asthma using the AFA equipment provided. Conventional asthma training was a didactic oral presentation. Arm 1 underwent conventional asthma training, arm 2 underwent scenario based training and arm 3 had a combination of the two. Each participant was followed up at 3 weeks and again between 3 and 7 months after the first visit.

**Results**

AFA skills improved significantly from baseline for those study arms who received scenario based training (arms 2 and 3) and was sustained over time. There was a significant difference in AFA skills between study arm 1 (77.3%) and study arms 2 and 3 (91.5 and 91.1%) (p = 0.000). After study arm 1 received the scenario-based training the mean skill score increased to 90% at visit 3 which was comparable to the other two study arms. AFA knowledge improved significantly in all study arms with no differences seen between arms.

**Conclusion**

Scenario-based training was superior to conventional training for AFA skills. There was no significant difference in AFA knowledge with scenario-based training versus the conventional asthma training.

**Reference**

1. Levy ML, Winter R. Asthma deaths: what now? Thorax. 2015;70(3):209–10.

## D32

### Knowledge and attitude towards pediatric asthma of health care practitioners (KAAP)

#### Roy Subhasis^1^*, Halder Indranil^2^, Ghosh Subhajit^3^

##### ^1^Consultant pediatrician, Columbia Asia Hospital Kolkata, India; ^2^Consultant and Associate Professor, JNU university, Kalyani, West Bengal, India; ^3^Cipla respiratory, Mumbai, India

**Correspondence:** Roy Subhasis - roysubha2006@gmail.com

*Clinical and Translational Allergy* 2018, **8(Suppl 2):**D32

**Introduction**

Childhood asthma is a major public health problem. There has been limited research into physician perception and attitude towards pediatric asthma in India.

The objective is to assess the knowledge, attitude and behaviour of three groups of health care practitioners, i.e. Pediatric pulmonologist, pediatrician and general practitioner towards pediatric asthma.

**Methods**

This is an observational cross-sectional survey of knowledge, attitude and behavior of health care practitioners through online self-administered questionnaire. Total 719 responses obtained from across India.

**Results**

Participants were distributed as follows General Physician 1%, General Pediatricians 82%, and Pediatric Pulmonologists 17%. Most responders (54%) stated that prevalence pre-school wheezing is 16–30%. Suspected/confirmed asthma in general practice is < 15% in pediatric age group (56%). According to 58% responders October to December has highest number of wheezy children. Bronchodilators prescribed in cough and cold (24%), in rhonchi (32%) and recurrent cough (38%). Recurrent cough (70%) followed by recurrent wheeze (23%) is the most common presenting symptoms in pediatric asthma. Asthma is mostly diagnosed clinically (626). 51% doctors stated that GINA guidelines is not that useful. Only 17% responders mentioned maternal smoking avoidance for primary prevention of childhood asthma with breast feeding (71%) is most common response. Around 19% participant reported growth retardation with inhaled corticosteroids. While initiating inhaler therapy more than 40% doctors spend < 10 min to counsel. Around 12% responders also used Peak Flow Meter in below 5 years. Around 20% of Device and inhalation technique demonstration done by unskilled personnel. For routine inhalation 9% still preferred nebulizer in below 5 years and Over 5 years almost 10% of doctors prefer nebulizer and MDI without spacer. In 5 years and above MDI with spacer (74%) is preferred route to deliver bronchodilator but almost 10% also used non-inhalational routes. Almost one-fourth (24%) prefer nebulizer to treat wheezy child under 6 years. In 5 years and above MDI with spacer (54%) followed by MDI with spacer and mask (36%) is preferred choice for inhaled steroid delivery. Levosalbutamol (59%) is preferred over salbutamol (40%). Almost 21% responders are unaware of dose in one salbutamol actuation. Budesonide (93%) is most preferred molecule. In one-third of patient’s moderate dose of budesonide equivalent prescribed. Around 17% prescriber still advise patients to buy nebulizer. Around 93% preferred valve spacer with rest either non-valve or no preference.

**Conclusion**

This countrywide survey showed there are gaps in knowledge, discrepancy in practice and attitude towards pediatric asthma.

## D33

### Evaluating acute asthma management at Red Cross Children’s Hospital, South Africa

#### Naidoo Shirani^1^*, Buys Heloise^1^, Kang Kristopher^2^

##### ^1^University of Cape Town, Cape Town, South Africa; ^2^University of British Columbia, Vancouver, Canada

**Correspondence:** Naidoo Shirani - shirani.naidoo@uct.ac.za

*Clinical and Translational Allergy* 2018, **8(Suppl 2):**D33

**Introduction**

SA has traditionally had high rates of mortality and morbidity related to Acute Severe Asthma. With improving access to care, we have re-evaluated the presentation and management of children with Acute Severe Asthma at our facility.

**Methods**

A retrospective chart review of patients with asthma < 13 years, with physician diagnosed asthma, triaged “Red “(i.e. requiring immediate medical attention), presenting to the Medical Emergency Unit between 01/01/2013 to 31/12/2013. Patients with any chronic underlying lung disease were excluded.

**Results**

95 patients were seen in 2013 (48 male, 47 female), who were predominantly 2–8 years of age, with an increased admission rate from April to October, and a large month to month variation. The highest frequency of presentations was from 0600 to 1200 hours, and the majority of patients presented directly from home, bypassing primary care facilities. Bronchodilators were started in 94 of 95 patients (1 exclusion due to inadequate notes). Fenoterol was the first line agent, with Ipratropium bromide used in 90 patients. The most common method of administration was continuous fenoterol and iprotropium nebulisations. (71.7% of patients). 44% of patients completed 3 SAB2nebulisations optimally (within first hour of treatment) and a further 21%received 3 nebulisations outside this time frame. 83% of patients received systemic steroids immediately, the majority via the oral route. There was high usage of antibiotics (40%) and chest XRays (82%), despite a 2% yield of an unexpected result on CXR. Additional treatment modalities were commonly required, such as IV Magnesium Sulphate (24%) and IV Salbutomol (21%), with no adverse effects noted. Respiratory support was predominantly non-invasive (2 patients commenced on HiFlo Oxygen, 10 commenced on CPAP and 1 patient requiring emergency intubation and ventilation). None of the children commenced on non-invasive support required escalation to IPPV during their illness.

**Conclusion**

The majority of patients presented during the day and bypassed primary care. They generally received continuous nebulisation with 3 doses of aSAB2 agent and Ipratropium (although the timing of doses can be expedited). There was 0% mortality, with only onepatient requiringintubation and ventilation. We have used modalities such as IVMagnesium sulphate, salbutomoland non- invasive respiratory support safely and effectively in the Emergency Unit in a resource constrained setting.

## D34

### Asthma management policies in Australian primary schools: are we doing enough?

#### Kate Luckie^1^*, Rebekah Jane Moles^1^, Vicky Kritikos^1,2^, Bandana Saini^1^

##### ^1^Faculty of Pharmacy, the University of Sydney, Sydney, Australia; ^2^Woolcock Institute of Medical Research, Sydney, Australia

**Correspondence:** Kate Luckie - kate.luckie@sydney.edu.au

*Clinical and Translational Allergy* 2018, **8(Suppl 2):**D34

**Introduction**

At least 2 out of every 25 students in Australian classrooms have a diagnosis of asthma, hence it’s vital that schools have policies and procedures to deal with asthma management issues. The aim of our research was to explore how national and state (Macro) policies were interpreted and implemented within primary (elementary) schools.

**Methods**

Semi- structured interviews were conducted with 12 primary (elementary) school principals. Interviews were audio recorded and transcribed verbatim, subsequently undergoing thematic content analysis.

**Results**

Recurrent themes were found to fall under three headings: Asthma training, communication about asthma management and access to reliever medicines within the school. Mandatory asthma training was insufficient to equip staff to deal with asthma emergencies and there was a lack of direction regarding communication processes across all schools. The size of the school tended to dictate the process. Access to individual rescue inhalers was more restricted in the larger schools. In5 of the 12 schools emergency rescue medication was kept with the teacher on duty.

**Conclusion**

Macro policies tended to offer too much flexibility. There could be potential for improved health outcomes with macro policies facilitating greater involvement of the health care sector, the school staff and the parent.

## D35

### Nasal obstruction is significantly improved after treatment with n/s Mometasone furoate in children with allergic rhinitis as indicated by Acoustic Rhinometry (AcR) and Visual Analog Scale (VAS) measurements

#### Marialena Kyriakakou^1^, Olympia Tsilochristou^1,2^*, Maria Dimou^1^, Vicky Xepapadaki^1^, Nikos Papadopoulos^1,3^, Nikos Douladiris^1^

##### ^1^Children’s Allergy Department, 2nd Pediatric Clinic, National and Kapodistrian University of Athens, Athens, Greece; ^2^Division of Allergy, Asthma and Lung Biology, King’s College London, London, United Kingdom; ^3^Division of Infection, Immunity and Respiratory Medicine, The University of Manchester, Manchester, United Kingdom

**Correspondence:** Olympia Tsilochristou - ol.tsilochristou@gmail.com

*Clinical and Translational Allergy* 2018, **8(Suppl 2):**D35

**Introduction**

Nasal obstruction is common in children with allergic rhinitis (AR). Such obstruction may be evaluated subjectively by a visual analog scale (VAS) or objectively by measuring the nasal cavity area by Acoustic Rhinometry (AcR) and evaluating the minimum cross-sectional area (MCA). Intranasal corticosteroids (INSs) such as mometasone are recommended as first line therapy for allergic rhinitis especially when nasal blockage is the major concern, however, data in children are few. The aim of this study was to explore the efficacy of mometasone treatment by using the VAS and AcR, in children with allergic rhinitis.

**Methods**

The study population was recruited among children complaining for nasal blockage while attending the Rhinitis Outpatient Clinic of the Allergy Unit, “P. and A. Kyriakou” Children’s Hospital, Athens, Greece during 2016–2017. Children completed a VAS and underwent AcR at a first visit (Day 1) and after treatment with mometasone, 1 puff/nostril, once daily for 15 days (Day 16). AcR was performed with the A1 Acoustic Rhinometer, (GM INSTRUMENTS LTD, Kilwinning, UK) and the minimum cross-sectional area (MCA) was evaluated. Statistical analysis was done with SPSS v.23.0. Results are expressed as the mean and standard deviation, with p < 0.05 set as significant.

**Results**

Fifty-six children (22 boys/16 controls-no treatment), aged 6–9 years were evaluated. No change in both VAS and MCA Day 1 vs. VAS and MCA Day 16; respectively, was observed in children who did not comply to the mometasone treatment (control group). In contrast, those receiving the medication achieved reduction of nasal obstruction as indicated by both VAS and AcR, compared to both baseline values and the control group (no treatment). VAS Day 1 vs. VAS Day 16 was significantly higher (VAS: 7.9235 cm ± 0.923 vs. 0.930 cm ± 1.033 respectively; p < 0.001) (Fig. 1). Additionally, MCA was significantly improved in both nostrils after treatment. (MCA right nostril Day1 vs. day 16: 0.65 cm^3 ±^ 0.74 vs. 1.01 cm^3^ ± 0.71 respectively; p < 0.001 and MCA left nostril Day1 vs. Day 16: 0.60 cm^3 ±^ 0.38 vs. 1.16 cm^3 ±^ 0.45 respectively; p < 0.001) (Fig. 2).

**Fig. 1:**
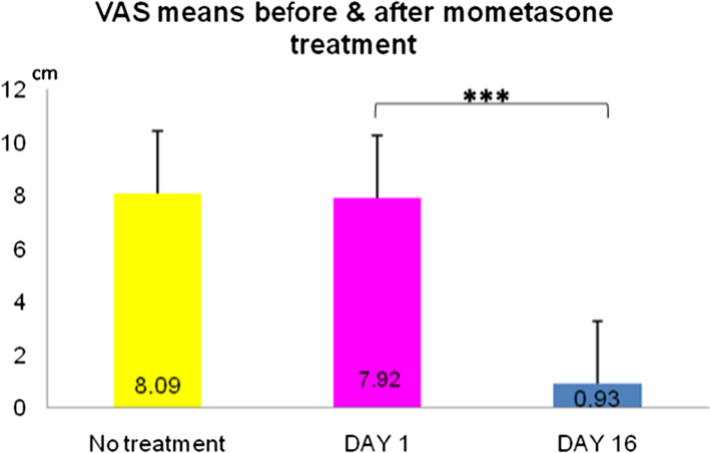
VAS means before and after mometasone treatment

**Fig. 2:**
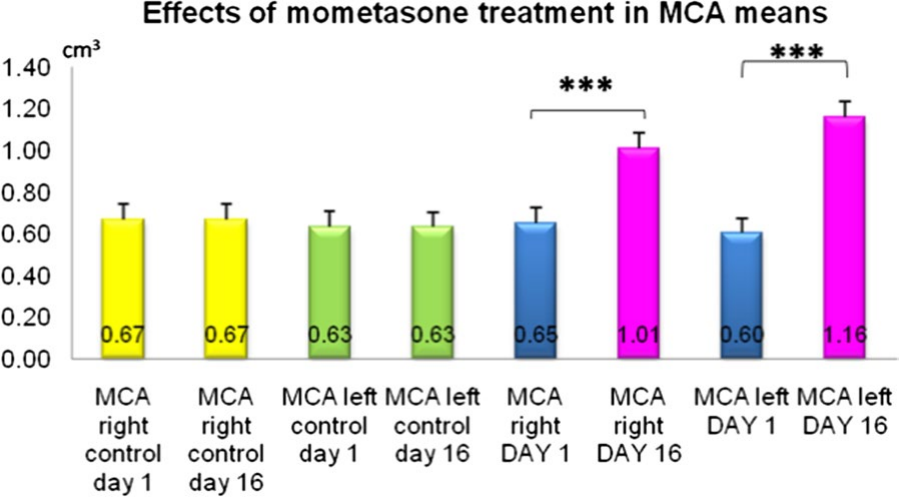
Effects of mometasone treatment in MCA means

**Conclusion**

The effectiveness of mometasone treatment for nasal obstruction in children with allergic rhinitis was confirmed by both AcR and VAS measurements.

## D36

### The investigation of the role of IL-21 and IL-33 in the pathogenesis of allergic rhinitis

#### Neriman Aydin^1^*, Işıl Bakis^2^, Ceren Gunel^3^, Buket Demirci^4^, Mete Eyigor^5^

##### ^1^Adnan Menderes University Medical Faculty Department of Medical Microbiology, Aydın, Turkey; ^2^Adnan Menderes University Institute of Health Sciences, Aydın, Turkey; ^3^Adnan Menderes University Medical Faculty Department of Ear Nose andThroat, Aydın, Turkey; ^4^Adnan Menderes University Medical Faculty Department of Medical Pharmacology Aydın, Turkey; ^5^Akdeniz University Medical Faculty Department of Medical Microbiology, Antalya, Turkey

**Correspondence:** Neriman Aydin - nkaydin@adu.edu.tr

*Clinical and Translational Allergy* 2018, **8(Suppl 2):**D36

**Introduction**

Allergic rhinitis is an inflammatory disease of nasal mucous membrane by immunoglobuin E (IgE)-mediated (allergic) reaction to aeroallergens.1 It was found that several cytokines are involved in the pathogenesis of allergic rhinitis 2, 3. In this study, it was aimed to investigate the role of IL-21 and IL-33 in allergic rhinitis by using a rat model.

**Methods**

A total of 21 rats were included in this study in three groups: (1) rats with allergic rhinitis, (2) rats with allergic rhinitis and corticosteroid (3) the control group. 4 Rats were anesthetized with xylazine-ketamine anesthesia and samples were taken. The levels of IgE, OVA sIgE, IL-21 and IL-33 were investigated in serum samples, and IL-21 and IL-33 levels were investigated in tissue samples by Enzyme Immune Assay (EIA) method.

**Results**

The IL-33 levels in tissue were found to be statistically higher in both allergic rhinitis (p = 0048) and corticosteroid + allergic rhinitis groups (p = 0.035) compared to the control group. Despite the lack of statistically significant difference in IL-21 tissue levels between the groups, the tissue levels in both allergic rhinitis and corticosteroids + allergic rhinitis groups were found to be higher than the control group. IgE levels in serum in the control group was found significantly higher than both the levels in allergic rhinitis group (p = 0.009) and corticosteroid + allergic rhinitis group (p = 0.011). Contrary to the serum IgE levels, OVA sIgE serum level was found to be the lowest in control group, however the difference was not statistically significant.

**Conclusion**

It is concluded that IL-33 and IL-21 have a role in the pathogenesis of allergic rhinitis; they are synthesized in higher levels in tissues with allergic rhinitis. Additionally, it is suggested that IL-21 has a role in downwards regulation of serum IgE levels.

**References**

1. Eifan AO, Durham SR. Pathogenesis of rhinitis. Clin Exp Allergy. 2016;46(9):1139–51.
2. Glück J, Rymarczyk B, Rogala B. Serum IL-33 but not ST2 level is elevated in intermittent allergic rhinitis and is a marker of the disease severity. Inflammation Research 2012, 5, 547–50.
3. Wang M1, Zhang W, Shang J, Yang J, Zhang L, Bachert C. Immunomodulatory effects of IL-23 and IL-17 in a mouse model of allergic rhinitis. Clin Exp Allergy. 2013;43(8):956–66.
4. Haenuki Y, Matsushita K, Futatsugi-Yumikura S, Ishii KJ, Tatsukata K, Yoshimasa I, Shigeharu F, Yasuda M, et al. A critical role of IL-33 in experimental allergic rhinitis. J Allergy Clin Immunol 2012, 130, 184–94

## D37

### Angiogenic factors in exhaled breath condensate—possible markers of disease progression in children with asthma

#### Katarzyna Grzela^1^*, Malgorzata Litwiniuk^2^, Alicja Krejner^2^, Wioletta Zagorska^1^, Tomasz Grzela^2^

##### ^1^Department of Pediatrics, Pneumonology and Allergology, Medical University of Warsaw, Warsaw, Poland; ^2^Department of Histology and Embryology, Medical University of Warsaw, Warsaw, Poland

**Correspondence:** Katarzyna Grzela - katarzyna.grzela@gmail.com

*Clinical and Translational Allergy* 2018, **8(Suppl 2):**D37

**Introduction**

Asthma is common chronic inflammatory disease of respiratory tract. Exacerbations of clinical symptoms and increased disease severity are associated with structural and functional changes in respiratory tract known as remodeling. Apart from hyperplasia of smooth myocytes and mucosal cells, as well as extracellular matrix enhancement in sub-epithelial mucosa, these changes include also some neo-vascularization. However, detailed role of angiogenesis in asthma-associated remodeling remains unclear.

Diagnostics and monitoring of asthma, especially in its early stages, are still difficult. Functional tests, e.g. spirometry, are often impossible to perform in some patients, particularly in young children. For this reason, there is strong need for new diagnostic approaches, of low invasiveness and not requiring patient cooperation. One of such methods is analysis of exhaled breath condensate (EBC).

**Methods**

In our study we analyzed 23 samples of breath condensates: 6 from severe asthma children, 8from mild asthma and 9 control samples from healthy children. For assessment we have used proteome profiler, the protein microarray dedicated to detect 55 angiogenesis-related factors.

**Results**

We have found that tested EBC samples contained several angiogenesis-related factors, including thrombospondin (TSP)-1, angiogenin (ANG), vascular endothelial growth factor (VEGF), angiopoetin-2 (Ang)-2, matrix metalloproteinase (MMP)-9 and tissue inhibitor of matrix metalloproteinases (TIMP)-1. All EBC samples from mild asthma and control groups revealed moderate signals corresponding to TSP-1. Small quantities of Ang-2 were found in EBC samples from mild asthma patients. In EBC samples from severe asthma we have found high levels of TSP-1, ANG, TIMP-1 and MMP-9.

**Conclusions**

We have shown for the first time, that exhaled breath condensates of asthma children contained broad spectrum of angiogenesis-related factors. Thus, our results support previous suggestions, that asthma progression is accompanied by significant changes in molecular network, which may be involved in angiogenesis control in respiratory tract. Moreover, we have demonstrated that those factors may effectively be studied in EBC using novel protein microarray.

## D38

### Pharmacokinetics of tiotropium in patients aged 6–11 years with moderate asthma following administration via the Respimat^®^ inhaler

#### Ashish Sharma^1^, Sabrina Wiebe^1^, Stanley Szefler^2^, René Aalbers^3^, Eckard Hamelmann^4^, Stanley Goldstein^5^, Michael Engel^6^, Petra Moroni-Zentgraf^7^, Christian Vogelberg^8^

##### ^1^Boehringer Ingelheim Pharma GmbH and Co. KG, Biberach, Germany; ^2^Children’s Hospital Colorado; University of Colorado Denver School of Medicine, Denver, Colorado, United States of America; ^3^Martini Hospital, Van Swietenplein 1, Groningen, Netherlands; ^4^Evangelisches Krankenhaus Bielefeld GmbH, Malvenstrasse 12, Bielefeld, Germany; ^5^Goldstein Stanley Asthma Care of Long Island, Rockville Centre, New York, New York, United States of America; ^6^Boehringer Ingelheim Pharma GmbH and Co. KG, Ingelheim, Germany; ^7^Boehringer Ingelheim Pty Ltd, Sydney, Australia; ^8^University Hospital Carl Gustav Carus, Technical University of Dresden, Dresden, Germany

**Correspondence:** Eckhardt Michael - michael.eckhardt@meditechmedia.com

*Clinical and Translational Allergy* 2018, **8(Suppl 2):**D38

**Introduction**

Tiotropium Respimat^®^ has been demonstrated to be efficacious and well tolerated as add-on to maintenance inhaled corticosteroids (ICS) ± additional controller therapy in children aged 6–11 years with moderate and severe asthma. The pharmacokinetic (PK) properties of tiotropium have been reported in adult and adolescent patients, but the PK of tiotropium in children with moderate persistent asthma requires elucidation. We studied single- and multiple-dose PK characteristics of tiotropium in patients aged 6–11 years with moderate persistent asthma (NCT01383499).

**Methods**

PK parameters of tiotropium were evaluated in plasma and urine samples using a subset of patients in a Phase II, randomized, double-blind, placebo-controlled, incomplete crossover trial of tiotropium; additionally, some patients who were not part of the subset consented to the 24-hour urine collection resulting in more urine data than plasma data. Overall, 24 patients were included who received tiotropium at 2.5 μg, or 5 μg as two puffs delivered by the Respimat^®^ inhaler once daily in the evening, added on to at least ICS (leukotriene modifiers were permitted throughout the trial).PK were determined after the first dose (first treatment only) and after 4 weeks of dosing.

**Results**

Tiotropium was rapidly absorbed following oral inhalation with a median t_max,ss_ following multiple dosing over 4 weeks ranging between 4.1 and 4.7 min for the two dose groups. An average of 3.17–4.32% of the nominal dose was excreted unchanged in the urine over 24 h post-single dose. At steady state, urinary excretion was 1.7 to 3.2-fold higher than post-single dose, and renal clearance was 278–358 mL/minute. Tiotropium exposure increased in an approximately dose proportional manner. PK parameters after multiple dosing are presented in Table 1.

**Table 1:** PK parameters after multiple dosing of tiotropium delivered by the Respimat^®^ inhaler in children aged 6–11 years with moderate asthma

Parameter	Unit	Tiotropium Respimat 2.5 µg			Tiotropium Respimat 5 µg		
		N	gMean	gCV [%]	N	gMean	gCV [%]
AUC_0–1,ss_	pg h/mL	3	2.08	31.7	5	3.04	38.3
C_max,ss_	pg/mL	6	2.42	48.7	5	4.10	97.2
C_pre,ss_	pg/mL				3	1.82	13.4
t_max,ss_*	min	6	4.1*	3.2–5.0*	5	4.7*	3.5–5.8*
CL_R0–3,ss_	mL/min	3	358	7.06	4	278	17.9
R_A_, Ae_0–24_		11	3.43	115	12	1.71	126
fe_0–3,ss_	%	11	2.88	48.3	12	2.02	65.2
fe_0–24,ss_	%	11	10.3	62.7	12	7.39	86.3

*Median, minimum–maximum. Ae_0–24_ = amount of tiotropium that was eliminated unchanged in urine from 0 to 24 h; AUC_0–1,ss_ = area under the curve from 0 to 1 h at steady state; C_max,ss_ = maximum measured concentration of the analyte in plasma at steady state; C_pre,ss_ = pre-dose steady state concentration of the analyte in plasma immediately before administration of the next drug administration; CL_R,0–3,ss_ = renal clearance of tiotropium in plasma from 0 to 3 h at steady state; fe_0–3 ss_ = fraction of tiotropium dose excreted in urine from 0 to 3 h at steady state; fe_0–24,ss_ = fraction of tiotropium dose excreted in urine from 0 to 24 h post-dose at steady state; gCV = geometric coefficient of variation; gMean = geometric mean; R_A_,_0–24_ = accumulation ratio from 0 to 24 h; t_max,ss_ = time from dosing to maximum tiotropium plasma concentration at steady state

**Conclusion**

These data establish the PK of tiotropium Respimat^®^ following administration of a single dose and at steady state in children aged 6–11 years with moderate asthma. Overall, the pattern of absorption, exposure and clearance at steady state was comparable in this age group to that previously published for adolescents and adults.

## D39

### *In vitro* and clinical characterisation of the antistatic valved holding chamber AeroChamber Plus^®^ Flow-Vu^®^ for administrating tiotropium Respimat^®^ in 1–5-year-old children with persistent asthmatic symptoms

#### Herbert Wachtel^1^, Mark Nagel^2^, Michael Engel^1^, Georges El Azzi^1^, Ashish Sharma^3^, Jason Suggett^2^

##### ^1^Boehringer Ingelheim Pharma GmbH and Co. KG, Ingelheim, Germany; ^2^Trudell Medical International, London, Ontario, Canada; ^3^Boehringer Ingelheim Pharma GmbH and Co. KG, Biberach, Germany

**Correspondence:** Eckhardt Michael - michael.eckhardt@meditechmedia.com

*Clinical and Translational Allergy* 2018, **8(Suppl 2):**D39

**Introduction**

When characterising any inhalation product, a comprehensive assessment including in vitro, pharmacokinetic (PK), as well as clinical results is required. We therefore assessed tiotropium Respimat^®^ when administered with the AeroChamber Plus^®^ Flow-Vu^®^ antistatic valved holding chamber (test VHC) using in vitro and PK data, as well as clinical results in 1–5-year-olds with persistent asthmatic symptoms.

**Methods**

We evaluated tiotropium delivered into a cascade impactor under fixed pediatric flow rates with and without holding times in the test VHC. Tidal breathing simulations and an ADAM-III Child Model were employed to assess the tiotropium mass likely to reach the lungs of preschool children when Respimat^®^ was administered with the test VHC. Clinical characterisation was based on a 12-week, double-blind, randomised, parallel-group trial of once-daily tiotropium Respimat^®^ or placebo as add-on to background therapy in 1–5-year-olds with persistent asthmatic symptoms (NCT01634113). PK data on systemic exposure to tiotropium Respimat^®^ administered with test VHC in preschool children was compared with pooled data from nine trials in older patients with symptomatic persistent asthma not using VHCs (NCT01383499/NCT01122680/NCT01233284/NCT01152450/NCT01696071/NCT00772538/NCT00776984/NCT01172808/NCT01172821).

**Results**

*In vitro* emitted mass decreased with lower flow conditions, indicating age-dependent dose reduction. In terms of dose per kg/body weight, delivered dosing at flow rates corresponding to preschool children was comparable to that at flow rates corresponding to older children (Table 1).

**Table 1:** In vitro medication delivery through the test VHC with small/medium face masks at different flow rates and holding times

Flow rate and corresponding age	Mask	Holding time, s	Mean medication delivery through test VHC, μg/dose	Body weight 50th percentile, kg	Medication delivered per dose, ng/kg^*^
4.9 L/min (6–12 months)	Small	0	0.85 (± 0.04)	7.5–9.9	86–113
		2	0.86 (± 0.14)		87–115
		5	0.55 (± 0.16)		56–73
		10	0.62 (± 0.02)		63–83
8.0 L/min (2–5 years)	Medium	0	0.74 (± 0.05)	12.3–18.0	41–60
		2	0.93 (± 0.05)		52–76
		5	0.72 (± 0.07)		40–59
		10	0.57 (± 0.05)		32–46
12.0 L/min (> 5 years)	Medium	0	1.16 (± 0.07)	18.0	64
		2	0.96 (± 0)		53
		5	0.78 (± 0.18)		43
		10	0.61 (± 0.02)		34

Data corresponding to age group 13–23 months are not available

*Inhalation of 2.5 µg tiotropium Respimat^®^ (as two actuations) in a 70 kg adult without use of the test VHC and face mask delivers approximately 2.5 μg or 36 ng/kg

Transmission and holding properties of tiotropium Respimat^®^ administered with the test VHC were fully sufficient for aerosol delivery of patients. Standardised tidal inhalation resulted in emitted mass from the test VHC of approximately one-third of labelled dose, independent of coordination and use of face mask, indicating predictable tiotropium administration by Respimat^®^ when used with the test VHC. Data generated from the anatomically correct ADAM-III model correlated well with standardised tidal breathing results, both in terms of total mass delivered and the mass delivered to filter (available to the lungs). In separate clinical trials, tiotropium exposure in 1–5-year-old patients using the test VHC, adjusted by height or body surface, was comparable with that observed in older patients who did not use VHCs; no overexposure was observed. The safety profile of tiotropium Respimat^®^ in 1–5-year-old patients was comparable to placebo.

**Conclusion**

This study supports administration of tiotropium Respimat^®^ with the AeroChamber Plus^®^ Flow-Vu^®^ test VHC in 1–5-year-old children with persistent asthmatic symptoms.

## D40

### Potential immunomodulation effect of *nigella sativa* on peripheral blood eosinophil count, serum IgE level and improvement of clinical manifestation in asthmatic children

#### Wisnu Barlianto^1,2^*, Muhammad Irawan^1,2^, Desy Wulandari^1,2^

##### ^1^Allergy-Immunology Division, Pediatric Department, Faculty of Medicine, Brawijaya University, Malang, Indonesia; ^2^Saiful Anwar General Hospital, Malang, Indonesia

**Correspondence:** Wisnu Barlianto - wisnu_barlian@yahoo.com

*Clinical and Translational Allergy* 2018, **8(Suppl 2):**D40

**Introduction**

One of the important goal of asthma treatment is to control the disease. Poor compliance with conventional asthma medications remains a major problem in achieving asthma control. It has been reported that black seed or *Nigella sativa* provides anti-inflammatory and anti-allergy activities. This study was aimed to investigate the effect of *Nigella sativa* as adjunctive therapy on peripheral blood eosinophil count, serum IgE level and clinical manifestation in asthmatic children.

**Methods**

A randomized, single blind-controlled trial involving twenty-eight children with asthma was done in Saiful Anwar General Hospital, Indonesia. All patients were given therapy according to standard treatment guidelines of asthma. *Nigella sativa* oil (NSO) were given as supplementary treatment at dose of 15–30 mg/kg/day for 8 week in treatment groups. Peripheral blood eosinophil count and serum IgE level were measured before and after treatment. Improvement of clinical manifestation was accessed by Asthma Control Test (ACT) Score.

**Results**

After 8 weeks of treatment, NSO group showed a significant reduction in blood eosinophils (6.89 ± 2.916% vs. 4.89 ± 1.546%, p = 0.038) and serum IgE level (703.88 ± 390.438 IU/ml vs. 385.98 ± 214.479 IU/ml, p = 0.034) compared to control group. There was no different of mean ACT score in both groups (20.29 ± 1.816 vs. 19.36 ± 1.151, p = 0.413). But there was significant improvement of ACT score between pre and post treatment in NSO group (16.57 ± 2.533 vs. 20.29 ± 1.816, p = 0.000). ACT score was associated with peripheral blood eosinophil count (p = 0.049, r = − 0.375) and serum IgE level (p = 0.001, r = − 0.587) in NSO group.

**Conclusion**

This study demonstrated that supplementation of *Nigella sativa* improves biochemical parameters and clinical manifestation in children with asthma.

## D43

### Neonatal antibiotic treatment increases the risk of asthma at age 12 years

#### Emma Goksör*, Frida Strömberg-Celind, Bernt Alm, Per Möllborg, Nils Åberg, Göran Wennergren

##### Department of Pediatrics, Institution of Clinical Sciences, University of Gothenburg, Gothenburg, Sweden

**Correspondence:** Emma Goksör - emma.goksor@vgregion.se

*Clinical and Translational Allergy* 2018, **8(Suppl 2):**D43

**Introduction**

Asthma is one of the most common chronic diseases in children. Disturbed microbiotica can affect the development of the immune system and thus the risk of asthma and allergy. Aims and objectives: To study the prevalence of and risk factors for asthma at 12 years and examine whether there were different risk factors for atopic compared to non-atopic asthma.

**Methods**

Data were obtained from a longitudinal cohort study of children born in 2003 in Sweden. The parents answered questionnaires from the age of 6 months until 12 years. We also obtained data from the Swedish Medical Birth Register. The response rate at age 12 years was 76% of those to whom the questionnaire was distributed (3637/4777), 64% of the 5654 included at admission in 2003.

**Results**

At 12 years of age, 6.4% reported current doctor-diagnosed asthma. Of these, 65% had atopic asthma and 35% non-atopic asthma. Risk factors are presented in Table 1.

**Table 1:** Adjusted odds ratios to be diagnosed with any asthma, atopic asthma, and non-atopic asthma at 12 years age

Risk factor	Any asthma aOR (95% CI) (n = 3622)	Atopic asthma aOR (95% CI) (n = 3540)	Non-atopic asthma aOR (95% CI) (n = 3471)
Neonatal antibiotics	1.9 (1.1–3.2)	2.2 (1.2–4.2)	1.4 (0.5–3.4)
Small for gastational age	2.6 (1.1–5.9)	2.3 (0.9–6.3)	3.8 (1.1–13.7)
Breastfeeding	1.2 (0.8–1.7)	1.1 (0.7–1.9)	0.5 (0.3–0.95)
Parental asthma	2.6 (1.9–3.7)	2.5 (1.7–3.6)	3.0 (1.7–5.0)
Food allergy first year of life	2.2 (1.3–3.7)	3.0 (1.8–5.1)	0.6 (0.1–2.5)

**Conclusions**

Neonatal antibiotic treatment increased the risk of atopic asthma at the age of 12 years. This suggests an immune-mediated effect. To be born SGA increased, while at least 4 months of breastfeeding decreased the risk of non-atopic asthma at the age of 12.

## D44

### How are asthmatic children doing? Follow-up and outcomes of a cohort seen in specialist clinics after 4 years

#### Marco Lozano Nuria^1^*, Moral Gil Luis^2^, Toral Pérez Teresa^2^, García Avilés Belén^3^, Caballero Caballero María^4^, Huertas Sánchez Ana^4^, González Toro Cristina^5^, Olivas Monteagudo Francisca^5^, Martínez Rovira Patricia^3^, Atienza Almarcha Teresa^3^, Rico Rodes Ángela^2^, Jordán Garcia Alfredo^2^

##### ^1^Department of Pediatrics, Hospital Vega Baja, Orihuela, Spain; ^2^Paediatic Respiratory and Allergy Unit, Hospital General Universitario de Alicante, Alicante, Spain; ^3^Department of Pediatrics, Hospital Clínico Universitario de San Juan, Alicante, Spain; ^4^Department of Pediatrics, Hospital Vinalopó, Elche, Spain; ^5^Department of Pediatrics, Hospital General Universitario de Elda, Elda, Spain

**Correspondence:** Marco Lozano Nuria - numalo27@hotmail.com

*Clinical and Translational Allergy* 2018, **8(Suppl 2):**D44

**Introduction**

The objective of this study was to investigate the characteristics and the evolution of an asthmatic children cohort attended in specialist clinics.

**Methods**

A retrospective review of a cohort of patients first seen with asthma in 2012 within 5 different Hospitals. Clinical history data were used regarding patients characteristics, number of visits, treatment and level of control over the following 4 years.

**Results**

656 patients, 62% male; median age of 5 years at the first visit with an interquartile range of 3–8 years; median age of 1 year at onset of first symptoms with an interquartile range of 0–3 years. 72% were considered atopic: 65% aeroallergen sensitization, 47% allergic rhinitis, 24% atopic dermatitis and 14% food allergies.

After 4 years, 26% of the patients continued follow-up, as a result of discharges or non-attendance at clinic.

Amongst the patients who continued follow-up over the 4 years, 33–48% did not require maintenance treatment, antileukotriene was reduced from 46 to 23%, low or medium doses of inhaled corticosteroidswere used by 21–25% and highdose of inhaled corticosteroids±long-acting beta2-agonists were required by 1–6%. Immunotherapy was prescribed to 21% of patients. Between 28 and 35% remained in clinical remission, 51–60% showed good asthma control, 11–15% had partial control and 0–1% poor control.

112 patients (17%) suffered “difficult” asthma, defined by the need for multiple visits, highdose of inhaled corticosteroids±long-acting beta2-agonists, or with poor control of their asthma. There were no differences based on age or sex, but they were more commonlyatopic patients (84–70%, p = 0.003), treated with immunotherapy (54–15%, p < 0.001) and continued followed-up over the 4 years (75–16%, p < 0.001). 2 patients received omalizumab.

**Conclusions**

The majority of patients improved with age and stopped being seen by specialist, but at least a quarter needed continuous specialised follow-up and 17% had “difficult” asthma, although poorly controlled asthma was uncommon.

## D45

### Viral status in asthmatic preschool children during a severe exacerbation: the VIRASTHMA 2 study

#### Stéphanie Lejeune^1^*, Ilka Engelmann^2^, Anny Dewilde^2^, Rodrigue Dessein^3^, Guillaume Pouessel^4^, Heloise Ducoin^5^, Sarah Mitha^5^, Céline Delvart^6^, Caroline Thumerelle^7^, Clémence Mordacq^7^, Muriel Pichavant^8^, Philippe Gosset^8^, Antoine Deschildre^7^

##### ^1^CHU Lille, Pediatric Pulmonology and Allergy Department. Hôpital Jeanne de Flandre, Université Lille, France; ^2^Virology laboratory, EA3610, CHU Lille, Université Lille, Lille, France; ^3^Bacteriology Departement, CHU Lille, Université Lille, Lille, France; ^4^Pediatric Departement, CH Roubaix V. Provo, Roubaix, France; ^5^Pediatric Departement, CH Lens E. Schaffner, Lens, France; ^6^Pediatric Departement, CH Arras, Arras, France; ^7^CHU Lille, Pediatric Pulmonology and Allergy Department. Hôpital Jeanne de Flandre, Université Lille, Lille, France; ^8^Lung infection and innate immunity—Center for infection and immunity in Lille, INSERM 1019, Lille, France

**Correspondence:** Stéphanie Lejeune - lejeunestephanie86@gmail.com

*Clinical and Translational Allergy* 2018, **8(Suppl 2):**D45

**Introduction**

During an exacerbation of asthma in preschool children (< 5 years), viruses, especially rhinoviruses (hRV) are the main triggers of an inflammatory process, leading to clinical symptoms. Previous works have formulated the hypothesis that these patients have a deficient innate immune response to these pathogens, enabling reinfection. The main purpose of the VIRASTHMA 2 study is to describe the inflammatory profile during and after the resolution of a severe exacerbation. This work focuses on microbiological status.

**Methods**

Multicentric prospective study in the Haut-de-France region (France). Asthmatic children aged 1–5 years, were included during a hospitalization for a severe exacerbation. Clinical history, atopic status, viral status (PCR in a nasal swab sample, hRV typing by amplification of the viral protein (VP) 2/VP4 region), bacteriological status (culture of an induced sputum) were assessed during the exacerbation and at steady state, 8 weeks later. We describe the first 105 patients.

**Results**

During exacerbation, a virus was identified in 93% of cases, a hRV in 74% (R + patients), an enterovirus in 13%, an adenovirus in 11%, a respiratory syncithial virus in 7%, a viral co-infection in 27%. Among R + patients, hRVC was found in 77%, hRVA in 23%, no hRVB was found. We observed a higher median PRAM severity score at admission in R + patients versus patients infected with another virus (6 vs. 4, p = 0.004) but no difference in the median length of hospitalization. There was no difference in the prevalence of severe intermittent asthma (26% vs. 24%, p = 1), a trend toward a higher prevalence of atopy (positive prick tests and/or specific IgE) in R + patients (59% vs. 31%, p = 0.053). Among the 67 performed bacteriological cultures, 60% were positive, identifying Haemophilus influenzae (n = 25), Moraxella catarrhalis (n = 20), and Streptococcus pneumonia (n = 12). In all, 37 patients (55%) had a viral/bacterial co-infection. At steady state, 52% were R + , hRVA in 65%, hRVC in 23%, hRVB in 12%. Among these patients, only 28% had clinical signs of viral infection. In all, 35% of patients were R + at exacerbation and at steady state, none of them were infected with the same hRV type at both times.

**Conclusion**

We confirm a high prevalence of hRV infection, especially hRVC, during exacerbation of asthma in young children, frequently associated with a bacteriological carriage or infection. At steady state, virus carriage was frequently observed. Exploring the host immune innate responses could help better understanding the pathogenic role of these microorganisms.

## D47

### The association between sensitisation to house dust mite and food allergy in infants with moderate to severe atopic dermatitis

#### Aideen Byrne^1^*, Maeve McAleer^2^, Siobhan Pyper^3^

##### ^1^Allergy Department, Our Lady’s Children’s Hospital, Dublin, Ireland; ^2^Dermatology Department, Our Lady’s Children’s Hospital, Dublin, Ireland; ^3^Faculty of Medicine, University of Southampton, Southampton, United Kingdom

**Correspondence:** Aideen Byrne - aideen.byrne@olchc.i.e.

*Clinical and Translational Allergy* 2018, **8(Suppl 2):**D47

**Introduction**

Early onset, severe atopic dermatitis (AD) is a recognised risk factor for food sensitisation and food allergy. However, not all infants with AD develop the same profile of food allergy. House Dust Mite (HDM) is known to stimulate both innate and adaptive responses promoting inflammation and barrier dysfunction. Early sensitisation to HDM is associated with development of asthma. We hypothesised that HDM sensitisation amplifies the development of food allergy in an already at risk population.

This aim of this study was to examine the effect of HDM sensitisation on food sensitisation profiles and the development of food allergy in infants with early onset AD.

**Methods**

This study was a retrospective, case controlled study with age matched controls. The patient cohort was identified through laboratory records at Our Lady’s Children’s Hospital Crumlin. All patients with sIgE testing to HDM performed between 2012 and 2016 were identified. However, only patients that had attended for treatment of AD were included in the study. Relevant clinical information was gathered from patients’ case notes.

**Results**

The study population comprised 140 infants with moderate to severe AD aged 4mth to 2 yr (13.8 months ± 5.8) of whom 59% were male and 41% were female. Onset of AD occurred before 3mth in 69% of infants and before 6mth in 93% of infants. No difference in either, time of onset of AD or severity, as measured by SCORAD, was identified between the HDM sensitised and HDM non sensitised populations. 78% of the total population were food sensitised. Sensitisation to peanut, wheat and soy was significantly higher in the HDM sensitised cohort. An association between sensitisation to 2 or more foods and HDM sensitisation was demonstrated (OR 2.28 p = 0.017). 56% of the total population had a history of an allergic reaction to food. Infants with HDM sensitisation were more likely to have food allergy (OR 3.11 P = 0.001). Furthermore, the number of clinically diagnosed food allergies/per child was also increased. A significantly greater number of HDM sensitised patients (39 versus 23) had a history consistent with an allergic reaction to egg (p = 0.006).

**Conclusion**

HDM sensitisation in infants with moderate to severe AD is independently associated with a risk of food sensitisation and food allergy. Early HDM sensitisation may be a useful biomarker of infants to prioritise for early introduction of food allergens in order to prevent development of food allergy.

## D50

### The serum vitamin D level in children with pneumonia

#### Anna Kosowska^1^*, Anna Prescha^2^, Daiva Gorczyca^3^

##### ^1^Department of Clinical Immunology, Wroclaw Medical University, Wroclaw, Poland; ^2^Department of Food Science and Dietetics, Wroclaw Medical University, Wroclaw, Poland; ^3^Third Department and Clinic of Pediatrics, Immunology and Rheumatology of Developmental Age, Wroclaw, Poland

**Correspondence:** Anna Kosowska - annakusek@gmail.com

*Clinical and Translational Allergy* 2018, **8(Suppl 2):**D50

**Introduction**

Lower respiratory tract infections including mainly pneumonia are very important public health problem in children. The majority of evidence suggests that vitamin D plays a role in the body’s immunity and immunomodulation. The role of vitamin D in respiratory infections is unclear. The aim of this study was to assess the association between vitamin D level and lower respiratory tract infection in children.

**Methods**

We recruited100 patients with pneumonia and fifty healthy children aged between 2 months and 15 years. In 60 patients with pneumonia the diagnosis was radiologically confirmed. Complete blood count and C-reactive protein (CRP) level was measured in pneumonia group. Determination of serum 25-hydroxyvitamin D [25(OH)D] level was performed using a competitive enzyme-linked immunoassay.

**Results**

We found no difference between the average level of serum 25(OH)D in pneumonia patients compared to control group (26.16 ± 15.67 ng/ml vs. 27.38 ± 10.90 ng/ml, p = 0.154). However there is a trend towards higher occurrence of vitamin D deficiency (serum level < 20 ng/ml) in patients with pneumonia (45% vs. 30%, p = 0.07). Children with vitamin D deficiency with pneumonia had significantly lower lymphocyte count compared with those without vitamin D deficiency (3.94 ± 2.06 vs. 5.61 ± 2.59 10^3/µl, *p *= 0.0008). We found no differences in total white blood count, platelet count, and CRP level.

**Conclusion**

The number of children with vitamin D deficiency was similar in pneumonia and control group. Due to the high occurrence of vitamin D deficiency, it is recommended that vitamin D level should be measured in children with pneumonia. Decrease of lymphocyte count in children with pneumonia can be associated with 25(OH)D inhibition of lymphocyte proliferation. The effects of vitamin D on infections and immunomodulation require further research.

## D51

### PD-L1^+^ regulatory B cells increase during milk oral immunotherapy

#### Bahar Torabi^1^*, Marieme Dembele^1^, Duncan Lejtenyi^1^, Moshe Ben-Shoshan^1,2^, Bruce D Mazer^1,2^

##### Research Institute of the McGill University Health Centre, Montreal, Quebec, Canada; ^2^ Division of Pediatric Allergy and Clinical Immunology, Montreal Children’s Hospital, McGill University, Montreal, Quebec, Canada

**Correspondence:** Bahar Torabi - bahar.torabi@mail.mcgill.ca

*Clinical and Translational Allergy* 2018, **8(Suppl 2):**D51

**Introduction**

Regulatory B cells (Bregs) have been implicated in venom immunotherapy and their role is being actively studied in non-IgE-mediated food allergies and autoimmune diseases [1–3]. No studies have examined the correlation between Bregs and IgE-mediated milk allergy, nor have the action of Bregs been examined in the treatment of food allergies with oral immunotherapy (OIT). Furthermore, there are currently no phenotypic, transcription factor, or lineage markers unique to regulatory B cells [4], making it a diverse and challenging focus of research. Programmed death-ligand 1 (PD-L1) is one of the surface molecules described on Bregs [5]. PD-L1 is an inhibitory ligand expressed on antigen-presenting cells and tumour cells.

**Methods**

Peripheral blood mononuclear cells were isolated from plasma of milk-allergic children undergoing milk OIT, at baseline and at the end of escalation phase. The cells were cultured for 72 h in various conditions and stained for CD19, CD27, CD38, CD5, CD24, PD-L1, and intracellular IL-10. Conditions included CpG-B, a known stimulator of IL-10-producing B cells, anti-IgM/IgG to activate the B cell receptor, and anti-CD40, IL4, IL21 for plasma cell and memory B cell induction. Milk proteins (casein, α-lactalbumin, β-lactoglobulin) were added to some conditions as specific antigens. Statistical analysis was done using the Wilcoxon matched-pairs signed rank test and a p-value less than 0.05 was considered significant.

**Results**

An interim analysis showed a significant increase in 6 patients in the CD19^dim^PD-L1^+^CD38^+^ population at the end of escalation phase in 3 conditions: CpG-B/anti-IgM/IgG/anti-CD40, anti-CD40/IL4/IL21, and anti-CD40/IL4/IL21 plus milk proteins. The median percentage difference between baseline and end of escalation phase was 8.35, 4.49, and 7.12% for the above conditions, respectively. The majority (89.16%, 95% CI 81.21–95.56%) of the CD19^dim^PD-L1^+^ population in all conditions were CD38^+^ cells.

**Conclusion**

PD-L^+^ regulatory B cells increase during milk OIT and may be part of the mechanism of successful desensitization in children. This population of Bregs could play a role in other allergic diseases as well. Further assessment with a larger sample size is underway.

**References**

1. van de Veen, W., Stanic, B., Yaman, G., Wawrzyniak, M., Sollner, S., Tec, S., Akdis, D.G., Ruckert, B., Akdis, C., Akdis, M. IgG4 production is confined to human IL-10-producing regulatory B cells that suppress antigen-specific immune responses. *J Allergy Clin Immunol* 2013;131:1204–12.
2. Duddy, M., Niino, M., Adatia, F., Hebert, S., Freedman, M., Atkins, H., Kim, HJ., and Bar-Or, A. Distinct effector cytokine profiles of memory and naive human B cell subsets and implication in multiple sclerosis. *J Immunol* 2007; 178(10):6092–9.
3. Noh, J., Noh, G., Kim, H.S., Kim, A.R., and Choi, W.S. Allergen-specific responses of CD19(+)CD5(+)Foxp3(+) regulatory B cells (Bregs) and CD4(+)Foxp3(+) regulatory T cell (Tregs) in immune tolerance of cow milk allergy of late eczematous reactions. *Cell Immunol* 2012; 274:109–114.
4. Tedder, T.F. B10 cells: a functionally defined regulatory B cell subset. *J Immunol* 2015;194:1395–1401.
5. Khan AR, Hams E, Floudas A, Sparwasser T, Weaver CT, Fallon PG. PD-L1hi B cells are critical regulators of humoral immunity. *Nat Commun*. 2015;6:5997.

## D53

### The usefulness of molecular based allergy diagnostics in a secondary pediatric referral center

#### Anne Karina Kjær, Signe Dreier, Ole D. Wolthers*

##### Asthma and Allergy Clinic, Children's Clinic Randers, Randers, Denmark

**Correspondence:** Ole D. Wolthers - akk.odws@dadlnet.dk

*Clinical and Translational Allergy* 2018, **8(Suppl 2):**D53

**Introduction**

Recent guidelines have suggested that molecular based allergy (MA) diagnostics may be used in the diagnostic work-up of selected cases of suspected peanut allergy, birch pollen allergy and associated cross-reactivity, insect allergy and in determining triggering allergens for specific immunotherapy. Guideline reports, however, have concluded that population-based studies involving evaluation of the usefulness of MA diagnostics are needed^1^. The aim of the present study was to evaluate the usefulness of MA diagnostics in a secondary pediatric referral center.

**Methods**

961 consecutively referred children and adolescents aged 0.2–18.8 (mean 6.9) years, 439 girls (45.7%) and 522 boys (55.3%) were included from the prospective Asthma and Allergy in a Secondary Pediatric Referral Center Study (AASP 2002) in the present survey. At referral based on history and clinical signs a suspected diagnosis of an IgE mediated condition was made, and conventional work-up i.e. skin prick testing and assessment of specific IgE panels in the blood and oral provocation tests (when needed) were performed. If a specific diagnosis could not be reached from the investigations, suspected peanut allergy, birch pollen allergy and associated cross-reactivity, insect allergy and triggering allergens for specific immunotherapy were assessed by MA diagnostics.

**Results**

Based on conventional work-up a diagnosis was established in 946 patients (97.7%). MA diagnostics were performed in 15 individuals (2.3%), 7 girls and 8 boys aged 3.2–17.8 (mean 10.6) years. Five cases of suspected peanut allergy, 7 of suspected birch pollen allergy and associated cross-reactivity, and 1 case of insect allergy and grass pollen allergy, respectively, were investigated. In 8 cases a specific diagnosis was established based on MA diagnostics; in 7 cases MA diagnostics could not improve diagnosis.

**Conclusion**

Most patients in a secondary pediatric referral center with suspected IgE mediated allergy can be managed by conventional diagnostic methods. MA diagnostics may be useful in a small and selected subgroup only in whom they may not be helpful in all cases.

**Reference**

1. Canonica GW, Ansotegui IJ, Pawankar R, et al. A WAO—ARIA—GA^2^LEN consensus document on molecular-based allergy diagnostics. World Allergy Organ J. 2013 Oct 3;6(1):17. 10.1186/1939-4551-6-17.

## P1

### Princess Asma—An effective asthma education resource for the pediatric population

#### Louise Kuo^1^*, Rhonda Trotman^1^, Azmain Chowdhury^1^, Jonny Coppel^1^, Lucy Gibson^2^, Rahul Chodhari^3^

##### ^1^University College London, London, United Kingdom; ^2^King’s College London, London, United Kingdom; ^3^The Royal Free London Foundation NHS Trust, London, United Kingdom

**Correspondence:** Louise Kuo - louise.kuo.13@ucl.ac.uk

*Clinical and Translational Allergy* 2018, **8(Suppl 2):**P1

**Introduction**

Asthma is a highly prevalent condition within the pediatric population estimated to affect 1 in 11 children in the UK [1]. Yet, despite the efficacy of asthma medication, asthma-related morbidity and mortality remain major issues with acute exacerbations a leading cause of Hospitalisation in developed countries [2] [3]. It has been predicted that 70% of emergency admissions are preventable [4] and largely attributable to poor patient education. This project aimed to enhance pediatric asthma awareness and understanding through the provision of an informative, interactive and enjoyable resource.

**Methods**

We designed an educational asthma booklet called ‘Princess Asma’ primarily targeted at girls aged 5–10. The efficacy of Princess Asma was determined by administering questionnaires before and immediately after reading the printed booklet. Questions assessed impact on asthma knowledge, testing on: signs and symptoms of an asthma attack; underlying airway changes; possible triggers of asthma attacks and appropriate inhaler and spacer use. Questions regarding perceived changes in understanding and the acceptability of Princess Asma were also included.

In addition, we designed an online questionnaire to record the responses of healthcare professionals to Princess Asma. This enabled us to assess the accuracy and scope of the information provided as well as the feasibility of using Princess Asma in a clinical environment.

**Results**

The responses of 16 children and 11 healthcare professionals, that included nurses and doctors, were assessed.

The pre-booklet and post-booklet mean test scores were 44.8 and 82.3% respectively; this was a significant increase (p < 0.05). The greatest improvements were seen in the proportion of children correctly answering questions related to reliever use, preventer use and underlying changes during an asthma attack. Of the children sampled, Princess Asma also improved perceived understanding of asthma (93.8%), was read with ease (87.5%) and was found enjoyable (87.5%).

Princess Asma was considered accurate, covered key asthma-related information and useful in clinic by all healthcare professionals sampled.

**Conclusion**

This pilot study suggests that Princess Asma is an enjoyable resource that successfully improves asthma understanding and confidence amongst the target pediatric population. Crucially it provides information related to asthma management that has the potential to reduce Hospitalisation rates amongst children. Healthcare professionals have shown they are both willing and able to foresee an application for Princess Asma in clinic. In future, we hope to distribute Princess Asma on a larger scale and study its potential longer-term benefits.

**References**

1. Asthma UK. Asthma facts and statistics [Internet]. [cited 20 April 2017] Available from: https://www.asthma.org.uk/about/media/facts-and-statistics/
2. Moorman JE, Akinbami LJ, Bailey CM, Zahran HS, King ME, Johnson C a, et al. National surveillance of asthma: United States, 2001–2010. [Internet]. 2012.1–67 p. [cited 22 April 2017] Available from: https://www.ncbi.nlm.nih.gov/pubmed/24252609
3. Asher MI, Montefort S, Björkstén B, et al.; ISAAC Phase Three Study Group. Worldwide time trends in the prevalence of symptoms of asthma, allergic rhinoconjunctivitis, and eczema in childhood: ISAAC Phases One and Three repeat multicountry cross-sectional surveys. Lancet. 2006; 368(9537):733–743. Erratum in Lancet. 2007;370(9593):1128.
4. DoH. An Outcomes Strategy for Chronic Obstructive Pulmonary Disease (COPD) and Asthma in England [Internet]. 2011;1–56 [cited 23 April 2017] Available from: http://www.dh.gov.uk/prod_consum_dh/groups/dh_digitalassets/documents/digitalasset/dh_128428.pdf

## P3

### Dynamic spirometry in preschool children

#### Emma Caffrey Osvald^1^*, Katarina Stenberg Hammar^2^, Cilla Söderhäll^2,3^, Gunilla Hedlin^2,4^, Jon Konradsen^2,4^

##### ^1^Astrid Lindgren Children’s Hospital, Stockholm, Sweden; ^2^Department for Women’s and Children’s Health, Karolinska Institutet, Stockholm, Sweden; ^3^Department for Biosciences and Nutrition, Karolinska Institutet, Stockholm, Sweden; ^4^Centre for Allergy Research, Karolinska Institutet, Stockholm, Sweden

**Correspondence:** Emma Caffrey Osvald - emma.caffreyosvald@gmail.com

*Clinical and Translational Allergy* 2018, **8(Suppl 2):**P3

**Introduction**

There are limited data concerning the relationship between preschool lung function and the occurrence of wheeze, in part because dynamic spirometry is technically and clinically difficult to achieve in this age group.

Within this prospective study on children with acute wheeze, we wanted to investigate the feasibility of undertaking dynamic spirometry in preschool children. Further, we wanted to investigate whether there was any association between preschool lung function (FEV_1_%, FEV_1_/FVC%) and episodes of wheeze or use of asthma medication.

**Methods**

Included in the study are 156 children aged 6 months to 4 years who attended the emergency department of a pediatric university Hospital with acute wheeze. The children were followed prospectively and dynamic spirometry was performed using Medikro spirometry software v3.0.2. Reference values for preschool children are published by Quanjer^1^. Data concerning episodes of wheeze, prevalence of atopy and use of asthma medication were ascertained by physician administered questionnaire. Spirometry data showed a normal distribution and student’s t-test was used for analysis.

**Results**

Of the 105 children followed up, 52 children performed spirometry. They were between 4.4 and 5.8 years (median 5 years, IQR 6 months). 28 (54%) children reported at least one episode of wheeze in the previous year and 28 (54%) children had regularly used short-acting bronchodilators.12 (23%) children were prescribed inhaled corticosteroids (ICS) and 16 (31%) children were prescribed a leukotriene receptor antagonist (LRA). There was no statistical difference in FEV_1_% between girls and boys (p = 0.2), however boys with parental atopy had a trend towards a reduced FEV_1_% compared to the rest of the group [82 (52–101) vs. 89 (57–118), p = 0.06]. Our data did not show any significant association between FEV_1_% or FEV_1_/FVC% and the number of wheezing episodes or the use of asthma medication. See Table 1.

**Table 1:** .

Variable	FEV_1_%	FVC%	FEV_1_%/FVC%
Gender			
Girls (n = 21)	89 (57–118)	77 (46–109)	112 (90–118)
Boys (n = 31)	84 (52–116)	74 (41–97)	107 (69–117)
Wheeze			
Yes (n = 28)	84 (52–105)	77 (54–109)	107 (69–117)
No (n = 24)	87 (86–118)	73 (41–97)	111 (90–118)
Parental atopy			
Yes (n = 17)	84 (57–118)	73 (41–97)	108 (69–118)
No (n = 35)	88 (52–116)	80 (57–109)	109 (90–119)
Bronchodilator use			
Yes (n = 28)	88 (64–118)	76 (51–109)	106 (69–119)
No (n = 24)	83 (52–116)	74 (41–97)	111 (85–118)
ICS			
Yes (n = 12)	86 (52–118)	74 (52–97)	109 (69–119)
No (n = 40)	88 (83–105)	77 (62–109)	118 (90–118)
LRA use			
Yes (n = 16)	91 (78–116)	76 (62–94)	109 (85–118)
No (n = 36)	84 (52–118)	75 (41–109)	109 (69–118)

**Conclusion**

Our study shows that it is possible to perform dynamic spirometry in children from 4 years of age. No association was found between lung function measurements and current clinical symptoms. However, the study indicates that boys with parental atopy may be at risk of reduced lung function. Hence, the results obtained may be useful to indicate which children will develop persistent asthma. In a recent study on preschool children with asthma, a closer relationship between pulmonary function and clinical characteristics was found using FEV_0.75_ (L)^2^, suggesting that FEV_0.75_(L) is a more appropriate marker of airway obstruction in this age group.

**References**

1. Quanjer PH, Stanojevic S, Cole TJ et al. Multi-ethnic reference values for spirometry for the3–95-yr age range. ERS 2012 Dec 1;40(6):1324–43.
2. Busi LE, Restuccia S, Tourres R, et al. Assessing bronchodilator response in preschool children using spirometry. Thorax 2017;72:367–372.

## P5

### Hypomagnesiemia and pediatric asthma control

#### Ivan Shishimorov*, Vladimir Petrov, Olga Magnitskaya, Igor Nefedov, Alex Perminov

##### Volgograd State Medical University, Volgograd, Russia

**Correspondence:** Ivan Shishimorov - drshishimorov@gmail.com

*Clinical and Translational Allergy* 2018, **8(Suppl 2):**P5

**Introduction**

Hypomagnesiemia is a possible factor which maintain uncontrolled bronchial asthma. Aim and objectives: Hypomagnesiemia prevalence and relationship with children bronchial asthma control were studied.

**Methods**

There were studied 211 bronchial asthma children (137 boys and 74 girls, average age − 11.63 ± 4.34 y.o.; uncontrolled/partial controlled/controlled bronchial asthma were as 94/56/61). Main assessment parameters were ACQ-5 test, FeNO, spirometry, eosinophil blood count, total IgE, Mg erythrocyte [Mg2 +]e and plasma [Mg2 +]p levels. The associations between [Mg2 +]e or [Mg2 +]p and another assessment parameters were analyzed with Spearman correlation. Receiver operating characteristic (ROC) curves were calculated for separate assessment parameters and asthma control. Associations with asthma control were determined within each combinations of particular parameters using eight separate logistic regression models.

**Results**

Hypomagnesiemia were determined only for [Mg2 +]e (average level was 1.8 ± 0.37 mmol/L). Average plasma Mg level was [Mg2 +]p at normal range (0.95 ± 0.14 mmol/L).There were revealed significant correlations between [Mg2 +]e and age (r = − 0.2, p = 0.004), daytime symptoms (r = − 0.24, p = 0.0004), any activity limitation due to asthma (r = − 0.26, p = 0.0001), SABA usage (r = − 0.26, p = 0.0001), FeNO (r = − 0.21, p = 0.0036), eosinophil blood count (r = − 0.34, p = 0.0001).ROC analysis AUC for [Mg2 +]e was 0.74 ± 0.039. Optimal [Mg2 +]e level was 1.64 mmol/L (J-index 0.48, Se = 88.9 and Sp = 59.5%). Associations with asthma control were determined for logistic regression model “FeNO (≤ 20 ppb) + [Mg2 +]e (> 1.64 mmol/L)”.

**Conclusion**

Magnesium erythrocyte level is important asthma control predictor.

## P7

### Eosinophil derived neurotoxin, a promising biomarker for diagnosis of asthma

#### Tina Ekenkrantz^1^*, Mizuho Nagao^2^, Magnus Borres^1^, Takao Fujisawa^2^, Anders Sjölander^1^

##### ^1^Thermo Fisher Scientific, Uppsala, Sweden; ^2^Allergy Center, Mie National Hospital, Tsu, Japan

**Correspondence:** Tina Ekenkrantz - tina.ekenkrantz@thermofisher.com

*Clinical and Translational Allergy* 2018, **8(Suppl 2):**P7

**Introduction**

Eosinophil Derived Neurotoxin (EDN), an eosinophil granule protein released during eosionophil activation, has been linked to the pathophysiology of asthma and may serve as a biomarker for diagnosis of asthma. We compared a recently developed EDN research assay with established methods for asthma diagnosis (Eosinophil Cationic Protein (ECP), exhaled nitric oxide (FeNO) and blood eosinophil fraction (EOS%)) to demonstrate the diagnostic value of EDN in childhood asthma.

**Methods**

Asthmatic (n = 37) and healthy (n = 86) children aged 6–18 years were analyzed for serum EDN, serum ECP, FeNO and EOS%. We used a novel research assay based on the ImmunoCAP platform to analyze serum EDN concentrations. ECP, FeNO and EOS% were analyzed according to the manufacturer’s instructions.

**Results**

The median concentrations for all four biomarkers were significantly higher in the asthma group compared to healthy controls (P < 0.0001) (EDN 74.1 µg/l (range 19.0–247.9) vs. 28.1 µg/l (range 6.5–230.5); ECP 40.6 µg/l (range 6.4–194.8) vs. 15.6 µg/l (range 10.6–114.4); EOS% 7.3% (range 1.2–20.0) vs. 2.9% (range 0.8–14.0); FeNO 32.5 ppb (range 5.0–181.0) vs. 14.0 ppb (range 0.0–106.0)).

The sensitivity and specificity for EDN was 70 and 76%, respectively, (cut-off 47 µg/l), 70 and 81% for ECP (cut-off 29 µg/l), 69 and 68% for FeNO (cut-off 19.5 ppb) and 76 and 72% for EOS% (cut-off 4.25%). The EDN concentration correlated with the ECP concentration (r = 0.80), EOS% (r = 0.77) and FeNO concentration (r = 0.47).

**Conclusion**

We have shown that EDN has the potential to distinguish between asthmatic and healthy children. EDN correlates with both ECP and EOS% but weaker with FeNO. Combining EDN measurement with one or several of the other biomarkers could have an additive value in the diagnosis of asthmatic children.

## P8

### Self-efficacy, asthma control and quality of life in adolescents with asthma taking part in an intervention study

#### Simone Holley^1^*, Rebecca Knibb^2^, Sue Latter^3^, Christina Liossi^4^, Frances Mitchell^5^, Cilla Snape, Graham Roberts^1,5,7^

##### ^1^Clinical and Experimental Sciences and Human Development in Health Academic Units, University of Southampton, United Kingdom; ^2^ Aston University, Birmingham, United Kingdom and Faculty of Medicine, Southampton, United Kingdom; ^3^ Faculty of Health Sciences, University of Southampton, United Kingdom; ^4^School of Psychology, University of Southampton, United Kingdom and Department of Pediatric Psychology, Great Ormond Street Hospital for Children NHS Foundation Trust, London, United Kingdom; ^5^ The David Hide Asthma and Allergy Research Centre, St Mary’s Hospital, Isle of Wight, United Kingdom; ^6^NIHR/Wellcome Trust Clinical Research Facility, University Hospital Southampton NHS Foundation Trust, Southampton, United Kingdom; ^7^NIHR Southampton Respiratory Biomedical Research Unit, University Hospital Southampton NHS Foundation Trust, Southampton, United Kingdom

**Correspondence:** Simone Holley - s.l.holley@soton.ac.uk

*Clinical and Translational Allergy* 2018, **8(Suppl 2**):P8

**Introduction**

Many adolescents with asthma have poor disease control despite the availability of effective therapies. Research has identified that self-efficacy is an important component of chronic disease self-management and may also be important for quality of life [1]. Adult studies have shown that higher self-efficacy is associated with improved asthma control and better quality of life [2]. We aimed to investigate the relationship between asthma control, self-efficacy, and quality of life adolescents with asthma.

**Method**

We recruited adolescents aged 12–18 years to take part in a randomised controlled trial of a new adolescent intervention aimed at improving asthma self-management. A prescription of at least one preventer medication and an Asthma Control Test (ACT) score of < 21 was a criteria for being included in the study. Self-efficacy to manage asthma was measured using a newly developed instrument—the Adolescent Asthma Self Efficacy Questionnaire [3] quality of life was measured using the Pediatric Asthma Quality of Life Questionnaire ([4]. Questionnaires were completed during the baseline visit in clinic.

**Results**

A total of 71 participants were recruited (AASEQ = 54; PAQoL = 28). We conducted a series of partial correlation co-efficients adjusting for gender as there were significant differences between boys and girls on the ACT and a number of sub-scales. As shown in Table 1, asthma control was not significantly associated with total self-efficacy, although it was in the expected direction (r = 0.24, p = 0.07). Better asthma control was associated with the beliefs subscale of the AASEQ. Better self-efficacy and improved asthma control were both significantly associated with improved quality of life.

**Table 1:** Partial correlations between asthma control, self-efficacy, and quality of life

	Asthma control	QoL symptoms	QoL activity	QoL emotion	QoL total
Asthma control		0.450*	0.375*	0.494*	0.480*
SEQ medication	− .020	− .132	0.027	− .085	− .078
SEQ symptom	0.010	0.064	0.067	0.258	0.208
SEQ beliefs	0.442*	0.603*	0.581*	**0.657**	**0.662**
SEQ friends, family, school	0.134	− .008	0.240	0.232	0.140
SEQ total	0.242**	0.285	0.426*	0.442*	0.398*

*p < 0.05; **p = 0.072

**Conclusion:** Our results suggest that the SEQ and PAQoL measure different constructs and that there is a complex relationship between self-efficacy, quality of life and asthma control. Better self-efficacy may be associated with having better asthma control and quality of life. Alternatively, having better asthma control may underlie better self-efficacy and better quality of life. Future longitudinal studies should assess the direction of causality between these three constructs to identify the ideal target for interventions to improve the life experience of adolescents with asthma.

**References**:

1. Cramm, J. M., Strating, M. M., Roebroeck, M. E., and Nieboer, A. P. (2013). The importance of general self-efficacy for the quality of life of adolescents with chronic conditions. *Social indicators research*, *113*(1), 551–561.
2. Lavoie, K. L., Bouchard, A., Joseph, M., Campbell, T. S., Favreau, H., and Bacon, S. L. (2008). Association of asthma self-efficacy to asthma control and quality of life. *Annals of Behavioral Medicine*, *36*(1), 100–106.
3. Holley, S., Knibb, R., Latter, S., Liossi, C., Mitchell, F., Snape, C., and Roberts, G. (submitted). Development and validation of the Adolescent Asthma Self-Efficacy Questionnaire (AASEQ)
4. Juniper, E.F., Guyatt, G.H., Feeny, D.H., Ferrie, P., Griffith, L.E. and Townsend, M. (1996) Measuring quality of life in children with asthma. *Quality of life research,* 5 (1), 35–46.

## P9

### Asthma exacerbation attendances in a pediatric emergency department

#### Sara Rolim*, Cláudia Teles Silva, Diana Bordalo, Joana Figueirinha, Fernanda Carvalho

##### Serviço de Pediatria, Centro Hospitalar do Médio Ave, Vila Nova de Famalicão, Portugal

**Correspondence:** Sara Rolim - slsrolim@gmail.com

*Clinical and Translational Allergy* 2018, **8(Suppl 2):**P9

**Introduction**

Asthma is the most common chronic disease in pediatric patients. Asthma exacerbations are a frequent reason for emergency department (ED) admissions. The aim of this study is to characterize emergency attendances for asthma exacerbations in a level 2 Hospital setting during the year of 2015.

**Methods**

Retrospective analysis of ED clinical records of children above 3 years of age and adolescents discharged with a diagnosis of “asthma” or “asthma exacerbation” during the year of 2015. The variables analyzed were: epidemiological characteristics, assessment according to the Manchester Triage System (MTS), previous medication and follow-up, treatment in an emergency setting and orientation and therapeutics at discharge.

**Results**

During 2015 there were 292 attendances for asthma exacerbation (8 per 1000 admissions). The median age was 9 years old and 69.5% were male. According to the MTS, 148 (50.7%) patients were classified as yellow, 70 (24%) as orange and 2 (0.7%) as red. Fourty one percent of patients had previous follow-up by a Pediatrician. Most patients were previously medicated with a short-acting bronchodilator (32.9%); 3.5% had an association long-acting bronchodilator/inhaled corticoid; 25% were medicated with an antileukotriene and 27.7% had no previous medication. Those who were treated with inhalation device none brought him to the ED. Hypoxemia exist in 71 (24.3%) patients and 19 of these were Hospitalized. In the ED, 82.2% received nebulized salbutamol, 15.7% nebulized salbutamol + ipatropium bromide and 50% a systemic corticoid (SC). A short course of SC was prescribed at the moment of discharge in 46.6% of patients and in 11% chronic medication was stepped up. Ninety percent were sent to their General Practitioner or Pediatrician, 2.7% to Outpatient Department and 7.5% were Hospitalized. There were 37 (15.5%) readmissions with a mean of 1.22 attendances per patient.

**Conclusion**

In this study there was a large number of ED attendances due to asthma exacerbations during 2015. However, only 10% were sent to Outpatient Department at the moment of discharge or needed Hospitalization. We verified that none of those who had an inhalation device brought him to the emergency room. So ED patients should be considered an important target for asthma education.

## P10

### Multiple atopic sensitization and health care utilization in a cohort of immigrant children from a cross-sectional study of respiratory health and atopy in relation to poor-quality housing in Malmö, Sweden

#### Jens C. Richter*

##### Division of Occupational and Environmental Medicine, Lund University, Lund, Sweden

**Correspondence:** Jens C. Richter - jens.richter@med.lu.se

*Clinical and Translational Allergy* 2018, **8(Suppl 2):**P10

**Introduction**

Exposure to allergens plays a role in the development of atopic sensitization and influences allergic phenotype. The effects of exposure to relevant allergens both from the indoor and outdoor environment are complex. Immigrant children and their families are exposed to new spectra of seasonal and perennial allergens, and often new lifestyle factors, such as diet, and living conditions. Furthermore health literacy and accessibility of healthcare systems will play a role in what impact atopic diseases will have on these populations.

**Methods**

As part of a larger study into the health in its social context of an immigrant population living in poor-quality housing in Malmö/Sweden, families with small children were identified from health care records (child treated in primary care with respiratory illness), and school records (matched for age range). Families were visited in their homes by health communicators fluent in their language. Family and individual level health data, including skin-prick-tests (SPT) for a standard panel of aeroallergens, were analyzed together with environmental exposures (mould, dampness, ETS, crowding and -in the part of the study presented here: health care utilization over 7 years at the primary care level (data linkage to relevant registries)

**Results**

130 families participated, with usable data for 359 children under the age of 13, and 230 parents. The overall exposure to potentially harmful factors was relatively high, the burden of atopy and respiratory diseases was significant. 232 children under the age of 13 had SPTs performed, 48 of which were positive, of these 11 showed sensitization against 2 or more allergens. The spectrum of sensitizations was comparable to a Swedish population (seasonal plant pollen; animal dander, moulds, and house dust mites (HDM). Utilization of primary health care resources amongst the polysensitized children was overall comparable to that of a gender- and age-matched non-polysensitized control group (n = 20) from the same cohort. Higher health care usage was seen in both groups only in children with a documented diagnosis of asthma.

**Conclusion**

In our cohort, it was rather the presence of an asthma diagnosis than polysensitization that drovehigher utilization of primary health care resources, confirming that atopic sensitization in itself is not a disease state, but rather a marker of potential for atopic disease.

## P12

### Prenatal maternal stress and pediatric asthma: a systematic review

#### Ummulkhulsum Y. Ibrahim*

##### School of Medicine, University of Central Lancashire, Preston, United Kingdom

**Correspondence:** Ummulkhulsum Y. Ibrahim - ummul_ashraf96@ymail.com

*Clinical and Translational Allergy* 2018, **8(Suppl 2):**P12

**Introduction**

The prevalence and incidence of asthma are on the rise with children mainly affected [1]. One factor that has been linked to the development of asthma in children is prenatal maternal stress (PNMS) [2]. Various studies have been carried out to determine if there is an association between PNMS and childhood asthma. In this systematic literature review, such studies were critically reviewed to establish the actual association. Establishing the actual association could potentiate the development of interventions to help reduce the incidence and hence prevalence of asthma.

**Method**

This literature review was done according to the PRISMA guideline [3]. Databases such as PubMed, PsychINFO, SCOPUS, EMBASE, MEDLINE and Cochrane Library were searched. Keywords used in the search include: “stress”, “pregnancy”, “child*” and “asthma”.

**Results**

A total of 173 publications were found of which 10 met the eligibility criteria for the review. The eligible studies used different stressors such as bereavement, adverse life events, natural disaster and job strain. The definition of asthma also varied across the studies. 6 studies recorded an overall positive association between PNMS and childhood asthma. 2 studies recorded a gender-specific positive association and 2 other studies recorded an age-specific positive association.

**Conclusion**

There is a potential positive association between PNMS and childhood asthma. However, the studies have various limitations which need to be addressed by future studies in order to establish the actual association and its pattern.

**References**

1. Wright, R. J. Prenatal maternal stress and early caregiving experiences: Implications for childhood asthma risk. Pediatric and Perinatal Epidemiology. 2007 Oct 11; 21(3):8–14.
2. Khashan, A. S., Wicks, S., Dalman, C., Henriksen, T. B., Li, J., Mortensen, P. B., Kenny, L. Prenatal stress and risk of asthma Hospitalisation in offspring: A Swedish population-based Study. Psychosomatic Medicine. 2012 Jun 28;74(6):635–641.
3. Moher, D., Liberati, A., Tetzlaff, J., Altman, D. G., andThe PRISMA Group. Preferred Reporting Items for Systematic Reviews and Meta-Analyses: The PRISMA Statement. PLOS Medicine. 2009 Jul 29; 6(7):1–6.

## P17

### Inflammatory markers of atopic asthma in children

#### Doina Plesca^1^, Ana-Maria Moiceanu Sovarel^2^*, Eugenia Buzoianu^1^, Lavinia Butum^3^, Varvara Toma^1^, Oana Varban^1^, Vlad Plesca^4^, Mariana Moiceanu^1^, Daniela Popeia^1^, Victoria Hurduc^2^

##### ^1^Dr. Victor Gomoiu Children Hospital, University of Medicine and Pharmacy “Carol Davila, Bucharest, Romania; ^2^ Emergency Hospital Elias- Allergology Department, Bucharest, Romania; ^3^Vitan Polyclinic, Bucharest, Romania; ^4^Dr. D. Hociota, O.r.l. Hospital, Bucharest, Romania

**Correspondence:** Ana-Maria Moiceanu Sovarel - anamaria.moiceanu88@gmail.com

*Clinical and Translational Allergy* 2018, **8(Suppl 2):**P17

**Introduction**

Atopic children with asthma and aeroallergen sensitization have often increased blood eosinophils (B-Eos) and elevated fraction of exhaled nitric oxide (FeNO). Both of them are markers of systemic, respectively local eosinophilic inflammation. Eosinophil cationic protein (S-ECP) is another marker of systemic eosinophilic inflammation that might be used in atopic children with asthma.

The aim is to assess the correlation between FeNO values, respectively B-Eos count, and serum eosinophil cationic protein (S-ECP) level in atopic asthmatic children with aeroallergens sensitization.

**Method**

A prospective study was conducted in “Victor Gomoiu” Clinical Children’s Hospital from May 2016 until May 2017. This study included 46 children aged between 5 and 18 years diagnosed with atopic asthma and aeroallergens sensitization.

In each patient FeNO was measured using chemiluminescence analyzer (NIOX MINO). B-Eos were determined using the complete blood count and S-ECP was measured in each patient serum using enzyme-linked immunosorbent assay (ELISA).

The normal FeNO value varied according to age. Thus the value is considered normal < 20 ppb in children aged 5–12 years old and in children aged 12–18 years old < 25 ppb; a normal B-Eos count is < 400 cells/mmc; a normal S-ECP is < 13.3 mcg/l.

Finally the correlation between S-ECP and FeNO, respectively S-ECP and B-Eos, were assessed using Pearson Chi Square test.

**Results**

16 patients had normal S-ECP; out of these 12 had normal of FeNOvalue and 4 had increased FeNO value; 11 had normal B-Eos count and 5 had increased B-Eos count.

30 patients had elevated S-ECP; out of these 12 had normal FeNO value and 18 had increased FeNO value; 9 were with normal B-Eos value and 21 with increased B-Eos value.

Using Pearson Chi Square test to evaluate the correlation between FeNO value and S-ECP in atopic children with asthma and aeroallergens sensitization we have obtained a p value0.0236 (< 0.05, statistically significant).

Using Pearson Chi Square test to evaluate the correlation between B-Eos count and S-ECP in atopic children with asthma and aeroallergens sensitization we have obtained a p value 0.0116 (< 0.05, statistically significant).

**Conclusion**

In atopic children with asthma and aeroallergens sensitization S-PCE value (marker of activated eosinophils) is correlated with other markers of local and systemic eosinophilic inflammation (FeNO and B-EOS).

## P18

### Parents perspective on exercise for asthma-diagnosed children

#### Seda Sirin Kose, Gizem Atakul*, Suna Asilsoy, Nevin Uzuner, Ozkan Karaman

##### Department of Pediatric Immunology and Allergy, Dokuz Eylul University, Faculty of Medicine, Izmir, Turkey

**Correspondence:** Gizem Atakul - drgizematakul@gmail.com

*Clinical and Translational Allergy* 2018, **8(Suppl 2):**P18

**Introduction**

Asthma is the most common chronic illness in childhood. Attacks can be triggered by exercise; when the activity is improper or asthma is uncontrolled. Families often think that they can prevent attacks by limiting their children’s activities.

The aim of this study is to determine the how the parents of asthmatic children think about exercise and whether they need education on asthma and exercise.

**Methods**

A questionnaire was given to parents of 5–17 year-old children with asthma to evaluate their parents’ knowledge on asthma and exercise. Sociocultural level of the family and exercise perspectives of the parents of asthmatic children were asked in this questionnaire.

**Results**

Questionnaires filled by parents of 183 children with mean age of 9.5 ± 3.3 years were evaluated.

49% of the families had a monthly income of 2000 TRY or above.

32% of the participants had family history of asthma.

48% of the parents were smoking, 62% of the parents with high education level were non-smokers while this rate was 46% at lower education levels.

42% of parents stated that exercise did not worsen the children’s asthma symptoms, 88% thought exercise was necessary. 38% children performed regular exercise. 68% parents did not know what their children needed to do before exercise, 61% wanted to be educated about what to do before the exercise.

42% fathers did not want to receive education, while the education level of the fathers increased, the desire to receive education increased (p > 0.05).

38% mothers did not want to receive education, no statistical relation was found between mother’s education level.

As the monthly income ratio of the family increased, the number of cases that regularly exercised increased also.

Parents of regularly exercising children were more likely to exercise than those who did not exercise regularly (p < 0.05).

**Conclusion**

These results support the fact that parents of children monitored in a university Hospital are not informed about exercise. Parents should be informed about physical activity and importance of exercise in asthma. Families should be informed and encouraged about exercise in childhood asthma.

## P21

### When asthma comes with chronic diarrhea

#### Guergana Petrova^1^*, Polina Shahid^2^, Snezhina Lazova^1^, Penka Perenovska^1^, Dimitrinka Miteva^1^, Vera Papochieva^1^, Nadejda Yaneva^3^, Stamatios Priftis^4^, Alexey Savov^3^

##### ^1^Medical University, Sofia, Pediatric Clinic, University Hospital “Alexandrovska”, Sofia, Bulgaria; ^2^Medical University, Sofia, Clinic of Clinical Allergy and Asthma, Univesrity Hospital “Alenadrovska”, Sofia, Bulgaria; ^3^National Genetic Laboratory (NGL), Sofia, Bulgaria; Medical University, Sofia, Bulgaria; ^4^Faculty of Public Health, Medical University, Sofia, Bulgaria

**Correspondence:** Guergana Petrova - gal_ps@yahoo.co.uk

*Clinical and Translational Allergy* 2018, **8(Suppl 2):**P21

**Introduction**

Chronic bronchial obstruction and chronic diarrhea can be signs of one disease or could be two separate disease entities in one patient.

**Case report**

We present two cases of patients presenting with chronic cough (due to bronchial obstruction) and chronic diarrhea.

The first case is a 6 year old boy referred to the clinic with suspicion for cystic fibrosis. The sweat test was negative, genetic analysis revealed only one CFTR mutation with variable significance; bronchodilator response was positive, as well as the Tissue transglutaminase antibody. We concluded the patient have asthma and celiac disease and indicated respective therapy with good effect.

The second case is a male, with history of protracted pneumonia as infant, followed by 10 years of “good health”. At the age of 13 the patient started treatment for asthma with inhaled corticosteroids and pancreatic enzymes due to his “exocrine pancreatic insufficiency”. Due to progressive loss of his lung function he was referred to the clinic at the age of 30 years. The sweat test was positive and we found two disease-causing CFTR mutations. The therapy was modified according ECFS guidelines.

**Conclusion**

Despite knowing the basic disease in a patient, every new symptom should be evaluated in both directions—as a presentation of the underlying disease and as a presentation of a new disease. Without proper investigations we could not precise the diagnosis, which could lead to devastating results (physical and psychological) for the patients.

**Consent to publish**

The authors have obtained parental informed consent of the patient mentioned in the article.

## P25

### The correlation between the basal plethysmography ratio RV/TLC with the decrease of FEV1 post effort after free running test, in allergic children with normal basal spirometry

#### Anxhela Gurakuqi Qirko^1^, Alkerta Ibranji^3^*, Sonila Borici^2^, Mira Xhixha^4^, Esmeralda Shehu^5^, Mirela Hasanaj^6^, Ervin Mingomataj^7^

##### ^1^Allergist MD, PhD, Lecturer at Faculty of Medicine, Tirana, Albania; ^2^Allergist MD, Department of Pediatrics, UHC “Mother Theresa”, Tirana, Albania; ^3^Allergist MD, “At Luigji Monti”, “Our Lady of Good Counsel University”, Tirana, Albania; ^4^Allergist, MD, Polyclinic Nr.1, Tirana, Albania; ^5^Allergist MD, Durres’s Hospital, Durres, Albania; ^6^Allergist MD, Polyclinic Nr.9, Tirana, Albania; ^7^Allergist MD, Department of Allergology, UHC “Mother Theresa”, Tirana, Albania

**Correspondence:** Alkerta Ibranji - alkertaibranji@gmail.com

*Clinical and Translational Allergy* 2018, **8(Suppl 2):**P25

**Introduction**

Very often there is a normal spirometry despite a clinical background of symptoms related to Exercise Induced Bronchoconstriction (EIB), mostly in children (1). Free running test provides more evidences for EIB and Bronchial Hyper Reactivity (BHR), especially in an allergic child. On the ground of a normal basal spirometry, if we do suspect a BHR according to clinical signs, we can perform also the plethysmography. Quite often it’s found an increase of RV and RV/TLC (as % of predicted), reflecting a dynamic “air trapping”. BHR by itself has “air trapping” in distal airways (3). The increase of RV/TLC could be very orientating to presume any BHR, even before any effort bronchial provocation test. Such comparison of RV/TLC with BHR is made with other bronchial provocations, like methacholine, in other studies (2).

Significant correlation between baseline RV/TLC % (cut off > 125% of RV/TLC predicted) (2) with decrease of FEV1 from baseline after 6 min free running test (cut off > 10% decrease FEV1post effort from baseline FEV1) (4). FEV1 is chosen because it has better repeatability and it’s more discriminative than PEF rate (6).

**Methods**

Prospective study, 37 children (5–17 years old, 21 girls and 16 boys), with allergy positive prick test (at least 1 aeroallergen), normal X-ray, no evidence of any infection last month. The majority had the first Lung Function measurement ever. The basal spirometry and plethysmography are performed, followed by a 6 min free running test, only when basal FEV1 > 80%. No nose clip during the run (for maximal cooperation) (5), with median maximal heartbeat 170/min, performed in the same hour interval (16–18 P.M), same conditions of temperature (7) and air humidity (April–May 2016). Spirometry and plethysmography are repeated during the first 5 min after the running test.

**Results**

Except 3 children with pre-effort FEV1 < 80%, which were not allowed to make the running test, the results of 34 children participating, are presented below:


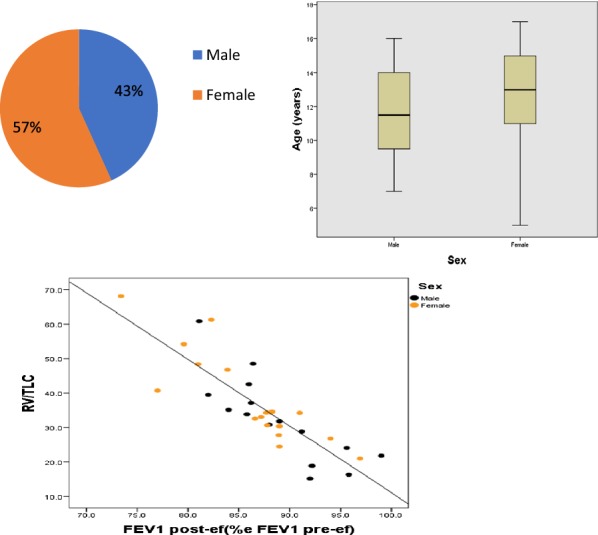


Correlation of basal RV/TLC % predict with FEV1 post effort (% basal FEV1) results statistically significant (p < 0.001).

**Table:** 

Variable	Pearson’s Correlation with basal RV/TLC% predict (95% confidence interval)	p value
FEV1 post effort (% FEV1 basal)	− 0.637 (− 0.901 to − 0.354)	0.001

**Conclusion**

The increase of RV/TLC > 125% of predicted value in baseline plethysmography is very suggestive of dynamic “air trapping” in atopic children with normal basal spirometry, with a significant correlation with BHR after effort.

## P26

### Thymic stromal lymphopoietin, IL-33 and periostin in infants with recurrent wheezing after severe bronchiolitis

#### Maria Luz Garcia-Garcia^1^*, Sergio Quevedo^1^, Cristina Calvo^1^, Ana Moreira^1^, Beatriz Sastre^2^, Ana Tellez^1^, Laura Remedios^1^, Nadia Alvarez^1^, Araceli Marques^1^, Nadia Alvarez^1^, Patricia Alonso^1^, Sara Bellon^1^, Francisco Pozo^3^, Inmaculada Casas^3^, Victoria Del Pozo^2^

##### ^1^Pediatrics Department, Severo Ochoa Universitary Hospital, Madrid, Spain; ^2^Immunology Department. IIS-Fundación Jiménez Diaz, Madrid, Spain; ^3^4respiratory Virus and Influenza Unit. National Microbiology Center (Isciii)., Madrid, Spain

**Correspondence:** Maria Luz Garcia-Garcia - marialuz.hso@gmail.com

*Clinical and Translational Allergy* 2018, **8(Suppl 2):**P26

**Introduction**

Much attention has recently been focused on thymic stromal lymphopoietin (TSLP)(1), IL-33(2) and periostin (3) in allergic diseases, but less is known about their role in viral bronchiolitis and the subsequent development of recurrent wheezing and asthma.

Our first aim was to characterize the response of TSLP, periostin, IL-10, IL-33 and IFN-γ in the nasopharyngeal aspirate (NPA) of infants with severe bronchiolitis. Additionally, we aimed to determine if detection of these proteins in infants hospitalized with bronchiolitis was associated with the severity of the episode and with the development of recurrent wheezing in the 2 years following the acute episode.

**Methods**

A follow-up study of 159 infants hospitalized for bronchiolitis, and a control group of 42 healthy-infants, was conducted from March/2014 to December/2015 at Severo Ochoa University Hospital (Madrid. Spain). Epidemiological and clinical data were collected through a structured questionnaire. Viral detection was performed by multiple polymerase chain reaction in NPA. We analyzed in nasal secretions, IFN-?, IL-10, TSLP, IL-33 and periostin. Patients were followed-up for 2 years after acute bronchiolitis. Data on wheezing episodes and maintenance treatment were collected through a structured questionnaire. The study was approved by the hospital Ethics Committee and informed consent from parents was obtained.

**Results**

At least one virus was detected in 159(87.3%) hospitalized infants. The most frequent were respiratory syncytial virus (RSV):149(70%) and rhinovirus (RV): 42(19.7%). Infants with bronchiolitis had higher levels of TSLP (P = 0.02), IL-33(P < 0.001) and periostin (P = 0.003) than healthy controls. Detectable levels of TSLP and periostin were more frequent in virus-positive than in virus-negative patients (P = 0.05). TSLP and IL-33 were also more common in coinfections, mainly RSV and HRV, than in single-infections (P < 0.05). No patient with bronchiolitis but with negative viral detection had detectable levels of nasal TSLP or IL-33. Infants with hospital stay ≥ 5 days were more likely to have detectable levels of nasal TSLP and periostin after adjusting by age (P = 0.01). Oral corticosteroid for wheezing was more frequently prescribed in the first year of follow-up in infants with positive TSLP (50% vs. 19.4%, p = 0.007). Also, children who required oral corticosteroids during the second year had a higher level of TSLP (69.2 pg/ml vs. 42.4 pg/ml, p = 0.007) and a lower level of IFN-γ (8.6 pg/ml vs. 27.2 pg/ml, p = 0.008) during the acute episode.

**Conclusion**

Severe bronchiolitis is associated with elevated nasal levels of TSLP, IL-33 and periostin. Children who developed recurrent wheezing and need for oral corticosteroids at 2 years of follow-up had significantly higher nasal TSLP and lower IFN-? values.

**Acknowledgements**

This study has been partially supported by FIS (Fondo de Investigaciones Sanitarias—Spanish Health Research Fund) Grants PI12/0129 and FEDER (Fondo Europeo de Desarrollo Regional); Alfonso X El Sabio, University Grant: VI Convocatoria Santander-UAX; CIBER de Enfermedades Respiratorias (CIBERES), a Carlos III Institute of Health Initiative.

**References**

1. Chauhan A, Singh M, Agarwal A, Paul N.Correlation of TSLP, IL-33, and CD4 + CD25 + FOXP3 + T regulatory (Treg) in pediatric asthma. J Asthma. 2015;52:868–72.
2. Wang Y, Wang L, Hua S. Interleukin-33 in children with asthma: A systematic review and meta-analysis. Allergol Immunopathol (Madr). 2017;45:387–392.
3. Li W, Gao P, Zhi Y, et al. Periostin: its role in asthma and its potential as a diagnostic or therapeutic target. Respir Res. 2015;16:57.

## P28

### Vitamin D levels and peak expiratory flow rate correlation in childhood asthma

#### Keya Rani Lahiri*, Vasundhara Chugh, Chinmay Chaudhari, Sadaf Siddiqui

##### D Y Patil School of Medicine, Navi Mumbai, India

**Correspondence:** Keya Rani Lahiri - drkeyalahiri@gmail.com

*Clinical and Translational Allergy* 2018, **8(Suppl 2):**P28

**Introduction**

Vitamin D is vital for lung development and maturation. It acts on vitamin D receptor in airway smooth muscle, enhancing steroid responsiveness. We studied 25(OH) vitamin D levels in asthmatic children. We correlated the levels with severity of asthma and Peak Expiratory Flow Rate (PEFR) pre and post vitamin D therapy and conventional inhalation therapy.

**Methods**

It was a prospective, randomised, comparative study including 60 patients. Institutional ethics committee approval was taken. Informed consent and assent was taken. We studied 30 patients each in the study and control groups. History and clinical examination were recorded in a pre-designed proforma. Both groups received conventional inhalational therapy. The study group received vitamin D supplementation (60,000 IU orally, weekly for 10 weeks). PEFR was measured in the control group and pre and post vitamin D therapy in study group. Statistical analysis was performed using Chi square test and Mann–Whitney test.

**Results**

Age ranged between 6 and 12 years with the mean of the study and control groups being 9.31 ± 1.86 and 9.13 ± 2.12 years respectively. The study comprised of 39 males (65%) and 21 females (35%). Intermittent asthma revealed 8 (13.3%) patients each; Mild persistent asthma had 15 (25%) and 17 (28.4%) patients each; Moderate persistent asthma had 7 (11.6%) and 5 (8.4%) patients each in study and control group. 25(OH) vitamin D levels were deficient in 46 (76.7%) and insufficient in 14 (23.3%) patients. In intermittent asthma patients, 12 were vitamin D deficient and 4 were insufficient; mild persistent asthma, 26 were vitamin D deficient and 6 insufficient; moderate persistent asthma, 8 were vitamin D deficient and 4 insufficient. The pre and post mean vitamin D levels were 16.11 ± 5.72 and 50.46 ± 27.87 respectively (p < 0.0001) in the study group and mean vitamin D level in control group was 15.49 ± 4.9. The mean PEFR values were 195.3 ± 53 and 212.2 ± 46.3 (p < 0.0001) in the study group. The mean PEFR values were 190.06 ± 47.3 and 196.6 ± 41.8 (p = 0.08) in the control group.

**Conclusion**

Vitamin D levels were low in all the 60 asthmatic patients. Administration of vitamin D may prove beneficial as an adjunct to conventional inhalation therapies in asthmatic children. PEFR is a simple, inexpensive diagnostic and monitoring tool for determining airflow obstruction.

## P29

### The Inspire Project: Identifying suitable methods for delivering tailored interventions for reducing asthma-related school absences

#### Christina J. Jones^1^*, Renske McFarlane^1^, Jeremy Mabbitt^2^, Esther Kissling^3^, Kate Gilchrist^4^, Tom Scanlon^1^, Kerry Clarke^4^, Gavin Thomas^4^, Edwina Wooler^5^, Somnath Mukhopadhyay^1,5^

##### ^1^Academic Department of Pediatrics, Royal Alexandra Children’s Hospital, Brighton and Sussex Medical School, Brighton, United Kingdom; ^2^Studybugs, Brighton, United Kingdom; ^3^EpiConcept, Paris, France; ^4^Brighton and Hove City Council, Brighton, United Kingdom; ^5^Brighton and Sussex University Hospitals NHS Trust, Brighton, United Kingdom

**Correspondence:** Christina J. Jones - c.jones@bsms.ac.uk

*Clinical and Translational Allergy* 2018, **8(Suppl 2):**P29

**Introduction**

Children with poor attendance tend to achieve less in both primary and secondary school, as well as later in life. Illness remains the most common cause of school absences. Asthma is known to be one of the most common non-communicable diseases in children. The Inspire Project aims to assess the feasibility of novel methodology to: i) identify common triggers that may predispose children to asthma exacerbations, and ii) investigate parents’ and teachers’ attitudes towards tailored asthma management guidance, in order to capture information to help reduce asthma-related school absences in the future.

**Methods**

Studybugs (studybugs.com) is a unique free online service and app used by 850 UK schools representing 23,000 children. Studybugs offers an efficient and secure way for parents to report their child’s school absence and was utilised for this project. The app was adapted to prompt parents to answer questions about what had triggered their child’s asthma exacerbation and subsequent school absence. Parents and teachers familiar with the app were invited to participate in qualitative interviews to provide feedback on the use of the app as an interventional tool. The quantitative data collection period was from 18th May 2017 to 25th July 2017.

**Results**

Forty-seven asthma absences from 43 unique children were reported, out of 52,454 absence reports. The response rate to the questions was 41.0% (16/39) (in eight episodes questions were not sent out for technical reasons). Parents considered their children’s asthma episodes were caused by hayfever 38.5% of the time, a cold 19.2% and failure of the reliever medication 15.4% of the time. Forty percent of parents planned on taking their child to their GP/asthma nurse/hospital as a result of this asthma exacerbation. Ten parents and teachers consented to be interviewed. Both groups reported ways in which the app might be tailored for intervention purposes, specifically seeing benefit of behavioural interventions, and the best format for delivering health information.

**Conclusion**

The Inspire project is showing the potential for Studybugs to provide a cost-effective means to collect community-wide data and deliver tailored interventions with the aim of improving children’s health. The large proportion of parents reporting hayfever as a trigger may reflect the spring/summer study period and data collection should be extended to account for seasonal variation. The response rate was high and parents and teachers were both receptive to the benefits of delivering guidance on asthma management via the app.

## P30

### Acoustic rhinometry in children: Is it a valuable tool for nasal obstruction?

#### Marialena Kyriakakou^1^, Olympia Tsilochristou^1,2^*, Maria Dimou^1^, Nikos Douladiris^1^, Nikos Papadopoulos^1,3^, Vicky Xepapadaki^1^

##### ^1^Children’s Allergy Department, 2nd Pediatric Clinic, National and Kapodistrian University of Athens, Athens, Greece; ^2^Division of Allergy, Asthma and Lung Biology, King’s College London, London, United Kingdom; ^3^Division of Infection, Immunity and Respiratory Medicine, The University of Manchester, Manchester, United Kingdom

**Correspondence:** Olympia Tsilochristou - ol.tsilochristou@gmail.com

*Clinical and Translational Allergy* 2018, **8(Suppl 2):**P30

**Introduction**

Nasal obstruction is a common symptom in individuals with rhinitis. Acoustic rhinometry (AcR) is a noninvasive tool for objective evaluation of nasal obstruction; it is rapid and requires minimal co-operation of the patient under investigation. AcR is mostly used in adults while it is still under validation in children as the smaller sized cavities affect the measurement of the area-distance function. The Visual Analog Scale (VAS) is a subjective, psychometric evaluation of any symptom severity, including obstruction, based on the patient’s perception. It is simple, and has been reported to be sensitive and suitable for everyday use. Few studies have investigated the correlation of the findings from AcR and VAS in children. The aim of this study was to investigate the accuracy and efficacy of AcR in comparison to VAS in children with nasal obstruction.

**Methods**

The study population was recruited among the children reporting nasal blockage while attending the Rhinitis Outpatient Clinic of the Allergy Service, “PandA Kyriakou” Children’s Hospital, Athens, Greece during 2016–2017. Children (6–9 years old) completed a VAS for nasal obstruction and underwent AcR at the same consultation. The minimum cross-sectional area (MCA) was measured during AcR, which was performed with the A1 Acoustic Rhinometer (GM INSTRUMENTS LTD, Kilwinning, UK). Data were collected and analyzed by SPSS v.23.0. Results are expressed as the mean and standard deviation, with p < 0.05 set as significant.

**Results**

Fourty children (22 boys), aged 6–9 years old were evaluated. Two groups were compared; Group (A) with severe nasal obstruction (VAS values: 7–10 cm) and group (B) with moderate nasal obstruction (VAS values: 4–7 cm). There were no children with mild or no nasal obstruction (VAS values: < 4 cm) in our population. The distributions of MCA and VAS were not normal (Saphiro-Wilc test). No significant correlation between VAS and MCA in both groups were found (group A: VAS vs. MCA; r = 0.49 and group B; r = − 0.09).

**Conclusion**

No statistically significant correlation was documented between AcR values and VAS results in rhinitic children reporting obstruction, suggesting that the methods may identify different aspects of the obstruction and/or that obstruction is experienced differently by each individual. Communication issues may also affect the reporting of obstruction in children. Additional studies are required to identify the optimal use of subjective and objective tools for the management of pediatric rhinitis.

## P31

### Clinical characteristics of childhood obesity in asthma. Bioasma study preliminar results

#### Ana Martinez-Cañavate^1^, M^a^ Amelia Gomez-Llorente^1,2^, Raquel Romero^3^, Natalia Chueca^2,3^, Carolina Gomez-Llorente^2,4, 5^*

##### ^1^Pediatric Unit, Hospital Materno-Infantil, Ciudad Sanitaria Virgen de las Nieves, Granada, Spain; ^2^ibs, Granada Instituto de Investigación Biosanitaria, Granada, Spain; ^3^Hospital Clínico San Cecilio, Granada, Spain; ^4^Department of Biochemistry and Molecular Biology II, University of Granada, Granada, Spain; ^5^CIBEROBN.ISCIII, Centro de Investigación Biomédica en Red Fisiopatología de la Obesidad y la Nutrición

**Correspondence:** Carolina Gomez-Llorente - gomezll@ugr.es

*Clinical and Translational Allergy* 2018, **8(Suppl 2):**P21

**Introduction**

Obesity and asthma are two chronic conditions that affect millions of people. Genetic and lifestyle factors such as diet, physical activity and early exposure to microorganisms are important factors. Recently, two major phenotypes of asthma with obesity have been described: one phenotype of early-onset asthma that is aggravated by obesity, and a second phenotype of later-onset that predominantly affects women (1).

The objective is to describe the main clinical and biochemical differences between obese and normal-weight prepubertal asthmatic children.

**Methods**

We recuited 34 prepubertal asthmatic children, 17 obese and 17 lean, which served as the control group in the Pediatric Unit of the Hospital Materno and from the Hospital Clínico, Granada, Spain. Clinical biomarkers and antropometic measurements were performed according to standardized methods. Data between obese and normal weight children were compared using the *t*-*student* or the non-parametric Mann–Whitney U test. In addition, a Spearman correlation between clinical and biochemical characteristics was performed. Statistical analysis was carried out using the SPSS 22.0 software for Windows (SPSS INC., Chicago, IL, USA).

**Results**

In agreement with the experimental designing, we have found a higher BMI in the obese group than in the control group (*P *=* 0.0001*). Moreover, we have also found a different in the Aspartate-alanine transaminase serum concentration (*P *=* 0.020)* and in the HOMA index (*P *=* 0.015).* In line with this, we have found a positive correlation between the BMI and the Alanine Amino ransferase enzyme (ALT) levels (*r *=0.516 *P *=* 0.007)* and with the HOMA index (*r *=0.655 *P *=* 0.0001).* Another interesting results is the positive correlation between Forced Expiratory Volumen 1 (FEV1) and BMI (r = 0.400 *P *=* 0.030).*

**Conclusion**

In this preliminar study, we have found that asthmatic obese children have higher BMI, ALT and HOMA index than in control group. Regarding asthma characteristic, an increase BMI is associated with a high FEV1 levels. The underlying mechanisms for this associated are not fully elucidated.

This work was supported by the Fundación Progreso y Salud Project number PI-0373-2014 and by Redes temáticas de investigación cooperative RETIC (SAMID RD12/0016/0015.

**Reference**

1. Gomez-Llorente MA, et al. Obesity and Asthma: a missing link. *Int. J. Mol. Sci.* 2017, 18, 1490; 10.3390/ijms18071490

## P32

### Risk factors for asthma severity in children—Synergic effect between tobacco smoke exposure and higher levels of allergen-specific IgE sensitization

#### Filipa Matos Semedo*, Tomaz Elza, Ana Paula Pires, Filipe Inácio

##### Immunoallergology Department, Hospital de São Bernardo, CH Setúbal, Setúbal, Portugal

**Correspondence:** Filipa Matos Semedo - pipa.semedo@gmail.com

*Clinical and Translational Allergy* 2018, **8(Suppl 2):**P32

**Introduction**

Asthma is a prevalent chronic disease that imposes a substantial burden on society. In particular, severe asthma is associated with the greatest share of asthma morbidity and economic burden, although its risk factors remain poorly understood. The aim of this study is to examine associations between risk factors for childhood asthma severity and molecular sensitization profile to perennial allergens.

**Methods**

Cross-sectional study including 72 children with allergic asthma from different outpatient departments (Pediatrics, Allergology, Pulmonology), with sensitization to *Dermatophagoides pteronyssinus*, assessed by total immunoglobulin E (IgE) and major specific IgE allergens Der p 1, Der p 2 and Der p 23. Asthma severity was assessed retrospectively from the level of treatment required to control symptoms and exacerbations. Risk factors for asthma severity were analysed.

**Results**

This study included 72 patients (71% males; mean age, 11.9 years), Median age at onset of asthma symptoms was 2.9 years; 69% also had allergic rhinitis and 19% atopic dermatitis. Potential risk factors for asthma severity were assessed. Concerning allergen sensitization, 37.5% were monosensitized to house dust mites *versus* 62.5% polisensitized patients (25% to *Alternaria alternata*). Wheezing respiratory tract illnesses due to viral infection presented in 71% of children until age of 3. Concerning environmental pollution 44% of patients lived in urban area, 8% had passive smoke exposure and 11% had severe asthma exacerbation in previous year. Patients were classified according to controller treatment for 3 months: mild asthma—as-needed reliever medication or low-intensity controller treatment such as inhaled corticosteroid (ICS) or leukotriene receptor antagonist; moderate/severe asthma—ICS with long acting beta-agonists. Children exposed to tobacco smoke presented with higher severity of asthma (Kruskal–Wallis, p < 0.03). Regarding molecular allergen sensitization, higher levels of specific IgE to Der p 1, Der p 2 and Der p 23 in children exposed to passive smoke were associated with more severe asthma (ANOVA p < 0.000, p = 0.001, p = 0.002, respectively).

**Conclusion**

Evidence supports an association between asthma development and tobacco smoke exposure, suggesting that may also increase risk of IgE sensitization. Current analysis supports passive smoking as a risk factor for asthma severity in children. Higher titters of allergen-specific IgE in children exposed to secondhand smoke correlate with poor asthma control, suggesting synergic effect between higher sensitization levels and passive smoking.

## P35

### Control of allergic rhinitis on the first year of house dust mite sublingual immunotherapy in adolescence

#### Silviya Novakova^1^*, Nonka Mateva^2^, Plamena Novakova^3^, Manuela Yoncheva^1^, Maria Staevska^3^

##### ^1^Internal Consulting Department, Allergy Unit, University Hospital “Sveti Georgi”, Plovdiv, Bulgaria; ^2^Department of Medical Informatics, Biostatistics and e-Learning, Faculty of Public Health, Medical University, Plovdiv, Bulgaria; ^3^Clinic of Allergy and Asthma, Sofia Medical University, Sofia, Bulgaria

**Correspondence:** Silviya Novakova - novakova6607@gmail.com

*Clinical and Translational Allergy* 2018, **8(Suppl 2):**P35

**Introduction**

Allergic rhinitis (AR) is a common problem in adolescence with increasing prevalence [1]. It impacts negatively on physical, social, psychological well-being and impairs productivity at school [2, 3]. Despite the availability of medications some patients may not achieve adequate disease control. By analogy with management of asthma, there is a general trend towards the generalization of the “control” approach to other chronic conditions, such as AR [4]. Furthermore, in the last recommendations of the European Academy of Allergology and Clinical Immunology, disease control is pointed to be one of the clinical outcome parameters in allergen immunotherapy trials for AR [5].

House dust mites (HDM) are among the leading causes of AR [6]. Patients with HDM allergy typically present with symptoms of moderate-to-severe disease [7]. HDM sublingual immunotherapy (SLIT) has been shown to be effective in reducing symptoms and medication requirements in children with AR [8]. However, data on efficacy of SLIT on control of AR is unavailable. The aim of our study was to evaluate control of AR in adolescence after 1 year of HDM SLIT.

**Methods**

In this real life study a total number of 32 adolescence [20 (62.5%) boys; mean age 14.1, SD 1.61] with clinically relevant sensitization to HDM and AR, treated with HDM SLIT were prospectively evaluated on the first year. Control was assessed by Rhinitis Control Assessment Test (RCAT). Total RCAT score 22 or more indicates that AR symptoms are well controlled.

**Results**

All included adolescence underwent HDM SLIT in the course of management of their AR according to the instructions of the manufacture (Table 1).

**Table 1:** .

Gender	n
Boys	20 (62.5%)
Girls	12 (37.5%)
Total	32 (100%)
Age (in years)	
Range 12–17	
Boys	14.2 (SD 1.6)
Girls	14.0 (SD 2.6)
Concomitant asthma:	n − 8 (25%)

Patients—characteristics (*n* number, *SD* standard deviation)

When assessed on the first year 25 (78.13%) of them were well controlled (Fig. 1): 16 (80%) boys and 9 (75%) girls. No significant gender difference in the number of controlled patients was established (p > 0.05) (Fig. 2).

**Fig. 1:**
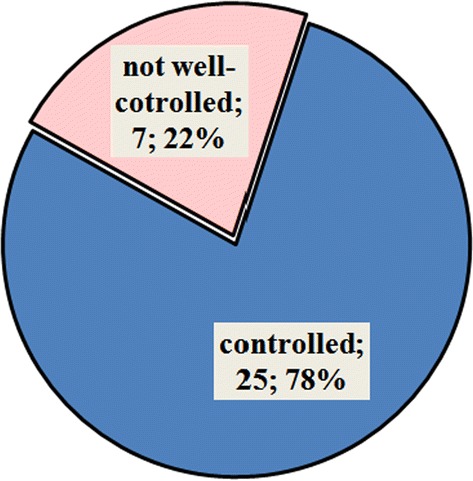
Allergic rhinitis control: patients’ distribution

**Fig. 2:**
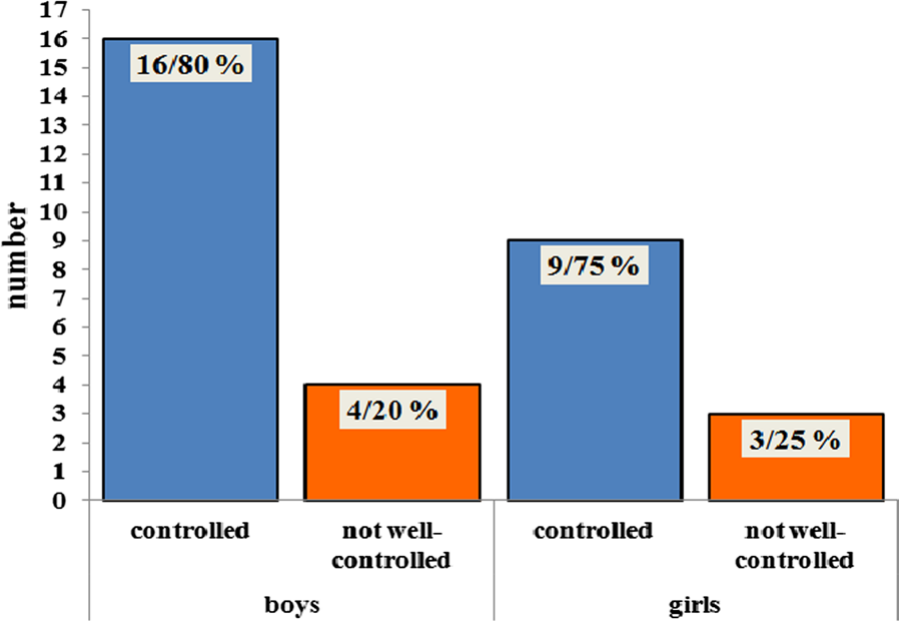
Allergic rhinitis control: gender distribution

Disease duration before SLIT initiation was evaluated: 4.08 (SD 1.96) years in well controlled and 3.86 (SD 0.90) in not well-controlled adolescence. No significant difference in disease duration was established (p > 0.05) (Fig. 3)

**Fig. 3:**
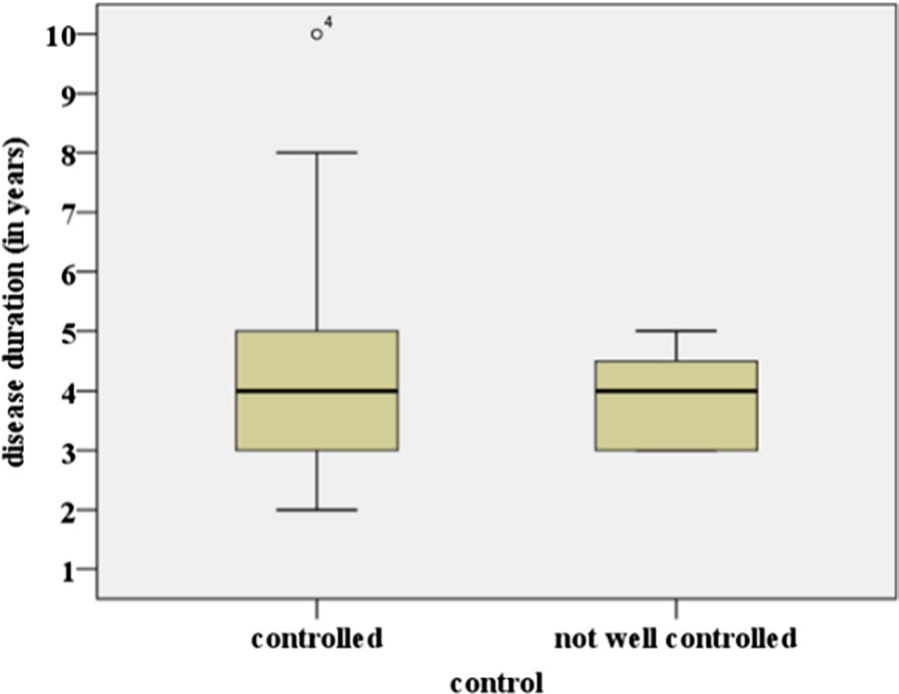
Allergic rhinitis control: relation with disease duration

**Fig. 4:**
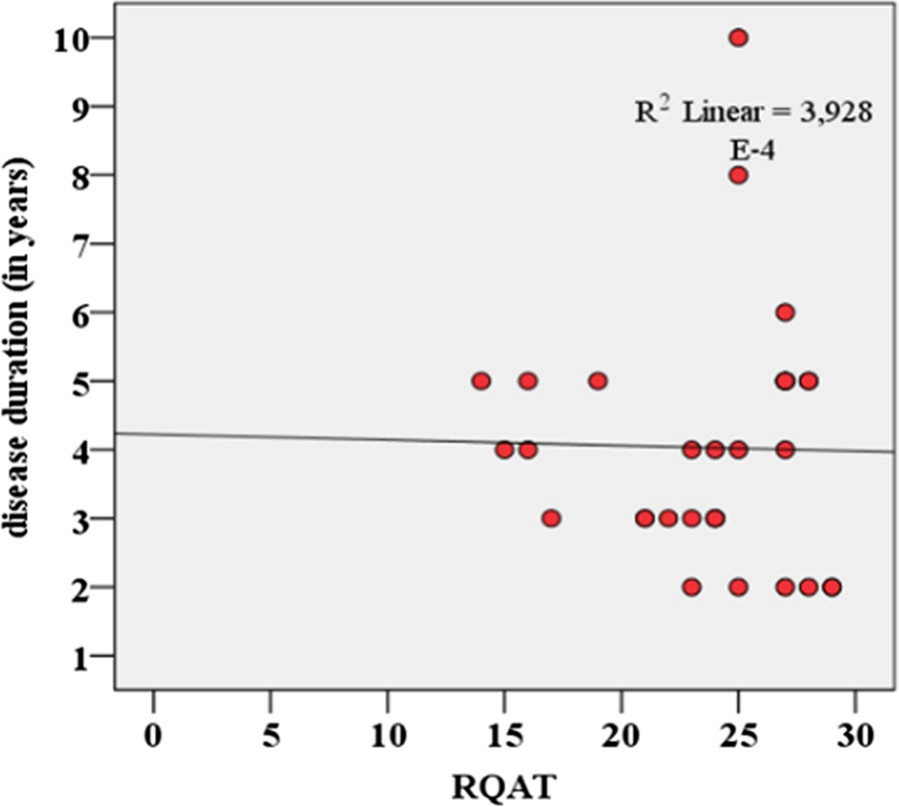
Correlation between disease duration and RQAT score

There was weak negative correlation between disease duration before HDM SLIT and control of AR symptoms when assessed by RCAT (Pearson correlation: − 0.02) (Fig. 4).

**Conclusion**

HDM SLIT could effectively control AR symptoms in adolescence even on the first year of treatment. Disease duration did not influence control, so it is not late to initiate SLIT at any time in eligible patients.

**References**

1. Roberts G, Xatzipsalt M, Borrego L M, Custovic A, et al. Pediatric rhinitis: position paper of the European Academy of Allergy and Clinical Immunology. Allergy. 2013; 68: 1102–1116.
2. Silva CHM, Silva T, Morales N, Fernandes K, et al. Quality of life in children and adolescents with allergic rhinitis. Braz J Otorhinolaryngol. 2009;75:642–649.
3. Mir E, Panjabi C, Shah A. Impact of allergic rhinitis in school going children. Asia Pas Allergy. 2012, 2(2):93–100.
4. Bousquet J, Anto J, Demoly P, et al. Severe chronic allergic (and Related) diseases: a uniform approach—A MeDALL—GA (2) LEN—ARIA position paper. Int Arch Allergy Immunol 158: 216–231, 2012.
5. Pfaar O, Demoly P, Gerth van Wijk, Bonini S, et al. Recommendations for the standardization of clinical outcomes used in allergen immunotherapy trials for allergic rhinoconjunctivitis: an EAACI Position Paper. Allergy 69: 854–867, 2014.
6. Mygind N. Allergic rhinitis. Chem Immunol Allergy. 2014;100: 62–68.
7. Demoly P, Broue-Chabbert A, Wessel F, Charter A. Severity and disease control before house dust mite immunotherapy initiation: ANTARES a French observational survey. Allergy Asthma Clin Immunol 2016; 12:1 -13.
8. Canonica GW, Cox L, Pawankar R, Baena-cagnani CE, et al. Sublingual immunotherapy: World Allergy Organization position paper 2013 update. World Allergy Org I. 2014; 7: 1–52.

## P37

### Establishment of a reference database for skin physical parameters in chinese children and adolescents

#### Jennifer Wing-ki Yau*, Kam-lun Ellis Hon, Ting Fan Leung

##### Department of Pediatrics, The Chinese University of Hong Kong, Prince of Wales Hospital, Shatin, Hong Kong, China

**Correspondence:** Jennifer Wing-ki Yau - wingki_yau@link.cuhk.edu.hk

*Clinical and Translational Allergy* 2018, **8(Suppl 2):**P37

**Introduction**

Atopic dermatitis (AD) is prevalent among children in Hong Kong. The disease is characterized by skin dryness, pruritus, and recurrent flexural dermatitis. The niche of the skin could be described by various physical parameters such as skin hydration (SH), transepidermal water loss (TEWL), redness (erythema) and pigmentation (melanin). Nowadays, biomedical devices for measuring these parameters have been developed, which can be integrated into routine primary care services and research. This study aims to build a dataset of these objective measurements to understand inter-individual variations among healthy children and adolescents in Hong Kong, and compare these measurements between AD patients and healthy controls.

**Methods**

Ethnic Chinese subjects aged below 20 years were recruited during the orientation day of our University and two health promotion events held in October 2016 and April 2017. SH, TEWL, erythema, and melanin were measured by Delfin MoistureMeterSC, Vapometer, and SkinColorCatch at the right mid-forearm respectively. These physical parameters were also measured in participants with self-reported eczema, and their AD severity was evaluated by Nottingham Eczema Severity Score (NESS). Participants with other active skin diseases were excluded. SH and TEWL were log-transformed before analyses. Independent T-test and Spearman’s correlation were used to analyse between-group differences and correlations respectively. All comparisons were made two-tailed, with p value < 0.05 being considered as statistically significant.

**Results**

A total of 245 subjects enrolled in this study. The mean (standard deviation [SD]) age of these healthy participants was 14.1 (4.6) years. Twenty-three percent of these subjects were male. Age was positively correlated with TEWL (ρ = 0.181, p = 0.016) and melanin (ρ = 0.253, p = 0.001). Male had significantly higher readings than female in terms of skin erythema (p < 0.001) and melanin (p = 0.010) measurements. Meanwhile, 69 subjects with AD were recruited during the two activities. The mean (SD) NESS was 5.8 (4.1). Age was positively correlated with TEWL (ρ = 0.237, p = 0.050). TEWL was significantly associated with AD after age adjustment (p < 0.001).

**Conclusion**

Skin integrity, redness and pigmentation are influenced by age and gender. TEWL may be a sensitive biomarker for AD in children and adolescents. This database of different cutaneous physical parameters serves as a reference for further research on different pediatric skin diseases in southern Chinese.

**Acknowledgements**

Funded by Research Committee’s One-off Fund for Research (3132910) of CUHK

## P38

### Henna tattoo allergy: more than para-phenylenediamine

#### Esozia Arroabarren*, Marta Anda, Susana Echechipia, Antonio Rodriguez, Maria Alvarez-Puebla, Blanca Garcia

##### Complejo Hospitalario De Navarra, Pamplona, Spain

**Correspondence:** Esozia Arroabarren - esoziaa@yahoo.es

*Clinical and Translational Allergy* 2018, **8(Suppl 2):**P38

**Introduction**

Temporary henna tattoos are an increasing cause of allergic contact dermatitis (ACD). Most cases have been attributed to the para-phenylenediamine (PPDA), added to increase tattoos' duration and darken their colours. However, other sensitizers may be implied too.

**Case report**

A 7-year old child developed a plaque of itchy erythema followed by localized vesicles and apparent eczema in the forearm. Symptoms began less than 24 h after the application of a temporary black henna tattoo in a street stall, during the summer. Symptoms persisted for 2 months with desquamation and residual hypopigmentation, reproducing tattoo’s shape. The patient made a full recovery. He referred no previous contact with hair dyes or tattoos.

Allergy work-up consisted on patch tests performed with standard set of the Spanish Group for Research in Contact Dermatitis [GEIDC] (TRUE TEST) and natural henna. Positive patch tests were observed at 48 and 96 h with nickel sulphate, P-tert-Butylphenol formaldehyde and Quaternium 15 (probably contained in the tattoo). Natural henna and PPDA tested negative.

**Conclusion**

ACD cases after black temporary tattoos are well known. Black henna tattoos may induce also other different clinical manifestations. Most of them have been attributed to PPDA. However, there have been only 2 previous reports of tattoo allergy due to the presence of formaldehyde in children. P-tert-Butylphenol formaldehyde can induce both sensitization and contact dermatitis during the first exposition.

Spanish Drug Agency has advised about PPDA allergy risk related to black henna tattoos. However, there is no current mention of other agents, such as P-tert-Butylphenol formaldehyde.

**Consent to publish**

The authors have obtained parental informed consent of the patient mentioned in the article.

## P39

### Novel STAT5B mutation causing atopic dermatitis, food allergies, drug allergy, hymenoptera allergy, and complex autoimmunity

#### Cathal O’Connor^1^*, Muriel Sadlier^1^, Alan Irvine^1^, Timothy Ronan Leahy^2^, Grainne O’Regan^1^

##### ^1^Dermatology department, Our Lady’s children’s Hospital, Crumlin, Dublin, Ireland; ^2^Immunology department, Our Lady’s children’s Hospital, Crumlin, Dublin, Ireland

**Correspondence:** Cathal O’Connor - cathaloconnor@umail.ucc.i.e.

*Clinical and Translational Allergy* 2018, **8(Suppl 2):**P39

**Introduction**

Signal Transducers and Activators of Transcription (STAT) proteins play key roles in growth factor-mediated intracellular signal transduction. Somatic gain of function (GOF) mutations in STAT3 and STAT5 have been described in a variety of haematopoietic malignancies which are often associated with autoimmune phenomena. Germline mutations in STAT1 and STAT3 are associated with early-onset lymphoproliferative disease, autoimmunity and increased susceptibility to infection. STAT5B mutations have not been associated with food, hymenoptera, or drug allergies.

**Case report**

An 8 months old girl was referred to our dermatology clinic with an urticarial eruption, present from 4 months of age, worse during cold exposure, and clearing only during intercurrent illnesses. Histology showed a leukocytoclastic vasculitis.

She had developed severe atopic dermatitis at 3 months. She had two reactions to almond involving immediate facial and periorbital angioedema and vomiting. She had a similar reaction to avocado, which was also associated with urticaria. She developed erythema and facial angioedema following treatment with oral penicillin. She developed facial angioedema and dysphonia following a wasp sting. All episodes responded to antihistamines. Interestingly, skin prick testing to almond and avocado was negative.

She had multiple features of autoimmunity, including alopecia totalis, aphthous ulcers, attacks of abdominal distension and diarrhea, and peripheral neuropathy. Following episodes of bleeding gums and epistaxis, von Willebrand disease was diagnosed.

She developed a morbilliform rash following MMR vaccination but has not otherwise demonstrated increased susceptibility to infection.

Investigations revealed eosinophilia, and intermittently reduced alternative and classical complement pathways. Inflammatory markers and amyloid A were normal. Immunodeficiency and autoimmune workup was negative. Sequencing of AIRE, NEMO, NOD2, STAT1, STAT3, and DOCK8 did not reveal pathogenic mutations. However, a novel heterozygous GOF mutation was detected in STAT5B p. N642H.

There is a strong family history of autoimmunity with coeliac disease, autoimmune thyroiditis, hyperparathyroidism, hypoparathyroidism, vitiligo, arthritis, lupus, inflammatory bowel disease, Behçet’s disease, and recurrent early pregnancy loss in first and second degree relatives. Her parents do not carry the STAT5B mutation.

Ruxolitinib, a JAK1/2 inhibitor was initiated in October 2016, with excellent effect.

**Conclusion**

This is the first report of isolated somatic STAT5b GOF mutation in childhood causing the constellation of atopic dermatitis, food allergies, hymenoptera allergy, eosinophila, and complex autoimmunity. The complete phenotype of this condition has not yet been determined.

**Consent to publish**

The parents of the patient in this case have provided written informed consent for publication of her case.

**References**

1. Kontro M, Kuusanmaki H, Eldfors S, et al. Novel activating STAT5B mutations as putative drivers of T-cell acute lymphoblastic leukaemia. Leukaemia. 2014;28(8):1738–42
2. Toubiana J, Okada S, Hiller J et al. Heterozygous STAT1 gain-of-function mutations underlie an unexpectedly broad clinical phenotype. Blood. 2016; 127(25):3154–64
3. Ma CA et al. Somatic STAT5b gain-of-function mutations in early onset nonclonal eosinophilia, urticaria, dermatitis, and diarrhea. Blood 2017 Feb 2;129(5):650–653.
4. Kanai T, Jenks J, Nadeau KC. The STAT5b Pathway Defect and Autoimmunity. Front Immunol. 2012 Aug 14;3:234.

## P40

### A distinct T helper subset contributes to the pathogenesis of classically recognized Th2-mediated Food allergic disorders

#### Erik Wambre*, Blake Rust, Nahir Garabatos Leitón, Veronique Bajzik, Kelly Aldridge

##### Benaroya Research Institute at Virginia Mason, Seattle, Washington, United States of America

**Correspondence:** Erik Wambre - EWambre@benaroyaresearch.org

*Clinical and Translational Allergy* 2018, **8(Suppl 2):**P40

**Introduction**

Food allergies manifest in a wide array of clinical symptoms and can lead to fatal anaphylactic reactions. Response to currently available immunotherapies varies greatly and some food allergic patients undergoing therapy experience adverse reactions. Biomarkers that can be used to address such heterogeneity will be of significant clinical interest.

**Methods**

Peanut allergy was used as an experimental model. A double-blind placebo-controlled food challenge (DBPCFC) was performed to rule out food allergy in a patient with a history of allergic symptoms. Direct ex vivo pMHCII tetramer staining and CD154 T cell assay were used to compare the functions and phenotypes of peanut specific T cell responses, both in the context of disease and clinical intervention.

**Results**

In subjects reacting to DBPCFC, we observed two distinct immunotypes in the Peanut-specific T cell populations: a classical allergic TH2A phenotype (CD27-CRTH2 + CCR4 + CCR6-) and a Th17-like phenotype (CD27-CRTH2-CCR4 + CCR6 +). Interestingly, peanut allergic subjects with high IgE levels tend to express a predominantly CRTH2 + phenotype while those with lower IgE levels tend towards a CCR6 + phenotype. We also observed a correlation between decrease of peanut-specific TH2A cells and achievement of peanut desensitization from oral immunotherapy.

**Conclusion**

Our study emphasizes the heterogeneity of allergen-reactive effector T cell responses in peanut allergic subjects, with two mutually exclusive immunotypes associated with food allergy. These immunotypes are likely the result of different immunologic mechanisms and therefore may require different immunotherapeutic approaches to bring about resolution. Enumeration and characterization of Food allergen-specific T cells can provide essential information about the potency of the immune response and can serve as useful biomarker in study allergic diseases. Peanut allergic Immunotypes dictate both disease manifestation and clinical treatment outcomes following Peanut OIT.

## P41

### Growth factors play major role in maturation process of airway epithelium in presence of atopy

#### Taka Styliani^1*^, Georgountzou Anastasia^1^, Maggina Paraskevi^1^, Kokkinou Dimitra^1^, Stefanopoulou Panagiota^1^, Stamataki Sofia^2^, Papakonstantinou Aliki^1^, Andreakos Evangelos^3^, Prokopakis Emmanouil^2^, Papadopoulos Nikolaos^1,4^

##### National and Kapodistrian University of Athens, 2nd Pediatric Clinic, Athens, Greece; ^2^ University of Crete, Department of Neurology and Sensory Organs, Heraklion, Greece; ^3^ Biomedical Research Foundation Academy of Athens, Athens, Greece; ^4^ University of Manchester, Center for Pediatrics and Child Health, Manchester, United Kingdom

**Correspondence:** Erik Wambre - takastella@hotmail.com

*Clinical and Translational Allergy* 2018, **8(Suppl 2):**P41

**Introduction**

Reepithelialization of the airway mucosa is an essential step toward restoring a normal functionalprotective barrier during the repair of airway epithelial wounds. In the developing lung, growth factors specify patterns of branching, and control airway size and cell fate, among other functions. In the fully developed lung, these signals are presumably balanced to maintain cellular activities at equilibrium, so that normal lung structure and function are preserved. There is still a significant gap of knowledge on the maturation process of growth factors from birth to adulthood and the influence of viral infections in this balance contributing to the generation of abnormal atopic epithelium.

The aim of the present study was to determine the role of growth factors in the maturation process of respiratory epithelium and to investigate the role of viral infections.

**Methods**

Primary nasal epithelial cells (NECs) in all ages (0–60 years) were derived from healthy (n = 26) and atopic (n = 37) donors. NECs were cultured and infected with Human Rhinovirus 1B (RV1B) and 16 (RV16). Growth factor (EGF, FGF2, VEGFA, PDGFAA and TGFA) were measured in uninfected and infeted cell culture supernatants at 48 h. Age-related reduction of remodeling and angiogenetic factors EGF, FGF2 and TGFA (p < 0.05) was observed in healty NECs.

**Results**

Opposing results were observed in atopic NECs, with increasing age-related values of these factors. Direct comparison of regression lines between healthy and atopic individuals,

different slopes were observed (p < 0.05) in EGF and FGF2 factors. RV1B induce higher levels of EGF and FGF2 (p < 0.05) and lower VEGF levels compared to uninfected condition (p < 0.05) in both healthy and atopic NECs. RV16 induce EGF, FGF2 and PDGFAA (p < 0.05) in both healthy and atopic NECs. The expression of TGFA do not influced by RV1B or RV16.

**Conclusions**

This is the first study investigating the maturation process of growth factors in airway epithelium. Atopic epithelium don’t seem to follow the same evolutionary line as healthy. The age-related reduction of basic growth factors (FGF2 and EGF) in healthy NECs reflects the temporal distancing from embryonic stages with strong developmental changes. On the other hand, the atopic NECs do not reduce these factors with age, reflecting their need of remodeling. The viral infections seem to induce strongly these factors and differentially influence healthy and atopic NECs during lifetime.

## P42

### Stevens–Johnson syndrome allegedly induced by herbal medicine

#### Shahid P.^1^*, Drenovska K.^2^, Shahid M.^2^, Vassileva S.^2^, Popov T.^1^

##### Department of Allergy, Medical University, Sofia, Bulgaria; ^2^ Department of Dermatology and Venereology, Medical University, Sofia, Bulgaria

**Correspondence:** Shahid Polina - poli.mu@gmail.com

*Clinical and Translational Allergy* 2018, **8(Suppl 2):**P42

**Introduction**

Stevens-Johnson syndrome (SJS) is a severe life-threatening skin condition with high mortality rate. Drugs are considered one of the most common causes of SJS. Herbal medicines may also be responsible for this syndrome even though there are only a few cases described in the literature. We report SJS occurring after administration of herbal medicine containing extracts from thuja (*Thuja occidentalis*), coneflower (*Echinacea purpurea et pallida*), and wild indigo (*Baptista tinctoria*) in the context of macrolide intake for current infection.

**Case report**

A 14-year-old boy with no history of pollen sensitization and atopy was prescribed a product of mixed herbs with proposed immunostimulating effect along with midecamycin treatment for fever and sore throat. Four days after initiation of therapy the child developed severe oral and genital erosions, bilateral conjunctivitis and worsened general condition. High fever and vomiting were accompanied by cough with purulent expectoration. Six days later, urticarial rash followed by “target” lesions on the face, trunk and limbs developed. Laboratory studies revealed markedly elevated erythrocyte sedimentation rate, mild proteinuria, hematuria, and leukocyturia. Systemic corticosteroids were used as a primary treatment and both herbal tablets and antibiotic were discontinued. The general symptoms quickly resolved and the skin and mucosal lesions completely epithelialized within 2 weeks.

**Conclusion**

SJS is a dermatological emergency that may result in severe morbidity and mortality. A comprehensive literature review revealed only isolated reports of SJS induced by herbs. In recent years herbal medicine consumption has increased while the safety of herbal drugs remains underinvestigated. In the present case, the intake of herbal tablets was combined with macrolide antibiotic, which could also be suspected as inducing agent. As macrolides have an excellent safety record with very few allergic or pseudo-allergic reactions, we rank midecamycin second to the herbal mix in terms of possible cause of SJS in our case. On the other hand, extracts from *Echinacea* spp., *Echinacea purpurea* and *Echinacea pallida* in our case, as well as wild indigo extracts, are increasingly reported to cause severe allergic reactions. In cases of multicomponent therapy, it is a challenge to identify the offending agent, and subsequent treatments strategies in these patients should be carefully tailored.

**Consent to publish**

The parents of the patient have provided written consent to publish.

## P44

### A case of cutaneous mastocytosis

#### Andreia Forno^1^, Alexandra Rodrigues^1^*, Pereira Bárbara^2^, Andreia Barros^1^, António Jorge Cabral^1^

##### ^1^Pediatrics Department, Hospital Dr. Nélio Mendonça, Funchal, Portugal; ^2^Dermatology Department, Hospital Dr Nélio Mendonça, Funchal, Portugal

**Correspondence:** Alexandra Rodrigues - alexandrabrod@gmail.com

*Clinical and Translational Allergy* 2018, **8(Suppl 2):**P44

**Introduction**

Mastocytosis is a rare disorder which prevalence is unknown, and is characterized by excessive mast cell accumulation. In the pediatric population the majority is limited to the skin (cutaneous mastocytosis) and presents itself during the first year of life.

**Case report**

The prevalence of anaphylaxis reported in the presence of this disease is higher than that reported in the general pediatric population, and more severe anaphylaxis symptoms have been documented in these patients.

The authors present the case of a 2 year old boy, presenting with brown macules and small purpuric skin lesions since 7 months of age. There was no history of systemic symptoms. Upon examination the patient presented the skin lesions in the head, chest and perineum. The pathognomonic Darier’s sign was present, establishing the clinical diagnosis. The rest of the physical examination was normal. Laboratory blood tests were normal, with no eosinophilia and total IgE within the normal range. Serum tryptase levels results have been ordered and are still pending. The patient initiated treatment with antihistamines and was prescribed a self-administration adrenaline pen. Because of the risk of mastocyte degranulation leading to systemic reactions, vaccination was performed in a Hospital setting.

**Conclusion**

Cutaneous mastocytosis usually has a transitional and benign course in the pediatric population, although there is the possibility of systemic reactions. A detailed anamnesis and laboratory tests, including a blood count and serum tryptase levels, is recommended in all patients. The first-line treatment is the use of H1 antihistamines, but the most important measure to implement is parental education, avoiding triggers of mastocyte degranulation and early recognition of systemic symptoms.

**Consent to publish**

The parents of the patient have provided written consent to publish.

## P49

### A retrospective claims database analysis of allergy testing amongst allergists, dermatologists and pediatricians for atopic dermatitis/eczema, allergic reactions, and urticaria in children

#### Chikoti M. Wheat^1∞^, Heather C. Rosengard^1^*^∞^, Corinne A. Keet^2≠^, Bernard A. Cohen^3≠^

##### ^1^Johns Hopkins University School of Medicine, Department of Dermatology, Baltimore, USA; ^2^Johns Hopkins University School of Medicine, Department of Pediatrics, Baltimore, USA; ^3^Division of Pediatric Dermatology, Johns Hopkins Childrens’ Center, Baltimore, USA

^∞^First authors that contributed equally to this work

^≠^Senior authors that contributed equally to this work

**Correspondence:** Heather C. Rosengard - hroseng1@jhmi.edu

*Clinical and Translational Allergy* 2018, **8(Suppl 2):**P49

**Introduction**

Interpreting allergy tests can be difficult, as most have relatively poor specificity. Little is known about the allergy test ordering practices of generalists and specialists caring for children.

The objective is to determine the rates at which allergists/immunologists, dermatologists, and pediatricians order allergy tests for children diagnosed with atopic dermatitis/eczema, allergic reactions, and urticaria.

**Methods**

A total of 191,388 children diagnosed with atopic dermatitis/eczema, urticaria, or allergic reactions were included in a retrospective analysis of the Humana database (2008–2015). Poisson regressions were used to quantitatively compare the number of allergy tests ordered by provider type.

**Results**

Allergists and immunologists consistently ordered percutaneous testing, specific IgE, and total IgE tests at the highest rate. For example, for atopic dermatitis/eczema, allergists/immunologists ordered 0.35 percutaneous tests and 0.32 specific IgEs per patient, compared to 0.08 and 0.08 for dermatologists and 0.05 and 0.07 for pediatricians, respectively. However, with the exception of percutaneous tests for urticaria, pediatricians ordered the highest total number of allergy tests: 50,689 compared to 20,090 ordered by dermatologists and 2,904 ordered by allergist/immunologists. Since these are claims data, we cannot comment on whether the allergy tests in question were appropriately ordered and interpreted.

**Conclusions**

Because pediatricians and dermatologists order many more allergy tests than allergists/immunologists, it is critical that education efforts target these specialties to ensure high-value, cost-conscious care.

## P50

### Food sensitization in patients with atopic dermatitis according to severity in a specialized outpatient clinic in Rio de Janeiro

#### Ekaterini Goudouris*, Camila Lira, Evandro Prado, Fernanda Pinto Mariz, Heloiza Silveira, Maria Fernanda AMA Motta

##### IPPMG—UFRJ, Rio de Janeiro, Brazil

**Correspondence:** Ekaterini Goudouris - egoudouris@gmail.com

*Clinical and Translational Allergy* 2018, **8(Suppl 2):**P50

**Introduction**

Atopic dermatitis (AD) is multifactorial disease. Food sensitization is quite common, mainly to cow’s milk and egg, according to literature. We aim to report sensitization to foods in patients with AD according to their severity in a specialized outpatient clinic in Rio de Janeiro.

**Methods**

Retrospective study, through a review of medical records of patients who started follow up between January 2016 and May 2017. We classified patients as: mild AD (MAD)—SCORAD < 25 and moderate-severe AD (MSAD)—SCORAD ≥ 25. We studied the total IgE levels and specific IgE profile, measured by the fluoroimmunoassay method, for cow’s milk (CM) and its proteins, egg yolk, wheat, soy, corn, peanut and beef. IgE values ≥ 0.35 kU/L were considered positive. Patients sensitized to one food were considered “monosensitized”, and those sensitized to 2 or more foods, “polysensitized”.

**Results**

Among 55 patients, aged between 3 months and 11 years (mean = 4.5 years), 15 (27%) were of the MAD group and 40 (73%), MSAD. Total IgE assay revealed that 33% of MAD and 39% of MSAD presented values > 3000 IU/mL. In the MAD group, 10 patients (66.6%) had positive specific IgE results, being 40% monosensitized, 50% polysensitized and 10% non-sensitized. In the MSAD group, 34 patients (85%) had positive specific IgE, being 18% monosensitized, 53% polysensitized and 29% non-sensitized. Among the foods most implicated in MAD, CM and egg were identified, followed by wheat. In MSAD, the egg is the most important food, followed by CM, wheat and peanuts.

**Conclusion**

**T**he analysis of the two groups showed that total IgE level was not related to the severity of AD and no significant differences were found in sensitization to one or more foods according to the severity of AD. Egg and CM are the foods most implicated in our patients both with mild and moderate-severe AD.

## P51

### Profile of pediatric patients in primary immunodeficiency investigation: why they are referred and by whom

#### Ekaterini Goudouris*, Camila Lira, Evandro Prado, Fernanda Pinto Mariz, Heloiza Silveira, Maria Fernanda AMA Motta

##### IPPMG - UFRJ, Rio de Janeiro, Brazil

**Correspondence:** Ekaterini Goudouris - egoudouris@gmail.com

*Clinical and Translational Allergy* 2018, **8(Suppl 2):**P51

**Introduction**

Primary immunodeficiencies (PIDs) are a heterogeneous group of immune system defects, which are characterized by recurrent infections, as well as allergic, inflammatory, autoimmunity, and malignant manifestations. Although relatively rare, they are increasingly known among general pediatricians and pediatric specialists, but usually focusing on infectious processes. We aim to describe the profile of referral of children and adolescents to an immunology service of a pediatric university hospital in Rio de Janeiro-Brazil.

**Methods**

Retrospective study, with data collection in medical records of patients attended in the period from 2013 to 2016. We analyzed the following data: origin and reason of the referral, diagnostic hypothesis formulated and final diagnosis.

**Results**

Of the 217 patients evaluated, 93 were referred by doctors from different pediatric specialties (68.8% from the same university institution and 31.2% from outside) and 124 from general pediatricians (59.7% from the same institution and 40.3% from outside). The most frequent reasons for referral were: recurrent infections (n = 100; 46.1%), severe infections (n = 21; 9.7%), recurrent fever (n = 14; 6.5%), and angioedema without urticaria (n = 14; 6.5%). Among recurrent infections, the most common was pneumonia (n = 31; 34.44%). Of the 146 patients whose diagnostic investigation was completed, the diagnosis of PID was excluded in 65% (n = 95), with the majority being referred for recurrent pneumonia. In 51 patients (35%) the diagnosis was confirmed, being repetitive infections, angioedema without urticaria and alterations in the levels of immunoglobulins the main reasons for referral in these cases. Of the patients referred by general pediatricians, the diagnosis of PID was confirmed in 28.6%, whereas by other specialists in 34.7% and by immunologists, in 76.9% of cases. The diagnostic hypotheses formulated were confirmed in 23 patients, 8.3% (n = 7) of those referred by general pediatrics, 11.1% (n = 7) by specialists non-immunologists, and 69.2% (n = 9) by immunologists.

**Conclusion**

The diagnosis of PID has not been confirmed in most cases, as seen in many other studies. Most referrals are made by general pediatricians, although they do not fit the diagnostic hypothesis in most cases. Repetitive infections are the main cause for diagnostic suspicion among these professionals. Recurrent fever and other manifestations are uncommon reasons for referral. Continued educational work with general pediatricians and other pediatric specialties is necessary.

## P53

### Pediatric refractory urticaria … what else?

#### Barathi Rajendra^1^*, Jin Ho Chong^2^

##### ^1^Department of General Pediatrics and Adolescent Medicine, KK Women’s and Children’s Hospital, Singapore, Singapore; ^2^Department of Pediatric Dermatology, KK Women’s and Children’s Hospital, Singapore, Singapore

**Correspondence:** Barathi Rajendra - Barathi.Rajendra@singhealth.com.sg

*Clinical and Translational Allergy* 2018, **8(Suppl 2):**P53

**Introduction**

Urticaria is a fairly common eruption in childhood. In the vast majority of cases, it is a skin-limited disease, running a benign course. Rarely it may signify more serious underlying disease. We present a case of a 5-year old boy who was treated for refractory urticaria for 6 months before he developed other symptoms that finally led to the diagnosis.

**Case report**

A 5-year old Chinese boy presented with prolonged fever of 2 months duration associated with an intermittent urticarial looking rash. He did not have any constitutional symptoms nor exhibit any localizing signs on physical examination. Extensive work up was performed including blood work (revealing mild transaminatis with minimally raised C-reactive protein and erythrocyte sedimentation rate and mild anaemia); microbiological studies, abdominal ultrasound scan, chest X-ray, autoimmune screen, bone marrow aspirate and 2-D echocardiography. A cervical lymph node biopsy revealed dermatopathic lymphadenitis. A focus of infection was never found and the child’s fever lysed after 5 days of intravenous Ceftriaxone. He was reviewed in the outpatient department over the next 2 months where he remained clinically well, but parents reported 2 further separate episodes of fever associated with an urticarial rash. He continued to demonstrate mild anaemia with minimally raised inflammatory markers on blood investigations. Prolonged courses of anti-histamines and leukotriene receptor antagonists did not lead to resolution of the rash. Three months after presentation, the parents agreed to a skin biopsy of the urticarial rash. However at this time, parents reported 1 week of high spiking fevers to 40 °C with rash. Clinical examination revealed a linear rash with left wrist effusion. A diagnosis of systemic onset juvenile idiopathic arthritis (JIA) was made. The child was started on Prednisolone and Methotrexate and continues to be monitored by the Rheumatologists.

**Conclusion**

The most common presenting features of systemic arthritis in children are fever, arthritis and rash; the rash can be fleeting and correlates to the acute febrile episodes. It is not unusual for the joint symptoms to develop months after the initial fevers and rashes. Our patient had a diagnosis of chronic idiopathic urticaria refractory to treatment. However urticarial ‘mimickers’ are often seen in the context of fevers and extra cutaneous manifestations. It is important to be aware of evolution of symptoms over time.

**Consent to publish**

The parents of this patient consented to the presentation of his case

## P54

### Measurement properties of quality-of-life measurement instruments for caregivers of children with atopic eczema: Systematic review

#### Christina J. Jones^1^*, Daniel Heinl^2^, Aaron M. Drucker^3^, Susanne Brandstetter^2^, Frank Dodoo-Schittko^2^, Tracey Sach^4^, Christian Apfelbacher^2,5^

##### ^1^Academic Department of Pediatrics, Royal Alexandra Children’s Hospital, Brighton and Sussex Medical School, Brighton, United Kingdom; ^2^Department of Medical Sociology, Institute of Epidemiology and Preventive Medicine, University of Regensburg, Dr.-Gessler-Str. 17, 93051, Regensburg, Germany; ^3^Department of Dermatology, The Warren Alpert Medical School of Brown University, Providence, RI, USA; ^4^Norwich Medical School, University of East Anglia, Norwich, United Kingdom; ^5^Department of Public Health and Primary Care, Brighton and Sussex Medical School, Falmer, United Kingdom

**Correspondence:** Christina J. Jones - c.jones@bsms.ac.uk

*Clinical and Translational Allergy* 2018, **8(Suppl 2):**P54

**Introduction**

Atopic eczema (AE) does not only have an often detrimental impact on affected children, but also on their family and caregivers. It is therefore of interest to measure the quality of life (QoL) impact on caregivers of children with eczema in clinical trials. The aim of this systematic review was to investigate the measurement properties of existing measurement instruments measuring the QoL of caregivers of children with AE.

**Methods**

We systematically searched the literature for studies on measurement instruments developed and/or validated for the measurement of QoL in caregivers of children/adolescents with AE. For the studies included, we assessed both the adequacy of investigated measurement properties as well as the methodological study quality using the COnsensus-based Standards for the selection of health Measurement INstruments (COSMIN) checklist. Results from different studies were summarized in a best-evidence synthesis for each measurement property of each instrument and formed the basis to assign four degrees of recommendation.

**Results**

Sixteen studies were included reporting on 20 instruments used to measure QoL in caregivers of children with AE: the Parents’ Index of Quality of Life in Atopic Dermatitis (PIQOL-AD) in seven languages, the Dermatitis Family Impact (DFI) in six languages, the Childhood Atopic Dermatitis Impact Scale (CADIS) in four languages, the Family Dermatology Life Quality Index (FDLQI) in Ukrainian, the Quality of Life in Primary Caregivers of Children with Atopic Dermatitis (QPCAD) in Japanese and a German questionnaire by Rueden et al. We found substantial validation gaps. For instance, none of the studies investigated measurement error. Overall, no instrument can be recommended for measuring QoL in caregivers of children with AE because none fulfilled all required adequacy criteria. With adequate internal consistency and reliability, the US version of the Childhood Atopic Dermatitis Impact Scale (CADIS) has the potential to be recommended depending on the results of future validation studies.

**Conclusion**

Currently, no instruments used to measure caregivers of children with AE can be highly recommended. Further studies filling validation gaps are needed.

## P55

### Der p 23—molecular components and allergic respiratory disease expression in children

#### Filipa Matos Semedo^1^*, Tomaz Elza^2^, Ana Paula Pires^2^, Filipe Inácio^2^

##### ^1^Immunoallergology Department, Hospital de São Bernardo, CH Setúbal; Faculdade Ciȇncia, Setúbal, Portugal; ^2^Immunoallergology Department, Hospital de São Bernardo, CH Setúbal, Setúbal, Portugal

**Correspondence:** Filipa Matos Semedo - pipa.semedo@gmail.com

*Clinical and Translational Allergy* 2018, **8(Suppl 2):**P55

**Introduction**

House dust mites (HDM) represent one of the most important inducers of respiratory allergies worldwide, with up to 85% of allergic asthmatic children being sensitized. Recently, a new major HDM allergen- Der p 23, was identified. It’s recognized by > 70% of HDM-allergic patients, exhibiting high allergenic activity. We sought to investigate a pediatric population of atopic patients with allergy to *Dermatophagoides pteronyssinus* concerning molecular major allergen profile, assessing the relevance of Der p 23 sensitization.

**Methods**

Retrospective study, including 81 children with allergic rhinitis and/or asthma and reactivity to *Dermatophagoides pteronyssinus* assessed by total immunoglobulin E (IgE) and major specific IgE allergens Der p 1, Der p 2. Serum samples were tested for IgE against Der p 23 and patients were analysed regarding allergic features.

**Results**

This study included 81 patients (70% males; mean age of 11.7 years), 89% with allergic asthma and 11% with allergic rhinitis only. Concerning prevalence of IgE reactivity, 90% of patients were sensitized to Der p 23. Mean IgE levels (kUA/l) in asthmatic versus rhinitic patients were, respectively: total Der p: 112.2 vs. 83.1; Der p 1: 58.7 vs. 35.2; Der p 2: 58.6 vs. 12.8; Der p 23: 22.3 vs. 12.5. Difference had statistical significance regarding Der p 23 (T test, p = 0.03). In group of asthmatic children, 68% began disease symptoms at age of 3 years old, having mean value of IgE to Der p 23 of 19.6 kUA/l. Also in asthmatic patients, 71% had multiple respiratory viral infections, having mean value of IgE to Der p 23 of 19.4 kUA/l. Severity of asthma, evaluated by treatment control, was higher in 32% of patients (high dose of inhaled corticosteroid or in association with long acting beta-agonists), with mean IgE against Der p 23 of 27.8 kUA/l.

**Conclusion**

Few is known about the prevalence of IgE against Der p 23 in pediatric patients. Our data indicated a significantly high rate of reactivity to this new major allergen. Mean value of specific IgE to all major allergens were higher in asthmatic children, comparing to those with only rhinitis, this difference being significant for Der p 23. It has been reported that high IgE antibody titers to HDM allergens increased the risk for severity of disease among asthmatic children. This study showed that asthmatic children with early symptoms, multiple respiratory infections and higher severity of illness presented with high levels of IgE to Der p 23.

## P56

### Severe anaphylaxis to fresh frozen plasma in congenital thrombotic thrombocytopenic purpura

#### Anamarija Čavčić^1^*

##### ^1^Department of Pediatrics, University Hospital Center Zagreb, Zagreb, Croatia

**Correspondence:** Anamarija Čavčić - anacavcic@yahoo.com

*Clinical and Translational Allergy* 2018, **8(Suppl 2):**P56

**Introduction**

Congenital form of thrombotic thrombocytopenic purpura (cTTP) is consequence of decreased ADAMTS13 activity secondary to mutations within the *ADAMTS13* gene [1]. Acute episodes of cTTP are treated with plasma exchange (PEX) or plasma infusion alone in a similar way to acquired TTP while immunosuppression is not indicated [2]. Despite treatment improvements, TTP still has a high mortality rate.

**Case report**

A 5 year-old boy with recurrent episodes of hemolytic anemia, thrombocytopenia and hematuria since early infancy was diagnosed with cTTP (activity level of ADAMTS13 < 5%, without evidence of anti-ADAMTS13 antibodies). The treatment with fresh frozen plasma (FFP) infusions 10 mg/kg every third week has been started. Due to allergic reactions complicating every exposure to plasma, prophylaxis with systemic steroids and antihistamines was used. Despite of prophylactic treatment the patient developed anaphylaxis, with generalised hives, angioedema, dyspnea, bronchospasm, stridor and hypoxemia (SpO2 89–90%). Epinephrine 0.5 mg has been administered immediately, intramuscularly along with supplemental oxygen followed by nebulized salbutamol 2.5 mg every 20 min. Methylprednisolone 1 mg/kg iv; diphenhydramine 1 mg/kg iv. and cetirizine 10 mg orally were the second line medications and recovery was achieved within an hour. Subsequently, FFP has been replaced by solvent/detergent-treated pooled plasma (OCTAPLAS LG). Throughout the 14 month period the evidence confirmed advantage of solvent/detergent-treated pooled plasma over the FFP regarding efficacy and safety.

**Conclusion**

Patients with cTTP have a significant lifetime exposure to plasma and therefore, the safest product of solvent/detergent-treated pooled plasma should be choice of therapy in pediatric patients with anaphylaxis to FFP.

**Consent to publish**

The patient has given written permission for the author to publish the case report.

## P57

### Treatment of anaphylaxis from general practitioners and specialists. Do they know what they are supposed to do?

#### Konstantinos Kakleas^1^*, Sophie Farooque^1^

##### ^1^Allergy Department, St Mary’s Hospital, London, United Kingdom

**Correspondence:** Konstantinos Kakleas - Konstantinos.kakleas@bartshealth.nhs.uk

*Clinical and Translational Allergy* 2018, **8(Suppl 2):**P57

**Introduction**

Anaphylaxis is a severe and potentially life-threatening multisystem allergic reaction. The prevalence of anaphylaxis is 0.3% [1] and the mortality 0.001% [2]. Prompt recognition and treatment by medical professionals is vital. The aim of this study was to assess the knowledge of anaphylaxis management by general practitioners and physicians.

**Methods**

Data were collected from a questionnaire designed by our team and given out prior to educational sessions. Data from 86 medical professionals was collected (General Practitioners (n = 44) and Specialist trainees (n = 42)

**Results**

70% of the participants (59/86) had received postgraduate anaphylaxis training and 63% (54/86) said they were confident in the treatment of anaphylaxis. However, only 10% correctly reported the correct dose, route and concentration of Adrenaline to be administered during an anaphylactic reaction.

In the case of anaphylaxis whilst 83% of respondents would use Adrenaline as first choice drug, 79%of respondents would administer incorrect adult dose, 70% would use an incorrect concentration of Adrenaline and9% would inject adrenaline subcutaneously. Only 30% of the participants answered correctly the adrenaline concentration (1:1000). Furthermore, over 65% of physicians were unaware of the ALS guidelines stating patients should be treated in a supine position with legs raised if their breathing is not impaired.

Doctors also were unclear about when Adrenaline is the drug of choice in anaphylaxis management. If the patient had urticarial rash as their only symptom then 14% of medical staff would give Adrenaline as the first line drug of choice before antihistamines and steroids. However if the urticarial rash was associated with throat tightness, hypotension or wheeze the percentages would increase to 75, 81 and 70% respectively. Only 37% of respondents would administer Adrenaline as a first line agent if wheeze was the only symptom and would preferentially treat with nebulised salbutamol.

**Conclusions**

Hospital physicians and GPs are frequently the first line responders in anaphylaxis. Immediate treatment according to UK resus council guidelines should include adrenaline, oxygen and fluids.

In this cohort only 10% of those questioned knew the correct dose, route and concentration of Adrenaline as per UK resuscitation council guidelines [3]. The majority of medical professionals would not give adrenaline if wheeze was the only symptom although in 20% of individuals with anaphylaxis wheeze is the only presenting feature (4). This knowledge gap suggests improved training and use of clear posters and cognitive aidscould improve the diagnosis and management of anaphylaxis by medical practitioners [5].

**References**

1. Panesar SS, Javad S, de Silva D, Nwaru BI, Hickstein L, Muraro A et al. The epidemiology of anaphylaxis in Europe: a systematic review. Allergy. 2013 Nov;68(11):1353–61
2. Dhami S, Panesar SS, Rader T, Muraro A, Roberts G, Worm M, et al. The acute and long-term management of anaphylaxis: protocol for a systematic review. Clin Transl Allergy. 2013 Apr 10;3(1):14.
3. https://www.resus.org.uk/resuscitation-guidelines/pediatric-advanced-life-support/
4. Sampson HA^1^, Muñoz-Furlong A, Bock SA, Schmitt C, Bass R, Chowdhury BA, Decker WW et al. Symposium on the definition and management of anaphylaxis: summary report. J Allergy Clin Immunol. 2005 Mar;115(3):584–91.
5. Kolawole H, Marshall SD, Crilly H, Kerridge RK, Roessler P ANZAAG/ANZCA Perioperative Anaphylaxis Management Guidelines. Anaesth Intensive Care 2017: 45 (2) 151–8

## P59

### Growth of children with food allergies in Singapore

#### Chong Kok Wee^1^*, Wright Karen^1^, Goh Anne^1^, Meyer Rosan^2^, Rao Rajeshwar^1^

##### ^1^KK Women’s and Children’s Hospital, Singapore, Singapore; ^2^Imperial College, London, United Kingdom

**Correspondence:** Chong Kok Wee - chong.kok.wee@singhealth.com.sg

*Clinical and Translational Allergy* 2018, **8(Suppl 2):**P59

**Introduction**

Although it is known that children with food allergies are at risk of impaired growth, no data has been published from South East Asia on growth related to type of allergy, number of foods eliminated and allergic co-morbidities.

**Methods**

Anthropometric data, including weight and length/height was collected during patient’s routine allergy clinic visit. Demographic data, including type of food allergy (IgE and non-IgE), foods eliminated and atopic co-morbidities were recorded. All data was collected anonymously as part of an international multicentre study. Malnutrition was defined according to World Health Organization standards [≤ -2 Z-score for weight for height (WH), weight for age (WA) and height for age (HA)].

**Results**

Seventy-four patients (51% male) were recruited over 1 month, with a median age at diagnosis of 8 months (IQR: 4–13) and at data collection of 25 months (IQR: 14–48). Sixty-two (84%) had IgE-mediated allergy, 8 (11%) mixed IgE and non IgE and 4 (5%) non IgE-mediated allergy. Food exclusions: 55% one food, 27% two foods, 8% three to four foods and 10% ≥ 5 foods. Only 1% were underweight (WA ≤ -2 Z-score) and 3% had WA ≥+2 Z-score. Excluding more than 1 food significantly reduced WA (p = 0.023). WA was significantly lower for those referred to the dietitian (p = 0.027). 5.6% were stunted (HA ≤ -2 Z-score). Factors significantly associated with stunting were underlying eczema (p = 0.03) and having an IgE-mediated (p = 0.03) or mixed type food allergy (p = 0.002). 1.4% were undernourished (WH ≤ -2 Z-score) and 1.4% were overweight (WH ≥+2 Z-score). Multivariate regression analysis found that children with multiple food allergies were significantly shorter (Z-score -1 lower) when compared to those avoiding only one food. Children had a lower WA if they had skin involvement as part of their symptom presentation.

**Conclusion**

This is the first survey documenting growth in children with food allergy in Singapore. We found that stunting is more common in this cohort and appears to be linked not only to the number of foods excluded, but also to co-existent eczema and type of allergic disease namely, IgE and mixed type allergies. Children referred to the dietitian were significantly smaller, suggesting that this group are in need of earlier dietetic and nutrition advice. The impaired growth in these children is likely due to a combination of factors: the general inflammatory response affecting the absorption and utilisation of nutrients, as well as the elimination diet.

## P60

### Evaluation of the oral food challenge with baked milk and egg in pediatric patients

#### Sirin Kose Seda*, Atakul Gizem, Asilsoy Suna, Uzuner Nevin, Karaman Ozkan, Anal Ozden

##### Dokuz Eylul University Faculty of Medicine Department of Immunology and Allergy, Izmir, Turkey

**Correspondence:** Sirin Kose Seda - sedasirin85@yahoo.com

*Clinical and Translational Allergy* 2018, **8(Suppl 2):**P60

**Introduction**

Food allergy in pediatric age group most commonly occurs with cow’s milk and egg. The oral food challenge (OFC) is considered the “gold standard” for diagnosing food allergy. Recent studies suggest that 70% of patients with cow’s milk and egg allergy can tolerate the baked products. It is also reported that the consumption of these products increases the tolerance to allergens. In this study, we aimed to investigate the clinical, laboratory findings of patients in whom OFC was performed with baked cow’s milk and egg.

**Methods**

This study was performed in Department of Immunology and Allergy of Dokuz Eylul University Hospital between March 2015 and July 2016. Twenty-nine patients, diagnosed as cow’s milk and/or egg allergy, with medical history, clinical findings and laboratory findings were enrolled in the study. In all patients, OFC with baked milk and/or egg was performed according to allergy type.

**Results**

Six patients with egg allergy, 10 patients with cow’s milk allergy, 13 patients with both cow’s milk and egg allergy were evaluated. Eleven (37.9%) patients were female and the mean age of patients was 36.5 ± 24.3 months (2–90). Eleven (37.9%) and 7 (24.1%) patients were admitted with the skin and gastrointestinal findings, respectively. In seven (24.1%) patients, anaphylaxis had occurred due to food allergy. Oral food challenges with baked cow’s milk and egg were performed in 8 (27.6%) and 1 (3.4%) patients, respectively. Also, in 20 (%69) patients, OFC with both baked cow’s milk and egg was performed. Complication of OFC was determined in only one case as vomiting. No adverse reaction was defined in 28 (96%) patients. In patients with cow’s milk allergy, patients with anaphylaxis (n = 5) had higher milk-specific IgE levels compared to patients without anaphylaxis (n = 5) (p = 0.047). After the OFC with baked products, 12 (41.3%) patients became able to consume cow’s milk and egg.

**Conclusion**

In this study, consistent with the literature, almost all patients with cow’s milk and egg allergy were able to well tolerate OFC with baked products. Moreover, nearly half of the patients became able to consume cow’s milk and egg safely, following the OFC with baked milk and egg.

## P61

### The effectiveness of symptom-based score on the diagnosis and follow-up of food allergy

#### Sirin Kose Seda*, Atakul Gizem, Asilsoy Suna, Uzuner Nevin, Karaman Ozkan, Anal Ozden

##### Dokuz Eylul University Faculty of Medicine Department of Immunology and Allergy, Izmir, Turkey

**Correspondence:** Sirin Kose Seda - sedasirin85@yahoo.com

*Clinical and Translational Allergy* 2018, **8(Suppl 2):**P61

**Introduction**

Symptom-based score (SBS) is a scoring system developed based on the symptoms and findings of the patients to predict cow’s milk allergy in primary health care. It can also be used in follow-up of infants with cow’s milk allergy.

Although recent studies were performed to assess the efficiency of SBS on the diagnosis of cow’s milk allergy in infants, there was limited data about the SBS in literature. In this study, we aimed to investigate the effectiveness of SBS on the diagnosis and in monitoring the response to treatment in patients with food allergy.

**Methods**

Between July 2016 and February 2017, we evaluated 93 cases who were diagnosed as food allergy, in pediatric immunology and allergy clinic. Symptom-based scoring form which includes crying, regurgitation, Bristol stool scale, skin symptoms (atopic dermatitis and urticaria score), and respiratory symptoms scores was performed. Demographic and laboratory findings were recorded for all patients before elimination diet treatment. After 8 weeks of treatment, SBS was recalculated.

**Results**

Ninety-three patients were enrolled in the study. Of these, 46 (49.5%) were female and 47 (50.5%) were male. The mean age of the patients was 5.6 ± 0.2 months. Atopy history in family was found in 72% of patients. Cow’s milk and egg allergy were determined in 17 (18.3%) and 23 (24.7%) patients, respectively. Both cow’s milk and egg allergy was determined in 14 (15.1%) patients. IgE-mediated, non-IgE-mediated and mixed immune reactions were revealed, in 26 (28%), 23 (24.7%) and 44 (47.3%) patients, respectively. Before elimination diet, mean SBS was calculated as 12.9–4.7. Eight weeks after the elimination diet, mean SBS decreased to 3.7 ± 2.7. In the analysis of IgE-mediated, non-IgE-mediated and mixed immune reaction groups, urticaria score and SBS score were found to be higher in IgE-mediated group than the other groups (p < 0.0001; p = 0.01). Bristol stool scale was higher in non-IgE-mediated group (p = 0.007).Atopic dermatitis score was found to be higher in mixed immune reaction group (p < 0.0001). Serum IgE level and percentage of eosinophils in complete blood count were higher in IgE-mediated group (p < 0.0001; p = 0.01). Eight weeks after the elimination diet, no difference was determined between the SBS of three groups.

**Conclusion**

The results of this study suggested that SBS can be used in diagnosis and monitoring the response to treatment in infants with food allergy. Moreover, this is the first study performed to date that revealed the effectiveness of SBS in egg allergy besides cow’s milk allergy.

## P62

### Supervised Feed Challenges—A safe and effective method of diagnosing food allergy?

#### Foley Gary, Kakleas Kostas, Ali LuuL, Ling Francis, Noimark Lee

##### Dept. of Paediatric Allergy, The Royal London Hospital, London, UK

**Correspondence:** Foley Gary - ofoghlug@tcd.i.e.

*Clinical and Translational Allergy* 2018, **8(Suppl 2):**P62

**Introduction**

The diagnosis of type one mediated food allergy still rests on the pillars of clinical history, examination and investigation - with clinical history holding a lot of weight. The investigations of choice when needed are skin prick testing (SPT), antigen specific immunoglobulin E (IgE) blood investigations and oral food challenges. Oral food challenges provide a real time experience of whether a food allergy exists or not. These can be quite time consuming, and when in doubt, waiting times can delay a confirmatory diagnosis. In recent years a new format of oral food challenge- the supervised feed has emerged. This has now been introduced in many hospitals to try and expedite food challenges for those who meet strict criteria.

**Methods**

A retrospective audit was performed in the Royal London Hospital using the oral food challenge computerised database. 54 supervised feeds to three common allergy associated tree nuts from the previous 12 months were analysed; hazelnut (n = 21), cashew nut (n = 17) and almond (n = 16). The criteria for a supervised feed to occur were: 1. No previous known serious allergic reactions, 2.SPT 0–2 mm and/or 3. Specific IgE < 0.1. Tolerance verses food allergy confirmation and severity of allergic reaction were the main outcomes measured.

**Results**

Of the supervised feeds, 48 children were tolerant of the food showing no immediate reaction, 5 (almond n = 3 and hazelnut n = 2) were shown to have immediate allergic reactions. Nearly half (n = 25) were supervised feeds were more than one tree nut was given (e.g. hazelnut + almond).Of this group 1 child suffered a mild allergic reaction and was subsequently booked into conduct separate food challenges on the tree nuts in question. No children required intramuscular adrenaline or respiratory support, and all allergic reactions (n = 5) were treated with second-generation antihistamines (cetirizine).

**Conclusion**

Our results show that supervised feeds, using specific criteria provide a safe and practical means of diagnosing or ruling out a specific food allergy. This can lead to a greater amount of challenges being performed earlier in the disease course giving an earlier indication to the extent of the food allergy. Given the results it may be possible to alter the supervised feed criteria, but further evaluation will be needed

## P63

### Two episodes of anaphylaxis by intake of Rosaceae fruits: cherry and apple in a Mediterranean country

#### Angela Llaneza*, Jose María Maillo del Castillo

##### Complejo Asistencial de Ávila, Ávila, Spain

**Correspondence:** Angela Llaneza - allanezamartin@gmail.com

*Clinical and Translational Allergy* 2018, **8(Suppl 2):**P63

**Introduction**

Allergic food reactions are the most common cause of anaphylaxis outside the Hospital setting. Fruits of the *rosaceae* family are the vegetable foods that most frequently produce allergic reactions. In the Mediterranean countries, allergic reactions following ingestion of *rosaceae* are more severe and are not preceded by sensitization to birch pollen, but to lipid transport proteins (LTP).

**Case report**

9-year-old boy shows anaphylaxis episode (urticaria, facial angioedema, hand edema and respiratory distress) while playing in an area of rose bushes, in May, and 3 h after having eaten pork loin with cheese and a cherry “that did not feel well”. In Primary care, methylprednisolone and intravenous dexchlorpheniramine were administered, with remission of symptoms. After being studied, it had been recommended to avoid *rosaceae* fruit intake and autoinjective adrenaline had been indicated, nevertheless a second event happened. Two years later, in April, he presented a new episode of anaphylaxis (pruritus and heat in the face and thorax, eyelids and lips edema, swelling of hands and respiratory distress) immediately after eating half a red apple, also transferred to the health centre where same treatment was given with slower resolution of symptoms.

Since childhood, after contact with peach and paraguayan skin, edema and erythema in contact zone appears. With spoonful of yogurt peach flavour also refers to lip edema. No rhinoconjunctival symptoms in spring, no bronchospasm.

Prick skin tests positive for grass and olive pollen, peach skin, cherry, apple, almond and LTP. IgE specific: peach class 3, pru p3 9.78 kU/l moderate level, pru p1 PR-10 not detected, cherry 7 kU/l moderate level, olive class 1, dactylis glomerata class 4, phleum pratense class 4, cynodon dactylon class 2.

**Conclusion**

We describe double severe episodes of anaphylaxis due to ingestion of *rosaceae* fruits in the time of grasses pollination as adjuvant factor.

In Spain, apple and cherry allergy is more frequently severe (> 35% systemic reactions). There is primary sensitization to other fruits of the family, such as peach, with sensitization to LTP that indicates possibility of systemic and severe reaction.

After the study, hypersensitivity to grass pollen is shown as usual as part of pollen-fruit syndrome, with *rosaceae*.

It is important to continue teaching health professionals and patients themselves on the correct treatment of an anaphylaxis event. Although those were satisfactorily resolved, the first line of treatment recommended in clinical guidelines: adrenaline IM, was not employed.

**Consent to publish**

The authors have obtained parental informed consent of the patient mentioned in the article.

## P64

### Silkworm pupa anaphylaxis

#### Charoenying Yingwan*, Chansakulporn Somboon

##### Division of Allergy and Immunology, Department of Pediatric, Faculty of Medicine, Srinakarinwirot U, Bangkok, Thailand

**Correspondence:** Charoenying Yingwan - yingwan1doc@yahoo.com

*Clinical and Translational Allergy* 2018, **8(Suppl 2):**P64

**Introduction**

Silkworm pupae (*Bombyx mori*) are the important source of protein and other nutrients delicacies for Asian and Thai people. Anaphylactic reactions after silkworm pupa ingestion have rarely been described in the medical literature.

**Case report**

We reported a 6-year-old, non-atopic female suffering an episode of anaphylaxis including generalized urticaria, crampy abdominal pain with vomiting and wheezing after ingestion of fried silkworm pupae for 30 min with no symptoms in other family members who ate the same. The diagnostic test was performed to confirm IgE-mediated reaction with skin prick test for silkworm pupa and the result was positive.

**Conclusion**

Silkworm pupae are the newly recognized food as the trigger of anaphylaxis. Healthcare professionals and consumers should be aware of life-threatening reactions from this food source. Major allergens of these silkworm pupae and risk of anaphylaxis after ingestion might need to be further explored.

**Consent to publish**

The parent of this patient has given her consent for her child‘s information for presentation and publication.

## P65

### Evaluation of knowlwdge about anaphylaxis among medicine students and implementation of a practical training program

#### Aída Del Campo García, Sara Pereiro Fernández*, Alicia Costas Aguado, Fernando Bandrés Sánchez-Cruz, Jose Ramón Fernández Lorenzo

##### Hospital Álvaro Cunqueiro, Vigo, Spain

**Correspondence:** Sara Pereiro Fernández - palas89@hotmail.com

*Clinical and Translational Allergy* 2018, **8(Suppl 2):**P65

**Introduction**

The increase of food allergies in children during the last decades has raised the anaphylaxis cases number. For this reason, it is absolutely necessary that any doctor knows how to identify and how to treat anaphylaxis correctly. Therefore, it should be part of the academic university health education.

**Methods**

We performed two methodological approaches. The first one was a descriptive study of the knowledge about anaphylaxis in a series of medical students using a questionnaire. The second one was an quasi-experimental analytical study in which we compared the previous results with those obtained after organized and carried out an anaphylaxis workshop. The workshop consisted in 45 min of a theoretical and practical training.

**Results**

We included 315 survey respondents (66% women) who belonged to 3°, 4°, 5° and 6° years of medicine. The successful answers number in the test which was carried out before the workshop was bigger among the two higher courses. The difference from the other courses was statistically significant. Before attending the workshop, 21.6% of the total recognize the anaphylaxis symptoms, rising to 81.9% later. 32.7% of them considered adrenaline as a treatment which you can choose or no, rising up to 94.2% afterwards. This represents a statistically significant change.

Regarding the right use of auto-injectors, it was well identified by the 44.4% of the students before attending the workshop. This percentage turns into 87.6% after the workshop. In relation to the questions posed as an anaphylaxis clinical case in pediatrics and before attending the workshop, 38.4% of the total would diagnose anaphylaxis and a percentage of 34.9 would act correctly. After attending the workshop, 93.6% would diagnose this allergic reaction and 69.5% would act properly, which represents a statistically significant change. 64% of the students rated negatively the received training about anaphylaxis in the university curriculum. 77% of the students valued the training positively.

**Conclusion**

The development of a theoretical and practical workshop on anaphylaxis in children and on the management of auto-injectable adrenaline within the university education in medicine increases the knowledge of future doctors and enables them to perform correctly in our sample. The success of our study suggests the need to include practical training in anaphylaxis in the medicine university curriculum.

## P66

### Diagnosis and management of cow’s milk protein allergy—How big is the gap between ideal and reality? A quality-of-care survey in Europe

#### Katharina Werkstetter^1^, Ania Chmielewska^2^, Jernej Dolinšek^3^, Frederiek Estourgie-van Burk^4^, Ilma Korponay-Szabó^5^, Kalle Kurppa^6^, ZrinjkaMišak^7^, Alexandra Papadopoulou^8^, Alina Popp^9^, Carmen RibesKoninckx^10^, Boglárka Szentes^1^, Peter Szitanyi^11^, Anna Theisen^1^, Riccardo Troncone^12^, Gabor Veres^13^, Christina West^2^, Sibylle Koletzko^1^*

##### ^1^Ludwig Maximilian’s University Munich Medical Center, Dr. von Hauner Children’s Hospital, Munich, Germany; ^2^Department of Clinical Sciences, Umeå University Hospital, Umeå, Sweden; ^3^University Medical Centre (UMC) Maribor, Maribor, Slovenia; ^4^Department of Pediatrics, Academic Medical Centre (AMC) Amsterdam, the Netherlands; ^5^Celiac Disease Centre, Heim Pál Children’s Hospital, Budapest, Hungary; ^6^Tampere Centre for Child Health Research, University of Tampere and Tampere University Hospital, Tampere, Finland; ^7^Referral Centre for Pediatric Gastroenterology and Nutrition, Children’s Hospital Zagreb, Zagreb, Croatia; ^8^Gastroenterology, Hepatology and Nutrition Unit, First Department of Pediatrics, “Aghia Sofia” Children’s Hospital, Athens, Greece; ^9^“Carol Davila” University of Medicine and Pharmacy, Bucharest, Romania; ^10^Department of Pediatric Gastroenterology and Hepatology, La Fe University Hospital, Valencia, Spain; ^11^Department of Pediatrics, First Faculty of Medicine, Charles University and General University Hospital, Prague, Czech Republic; ^12^Department of Translational Medical Science and European Laboratory for Food-Induced Disease, University Federico II, Naples, Italy; ^13^First Department of Pediatrics, Semmelweiss University, Budapest, Hungary

**Correspondence:** Sibylle Koletzko - sibylle.koletzko@med.uni-muenchen.de

*Clinical and Translational Allergy* 2018, **8(Suppl 2):**P66

**Introduction**

In 2012 the ESPGHAN published guidance for diagnosis and management of cow’s milk protein allergy (CMPA) [1]. We conducted a quality-of-care survey across Europe to evaluate the implementation in primary care practice.

**Methods**

From 2/2015 to 12/2016, an anonymous online-survey was sent to pediatricians and/or general practitioners in 13 countries (Croatia, Czech Republic, Finland, Germany, Greece, Hungary, Italy, Poland, Romania, Slovenia, Spain, Sweden and the Netherlands). Participants were invited via email by their respective medical association. The survey included demographic questions and medical case-examples with multiple-choice answers regarding CMPA management.

**Results**

In total 2551 physicians completed the survey (72% female, 86.8% pediatricians). Being asked how to exclude CMPA in a 10-month old infant with chronic diarrhoea and failure to thrive, 68% correctly chose an elimination diet and challenge procedure in case symptoms improve. However, 19% regarded a negative specific IgE result and 8% a negative skin prick-test as sufficient to exclude CMPA, while 5% would eliminate lactose. The question which other formulas are allowed for an infant diagnosed with CMPA, but refusing extensively hydrolysed formula, was correctly answered by 63% with amino acid-based and 51% soy-based formula, but 19% considered partially hydrolysed, 11% goat’s milk-based and 6% lactose-free cow’s milk-based formula as adequate. The question what to advise in a so far exclusively breast-fed 5-month-old infant developing swelling of lips and eyelids on drinking his 2nd bottle of infant formula, was correctly answered by 26% to resume complete breastfeeding under usual diet of the mother, while 46% would advise breast-feeding under maternal elimination of dairy products, 21% would switch to an extensively hydrolysed and 6% to an amino-acid-based formula. Being asked what to advise for the same child in terms of complementary foods (CF), 53% would start but strictly avoid CMP, while 15% would also eliminate other potent allergens until 12 months, 25% would recommend CF after 6 months and 5% would start without any restrictions. When having tested this child negative for specific IgE, 46% would still perform supervised CMP challenge, 36% would continue elimination diet until 12 months, 7% would consider CMPA as unlikely, 6% would test C1-esterase-inhibitor-deficiency and 5% for IgG against CMP.

**Conclusions**

Our results disclose major deficits in the management of CMPA, particularly how to test, when to perform elimination diet and what types of infant formulas to use. Appropriate dissemination and training activities in primary health care settings are needed.

**Reference**

1. Koletzko S, Niggemann B, Arato A, et al. Diagnostic Approach and Management of Cow’s-Milk Protein Allergy in Infants and Children: ESPGHAN GI Committee Practical Guidelines. JPGN 2012;55:221–229

## P67

### Legume sensitisation, allergic reaction and treatment in an East London population

#### Anna Burford, Garry Foley, Luul Ali, Frances Ling, Rozalynd Gourgey, Konstantinos Kakleas*, Lee Noimark

##### Pediatric Allergy Department, Royal London Hospital, London, United Kingdom

**Correspondence:** Konstantinos Kakleas - koskakl2@yahoo.gr

*Clinical and Translational Allergy* 2018, **8(Suppl 2):**P67

**Introduction**

Legumes are recommended by health organisations as staple food due to their low cost, high protein, lipid and vitamin content [1]. The widespread use of legumes has resulted in an increased prevalence of allergy. There is also significant cross-reactivity between different legumes due to the presence of structurally homologous proteins [2]. The aim of this study was to assess sensitisation and clinical reaction to various legumes.

**Methods**

Data was retrospectively collected from electronic records for children diagnosed with legume allergy (lentils, beans, chickpea, green pea). 32 patients were identified. Carers/patients completed a questionnaire. Skin prick testing was undertaken for 13 different legumes.

**Results**

Mean age of first reaction was 1.78 years (SD ± 1.7 years). Red lentil was reported as the most common allergenic legume (37.5%, 12/32) followed by chickpea (9.4%) and 9.4% reacted to both chickpea and red lentil. Half of patients with legume reactions reported involvement of respiratory system (50%, 16/32), however only one patient had been treated with adrenaline (3.1%). One in four tolerated chickpeas, 15% haricot beans, 9.1% green pea and 6% red lentils. Children who tolerated chickpea had skin prick tests (SPT) between 0 and 7 mm. Patients who tolerated green pea and lentil had SPT 0–3 mm and all patients who tolerated haricot bean had 0 mm. 9.3% reacted to multiple pulses. 66% of patients who reacted to red lentil tolerated chickpea and beans, whereas 33% of patients with chickpea allergy tolerated red lentils.

**Conclusion**

Legume allergy presents in the first 3 years of life. Red lentils and chickpeas are the commonest allergens. 10% of these East London patients had reactions to multiple legumes. The majority of patients with red lentil allergy tolerated chickpea and beans, but only one-third of patients with chickpea allergy tolerated red lentil. Management of anaphylaxis was suboptimal.

**References**

1. Duranti M. Grain legume proteins and nutraceutical properties. Fitoterapia. 2006 Feb;77(2):67–82.
2. Verma AK, Kumar S, Das M, Dwivedi PD. A comprehensive review of legume allergy. Clin Rev Allergy Immunol. 2013 Aug;45(1):30–46

## P68

### A thematic analysis of coping in adolescents aged 12–16 years old with food allergy

#### Jennifer Hammond*, Richard Cooke, Rebecca Knibb

Aston University, Birmingham, United Kingdom

**Correspondence:** Jennifer Hammond - hammojl2@aston.ac.uk

*Clinical and Translational Allergy* 2018, **8(Suppl 2):**P68

**Introduction**

The prevalence of food allergy appears to be increasing, and the highest proportion of fatalities have been linked to this population. However, research in this age group specific to coping is limited.

**Methods**

Participants were recruited via allergy clinics in Birmingham, or via advertisements by Allergy UK. Individual interviews were conducted with nineteen adolescents (face to face or over Skype) between the ages of 12 and 16 years old, recorded and then transcribed verbatim. Data was analysed using thematic analysis, following the guidelines by Braun and Clarke (2006).

**Results**

The thematic analysis produced four key themes that explore how adolescents cope with their food allergy. (1) calculating and managing risk, (2) growing up with food allergy, (3) facilitating and using social support, (4) emotional experience of food allergy. These themes highlight the complexities involved with coping with a food allergy, particularly as different environments and experiences can influence certain coping strategies, and evaluation of risk. Risk-taking can occur if an adolescent assesses their risk to be low when it’s not. However, many have a strategy in place to ensure their safety, and avoid allergic reactions. Growing up with a food allergy can be challenging in terms of the transition of responsibility from the parent to the adolescent, to changes in their social experience. However, duration living with a food allergy can be beneficial with regards to the development and use of coping strategies. Social support remains important, and although many facilitate support by teaching friends and peers about their food allergy, there is still a heavy reliance on parents, particularly for emotional support. The adolescents interviewed also discussed the emotional experience of living with a food allergy, and how they cope with this.

**Conclusion**

This study has given us the opportunity to further explore how adolescents between the ages of 12–16 cope with a food allergy. There is a difference in how this age group calculate risk dependent on their experience or environment which can influence their risk-taking behaviour. It has demonstrated how coping strategies are affected by a change in responsibility, and how they adapt to this, particularly as they begin to socialise more independently with their friends. There is still a dependence on parent’s emotional social support, particularly around feelings of anxiety and sadness around their food allergy. These findings help us to add to the existing literature in this field, which is limited in this age group.

## P69

### Date palm pollen: a trigger of anaphylaxis and allergic rhinitis in the same patient

#### Shendi Hiba*

##### Tawam Hospital, Alain, United Arab Emirates

**Correspondence:** Shendi Hiba - hshendi@seha.ae

*Clinical and Translational Allergy* 2018, **8(Suppl 2):**P69

**Introduction**

Allergic rhinitis is common in the Middle East, affecting 10% of individuals aged 4 years or older [1]. In the United Arab Emirates (UAE), common triggers are dust, grass/tree pollen and animal dander [2, 3].

Date palm pollen is significant trigger in areas where date palm trees are grown; including the Middle East, Mediterranean and North Africa [4–6].

Dates are a sweet fruit commonly eaten in the Middle East and neighbouring countries. Allergy to date fruit has been described [7].

The oral administration of date palm pollen has been used to improve male fertility [8] and as a suspension to reduce mouth ulcers and pain in cancer patients [9]. In some areas of the UAE, the pollen is occasionally incorporated into a cooked dish, or eaten as a light snack directly from the palm tree itself.

**Case report**

This case describes an 11 year old Emirati boy, who presented with generalised urticaria, facial swelling and shortness of breath immediately following ingestion of fresh date palm pollen directly from the tree. He was taken to Hospital where he was noted to have generalized urticaria, facial angioedema, respiratory distress and wheezing.

He has a 5 year history of intermittent, allergic rhinitis and conjunctivitis symptoms that tend to worsen on exposure to palm trees. They do not have pets at home and he is able to tolerate a varied diet including date fruit, without adverse effects.

Specific IgE testing confirmed sensitization to date palm pollen.

The results of specific IgE tests performed on Phadia 250 are shown in Table 1.

**Table 1:** Results of specific IgE tests

Allergen	Specific IgE level reference (0–0.34)
Bahia grass	0.03KUA/L
Bermuda grass	0.02KUA/L
Cat dander epithelium	5.94KUA/L
Cockroach	0.05KUA/L
Date	0.04KUA/L
Date palm pollen	33.3KUA/L
Dog dander	1.10KUA/L
House dust mite panel	0.14KUA/L
Goat epithelium	0.22KUA/L

**Conclusion**

A case of anaphylaxis following the ingestion of date palm tree pollen in a patient with allergic rhinitis is described.

Anaphylaxis induced by the ingestion of bee-pollen has been reported [10]; interestingly, this may be the first report of anaphylaxis induced by the oral consumption of date pollen.For individuals with allergic rhinitis triggered by date palm pollen, avoidance of the ingestion of date palm pollen is advised.

**Consent to publish**

Written consent for presentation and publication has been obtained from the patient’s parent.

**References**

1. Abdulrahman H, Hadi U, Tarraf H, Gharagozlou M, Kamel M, et al. Nasal allergies in the Middle Eastern population: results from the “Allergies in Middle East Survey”. Am J Rhinol Allergy 2012; 26:S3–23.
2. Mahboub B, Al-Hammadi S, Prakash VP, Sulaiman N, Blaiss M, et al. Prevalence and triggers of allergic rhinitis in the United Arab Emirates. WAO Journal 2014; 7:19.
3. Alsowaidi S, Abdulle A, Shehab A, Zuberbier T, Bernsen R. Allergic rhinitis; prevalence and possible risk factors in the Gulf Arab population. Allergy 2010; 65:208–12.
4. Hasnain SM, Al-Frayh AR, Subiza JL, Fernandez-Caldas E, Casanovas M, et al. Sensitization to indigenous pollen and moulds and other outdoor allergens in allergic patients from Saudi Arabia, United Arab Emirates, and Sudan. WAO Journal 2012; 5:59–65.
5. Huertas AJ, Lopez-Saez MP, Carnes J. Clinical profile of a Mediterranean population sensitised to date palm pollen (*Phoenix dactylifera*). A retrospective study. Allergol Immunopathol (Madr) 2011:39–145–9.
6. Serhane H, Amro L, Sajiai H, Yazidi AA. Prevalence of skin sensitization to pollen of date palm in Marrakesh, Morocco. J Allergy (Cairo) 2017. Epub 2017 Feb 8.
7. Kwaasi AA, Harfi HA, Parhar RS, Al-Sedairy ST, Collison KS, et al. Allergy to date fruits: characterization of antigens and allergens of fruits of the date palm (Phoenix dactylifera L.). Allergy 1999; 54:1270–7.
8. Rasekh A, Jashni HK, Rahmanian K, Jahromi AS. Effect of palm pollen on sperm parameters of infertile men. Pak J Biol Sci 2015; 18; 196–9.
9. Elkerm Y, Tawashi R, Date palm pollen as a preventative intervention in radiation and chemotherapy-induced oral mucositis: a pilot study. Integr Cancer Ther 2014; 13:468–72.
10. Choi J, Jang Y, Kim C, Hyun I. Bee pollen-induced anaphylaxis: a case report and literature review. Allergy Asthma Immunol Res 2015; 7:513–517.

## P70

### How reliable is a patient history?—Symptom recall over time in young people and their parents

#### Nandinee Patel*, Goncalo Abrantes, Sarah Lindsley, Joan Bartra, Marta Vazquez-Ortiz, Paul J. Turner

##### Section of Pediatrics, Imperial College London, London, United Kingdom

**Correspondence:** Nandinee Patel - nandinee.patel@imperial.ac.uk

*Clinical and Translational Allergy* 2018, **8(Suppl 2):**P70

**Introduction**

An accurate clinical history is essential in the risk assessment of food-allergic patients, but patients are often seen many months after a food-induced allergic reaction. We assessed patient and parent recall of symptoms occurring at an observed food challenge, up to 1 year later.

**Method**

A brief, standardized interview was conducted at set time points up to a year following completion of a double-blind, placebo-controlled food challenge (DBPCFC) to peanut. The interview assessed recall and perception of reaction symptoms. Parents and patients were interviewed separately, immediately after challenge and then at 3–6 weeks, 6 and 12 months later.Data was analysed for differences over time, and concordance between parent and patient report. The study was approved by the NHS Health Research Authority and informed consent/patient assent was obtained.

**Results**

Fifty-four young people (age 8–17 years, 54% boys) and their parents participated; 15 (28%) experienced anaphylaxis. Immediately following a reaction, both the participants and their parent recall approximately 50% of symptoms correctly, which is improved with specific prompting from the healthcare professional enquiring about organ-specific symptoms. Anaphylaxis was correctly identified in most cases, but this is potentially confounded by the automatic use of parenteral adrenaline to treat anaphylaxis in the study. However, recall worsens over time, with over half of young people no longer giving a history of anaphylaxis after 6 + months, despite prompting by the healthcare professional.

**Conclusions**

Symptom recall worsens over time, but may be unreliable even hours after a reaction, with most patients and their parents under-reporting symptoms. Importantly, many anaphylactic reactions may not be classified as such by a healthcare professional taking a history months later due to this phenomenon. It is important to document reaction symptoms at the earliest opportunity in order to facilitate patient assessment and management strategies.

## P71

### Conversation groups for adolescents with severe food allergy at the Allergy Clinic, Gentofte University Hospital—a pilot project

#### ^1^Pernille Allesen-Holm, ^1^Astrid Frostholm Møller, ^1^Majbrit Høite Hansen*, ^2^Kirsten Skamstrup Hansen

##### ^1^Allergy Clinic, Gentofte Hospital, Gentofte, Denmark; ^2^Allergy Clinic and Department of Pediatrics, Gentofte and Herlev Hospital, Gentofte, Denmark

**Correspondence:** Majbrit Høite Hansen - majbrit_hh1@hotmail.com

*Clinical and Translational Allergy* 2018, **8(Suppl 2):**P71

**Introduction**

The primary goal was to establish a conversation group where adolescents could meet equals and discuss challenges and experiences when dealing with severe food allergy. Secondarily with in-put from the adolescents, we wanted to improve the Clinics offer to these adolescents and this specific patient group.

**Method**

The conversation groups were scheduled as afternoon sessions of 1½ h. We aimed at 5–8 participants in the age 12–20 years.

Two nurses mediated the session—the conversation took place in a relaxed atmosphere at the Allergy Clinic, i.e. in surroundings known to the participants. The conversation was free, but cardboard cards with predefined topics were used as a guide.

At the end of each session, the participants were asked to fill out a questionnaire about their personal out-come of the conversation.

**Results**

In all, 5 sessions with different participants have been conducted since spring 2014.

We have experienced that the adolescents, when they meet peers in this set-up, take the opportunity to talk about the impact of the disease on their daily life, and that they exchange coping strategies regarding the life with severe food allergy.

In the questionnaire all participants responded that it had been a positive experience and that they would like to participate again.

**Conclusion**

Based on the results of the present study, we have implemented conversation groups as an offer to adolescents with severe food allergy two times a year.

Further, we have initiated a new project regarding the transition and education of the adolescent patients with severe allergy.

## P74

### Allergy to *Limanda aspera* (yellowfin sole)

#### Marta Viñas^1^*, Fernando Pineda^2^, Adriana Izquierdo-Dominguez^1^, Miriam Castillo^2^, Maria Jose Castillo^1^, Judit Barrena^1^, Belen Delavalle^1^, Marcel Ibero^1^, Nora Hernandez^1^

##### ^1^Hospital de Terrassa, Terrassa Barcelona, Barcelona, Spain; ^2^Laboratorio Diater, Madrid, Spain

**Correspondence:** Marta Viñas - martavinas@hotmail.es

*Clinical and Translational Allergy* 2018, **8(Suppl 2):**P74

**Introduction**

We report the case of a 2-year-old boy who after eating or touching *Limanda aspera* (a kind or white fish) in the nursery, immediately presented facial urticaria. He tolerates the rest of fishes like sole, cod, monkfish, hake or tuna. Personal history of atopy: allergic to nuts presenting acute urticarial with hazelnuts and almonds. The yellowfin sole, *Limanda aspera*, is a flatfish of the Pleuronectidae family that lives on soft, seafloor at depths of up to 700 metres. Its natural habitat is the temperate waters of the Northern Pacific.

**Method**

Prick test with different, nuts, fishes and *Anisakis simplex* were performed. Total and specific IgE were determined for prick test positive extracts and fish. Specific allergy study included SDS-PAGE with Western Blot to *Limanda aspera* extract.

**Results**

Prick tests were positive for all nuts and negative for all fish and *Anisakis*. Prick by prick for cooked *Limanda* was negative, but positive for raw *Limanda*. Total IgE: 761.6 U/mL. IgE specific to rGad c 1: 0.27, sole: 0.25; almond: 4.27; hazelnut: 26.9; peanut: 11.9; pistachio: 41.2, chestnut: 9.24, pine nut: 1.71, rAra h 2 < 0.1, rAra h 9: 10.1, rJug r 3: 12.9, rJug r 1: 27.2 kU/L. Western Blot recognised two proteins of 35 and 37 kDa in the extract of *Limanda aspera* and no band for tuna, cod, sole, *Anisakis simplex*, or hake.

**Conclusions**

We report a case of food allergy to *Limanda aspera* with good tolerance to the rest of fish species. Western Blot recognised two proteins with molecular weights of 35 and 37 kDa for *Limanda aspera.*

**Consent to publish**

The child’s mother has signed a written informed consent for presentation and publication of this case report.

## P76

### Food protein-induced enterocolitis to cephalopoda in adults

#### Purificacion Gonzalez-Delgado^1^*, Victoria Moreno^2^, Teodoriked Jimenez^1^, Begoña Cueva^1^, Mariela Lindo^1^, Javier Fernandez^1,2^

##### ^1^Allergy Service, General Universitary Hospital, Alicante, Spain; ^2^Medicine Department, Universidad Miguel Hernandez, Elche, Spain

**Correspondence:** Purificacion Gonzalez-Delgado - gonzalez_pur@gva.es

*Clinical and Translational Allergy* 2018, **8(Suppl 2):**P76

**Introduction**

In the last years an increased interest on non-IgE mediated food allergy has been observed, especially on food protein-induced enterocolitis (FPIES), an entity that is usually described in the pediatric population. Although FPIES is accepted that can occur at any age, the number of reports is very scarce in adults.

**Methods**

We report the clinical features of five adults with exclusively gastrointestinal symptoms after ingestion of cephalopoda (squids, octopus, cuttlefish) that were studied in our Allergy section.

Skin tests with cephalopoda, specific IgE and oral food challenge (OFC) were carried out to confirm diagnosis or to know tolerance. A complete blood count with differential was obtained before the OFC and 6 h later if the challege was positive.

**Results**

All patients related abdominal pain, vomiting and three of them, in addition also had diarrhea. Four patients were female and one male, median age at diagnosis was 38.6 years (range 21–46). The median time of symptoms onset after ingestion was 63 min (range 30–120). Resolution of symptoms occurred between 4 and 48 h spontaneously

The diagnosis was established after a median of 7 previous reactions (5–10).

Skin tests with cephalopoda were negative in all the patients. Specific IgE to squid and octopus was undetectable.

OFC was performed in three patientsbeing positive, two patients refused OFC because repetitive or severe reactions. A median increase of 1100 leucocyte was detected after positive OFC.

**Conclusion**

We report five adults with symptoms of FPIES after cephalopoda ingestion. Abdominal pain and repetitive vomiting were the predominat features.

Although patients present repetitive episodes, the negative skin tests andthe lack of urticaria or respiratory distress produces a delay in diagnosis. Otherwise the entity is probably underdiagnosed due to the patients avoid ingestion of the causative food and do not seek medical attention in most cases.

## P77

### Multiple food allergies in childhood is associated with the persistence of food allergy into adolescence

#### Rebecca McCarthy^1^*, Genevieve Southgate^1^, Zaraquiza Zolkipli^2^, Nicola J. Graham^1^, Gabrielle A. Lockett^1^, Yvonne Tan^1^, Emma Grainger-Allen^2^, Devasmitha Venkataraman^3^, Eleanor Minshall^4^, John W. Holloway^1^, Mich Erlewyn-Lajeunesse^1,2^

##### ^1^Faculty of Medicine, University of Southampton, Southampton, United Kingdom; ^2^The Allergy Clinic, Southampton Children’s Hospital, Southampton, United Kingdom; ^3^South Tees Hospital NHS Foundation Trust, James Cook University Hospital, Middlesbrough, United Kingdom; ^4^Department of Pediatric Allergy, Cambridge University NHS Hospitals Foundation Trust, Cambridge CB2 0QQ, United Kingdom

**Correspondence:** Rebecca McCarthy - rm2g14@soton.ac.uk

*Clinical and Translational Allergy* 2018, **8(Suppl 2):**P77

**Introduction**

Food allergy (FA) in childhood is a dynamic process. Some children develop secondary tolerance having previously been allergic, whereas others have allergic sensitization that persists into adulthood. Transient FAs typically involve milk, soy, wheat or egg allergens, whilst persistent FAs are associated with peanut, tree nut, fish and shellfish. It is unclear why certain allergens provoke a persistent immune response whilst others permit the development of tolerance. There may be host factors as well as other environmental factors at work. We sought to investigate the clinical phenotypes of children with persistent FAs and compare them to those who outgrew FA. In this abstract we describe the sensitization profile of children with persistent food allergies.

**Methods**

We undertook at case control study of children with persistent FA comparing them to children who had outgrown FA. Patients aged 4–20 with proven tolerance to a previous allergen were allocated to transient FA group (n = 31). Patients aged 12–20 with positive diagnostics for FA were allocated to the persistent FA group (n = 48).

**Results**

The only allergens present in isolation were peanut, treenut, egg and milk. Egg and milk allergies in isolation were less likely to be persistent than nut allergies, however these results were not significant (2/10 (20%) vs. 14/25 (56%) (p = 0.71)) (Tables 1, 2).

**Table 1:** Allergy groups associated with nut and non-nut allergies

	Transient FA	Persistent FA
Non-nut allergy	8	2
Nut allergy	11	14

**Table 2:** Allergy group status compared to number of allergens present

	Mono FA	Multiple FA
Transient FA	19	12
Persistent FA	16	32

Multiple FA was more common amongst persistent FA (32/48 (66.7%) vs. 12/31 (38.7%), p = 0.015).

Several patients (n = 8) had multiple allergies in infancy, but would outgrow some but not all of them (partial persistent FA). All of these patients had at least one persisting allergy, of which peanut was present and persistent in 100%. Similarly, those with multiple FA tended to outgrow egg and milk allergies, while treenut was more likely to persist (Egg 6/6 (100%); Milk 2/2 (100%); Treenut 4/6 (66.7%)). Small group numbers limit the reliability of this data.

When adjusted for eczema status, there was no significant difference between FA group and persistence of eczema (TFA 10/31 (32.3%) vs. PFA 14/40 (35%)).

**Conclusion**

Our findings suggest multiple food allergies are more likely to persist. This knowledge could lead to multiple food sensitisations in childhood being used as a prognostic marker for allergy. Mono FA, which is persistent, is associated with nuts, but other allergens are sometimes involved, the reason for this sensitisation is still unknown. Whilst eczema is linked to food allergen sensitisation, our study has found current eczema does not appear to be a factor in persistence.

## P78

### The safety and efficacy of a strictly structured gradual exposure protocol to baked and heated milk in the treatment of milk allergic children

#### Adi Efron^1^, Liora Halevi^1^, Yuri Zeldin^2^, Avner Reshef^3^, Nancy Agmon-Levine^3^, Ron Kenett^4^, Mona Kidon^5^*

##### ^1^Sackler School Medicine, Tel Aviv University, Tel Aviv, Israel; ^2^Faculty of Medicine, Ben Gurion University, Beer Sheva, Israel; ^3^Allergy and Clinical Immunology Unit, Tel Hashomer, Ramat Gan, Israel; ^4^6.KPA Group and Institute for Drug Research, School of Pharmacy, Hebrew University, Jerusalem, Israel; ^5^Pediatric Allergy Safra Children’s Hospital, Tel Hashomer, Ramat Gan, Israel

**Correspondence:** Mona Kidon - Mona.Kidon@sheba.health.gov.il

*Clinical and Translational Allergy* 2018, **8(Suppl 2):**P78

**Introduction**

A significant proportion of milk allergic children can tolerate extensively heated and baked milk (EHBM). Exposure to these food products may promote tolerance to unheated milk, however, no consensus exists as to the appropriate treatment protocols utilizing EHBM for children with milk allergy. We retrospectively evaluated a well-controlled and structured gradual exposure protocol (SGEP) with EHBM for the treatment of children with CMA

**Methods**

In a case control study, milk allergic children aged 1–4 years of age who were treated with a SGEP-EHBM were compared to children treated with strict avoidance at least until 4 years of age. Data was collected from medical records of children in community and hospital based allergy clinics and from validated telephone questionnaires. Data analysis was performed using nominal logistic regression, the Cox proportional hazard model and generalized regression after an evaluation of the matched case control criteria with propensity scores.

**Results**

42 milk allergic children, 26(62%) males, mean age at intervention 21 months (12–47), were treated with SGEP-EHBM and followed to a mean age of 48 months (24–88). The mean age at resolution of CMA in this intervention group, was compared to a group of 68 milk allergic children following strict avoidance at least until 4 years of age and followed to a mean age of 71 months. We matched for baseline characteristics such as tolerance to heated milk, presence of Atopic Dermatitis (36% vs. 29% controls), asthma (36% vs. 28% controls) and initial anaphylaxis to milk (40% treated vs. 40% controls.) The mean age of resolution of allergy in treated children was 34 months vs. 57 months in control group (p < 0.05). At last follow up, 86% of treated children were tolerant to unheated milk vs. 52% in controls. In the intervention group, there were no adverse events requiring self-administration of epinephrine during or after completion of SGEP.

**Conclusion**

A structured protocol with EHBM appears to be safe and to promote faster resolution of CMA

## P79

### Teaching teachers: towards an educational strategy to improve school teachers’ knowledge on food allergy and anaphylaxis

#### Paloma Poza-Guedes^1^*, Rosa Gloria Suárez López de Verg^2^, Ruperto González-Pérez^1^

##### ^1^Hospital Universitario de Canarias, Sta Cruz de Ten, Spain; ^2^Salud Publica-SCS, Sta Cruz de Ten, Spain

**Correspondence:** Paloma Poza-Guedes - alergocan@hotmail.com

*Clinical and Translational Allergy* 2018, **8(Suppl 2):**P79

**Introduction**

Every year, 1/10,000 children experiences an anaphylactic reaction. Most of these events including attack-related deaths that may happen in school hours. In this study, our goals were to investigate school teachers’ knowledge about food allergy and anaphylaxis, and try to improve it.

**Methods**

During the first half of this school year, a total of 256 teachers working in state schools were included. An educational on-line project held in Tenerife (Spain) was carried out through collaboration of oficial states: Dirección General de Ordenación, Innovación y Promoción Educativa in cooperation with Dirección General de Salud Pública. A pre-/post-tests were used to assess the knowledge that teachers had acquired on food allergy: causes, symptoms, and treatment of food allergic reactions was investigated.

**Results**

About 80% of the teachers considered that food allergy and anaphylaxis is a worrying problem for the teaching staff while only 7.4% of them agreed that it was a worrying issue only for the schoolchild's family. While 14% of the teachers considered themselves prepared to face a severe food allergic reaction in the school, only 23% of them were aware of an adrenaline autoinjector use, and knew where to apply it. More than 80% of the teachers’ responses considered that this specific educational on-line intervention on food allergy and anaphylaxis would improve their knowledge and management on this subject.

**Conclusion**

We believe that our results shows that currently school teachers included are not adequately trained in food allergy and anaphylaxis. There is an urgent need to implement allergy management plans and policies to develop new education strategies including all school staff.

## P80

### Outcome of real-life simulation in the management of anaphylaxis in a Pediatric Emergency Department

#### Simona Barni^1^*, Mattia Giovannini^1^, Francesca Mori^1^, Elio Novembre^1^, Marco De Luca^2^

##### ^1^Allergy Unit, Department of Pediatrics, Anna Meyer Children’s University Hospital, Florence, Italy; ^2^Simulation and Risk Management Unit, Anna Meyer Children’s University Hospital, Florence, Italy

**Correspondence:** Simona Barni - simonabarni@hotmail.com

*Clinical and Translational Allergy* 2018, **8(Suppl 2**):P80

**Introduction**

Anaphylaxis is a life-threatening, rapid onset hypersensitive reaction and it is one of the most common emergencies although under-recognized and under-treated [1].

Simulation is a tool that increases exposure to rare events in a safe enviorment and it allows trainers to develop skills without harm to patients [2].

The purpose of our study was to determine if real-life simulation training improves operational performance in a clinical setting and additionally if it modifies the time latency between the anaphylactic event and the allergological evaluation over ten the years before and after the setting of simulation training as part of the annual educational plan of Anna Meyer Children’s Hospital.

**Methods**

All patients with anaphylaxis referred to the Pediatric Emergency Department (PED) of Anna Meyer Children’s Hospital from 2004 to 2010 [pre-simulation (PRE-s) period] and from 2011 to 2016 [post-simulation (POST-s) period] were retrospectively included in this observational, descriptive study. Diagnosis of anaphylaxis was based on the EAACI guidelines [3]. Clinical characteristics, pharmacological treatments, suspected triggers and results of the allergy work-up were recorded and compared between the two time periods (PRE-s and POST-s).

**Results**

From 2004 to 2010, 82 out of 873 patients, conducted to the PED of Anna Meyer Children’s Hospital for suspected allergic reactions, filled the criteria of anaphylaxis.

The medications used to treat the anaphylactic reactions before (Pre-PED) and after (In-PED) the arrival at the PED were shown in Table 1. The mean time latency between the anaphylactic event and the allergological evaluation was 31.15 ± 43.3 days.

From 2011 to 2016, 136 out of 481 patients evaluated for suspected allergic reactions were anaphylaxis.

The medications used to treat the anaphylactic reactions Pre-PED and In-PED were shown in Fig. 1b. The mean time latency between the anaphylactic event and the allergy evaluation was 27.6 ± 34.9 days.

The epinephrine use has been significantly increased (p < 0.05) comparing the two time periods (PRE-s and POST-s): 1 out of 41 (2.4%) patients and 6 out of 59 (10%) patients, respectively.

During the two time periods (PRE-s and POST-s) we observed also a significant increase (p = 0.011) of the number of patients who underwent a complete allergy work-up: 49 out of 82 (60%) and 120 out of 136 (88.2%) patient, respectively.

**Conclusion**

According to our results the real-life simulation improved the management of anaphylaxis in terms of increase of epinephrine use and number of patients referred to the allergy unit for evaluation.

**References**

1. Johnston EB, King C, Sloane PA et al. Pediatric anaphylaxis in the operating room for anesthesia residents: a simulation study.Paediatr Anaesth. 2017 Feb;27(2):205–210.
2. Bradley P. The history of simulation in medical education and possible future directions. Med Educ. 2006 Mar;40(3):254–62.
3. Muraro A, Werfel T, Hoffmann-Sommergruber K et al. EAACI food allergy and anaphylaxis guidelines: diagnosis and management of food allergy. Allergy. 2014 Aug;69(8):1008–25.

**Table 1:** Treatment of anaphylactic reaction in PRE-s and POST-s period

Medications		2004–2010 (n; %)	2011–2016 (n; %)	*p*
Anthistamines	Pre-PED	23;28	40;43	> 0.05
	In-PED	44;54	99;70	< 0.05
Corticosteroids	Pre-PED	31;39	59;30	> 0.05
	In-PED	49;60	95;73	< 0.05
Bronchodilatators	Pre-PED	14;17	10;7	< 0.05
	In-PED	18;22	18;13	> 0.05
Epinephine IM	Pre-PED	5;6	8;6	> 0.05
	In-PED	1;2, 4	6;10	< 0.05
No treatment	Pre-PED	35;43	48;73	> 0.05
	In-PED	19;23	12;30	< 0.05
Oxigen	Pre-PED	np	np	–
	In-PED	np	5; 4	> 0.05
Fluid IV	Pre-PED	np	3; 33	> 0.05
	In-PED	np	33; 24	< 0.05
Epinephrine aerosol	Pre-PED	np	3; 2	> 0.05
	In-PED	np	1; 0.7	> 0.05

## P81

### Avocado and banana-induced Food Protein–Induced Enterocolitis Syndrome (FPIES): case report of a rare trigger

#### Evangelia Stefanaki^1^*, Argiro-Stamatia Manogiannaki^2^, Angeliki Tzagkaraki^2^, Angeliki Ftylaki^1^, Maria Anatoliotaki^1^, Sofia Stefanaki^2^, Georgia Vlachaki^2^

^1^Outpatients Pediatric Allergy Clinic, Venizelion General Hospital, Heraklion, Greece; ^2^Department of Pediatrics, Venizelion General Hospital, Heraklion, Greece

**Correspondence:** Evangelia Stefanaki - linastef74@gmail.com

*Clinical and Translational Allergy* 2018, **8(Suppl 2):**P81

**Introduction**

FPIES is an increasingly diagnosed non-IgE mediated hypersensitivity reaction characterized by profuse vomiting with or without diarrhea leading to dehydration, hypotonia and lethargy. Common triggers differ around the world including milk, soy, rice, poultry, fish.

**Case report**

A 7 month old boy presented in our Outpatients Pediatric Allergy Clinic with a history of reactions to fruits. He was growing well and had no history of atopic dermatitis or wheezing episodes. He was exclusively breastfeeding until 5 months of age when he started tasting fruits, added one by one in small quantities—1st day apple, 2nd day plus pear, 3rd day plus banana, 4th day plus kiwi, 5th day plus strawberry, 6th day plus nectarine. On day 5, he also started mixed vegetables (½ teaspoon of potato, carrot, zucchini and celery). On day 6 and about 30 min after ½ teaspoon of mixed vegetables and 3 h after mixed fruits he vomited once. Next day he tried again ½ teaspoon of the same mixed fruits and 30 min later he started profuse vomiting for 6 h with nausea, pallor and hypotonia. He was admitted in hospital for 24 h where he was rehydrated. He stopped all solids and 1 week later he was reintroduced fruits one by one every 3 days and more than 30 min after tasting less than ½ teaspoon of banana he started again profuse vomiting for 4 h with hypotonia 0.4 h later he also had 3 diarrheas. He continued breastfeeding and eating all fruits and vegetables he had already tried before plus veal and spaghetti except banana. At 6.5 months of age he tried ½ teaspoon of avocado (family culture) and 1 h later he reacted again with 6 vomits and diarrheas 4 h later. Skin prick tests and specific IgEs were performed to avocado, banana, cow’s milk, egg white, egg yolk, cod, peach, peanut, cashew and all were negative.

Mother was informed to avoid giving avocado and banana to our young patient and to proceed to other solids one by one but without special exclusions.

**Conclusion**

To our knowledge there is only one report in the literature of FPIES to avocado coming this year from USA so this is the first official report in Europe and the first avocado-banana FPIES case. Clinicians should be aware that less common solid foods could also be triggers of FPIES.

**Consent to publish**

The authors have obtained parental informed consent of the patient mentioned in the article.

## P82

### Abdominal cramps, a rare clinical manifestation of food protein-induced allergic proctocolitis

#### Esmeralda Shehu^1^*, Anxhela Gurakuqi Qirko^2^, Diana Qama^3^, Klejta Xhafaj^4^, Mirela Hasanaj^5^

##### ^1^Internal Medicine, Regional Hospital of Durres, Durres, Albania; ^2^University of Medicine, Department of Pathophysiology, Tirane, Albania; ^3^Internal Medicine, Regional Hospital of Berat, Berat, Albania; ^4^Polyclinic nr 3 Tirane, Albania; ^5^UHC Mother Teresa, Tirane, Albania

**Correspondence:** Esmeralda Shehu - shehuesmeralda@yahoo.com

*Clinical and Translational Allergy* 2018, **8(Suppl 2):**P82

**Introduction**

Food protein-induced allergic proctocolitis (FPIAP) is a non IgE mediated allergic disease which appears in the first few months of life. Approximately 60% of cases occur in breastfed infants, where the principal causes are cow’s milk proteins. Bloody and mucoid stools are the most common clinical presentation and, abdominal cramps are rare in FPIAP.

**Case Report**

A 7 months old boy, mainly breastfed presented blood and mucus within the stool and, abdominal cramps several times a day. Firstly, he experienced these symptoms at the age of 1 month. At that time he developed a moderate form of eczema too. He was referred at the pediatrician who treated him with simethicone and emollients. But besides the treatment all the symptoms persisted. At the age of 3 months he was referred at another clinic with the same complaints. He continued to take simethicone, emollients and added probiotics with a slight clinical improvement of AD. Rectal bleeding and abdominal cramps still persisted. At the age of 7 months he was referred at our allergy clinic. A complete blood count, lever and renal function were normal. GI infections were ruled out by culture and sensitivity testing. CRP was increased and an abdominal ultrasound resulted normal. His father suffered ulcerative colitis and allergic rhinoconjuctivitis.

On physical examination he showed normal growth indicators according to WHO standards. FPIAP, ulcerative colitis and food induced allergic eczema were clinically suspected. Total IgE was slightly increased, specific IgE to milk was 0.25 IU/ml. Colonoscopy revealed multiple ulcerated inflammatory areas of intestinal mucosa. Biopsy from the colon indicated chronic colitis with eosinophilic infiltration. The FPIAP diagnosis was made. We recommended the mother to start on a strict elimination diet of cow milk and other dairy products and, to continue the breastfeeding. He started the therapy with pimecrolimus cream and emollients for his eczema. After 2 weeks he didn’t experience any abdominal cramp and the stools slowly normalized. His atopic dermatitis was improved significantly. The patient continues his regular follow up in our clinic. We successfully performed a milk challenge with a very good tolerance when he was 1 year old.

**Conclusion**

Clinicians should consider FPIAP as a possible diagnosis in children with bloody and mucoid stools and abdominal cramps. Colonoscopy and biopsy are very important tools to exclude other colon diseases and to support allergic FPIAP diagnosis. The exclusion of cow’s milk from nursing mother diet proved effective in our case.

**Consent to publish**

The parents of the patient has provided written consent to publish.

## P83

### Impact of counselling on the safety profile of oral immunotherapy for food allergy during maintenance phase

#### Stefania Arasi^1^*, Lucia Caminiti^1^, Giuseppe Crisafulli^1^, Giovanni Passalacqua^2^, Giovanni Pajno^1^

##### ^1^University of Messina, Messina, Italy; ^2^Allergy and Respiratory Diseases, Ospedale Policlinico San Martino - University of Genoa, Genoa, Italy

**Correspondence:** Stefania Arasi - stefania.arasi@yahoo.it

*Clinical and Translational Allergy* 2018, **8(Suppl 2):**P83

**Introduction**

Oral immunotherapy (OIT) can effectively induce a clinical desensitization in patients with persistent IgE-mediated allergy to cow’s milk (CMA) and hen’s egg (HE). However, its safety remains one of the major concerns, as adverse events (AEs) are quite frequent, unpredictable and unexpected. AEs can occur with a previously tolerated dose of the offending food during the maintenance phase of desensitization, usually managed at home.

The objective is to assess the impact of a specific counseling and a specific written plan to avoid or reduce AEs occurrence during the maintenance regimen.

**Methods**

A retrospective analysis was conducted, including two homogeneous groups successfully desensitized by the same OIT protocols. Group A not receiving specific counseling about the possible AEs and Group B receiving counseling and a written plan on how to avoid or reduce AEs during the maintenance regimen.

**Results**

Group A included 62 patients, 35 desensitized for CM and 27 for HE between 2004 and 2012 (18 male, age range 4–13 years**)** and Group B included 34 patients, 10 desensitized for CM and 14 for HE between 2013 and 2016 (17 male, age range 4–14 years). Overall, in Group B (who received a written plan), the rate of AEs was 3% of patients versus 21% in Group A (p = 0.002) (Table 1).

**Table 1:** Description of the adverse events (AEs) during maintenance phase in the two groups

	GROUP A (n = 62)		GROUP B (n = 34)	
	COW'S MILK	HEN'S EGG	COW'S MILK	HEN'S EGG
Patients (n)	35	27	20	14
Mean age (range)	9 (4–13)	7 (4–11)	8 (4–12)	8 (4–14)
Male/female	18/17	15/12	11/9	6/8
Mild-moderate reactions	4	0	1	0
Severe reactions	5	3	0	0
Eosinophilic esophagitis (n)	1	0	0	0
Total patients with AEs	10	3	1	0

**Conclusion**

With a proper information and a structured written instruction plan, the risk of possible AEs during the maintenance phase of food desensitization can be significantly reduced, still maintaining the efficacy of the treatment.

## P84

### Etiology and characteristics of patients presenting with anaphylaxis to the Pediatric Emergency Centers in Qatar

#### Mehdi Adeli*, Kahlid Alyafei, Ahmed AlShami, Sabha Nisar

##### Hamad Medical Corporation, Doha, Qatar

**Correspondence:** Mehdi Adeli - madeli@hamad.qa

*Clinical and Translational Allergy* 2018, **8(Suppl 2):**P84

**Introduction**

Anaphylaxis is an acute life–threatening allergic reaction. Qatar having the highest GDP per capita aims to provide the best healthcare services. There are five Pediatric Emergency Centers (PEC) distributed countrywide, serving an average of 610,000 patients annually. The triggers, co morbid diseases and management due to anaphylaxis have not been locally investigated yet and this is the first study on the topic.

This retrospective study is aimed to analyze the characteristics and etiology of patients presenting with anaphylaxis.

**Methods**

Patient records of all children younger than 14 years, who presented to PEC during the period of 2011 to 2016 were identified by using ICD- 9 diagnostic codes for anaphylaxis. The patient charts for clinical presentation, management, pre-existing comorbid and outcome were reviewed and classified according to the criteria set by National Institute of Allergy and Infectious Diseases/Food Allergy and Anaphylaxis Network for Anaphylaxis diagnosis.

**Results**

Anaphylaxis was identified in 390 out of the 1051 files reviewed. The incidence was 13.3 per 100,000 visits. Patients below 1 year of age were 17%. The mean age was 3 years and males 69%. Among triggers; food items were 54%, insect venom 25%, medication 7%, aeroallergens 3% and Idiopathic 10%. Asthma was associated with all 5 cases admitted to PICU and with 55% of recurrent anaphylaxis (36%) with no deaths reported. Biphasic reactions were observed in 2%. Comorbid conditions including asthma in 42%, eczema in 27% with 23% below 1 year of age. Among the food; nuts were 38%, unspecified nuts 26%, peanuts 5%, dairy products 10% with 85% of these cases below 2 years, sesame seeds and egg 7% for each, seafood 4% while camel milk in 1 case. Black ant was 48% among insect bites. Horse dander in 2 cases, camel hair, latex and MMR vaccine each had one case. Presenting symptoms include cutaneous in 92% of cases, respiratory 73%, gastrointestinal 41%, cardiac 9% and neurological 5%. Intramuscular adrenaline was used for treatment in 91%, antihistamines in 88%. Epinephrine auto-injectors were prescribed upon discharge for 83%. Referral to allergy clinic was seen in 82%, where 60% were followed up and investigated.

**Conclusion**

Anaphylaxis is a life threatening problem, affecting 1 in 1000 Qatari pediatric population. Physician and community awareness of anaphylaxis; its etiology, management and comorbid diseases associated with it is highly recommended.

## P85

### Use of self-injectable epinephrine among children with food allergy

#### Alberto Alvarez-Perea*, Victoria Fuentes-Aparicio, Paula Cabrera-Freitag, Sonsoles Infante, Oliver Muñoz-Daga, Jose M. Zubeldia, Maria L. Baeza

##### Allergy Service, Hospital Materno Infantil Gregorio Marañón, Madrid, Spain

**Correspondence:** Alberto Alvarez-Perea - alberto@alvarezperea.com

*Clinical and Translational Allergy* 2018, **8(Suppl 2):**P85

**Introduction**

Food allergy is the most important trigger of anaphylaxis among the pediatric population. Epinephrine is the drug of choice for the treatment of anaphylaxis, and self-injectable epinephrine devices (SIE) are the preferred method to early treat it in the community. However, its use seems scarce.

**Methods**

A cross-sectional study was led in a third-level hospital in Madrid, Spain, from September to November 2016. Two hundred consecutive patients attended in the Pediatric Allergy Unit with a diagnosis of food allergy were asked to complete a survey related to their allergy. Patients that had previously received a SIE prescription were recruited. A written informed consent was obtained.

**Results**

The survey was filled by 193 patients, of which 103 (53.4%) had been prescribed SIE. The cohort included 40 girls (38.8%) and 63 boys (61.2%), with a median age of 9 years (interquartile range 5). Seventy-nine of them (76.7%) had a history of anaphylaxis, 84 (81.6%) had other allergic diseases. The main food allergy elicitors were nuts (54.4%), cow’s milk (35.9%) and egg (34%). Time of evolution since the food allergy diagnosis: less than 1 year 5.8%, 1–5 years 22.3%, over 5 years 71.8%.

Eighty-eight (85.4%) of the patients who had been prescribed SIE declared to always carry the device with them.

Fifty-four (52.4%) of the patients that got a SIE prescription, had a food-triggered allergic reaction after the prescription, within the previous year. Twenty of them (37%) had suffered anaphylaxis. Overall, 12 (22.2%) had received epinephrine. Only 4 (7.4%) of them had used their SIE, while 9 (16.7%) had received epinephrine in a healthcare center.

**Conclusion**

Accidental exposures are not uncommon among children already diagnosed at risk of food-triggered anaphylaxis. Although most of the patients who had SIE prescribed declared to carry it, they rarely use it. Anaphylaxis in the community remains undertreated.

## P86

### Food allergic consumer’s views and practices regarding allergist advice and food allergen labeling

#### Sophia Koo^1^, Georgios Raptis^2^*, Konstantinos Gerasimidis^1^

##### ^1^Human Nutrition, University of Glasgow, School of Medicine, Dentistry and Nursing, Glasgow, United Kingdom; ^2^Royal Hospital for Children, Glasgow, United Kingdom

**Correspondence:** Georgios Raptis - george.raptis@nhs.net

*Clinical and Translational Allergy* 2018, **8(Suppl 2):**P86

**Introduction**

Advice from healthcare professionals on how to interpret the food allergen labelling plays an important role in assisting patients and parents in managing food allergy. On the other hand, the Precautionary Allergy Labelling, is troublesome to many food-allergic consumers due to its ambiguities. Therefore, implementation of a risk-assessment approach, such as the Voluntary Incidental Trace Allergen Labelling (VITAL) used elsewhere is recommended to improve the current food allergen labelling. The aim of this survey was to evaluate allergists’ advice on food allergen labelling, as well as to understand food-allergic patient and parental views and practices based on the food allergen labelling.

**Methods**

Patients and parents attended a tertiary food allergy clinic were asked to complete a 14-item validated questionnaire at the end of their consultation

**Results**

Data from a total of 70 respondents were obtained and analyzed. Advice from the allergist was valued by the food-allergic consumers, yet the majority of them greatly depended on the information provided on the food labels when purchasing food. 43% of the respondents claimed that the PAL statement was not useful to them and the majority of the food-allergic consumers (86%) agreed that manufacturers should carry out a risk-assessment approach for the PAL. For those with severe-multiple food allergies, quality of life was much more affected due to limited food products they can purchase.

**Conclusion**

Findings of this small survey stress the importance of shared responsibility between stakeholders responsible to assist and protect individuals with food allergy. Advice from the allergist, along with clear and consistent labelling of food allergens may alleviate patient and parental anxiety in managing food allergy.

## P87

### The prevalence of pollen food syndrome and its sensitization profiles in Ukrainian pollen-allergic children

#### Tetiana Umanets*, Yuriy Antipkin, Volodymyr Lapshyn, Svitlana Matveeva

##### Institute of Pediatrics, Obstetrics and Gynecology, Kiev, Ukraine

**Correspondence:** Tetiana Umanets - tetiana.umanets@gmail.com

*Clinical and Translational Allergy* 2018, **8(Suppl 2):**P87

**Introduction**

Pollen-food syndrome (PRS) is mediated by immunoglobulin E antibodies; symptoms arise as a result of cross-reactivity between pollen and plant derived food, mainly in adults. Unfortunately, there is no data about the prevalence of PFS in Ukrainian children with pollinosis.

The aim of this study was to evaluate the prevalence of PFS and its sensitization profiles in Ukrainian children with seasonal allergic rhinitis/rhino conjunctivitis.

**Methods**

A total of 140 patients aged 5–17 with seasonal allergic rhinitis/rhino conjunctivitis were enrolled in this study. The questionnaire on rhinitis and food symptoms, skin prick testing with commercial extracts and fresh fruits or vegetables, specific Ig E to major pollen molecules, profillins (Bet v 2, Phl p 12) and LTP (Art v 3) using ImmunoCAP were performed.

**Results**

Clinical diagnosis of PFS was made in 51 (36.4%) patients with median aged 9 y.o. and 38 (74.5%) of those were male. In patients sensitized to weed, birch tree and grass pollen, the prevalence of PFS ranged respectively 54.9%, 36.1%; 3.6%. Nuts, apple, bananas and tomatoes were the food most commonly implicated. Among included patients, 5 (3.6%) of the children had a history of anaphylaxis to nuts. Ig E to Bet v 1 and Bet v 2 was observed in 6 (27.3%) PFS (+) patients and associated with > 2 fruits more likely to be hazelnut and apple vs. 2 (5.1%) PFS (-) birch-allergic patients. In the group of PFS (-) grass-allergic patients, two patients showed Ig E to Phl p 12. There were no patients with IgE to Atr v 3.

**Conclusion**

Birch and weed–related PFS are common in Ukrainian pollen-allergic children, with nuts and fruit predominantly implicated. The main source of profiling sensitization was birch pollen, and profiling sensitization was associated with PFS and larger number of offending foods.

## P88

### Introduction and maintenance of early adaptive training protein blends in support of infant nutritional goals: safety and acceptability

#### Jany Holl^1^*, Lucy Bilaver^1^, Daniel Finn^1^, Kay Savio^2^

##### ^1^Northwestern University, Chicago, IL, United States of America; ^2^Focus Pointe Global, Inc., St. Louis, MO, United States of America

**Correspondence:** Jany Holl - j-holl@northwestern.edu

*Clinical and Translational Allergy* 2018, **8(Suppl 2):**P88

**Introduction**

Childhood food allergy affects about 8% of US children [1]. Recent research has revealed protective effects of early dietary introduction of allergenic foods on the development of food allergy for infants, including those at elevated risk [2, 3, 4]. The goal of this study was to evaluate the safety and acceptability of a blend of 16 common allergenic proteins (peanut, soy, almond, cashew, hazelnut, pecan, pistachio, walnut, wheat, oat, milk, egg, cod, shrimp, salmon, sesame) combined with 400 IU of Vitamin D into a food supplement powder.

**Methods**

Caregivers were instructed to mix the powder into a solid or liquid feeding once a day. All procedures were deemed exempt by the Northwestern University Institutional Review Board. A national sample (from 32 states) of healthy infants, 5–11 months of ages, without severe eczema, participated in the 28-day placebo period followed by a 28-day randomized, blinded, placebo-controlled period. Caregivers added one packet (approximately 1 tablespoon) of the placebo/food supplement powder to a feeding once a day. Caregivers were instructed to observe their infant for 2 h after the feeding and to record any symptom(s) or allergic type reaction(s) including anaphylaxsis [5] occurring within 2 h after the feeding, any reaction-related prescribed medication or medical care, in a daily, web-based diary.

**Results**

Figure 1 shows enrollment and completion rates of the study. Of the 19,208 placebo ingestions, 2% of ingestions resulted in a reported symptom (e.g., cough, diarrhea). Of the 8827 food supplement ingestions to date, no infant has had any reported allergic reaction, received any prescribed medication, or received medical care related to a reaction within 2 h of ingestion. Final results will be available for the entire cohort in 3 weeks.

**Fig. 1:**
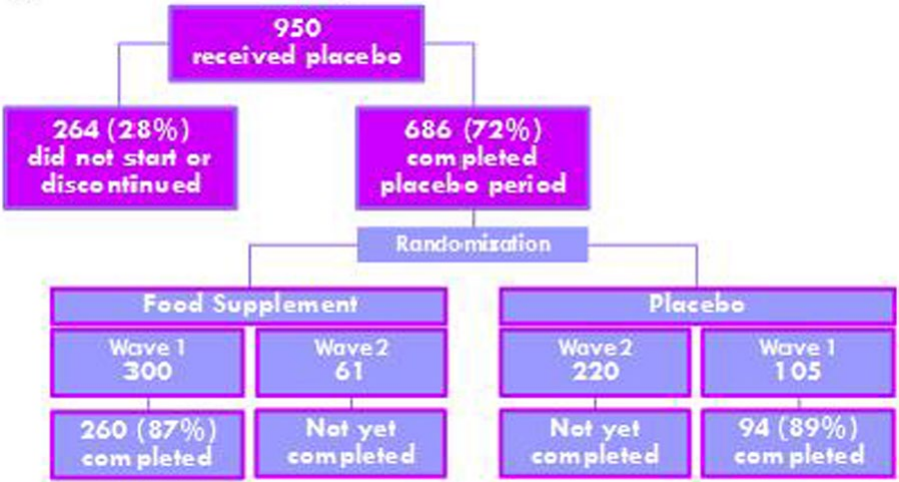
Enrollment and completion rates

**Conclusion**

At present, this study strongly suggests that the food supplement is safe and feasible for infants. Future study should assess the effect of the food supplement on immunologic responses to the allergenic proteins and on the longer-term incidence of food allergy.

**References**

1. Gupta et al. The Prevalence of Childhood Food Allergy. Pediatrics 2011
2. Fleischer et al. Primary Prevention of Allergic Disease Through Nutritional Interventions. Allergy Clin Immunol 2013
3. Perkin et al. Randomized Trial of Introduction of Allergenic Foods in Breast-fed Infants. N Engl J Med 2016
4. Du Toit et al. Randomized Trial of Peanut Consumption in Infants at Risk for Peanut Allergy. N Engl J Med 2015
5. Sampson et al. Second Symposium on the Definition and Management of Anaphylaxsis: Summary Report—Second National Institute of Allergy and Infectious Disease/Food Allergy and Anaphylaxsis Network Symposium. JACI 2006

## P89

### Food refusal in autism: Is it undiagnosed food allergy?

#### Miranda Crealey*, Aideen Byrne

##### Department of Pediatric Allergy, Our Lady’s Children’s Hospital Crumlin, Dublin, Ireland

**Correspondence:** Miranda Crealey - mirandac@eircom.net

*Clinical and Translational Allergy* 2018, **8(Suppl 2):**P89

**Introduction**

Autistic spectrum disorder (ASD) is a neurodevelopmental disorder associated with restrictive or repetitive behaviours and difficulties with verbal and interpersonal communication. There has been a 269% increase in prevalence in ASD from 4.2 per 1000 in 1996 to 15.5 per 1000 in 2010 [1]. The incidence of food allergy is also increasing [2]. Dysbiosis of the human microbiome has been linked with both food allergies and autism [3].

Estimates suggest upwards of 90% of children with ASD experience some type of feeding related concern [4]. Food selectivity (i.e., only eating a narrow variety of foods by type texture, and/or presentation) represents the most pervasive feeding issue while rejecting most (if not all) fruits, vegetables, and/or proteins [5]. Concurrently, food refusal is well described in children with food allergy; selective avoidance of egg containing foods by young children with egg allergy. However, more complex feeding disorders are increasingly being recognised in children with food allergy with fussy eating at baseline being more common [6]. The incidence of food allergy in children with autism is unknown but is likely significantly underestimated. Food refusal may represent avoidance from foods which cause symptoms of food allergy e.g. oral tingling/itching in oral allergy syndrome.

**Case report**

Table 1 reports the clinical characteristics of three children with ASD attending our tertiary referral allergy service. All three have at least one confirmed immediate allergic reaction to food. All 3 children were sensitised to an extensive profile of foods.

**Table 1:** Clinical characteristics of 3 children with ASD and confirmed food allergy

	Case 1	Case 2	Case 3
Referral source	ED	GP	dermatology
Reason for referral	Egg anaphylaxis	Multiple food refusals	Multiple food reactions
Age	3	7	9
Atopic disease	Eczema, asthma	Eczema, asthma	Eczema, asthma, AR
Clinical reactions to food	Egg anaphylaxis	Milk	Milk, egg, kiwi, wheat
Food intake	Repeatedly spits out food Multiple food refusals	Food refusal and aversion to most foods since weaning	Multiple food refusals, avoids all fruit and vegetables
Sensitisations (SPT or specific IgE)	Egg, peanut, treenuts, salmon, tuna, cod, sesame seed, beef, chicken, banana, broccoli.	Egg, peanut, treenuts, cod, wheat, chicken, sesame seed, banana, rice, carrot, pear, soya, Pru P 4 Profilin Peach	Wheat, milk, peanut, tree nuts, cod, soya rPru P 3 LTP Peach rPru P 4 Profilin Peach Birch PR-10 rBet v1 Birch Profilin rBet v2

*ASD* autistic spectrum disorder, *ED* emergency department, *GP* general practioner, *AR* allergic rhinitis, *SPT* skin prick test

All 3 children display behaviours to multiple foods that they may or may not be sensitised to including food refusal, aversion, spitting out, gagging leading to significant dietary restriction. Case 2 failed to thrive and had vitamin D deficiency and was extensively investigated to out rule other organic causes e.g. Eosinophilic Oesophagitis. All children were non-verbal. Parental anxiety was significant in all cases. Food Challenges were not feasible due to the children’s behaviours thus it is impossible to establish for certain whether specific food refusals were due to dysfunctional eating habits of ASD or true food allergy symptoms.

**Conclusion**

We wish to highlight that physicians face challenges both in the diagnosis and management of food allergy in the autistic child. The history may be that of multiple food refusal and food aversions. Symptoms of non-IgE mediated allergy and of oral allergy syndrome (OAS) may not be easily identifiable to the parent or the physician and may not be communicated by the autistic child. Investigations are limited and often multiple sensitisations are present. Skin prick testing and blood testing may not be successful with oral food challenges often impossible in an autistic child. We recommend that parents must be guided by food refusals in these children and that they are supported by dietetics, psychologists and speech and language to avoid nutritional deficiencies and a feeling of abandonment.

**Consent to puplish**

The parents of all 3 cases provided informed consent.

**References**

1. Van Naarden Braun K, Christensen D, Doernberg N, Schieve L, Rice C, Wiggins L, et al. Trends in the prevalence of autism spectrum disorder, cerebral palsy, hearing loss, intellectual disability, and vision impairment, metropolitan atlanta, 1991–2010. PLoS One. 2015;10(4):e0124120.
2. Branum AM, Lukacs SL. Food allergy among children in the United States. Pediatrics 2009;124(6):1549–55.
3. Haahtela T. What is needed for allergic children? Pediatr Allergy Immunol. 2014;25(1):21–4.
4. Ledford JR, Gast DL. Feeding problems in children with autism spectrum disorders: A review. Focus Autism Other Dev Disabl. 2006; 21:153–166.
5. Sharp WG, Jaquess DL, Luckens CT. Multi-method assessment of feeding problems among children with autism spectrum disorders. Res Autism Spectr Disord. 2013; 7:56–65.
6. Maslin K, Dean T, Arshad SH, Venter C. Fussy eating and feeding difficulties in infants and toddlers consuming a cows’ milk exclusion diet. Pediatr Allergy Immunol 2015: 26: 503–508.

## P90

### Do parents successfully introduce peanut following a negative open peanut challenge in young children?

#### Helyeh Sadreddini^1^, Heidi Ball^1^, Kristian Bravin^1^, David Luyt^1^, Mhorag Duff^2^, Gary Stiefel^1^*

##### ^1^University Hospitals of Leicester NHS Trust, Leicester, United Kingdom; ^2^Leicestershire Partnership NHS Trust, Leicester, United Kingdom

**Correspondence:** Gary Stiefel - garystiefel@icloud.com

*Clinical and Translational Allergy* 2018, **8(Suppl 2):**P90

**Introduction**

The LEAP study showed that in infants at high risk of peanut allergy (PA) early introduction (between 4 and 11 months old) and regular consumption of peanut reduced the risk of subsequently developing PA. These findings were confirmed in the EAT study but in a cohort of unselected infants. The observations seen in these two studies may well be dose dependent as compliance seemed to impact on the PA preventative efficacy of early peanut introduction. We consequently assessed in our allergy service whether early peanut introduction following a negative peanut challenge is successful in a clinical setting; and how parents perceived this.

**Methods**

In the Children’s Allergy Service in Leicester, we started assessing children < 2 years old presenting predominantly with egg allergy and/or moderate to severe eczema for peanut allergy from March 2015. Where peanut SPTs were ≥ 1 mm, patients’ were offered an oral peanut challenge to determine allergy. We conducted telephone interviews to assess progress in February and March 2017 of patients with negative challenges to December 2016. Parental satisfaction was also sought.

**Results**

The carers of 65% (41/63) infants with negative challenges were successfully contacted. Twelve meet LEAP criteria, 12 meet LEAP criteria but were > 12 months old and 17 did not meet the criteria. Peanut was successfully introduced in 34 (83%); 28 consumed peanut at least once a week of which 15 were more than 3 times a week. Fourteen consumed at least 2 g per portion and a further 9 managed a 2 g portion some of the time.

Reasons for failure to introduce peanut were parental anxiety, disliking peanut, burdensome and in 2 cases suspected allergic reactions (Rash after 2 h and eczema flare).

Parents contacted were satisfied with the clinic appointment and subsequent challenge service as over 80% scored ≥ 9 on a Likert scale.

**Conclusion**

We showed that after successful peanut challenge in young children, a high proportion of carers subsequently interviewed introduced peanut into their child’s diet. However as a third were not contacted, we can only be sure that just over half complied with advice. Perhaps closer contact with families may improve feedback and compliance although those who were contacted expressed satisfaction with our service.

## P91

### Wheat-dependent exercise-induced anaphylaxis: a rarity to remember

#### Nafsika Sismanoglou*, Dinkar Bakshi

##### Nottingham Children’s Hospital, Nottingham University Hospitals Trust, Nottingham, United Kingdom

**Correspondence:** Nafsika Sismanoglou - nausicasigma@gmail.com

*Clinical and Translational Allergy* 2018, **8(Suppl 2):**P91

**Introduction**

Wheat is a widely consumed cereal and one of the commonest food allergens^(1)^.Occasionally, wheat ingestion triggers symptoms with varying levels of physical activity and presence of cofactors (e.g. NSAIDS, oestrogens, infections)^(2)^. Wheat-dependent exercise-induced anaphylaxis (WDEIA) is rare, with estimated prevalence of under 0.1%^(3)^.Although potentially life threatening, diagnosis is challenging due to the inconsistency of symptoms. A ‘provocation’ food challenge followed by exercise in a controlled environment remains the gold standard for diagnosis ^(4)^.

**Case report**

A 13 year old girl was diagnosed with tree nut allergy at 2 years age. She was sensitised to pistachionut, cashewnut and walnut, both on skin prick testing (SPT) and specific IgE antibody (sIgE) testing. She had seasonal allergic rhinitis with grass pollen sensitisation and atopic background with a total IgE antibody level of 500 kU/L. She was not sensitised to wheat on tests. The girl had outgrown her infantile atopic dermatitis and did not have symptoms of asthma.

About 3 years prior to the presentation, she had an episode of generalised rash following ingestion of bread at school. She continued to have sporadic episodes of mouth itching, flushing, generalised urticaria, colicky abdominal pain, vomiting and diarrhoea, associated with ingestion of pasta followed by vigorous physical activity (tennis, running). The symptoms would manifest within minutes of initiating exercise, and would always subside with antihistamines. She is a keen tennis player with no symptoms when avoiding wheat prior to exercise. The history was strongly indicative of WDEIA, therefore a ‘provocation’ food challenge with exercise was deemed redundant. Currently, she has gluten free diet in school and is careful to avoid exercise following wheat ingestion at home. She is excluding all nuts in her diet and was prescribed antihistamines and adrenaline autoinjector devices for emergency use.

**Conclusion**

WDEIA is a rare condition, with inconsistent symptoms and variable presentation, accounting for delays in diagnosis up to 62 months^(5)^.The lack of an identifiable trigger causes not only unnecessary anxiety to families, but also restrictive and impracticable diet patterns. The diagnosis is dependent on a good history since both SPT and sIgE antibody testing may not show sensitisation to wheat^(6)^. Notably, a delay in diagnosis exposes patients to the risk of severe or even a life threatening reaction. Therefore, there is a clear need to raise awareness amongst clinicians about WDEIA.

**Consent to publish**

The family gave consent for publication of the anonymised clinical details used in this abstract.

**References**

1. Cianferoni A. Wheat allergy: diagnosis and management. *Journal of Asthma and Allergy.* 2016:9 13–25
2. Wolbing F, Fischer J, Koberle M, Kaesler S, Biedermann T. About the role and underlying mechanisms of cofactors in anaphylaxis. *Allergy* 2013, 68:1085–1092
3. Barg W, Medrala W, Wolanczyk-Medrala A, Exercise-induced anaphylaxis: An update on diagnosis and treatment. *Curr Allergy Asthma Rep.* 2011, 11:45–51
4. Povesi—Dascola C and Caffarelli C. Exercise-induced anaphylaxis: A clinical view.*Italian Journal of Pediatrics* 2012, 38:4
5. Wong GK, Huissoon AP, Goddard S, Collins DM, Krishna MT. Wheat dependent exercise induced anaphylaxis: is this an appropriate terminology?, *J Clin Pathol.* 2010 Sep;63(9):814–7.
6. Kleiman J, Ben-Shoshan M. Food-dependent exercise—induced anaphylaxis with negative allergy testing, *BMJ Case Rep* 2014.

## P92

### The association of stress during pregnancy with the development of food allergies in childhood: a pilot retrospective questionnaire based study

#### Soo K. Oh^1^, Susan Leech^2^, Imran Ali^1^, Cherry A. Alviani^2^*

##### ^1^Department of Medicine, King’s College, London, United Kingdom; ^2^Department of Pediatrics, King’s College Hospital, London, United Kingdom

**Correspondence:** Cherry A. Alviani - cherryalviani@googlemail.com

*Clinical and Translational Allergy* 2018, **8(Suppl 2):**P92

**Introduction**

Food allergy is increasingly common. Prenatal maternal stress is associated with asthma and allergic rhinitis, but its role in food allergy has not been clarified. This retrospective case–control study investigates whether mothers of children with food allergy experienced greater prenatal stress than mothers of children without food allergy.

**Methods**

Mothers of children attending the Allergy and General Pediatric Clinics at King’s College Hospital and Croydon University Hospital, from March to July 2016, were asked to complete a questionnaire. The questionnaire captured pre-and post-natal demographic details. Prenatal stressful experiences were recorded by 11 events from the Sarason’s Life Experience Survey, with two additional free-text entries. A total of 13 negative events were recorded per mother. Perceived pre-natal stress was assessed using a modified Cohen’s Perceived Stress Scale and Prenatal Distress Questionnaire, generating a 9 item questionnaire with Likert scale responses. Differences in stress scores were analyzed using SPSS. Regional ethical approval was obtained and consent implied upon completion of a questionnaire.

**Results**

32 mothers of children with food allergy (FA) and 40 mothers of children attending general pediatric clinics (controls), without food allergy, were recruited. Children were older in the FA group (2.89 years vs. 1.96 years p = 0.019). The FA group had a higher rate of emergency caesarean (25.8% vs. 7.50%; p = 0.048), higher rate of eczema (75% vs. 35.9%; p = 0.001) and higher rate of atopic family history (90.63% vs. 60.53%; p = 0.006). There was trend towards a higher number of pre-natal stressful events within the FA group- 1.94 (SD 2.11) events per mother vs. 1.58 (SD 1.85) in controls, which was not statistically significant (p = 0.761). In particular, higher rates of illness during pregnancy were reported by mothers of FA children. There was no difference between the two groups in maternal perceived stress, with an average score of 82.6 (SD.31.9) in the FA group vs. 88.3 (SD 40.1) (p = 0.514, 95% CI -11.70 to 23.18).

**Conclusion**

This study demonstrates a trend towards higher number of pre-natal negative events, particularly medical illness, in mothers of children developing food allergy compared to controls recruited from general pediatric clinics. This difference was not statistically significant, but study numbers were small. FA children were older than controls, and the control group had higher rates of atopy than expected from a general population. Further exploration of the link between maternal pre-natal illness and food-allergy is needed.

## P93

### Pediatric anaphylaxis in the Emergency Department: incidence, provocative factors and use of epinephrine

#### Hiske Mevius^1∞^*, Miranda Wiggelinkhuizen^2∞^, Wilma Vriesman^1^

##### ^1^Pediatric Department, Albert Schweitzer Hospital, Dordrecht, The Netherlands; ^2^Pediatric department, Erasmus MC - Sophia Children’s Hospital, Rotterdam, The Netherlands

^∞^First authors that contributed equally to this work

**Correspondence:** Hiske Mevius - hiskemevius@gmail.com

*Clinical and Translational Allergy* 2018, **8(Suppl 2):**P93

**Introduction**

Anaphylaxis is a severe, potentially fatal systemic reaction with a rapid onset, after contact with a causative allergen (1). The exact incidence of pediatric anaphylaxis in the Emergency Department (ED) is unknown (1, 2). According to a recent European study, an estimated range is from 0.11 to 0.41%(3), with a rising worldwide overall incidence (4, 5). The objective of this study is to determine the incidence of pediatric anaphylaxis in the ED and to define causative allergens. As some studies show striking evidence for non adherence to the protocol of treatment of anaphylaxis (6–10), reflected by the low percentage of epinephrine application, we studied the frequency of epinephrine gifts as well.

**Methods**

A retrospective study was performed at the ED in a large general hospital in The Netherlands. All patients from 0 to 18 years that presented from January 2012 until December 2016 with anaphylaxis, with Sampson score ≥ 3, were included (Fig. 1). Age, sex, medical history, medication, symptoms at presentation, management before and in the ED and at discharge, follow up, including return visits and diagnostics performed, were extracted from medical records.

**Fig. 1:**
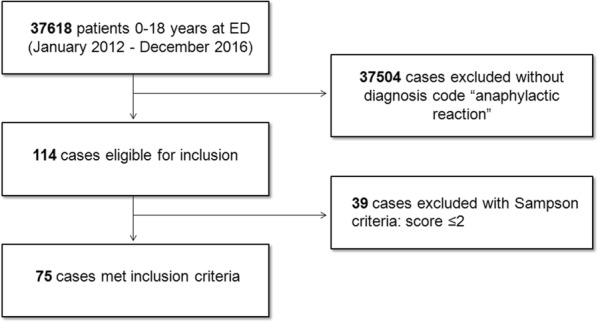
Flowchart of participants

**Results**

Out of 37618 pediatric patients that presented in the ED during our study period, 75 participants met our inclusion criteria, resulting in an incidence from 0.20%. Nutrition represented 91% (68/75) of the causative allergens; with cashew as main provocative factor with 28% (21/75), followed by peanut with 17% (13/75) (Fig. 2). In 40% of all participants (30/75) the allergen, that caused anaphylaxis, was already confirmed and no tests were repeated to confirm the diagnosis at this presentation. In 53% (40/75) the allergen was stated by clinical history and sensitization tests and in 44% (33/75) oral food challenges were performed to confirm the trigger or to reject other possible allergens as a cause. A percentage of 61% (49/75) received epinephrine, from which 36% (27/75) out of hospital and 25% (19/75) in the ED.

**Fig. 2:**
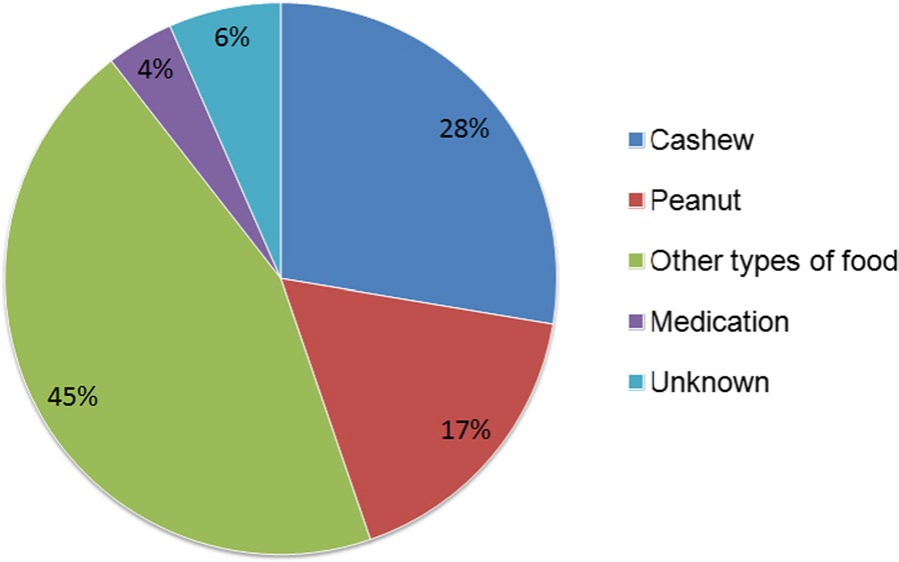
Causative allergens

**Conclusion**

The incidence of pediatric anaphylaxis in the ED is consistent with recent previous studies (3). Allergic reaction to food was most common. Type of allergen is however surprising, with cashew allergy appearing to be obviously more common than expected allergy to peanut. We advise a complete allergy work-up in all children with anaphylaxis, to objectively define the causative allergen in case of anaphylaxis. In our study, administration of epinephrine was only 61%, in this revealing non adherence to local protocols. This is consistent with findings of other studies (6–10).

**References**

1. Simons FE, Ardusso LR, Bilo MB, El-Gamal YM, Ledford DK, Ring J, et al. World allergy organization guidelines for the assessment and management of anaphylaxis. The World Allergy Organization journal. 2011;4(2):13–37. Epub 2011/02/01.
2. Lieberman P, Nicklas RA, Oppenheimer J, Kemp SF, Lang DM, Bernstein DI, et al. The diagnosis and management of anaphylaxis practice parameter: 2010 update. The Journal of allergy and clinical immunology. 2010;126(3):477–80 e1-42. Epub 2010/08/10.
3. Alvarez-Perea A, Ameiro B, Morales C, Zambrano G, Rodriguez A, Guzman M, et al. Anaphylaxis in the Pediatric Emergency Department: Analysis of 133 Cases After an Allergy Workup. The journal of allergy and clinical immunology In practice. 2017. Epub 2017/04/09.
4. Ben-Shoshan M, Clarke AE. Anaphylaxis: past, present and future. Allergy. 2011;66(1):1–14. Epub 2010/06/22.
5. Decker WW, Campbell RL, Manivannan V, Luke A, St Sauver JL, Weaver A, et al. The etiology and incidence of anaphylaxis in Rochester, Minnesota: a report from the Rochester Epidemiology Project. The Journal of allergy and clinical immunology. 2008;122(6):1161–5. Epub 2008/11/11.
6. Hitti EA, Zaitoun F, Harmouche E, Saliba M, Mufarrij A. Acute allergic reactions in the emergency department: characteristics and management practices. European journal of emergency medicine : official journal of the European Society for Emergency Medicine. 2015;22(4):253–9. Epub 2014/05/21.
7. Lauritano EC, Novi A, Santoro MC, Casagranda I. Incidence, clinical features and management of acute allergic reactions: the experience of a single, Italian Emergency Department. European review for medical and pharmacological sciences. 2013;17 Suppl 1:39–44. Epub 2013/04/26.
8. Brown AF, McKinnon D, Chu K. Emergency department anaphylaxis: A review of 142 patients in a single year. The Journal of allergy and clinical immunology. 2001;108(5):861–6. Epub 2001/11/03.
9. Braganza SC, Acworth JP, McKinnon DR, Peake JE, Brown AF. Pediatric emergency department anaphylaxis: different patterns from adults. Archives of disease in childhood. 2006;91(2):159–63. Epub 2005/11/26.
10. Gaeta TJ, Clark S, Pelletier AJ, Camargo CA. National study of US emergency department visits for acute allergic reactions, 1993 to 2004. Annals of allergy, asthma and immunology : official publication of the American College of Allergy, Asthma, and Immunology. 2007;98(4):360–5. Epub 2007/04/27.

## P94

### Cow’s milk allergy and IgE sensitization to cow’s milk protein in lacto-vegetarian children

#### Dmitry Yasakov, Leyla Namazova-Baranova, Svetlana Makarova*, Olga Kozhevnikova, Marina Snovskaya, Tamara Chumbadze, Oksana Ereshko

##### FSAI “NSPCCH” of the MH of the Russian Federation, Moscow, Russia

**Correspondence:** Svetlana Makarova - sm27@yandex.ru

*Clinical and Translational Allergy* 2018, **8(Suppl 2)**:P94

**Introduction**

The popularity of vegetarian diets has increased in recent years, and many parents encourage their children to pursue these types of diets. Increased interest in vegetarian diets (vegan and lacto-ovo-vegetarian) is observed in Russia as well. However, whether such nutrition with the limited set of products play role in development of food allergy or sensitization to this food remains unknown. Aim of the study—to assess the IgE sensitization and symptoms of cow’s milk protein allergy (CMPA) in children following lacto-vegetarian diet.

**Methods**

The study included 69 children aged 1–15 year consuming vegetarian dietary patterns: 18 lacto-ovo-vegetarians, 46 lacto-vegetarians and 5 children (2 ovo-vegetarians, 3 vegans), who restricted their diet after long-term lacto-vegetarian period due to reactions to cow’s milk (CM). The IgE level to CM proteins was measured in blood samples by UniCAP.

**Results**

Self-reported (or parents-reported) allergic reactions to any food had 49 children from 69 (71%). 7 patients had diagnosed atopic dermatitis, 1—oral allergy syndrome, 61 had recurrent skin symptoms. 24 children (34.8%) had self-reported allergic reactions to dairy products. 8 patients (11.6%) had sIgE sensitization to cow’s milk proteins (0.35 ≤ sIgE ≤ 3.5 kUA/l) (3 lacto-ovo-vegetarians, 4 lacto-vegetarian, 1 vegan). Diagnostic elimination diet and oral food challenge test was performed in 16 patients, after this CMPA was confirmed in 10 children.

**Conclusion**

The problem of CMPA often occur among lacto-ovo-vegetarian children. As a result of these allergic reactions parents can restrict or not to change the diet of their children. Consequently it is necessary to pay the attention of pediatricians and allergists for care not only nutritional status of these children, but also allergic sensitization.

## P95

### Are Fish-Oil Supplements (FOS) safe to use in children with fish allergy? A pilot study looking at current trends amongst children with fish allergy

#### Ekaterina Khaleva^1^, Constantinos Kotsapas^2^*, Wei Chern Gavin Fong^3^, Hamed Al-Shammari^4^, Rachel De Boer^2^, Ruth Chalmers^2^, Lauri-Ann Van Der Poel^2^

##### ^1^Saint-Petersburg State Pediatric Medical University, Saint-Petersburg, Russia; ^2^Pediatric Allergy Service, Guys and St Thomas Hospital NHS Foundation Trust, London, United Kingdom; ^3^Queen’s University Belfast, Belfast, Northern Ireland; ^4^King Saud University Hospital, Riyadh, Saudi Arabia

**Correspondence:** Constantinos Kotsapas - kotsapas@gmail.com

*Clinical and Translational Allergy* 2018, **8(Suppl 2):**P95

**Introduction**

Essential fatty acids, known as Omega-3, are becoming increasingly popular due to growing evidence of their beneficial role for optimal health, including cardiovascular health and in allergic disease. National U.K. guidance recommends eating two portions of fish per week, one of which should be oily fish which contains the highest levels of Omega-3. An alternative is to use fish oil supplements (FOS). However, there is little to no data on whether these supplements may be safely taken by those with seafood (fin-fish or shellfish) allergy. Although pharmaceutical-grade fish oil is believed to be of negligible allergenicity, most FOS carry warnings for fish allergy. This retrospective pilot study looks at trends and motivations for taking FOS amongst fish allergic individuals.

**Methods**

Demographic information was collected for 90 patients with fish allergy seen in a tertiary children’s allergy service over a 10-year period. At the time of the study 41 (45%) were adults. We telephoned and completed questionnaires exploring history of reactions, allergic co-morbidities, as well as attitudes and use of fish and non-fish oil-derived supplements, for 19 children and 17 now adult patients.

**Results**

A history of previous hospital attendance and/or adrenaline auto-injector use was positive in 10/17 (58%) of adult patients compared to 1/19 (5%) children; and 16/17 adults (94%) were still avoiding all seafood compared to 6/19 children (31.5%).

Awareness of FOS and Omega-3 s’ potential benefits was high overall. 3/17 adults and 8/19 children were taking a multi-vitamin, whilst 2/17 adults and 1/19 children were taking a flaxseed oil supplement. 6/36 patients had previously taken a FOS with 2 quoting reactions. These were offered FOS challenges. Interestingly 3 parents were currently giving their children multi-vitamins reaction-free, unaware that these contained fish-oil derived Omega-3.

14/17 adults and 15/19 parents reported fear of reaction to FOS as a reason for not currently taking a FOS, with 20/29 scoring their anxiety at maximum. Most of these appeared happy to take a FOS if proven safe.

**Conclusion**

This pilot study reveals growing awareness and popularity of using FOS, specifically amongst medically diagnosed fish-allergic individuals. It also highlights patients who avoid FOS out of fear and those tolerating Omega-3 s unknowingly. Evidence of true reactions from FOS or Omega-3 remains unproven. Patterns of seafood allergy with relevance to reaction risk should be further explored for safe, tailored nutritional advice to our fish allergic patients.

## P96

### Fish Roe: A “Hidden” allergen in Japanesse food?

#### Angela Claver*, Manuel Morales, Begoña Navarro, Carolina El Duque, Elena Botey, Anna Cisteró-Bahima

##### Alergia Dexeus, Instituto Universitario Dexeus, UAB, Barcelona, Spain

**Correspondence:** Angela Claver - aclamonzon@gmail.com

*Clinical and Translational Allergy* 2018, **8(Suppl 2):**P96

**Introduction**

Salmon roe (SR) is a component of traditional Japanesse Food. As Sushi has become widespread, so has SR consumption. Allergic reactions to roes from fish are not commonly reported in western countries and only a few cases can be found in the literature.

**Case report**

A 4-year-old girl was seen in consultation for an anaphylactic reaction after ingestion of 1 roe of salmon. She had a history of egg allergy with tolerance to cocked egg. Her parents were eating japanesse food and she tasted one SR. Within 15 min after ingestion she developed facial angioedema and hoarse voice with no wheezing. Her parents gave her a dose of oral antihistaminic and took her to hospital where she was given oral corticosteroids with resolution. Two years before, she presented facial urticaria and angioedema after ingestion of egg-containing soup and touching SR. Then, symptoms were wrongly attributed to egg allergy. The girl eated fish (salmon and white fish) and shellfish after the reaction with no symptoms.

The skin prick testing were negative to commercial fish (tuna, codfish, hake and sole), shellfish (clam, shrimp and squid) and *Anisakis*. Prick to prick with salmon roe was positive (26 mm wheal flare). The results of prick by prick with cooked and smoked salmon were negative and the diameter of sturgeon (Russian beluga) and raw salmon wheals were less than histamine control (5 mm flare). Salmon specific- IgE was < 0.1 KU/L and Serum sample was taken for roe specific IgE measurement and immunoblotting. We did not perform an oral challenge with roe in our patient given the severity of the initial reaction.

**Conclusion**

Our patient developed a severe reaction to SR without concomitant fish allergy. Fish roe may be a cause of allergic reactions and clinicians should be aware of possible reactions to roe in patients who test negative for fish or shellfish allergy when there is a suspicion of seafood allergy.

**Consent to publish**

The parents of the patient have provided written consent to publish.

## P97

### A critical assessment of an early introduction pathway for food allergy prevention in a tertiary pediatric allergy service

#### Emma Soo^1^*, Jacob Clayton^1^, Katherine Knight^2^, Alia Boardman^2^, Rachel de Boer^2^, George du Toit^2^, Susan Chan^2^, Kate Swan^2^, Helen Brough^2^, Roisin Fitzsimons^2^, Thomas Marrs^2^

##### ^1^University of King’s College London, London, United Kingdom; ^2^Guy’s and St Thomas’ NHS Foundation Trust, London, United Kingdom

**Correspondence:** Emma Soo - emma_soo@hotmail.com

*Clinical and Translational Allergy* 2018, **8(Suppl 2):**P97

**Introduction**

Food allergy can be prevented by the early introduction of allergenic foods amongst infants, however the timing of supervised introduction is crucial. We evaluated the prevalence of milk, egg and peanut sensitisation amongst infants in our service and the uptake of supervised introductions. 

**Methods**

A retrospective data analysis was undertaken of 85 consecutive infants attending clinical appointments between 02/08/2016 and 18/01/2017. 

**Results**

The infants were 8 months 29 days old on average. All 85 patients received skin prick testing, with 72.9%(62/85) tested for egg sensitisation, 62.4%(53/85) for milk and 62.0%(63/85) with peanut extract. 38.4%(28/73) patients demonstrated no sensitisation to egg, milk or peanut on skin testing. 

Amongst the sensitised infants with a positive skin test less than 5 mm, we offered 28.6%(2/7) supervised introduction to cooked egg, none 0%(0/4) were offered supervised milk introduction and 42.9%(3/7) were offered supervised peanut introduction. A proportion of patients were not offered supervised introduction owing to accompanying serum specific IgE results or current uncontrolled eczema.  12 of these 85 infants attended for supervised introduction, six of which were to milk, egg or peanut. 1 infant attending for peanut introduction demonstrated skin sensitisation to peanut < 5 mm. The remaining five infants undertook their supervised introductions at ≥ 5 mm skin sensitisation. The 12 infants supervised introductions took place a median of 115.5 days after being offered. 

**Conclusion**

We highlight the importance of having a well defined and dedicated pathway within a tertiary allergy service to achieve early introduction. We aim to: 

- Increase the proportion of infants sensitised < 5 mm who are offered supervised introductions
- Offer supervised introduction promptly

## P98

### Life after LEAP; A Dutch experience of safe peanut introduction in young infants

#### Anne de Kievit*, Dana-Anne de Gast-Bakker, Marike Stadermann

##### Pediatric department, Diakonessenhuis, Utrecht, The Netherlands

**Correspondence:** Anne de Kievit - annedekievit@hotmail.com

*Clinical and Translational Allergy* 2018, **8(Suppl 2):**P98

**Introduction**

After publication of the LEAP study in 2015, we actively started advising parents of young atopic infants visiting our clinic to introduce peanut at an early age. After a 10 month old atopic boy, who visited our allergy clinic, developed an anaphylactic reaction to a small amount of peanut in an unattended circumstance at home, we started using an early peanut introduction protocol.

**Methods**

From the start of July 2015, all young infants between the age of 4 and 11 months visiting our allergy clinic, who didn’t consume peanut on a regular basis, were included.

If eczema was present, this was first treated before performing a skin prick test for peanut. A skin prick test of 0–3 mm was regarded as negative and introduction of peanut was subsequently considered as “low risk”. Peanut introduction was performed using an updosing protocol, either at home or in the clinic depending on parent’s preferences. Follow up of 2–10 weeks was conducted until weekly consumption of peanut was achieved.

**Results**

54 patients were included, with a mean age at peanut introduction of 10 months. 91% of the patients had mild to severe eczema, 65% had a positive atopic family history. All skin prick tests, except one, were negative. Peanut introduction at home was fulfilled in 32-‘skin prick negative patients’. One patient reported a mild allergic reaction (generalized urticaria) which was treated with antihistamines. Clinical introduction was fulfilled in 22 patients, two patients showed an allergic reaction. One patient developed a mild allergic reaction (generalized urticaria and angio-edema) during introduction. The second patient developed anaphylaxis 4 months after introduction. Peanut elimination was continued after introduction because of severe peanut allergy in the family.

In 50 out of 54 infants peanut was safely introduced. All patients continued consuming peanut during follow up.

**Conclusion**

A skin prick test is a useful predictor of clinical reactivity to peanut in atopic young infants. Early introduction of peanut in atopic young infants can safely be achieved. After early introduction the challenge is to obtain regular peanut consumption.

## P99

### Rabbit has an allergy test

#### Sue Ware, Margaret Potter (joint first author), Penny Salt, Leanne Goh

##### Pediatric allergy clinic, University College London Hospital, London, United Kingdom

**Correspondence:** Wong Leanne - leanne58@me.com

*Clinical and Translational Allergy* 2018, **8(Suppl 2):**P99

**Introduction**

Living with food allergy significantly impacts quality of life of children and their families. Allergy diagnostic tests can add further distress. The health play specialists (HPS) role is vital to ensure that the well-being of patients is upheld and trauma alleviated during testing. Their interventions also facilitate efficiency improving the patient journey. Without HPS support, patients are often reluctant and can refuse leading to longer waiting times and further distress to patients, parents and staff. Good first experiences often make subsequent testing easier. Active and passive distraction methods are provided by the HPS. However, Piaget’s theory recognises that young children are not able to conceptualise abstractly and require concrete physical symbols to exemplify a concept to understand and prepare. Patients aged 2–5 years in this ‘pre-operational developmental stage’ needing the most HPS support.

**Methods**

Appropriate preparation materials were developed by the UCLH HPS to support this age group.

The preparation materials consist of:

1. Practise skin prick testing kit–lancet tip removed, pipette, tissue, container of water (Fig. 1)

**Fig. 1:**
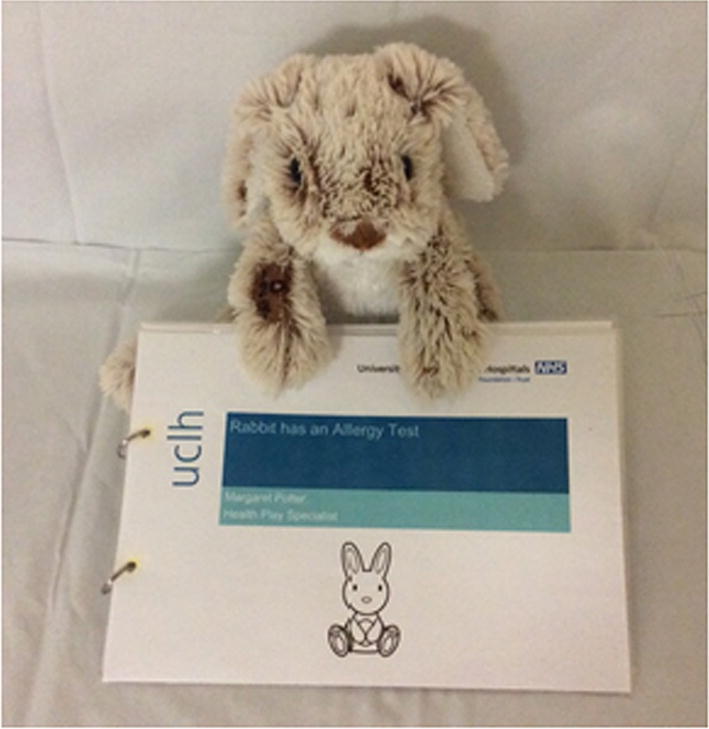
.
2. A story book “Rabbit has allergy testing” (Fig. 2)

**Fig. 2:**
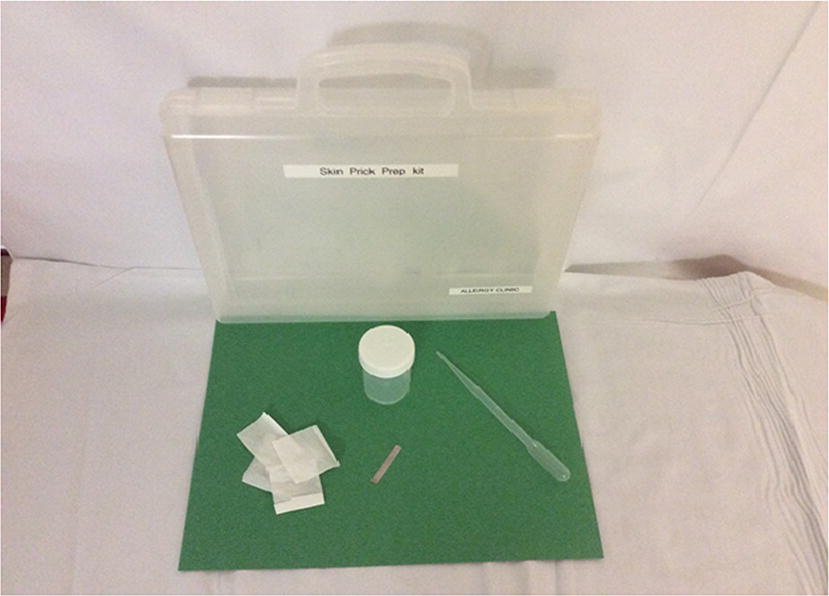
.
3. A soft toy rabbit with numbers written on one arm (Fig. 2)

These materials enable patients to practise the skin prick test on the toy rabbit helping them to familiarise themselves with the equipment. The nurse then practises using these materials on the patient who subsequently accepts introduction of the real lancet during the actual test.

These materials were introduced in allergy clinic in February 2016 and continue to be used to date. Feedback was sought via a short questionnaire.

**Results**

100% found it useful and would recommend it for other families.

Feedback comments include:

“The kit was great in preparing my child”, parent A

“It’s a simple story that children understand”, parent B

“I thought a bigger needle would be used so it was reassuring for me too”, parent C

“All children should read the book before the test”, parent D

“This is valued tool which improves patient experience”, staff A

“It improves compliance, decreases anxiety and is informative”, staff B

“Parents feel empowered to support their child during testing”, staff C

“Has improved efficiency and waiting times’, staff D

**Conclusion**

These materials are acceptable and helpful interventions that support the patient experience by reducing anxiety and improving preparation for allergy tests. They have been well received by patients, families and staff.

## P100

### Anaphylaxis after Elosulphase A infusion: enzyme replacement therapy adverse reactions’ management

#### Esozia Arroabarren*, Elena Aznal, Marta Anda, Jorge Alvarez-Garcia, María Teresa Lizaso, María Teresa Aldunate

##### Complejo Hospitalario de Navarra, Pamplona, Spain

**Correspondence:** Esozia Arroabarren - esoziaa@yahoo.es

*Clinical and Translational Allergy* 2018, **8(Suppl 2):**P100

**Introduction**

Enzyme replacement Therapy (ERT) may delay lysosomal diseases' progression. ERT can induce adverse reactions due to different mechanisms. First ERT for mucopolysaccharidosis (MPS) IV, Elosulphase A (VIMIZIN^®^), has been recently marketed. There have been no previous reports of hypersensitivity reactions to this enzyme.

**Case report**

A 5-year old child affected by MPS IV A was referred to Allergy Consults after 2 adverse reactions after Elosulphase A infusion. He had received Elosulphase A weekly for 5 months (2 mg/k) uneventfully. Standard infusion requires premedication with an antihistamine, a non-steroidal anti-inflammatory drug (NSAID) and a 4-hour long infusion. At 22th week, despite these measures, he developed an urticaria episode, solved with antihistamines and steroids. The following week, Elosulphase A was administered with a slower infusion rate (8-hour), plus the addition of a 2nd antihistamine and steroids to the premedication. Despite these measures, the patient developed an anaphylaxis (urticaria, wheezing and hypoxia) before the completion of elosulphase infusion. Treatment was suspended.

Allergy work-up consisted on skin prick tests ([1/10 and undiluted VIMIZIN^®^ (1 mg/ml) and intradermal (1/100 and 1/1000 dilutions)]) tests if needed. A basophil activation test (BAT) was performed with 5 Elosulphase A concentrations. Five healthy controls underwent skin tests and BAT was performed in 3 controls.

Prick test was positive with the undiluted enzyme. BAT was also positive. Controls tested negative both in skin and BAT tests.

A desensitization procedure with 4 serial dilutions (1:10.000 to 1:1; starting dose: 0.0144 micrograms) and premedication (antihistamines, montelukast and NSAID) was proposed (estimated time: 10 h). Elosulphase A was administered weekly for 5 weeks in desensitizing protocol, requiring infusion rate changes (11–22 h) and premedication additional doses, due to recurrent urticaria episodes. Therefore, Omalizumab (XOLAIR^®^, [75 mg/4 weeks]) was added to the desensitization procedure. Afterwards, the patient developed one urticaria during the 1st desensitization, performed 4 days after omalizumab's initial dose. Further doses have elicited no symptoms and have allowed shortening desensitization’s duration (9 h).

**Conclusion**

Most ERT adverse reactions may be resolved slowing the infusion rate and using premedication. Our patient developed an anaphylaxis despite these measures. Immediate hypersensitivity was confirmed by skin and BAT results. Desensitization is the only option for these patients. However, in our patient, Omalizumab's addition was necessary to achieve procedures' tolerance.

**Consent to publish**

The authors have obtained informed consent of the parents of the mentioned patient.

## P101

### Evaluation of allergy recording in the clinical records of patients with cystic fibrosis

#### Thomas Bradbury*, Noreen West, Nicola Jay, Sally Hutchinson

##### Sheffield University, Sheffield, United Kingdom

**Correspondence:** Thomas Bradbury - tabradbury1@sheffield.ac.uk

*Clinical and Translational Allergy* 2018, **8(Suppl 2):**P101

**Introduction**

To identify the incidence of drug allergy in the Cystic Fibrosis (CF) population at Sheffield Children’s Hospital (SCH) and to identify whether an allergy alert is present in all clinical records where an allergy is suspected.

**Methods**

The electronic records of 151 CF patients attending the SCH were reviewed. Firstly, it was determined whether an Electrical Document Management System (EDMS) alert was present for any drug allergy. Medical and pharmacy records were then reviewed for evidence of previous allergy. The type of drug, the nature of the reaction and whether the patient was referred for formal allergy testing were recorded.

**Results**

39 potential drug allergies were noted in the CF population. The most common recorded reaction was to ceftazidime. Reactions were also common to meropenem, tazocin and co-amoxiclav. Of these suspected drug reactions 24 had an EDMS alert present, while 3 had been referred for formal allergy testing at SCH. This low referral rate has led to confusion regarding which recorded allergies are true allergies and, in some cases, difficulty identifying the causative agent when multiple drugs could have potentially caused the reaction. 42 potential drug intolerances were also noted in the CF population, of which 14 had an EDMS allergy alert present.

**Conclusion**

From our results, we can conclude that there are a large number of potential drug allergies in our CF population. Almost all these reactions are to antibiotics, which is likely due to the high lifetime exposure to these agents. The low referral rate of these suspected allergies for formal allergy testing is of significance as it impacts on whether these agents can be used again in management. A more robust system is recommended to ensure allergies are documented correctly and referred for testing.

## P105

### The effect of a hydrolysed infant formula with added synbiotics on gut microbiota composition and clinical effectiveness in infants at high risk of developing allergy: TEMPO study design

#### Nikolaos G. Papadopoulos^1,2^, Anneke Rijnierse^3^*, Mieke F. Roelofs^3^, Harm Wopereis^3,5^, Johan Garssen^3,4^, Jan Knol^3,5^

##### ^1^Centre for Pediatrics and Child Health, Institute of Human Development, University of Manchester, Manchester, United Kingdom; ^2^Allergy Department, 2nd Pediatric Clinic, University of Athens, Athens, Greece; ^3^Nutricia Research, Utrecht, The Netherlands; ^4^Utrecht Institute for Pharmaceutical Sciences, Utrecht, the Netherlands; ^5^Laboratory of Microbiology, Wageningen University, Wageningen, the Netherlands

**Correspondence:** Anneke Rijnierse - anneke.rijnierse@danone.com

*Clinical and Translational Allergy* 2018, **8(Suppl 2):**P105

**Introduction**

Optimal development of gut microbiota in early life is thought to contribute to an optimal immune maturationand, thus may have a role in allergy prevention [1]. Oligosaccharides (prebioics) and live bacteria in human milk are known to effectively shape the gut microbiome in early life, which is marked by dominant levels of bifidobacteria in breastfed infants compared to formula fed infants [1, 2]. Early dietary intervention with specific prebiotics was demonstrated to modulate the gut microbiome closer to that of breastfed infants [3]. Moreover, a specific combination of pre- and probiotics (synbiotics) showed beneficial effects in allergy management in early life [4, 5, 6]. Infant formulas based on partially hydrolysed proteins (pHP) are developed for infants at increased risk of developing allergy, however the beneficial clinical effects of reduced allergen exposure by such formulas is currently being debated and challenged [7].

The TEMPO study is a randomized controlled double blind trial [NCT03067714] that aims to investigate the effect of a partially hydrolysed protein infant formula with synbiotics (pHP synbiotics) on gut microbiota composition and clinical effectiveness in infants at high risk of allergy.

**Methods**

A total of 600 infants at high risk of allergy based on family history are randomized to either pHP synbiotics or an intact cow’s milk protein-based infant formula without synbiotics (IF). Formula intervention initiates before the age of 16 weeks (combination with breastfeeding allowed) until the age of 12 months. In addition, 100 exclusively breastfed infants at high risk of developing allergy are included in the study as a reference group.

**Results**

Microbiome composition is determined in faecal samples collected before start study product intake and at the age of 17, 26, 39, and 52 weeks. Primary endpoint is levels of bifidobacteriaat 17 weeks of age in the pHP synbiotics group compared to the IF group in a predefined key-group-of-interest (vaginally-delivered, fully formula fed before the age of 4 weeks). Clinical assessment of allergic manifestations is done throughout the study in the total study population. In addition, data on growth, safety and tolerance, as well as biological samples to analyse immune and additional microbiome parameters, are collected.

**Conclusion**

This study is expected to demonstrate modulation of the gut microbiota towards a more favourable composition, marked by increased levels of bifidobacteria. In addition, this study will explore the clinical effectiveness of pHP synbiotics on allergy development, which will be verified in a separate randomized controlled trial.

**References**

1. Wopereis et al., The first thousand days—intestinal microbiology of early life: establishing a symbiosis. Pediatr Allergy Immunol. 2014 Aug;25(5):428–38.
2. Oozeer et al., Intestinal microbiology in early life: specific prebiotics can have similar functionalities as human-milk oligosaccharides. Am J Clin Nutr 2013;98(2):561S-71S.
3. Wopereis et al., Intestinal Microbiota in Infants at High-risk for Allergy: Effects of Prebiotics and Role in Eczema Development. JACI (submitted for publication)
4. Van der Aa, L.B., et al., Effect of a new synbiotic mixture on atopic dermatitis in infants: a randomized-controlled trial. Clin Exp Allergy, 2010. 40(5): p. 795–804.
5. Van der Aa, L.B., et al., Synbiotics prevent asthma-like symptoms in infants with atopic dermatitis. Allergy, 2011. 66(2):170–7.
6. Cuello-Garcia, World Allergy Organization—McMaster University guidelines for allergic disease prevention (GLAD-P): prebiotics. World Allergy Organization Journal. 2016, 9:10
7. Boyle, R.J., et al., Prebiotic-supplemented partially hydrolysed cow’s milk formula for the prevention of eczema in high-risk infants: a randomized controlled trial. Allergy, 2016. 71(5): p. 701–10.

## P106

### A year in allergy: experience of a teaching hospital

#### Antonio Jorge Cabral^1^*, Isis Monteiro^2^, Rute Machado^3^, Joana Fermeiro^3^, Ricardo Fernandes^3^, Ana Margarida Neves^3^

##### ^1^Serviço de Pediatria, Hospital Central do Funchal, Sesaram, EPE, Funchal, Portugal; ^2^Serviço de Pediatria, Hospital Nossa Senhora do Rosário, Barreiro, Portugal; ^3^Unidade de Alergologia Pediátrica, Departamento de Pediatria, Hospital de Santa Maria, CHLN, Lisbon, Portugal

**Correspondence:** Antonio Jorge Cabral - jorge.cabral@vodafone.pt

*Clinical and Translational Allergy* 2018, **8(Suppl 2):**P106

**Introduction**

Allergic diseases have shown increased incidence throughout the years, englobing some of the most common chronic illnesses. This is particularly true in the pediatric population, in whom major morbidity is associated with increased health costs, especially when uncontrolled. Thus, the existence of specialized pediatric units is necessary to assess and treat these children to diminish morbidity and facilitate their transition to a healthy adulthood.

**Methods**

Characterization of all the patients in a specialized pediatric allergy consultation of a tertiary teaching Hospital in a 1-year period, excluding specific consultations, namely food and drug allergy.

**Results**

1784 patients were followed in a total of 2298 consultations, of which 361 (15.7%) were first consultations. 1089 (61%) were male and 695 (39%) were female with a median age of 10 years (max: 20 years; min: 2 months). Most cases were referred from primary health care (32.3%), others from different subspecialties within the same Hospital (26.7%) and the pediatric emergency room (19.6%). The most common diagnoses were rhinitis (45.3%), asthma (32.2%) and atopic dermatitis (11.3%). A total of 989 patients have both asthma and rhinitis. Almost half (48.3%) of all patients with rhinitis have asthma, while almost 64% of all asthmatics also suffer from rhinitis. Skin prick tests were performed on 1512 (84.8%) of the patients, 65.7% of which were positive. Total and specific IgE were measured in 1100 (61.7%) and were positive in 906 (50.8%) of patients. Pulmonary function evaluation was performed in 998 (55.9%) of patients, with a total of 1424 spirometries, of which 387 (27.1%) showed changes, however 54 were inconclusive due to poor collaboration, mainly in younger children. In this unit, specific immunotherapy was also prescribed, with a total of 282 (15.8%) patients receiving treatment. A total of 74 (4.2%) patients were discharged from the consultation and referred mainly to primary health care (68.9%) and to the adult allergy consultation (16.2%).

**Conclusion**

In our experience, allergic pathology affects all ages, with a male predominance. As expected, the main diagnoses are rhinitis and asthma, with many patients suffering from both. Most patients have positive skin prick tests and over half of all investigated had elevated IgE levels and positive specific IgE. Mainly due to age limitations, only half the patients could perform spirometry. Only a few patients are ultimately discharged, exposing the chronicity of this type of disease.

## P107

### Relation between immunisation with DTaP (Diphteria, Tetanus, Pertussis) containing vaccine until 7 months and atopic dermatitis until the age of 6 years

#### Jagelaviciene Agne^1^*, Usonis Vytautas^1^, Drevinskiene Lilijana^2^

##### ^1^Clinic of Children’s Diseases of Vilnius University Medical Faculty, Vilnius, Lithuania; ^2^Centro clinic, Vilnius, Lithuania

**Correspondence:** Jagelaviciene Agne - a.jagelaviciene@gmail.com

*Clinical and Translational Allergy* 2018, **8(Suppl 2):**P107

**Introduction**

Recent studies show that postponed vaccination with DTaP (DTaP/IPV/Hib) containing vaccine at least for 1 month could be related to eczema diagnosis reduction.

Our aim was to determine how variation in vaccination time with DTaP and different number of doses received until the age of 7 months could be related to eczema (atopic dermatitis) diagnosis and its’ course during the first years of life.

**Methods**

We collected the data of children, born in 2009, from the database of one out-patient clinic in Vilnius. Retrospectively we have analysed vaccination status, visits to allergist, children pulmonologist and pediatrician or general practitioner and diagnoses of allergic diseases until 6 years of age. Information about vaccination coverage was retracted from the same database. Permission to perform the study was obtained from the Regional bioethics committee.

**Results**

During the year 2016 we included in the study 1294 children, born in 2009. 1256 (97.1%) children have received three doses of DTaP vaccine overall, but only 1018 (78.7%) on time—by the end of 6th month (plus 1 month). Frequency of allergic diseases did not differ between boys and girls until the age of 6 years, but atopic dermatitis, confirmed by allergist, was more frequently diagnosed in boys until 1 year of age (14.3% boys vs. 10.5% girls, p = 0.037). Atopic dermatitis during the first year of life was predominant diagnosis in unvaccinated children until 7th month with DTaP: 15 out of 64 (23.4%), accordingly to vaccinated with at least one dose of DTaP—145 out of 1230 (11.8%), p = 0.009. Children with confirmed atopic dermatitis during the first year of life were also more prone to postpone or skip DTaP vaccination later on. Vaccination status did not influence the frequency of other allergic diagnoses. We did not determine any relation between the diagnosis of atopic dermatitis until the age of 2 years and up to 6 years and the time of the first DTaP dose: on time (at 2 months plus 1 month) vs. delayed (later than 3 months). There was no relation between the number of DTaP doses received up to 7 months and atopic eczema.

**Conclusions**

Vaccination is not a risk factor for atopy, but eczema or atopic dermatitis still remains one of the most common reasons for vaccination program disturbance without a reasonable background.

## P108

### Safety startup of a high-dose cluster subcutaneous immunotherapyin allergic children under 5 years old

#### Josué A. Huertas Guzmán*, Helena Larramona Carrera, Boris E. Pérez, Montserrat Bosque García, Laura Valdesoiro Navarrete, Oscar Asensio De La Cruz, Xavier Domingo Miró

##### Pediatric Pulmonology and Allergy Unit, Hospital of Sabadell, Parc Taulí Corporation, Barcelona, Spain

**Correspondence:** Josué A. Huertas Guzmán - jahg17@yahoo.com

*Clinical and Translational Allergy* 2018, **8(Suppl 2):**P108

**Introduction**

Alergen-specific subcutaneous immunotherapy is an effective therapy for the treatment of IgE mediated diseases inducing a state of clinical tolerance. The cluster induction therapy allows the maintenance dose to be reached in less time through sequential doses in the same day or several days. Data on the safety startup of high-dose cluster subcutaneous immunotherapy (CSI) in allergic children under 5 years old is scarce. The main objective of this study was to determine the incidence (I) of systemic reactions (SRs) and associated risk factors during the startup CSI in allergic children under 5 years old.

**Methods**

Observational retrospective study of 1 year (2017–2016) in patients ≤ 5 years of age who received the startup CSI (1 or 2 days) at the immunotherapy unit of this Hospital. The studied variables were: SRs according to EAACI classification, age, sex, body mass index (BMI), specific IgE (CAP), vaccine type, therapeutic indication, step of asthma treatment according to GEMA 4.1 and antecedents of asthma exacerbations per year. Data was analyzed statistically using Pearson Chi squared test and cross-tables.

**Results**

A total of 104 injections with different extracts: 82 (81.6%) allergoids and 22 natives (18.4%) were applied to 38 sensitized pediatric patients, 19 boys (50%), age ẋ:4.4 years (2–5 years); with allergic asthma and/or allergic rhinitis (21, 15 and 2 patients). The principal sensitization was Dermatophagoides Pteronyssinus (86.8%); 4 mild to moderate SRs were registered (EAACI SRs SCORE ≤ II), all of them by mites; I: 10.52% per patient, I: 3.85% per injection. All the SRs occurred after the second allergoid injection. The patients with SRs were: 75% girls, had a median total IgE 452UI/ml, step of asthma treatment ≥ 3 (100%), 4.5 asthma exacerbations per year and BMI ẋ:17.17 kg/m2. The CAP levels were higher in patients with SRs (p = 0.006). All SRs were well developed within concomitant rescue medications in the immunotherapy unit. All patients received the full cumulative induction dose.

**Conclusions**

The startup of a high-dose CSI is a safe procedure for children ≤ 5 years old (I: 10.52% per patient, I: 3.85% per injection). A risk factor for SRs in these allergic children was higher CAP levels to Dermatophagoides Pteronyssinus (ẋ: 97.33KU/L).

## P109

### Vitamin D status and allergic disease in a rural Portuguese pediatric population

#### Inês Falcão*, Cláudia Teles Silva, Fernanda Carvalho

##### Pediatric Department - Centro Hospitalar Médio Ave—Vila Nova de Famalicão, Portugal

**Correspondence:** Inês Falcão - Inespatriciofalcao@gmail.com

*Clinical and Translational Allergy* 2018, **8(Suppl 2):**P109

**Introduction**

The protective role of vitamin D against allergy is supported by associations between low serum 25-hydroxyvitamin D (25(OH)D) level and higher rates of asthma, food allergy and IgE sensitization. Recent studies implicated the importance of vitamin D in innate immune defense system and pathogenesis of allergic diseases.

The authors proposed to investigate relationship between vitamin D status and allergy in a Portuguese pediatric sample.

**Methods**

Serum 25(OH)D levels of 97 atopic children and adolescents and 30 non-allergic controls were measured by immunoassay. Subjects were categorized into vitamin D deficiency (≤ 20 ng/mL), insufficiency (21–29 ng/mL), and sufficiency (≥ 30 ng/mL) groups. Control of atopic disease was evaluated and categorized in controlled and uncontrolled asthma, rhinitis or both according Asthma Control and Allergic Rhinitis—Test (CARAT). Atopic biomarkers were also measured for analysis. Statistical analysis was performed using the Xi-square test with a significance level of 0.05.

**Results**

The mean age of atopic and control groups were 12 and 8 years, respectively (p < 0.001). The overall proportion of vitamin D insufficiency and deficiency in this sample was 70.9%. The mean serum 25(OH)D level was 25.6 ng/mL in atopic patients and 24.9 ng/mL in controls (p = 0.727) and proportion of subjects in deficiency, insufficiency and sufficiency groups were similar (p = 0.803). Both total IgE level and circulating eosinophils (Eos%) were similar in these groups (p > 0.2).

Patients with controlled and uncontrolled disease were similar for age, sex, IgE and Eos% levels (p > 0.1), but not for deficiency and insufficiency of 25(OH)D. Uncontrolled allergic disease was correlated with a higher proportion (86.2%) of low levels of vitamin D (p = 0.033).

**Conclusions**

Although the small size sample does not allow us to draw conclusions, we found that vitamin D deficiency and insufficiency are prevalent in our pediatric population. Serum 25(OH)D levels are similar in both groups, atopic and control. Low levels of vitamin D are correlated with uncontrolled atopic disease.

## P110

### DRESS Syndrome due to cefotaxime in a 4 year old girl

#### Stefanaki Evangelia*, Athanasopoulos Emmanouil, Giatzaki Maria, Samonakis Emmanouil, Skopetou Konstantina, Vlachaki Georgia

##### Venizelio General Hospital, Heraklion, Greece

**Correspondence:** Stefanaki Evangelia - linastef74@gmail.com

*Clinical and Translational Allergy* 2018, **8(Suppl 2):**P110

**Introduction**

DRESS syndrome (Drug Reaction Eosinophilia Systemic Symptoms) is a rare drug-induced hypersensitivity reaction which presents with fever, rash (non specific morbiliform, later confluent, infiltrated with purpuric lesions) with facial edema, lymphadenopathy, hematological abnormalities (eosinophilia)and internal organ involvement (liver, kidney, lungs etc.). It typically occurs 2–8 weeks after drug intake and it has been correlated with HHV-6 or Ebstein Barr Virus reactivation.

A 4 year old girl was transferred to our clinic from another Hospital where she was admitted for pneumonia 21 days ago. During the last 4 days fever had reappeared and she had also developed rash.

**Methods**

She was under treatment with cefotaxime (18th day), clindamycin (10th day) and teicoplanin (2nd day). In admission she had a generalised, confluent morbiliform exanthema appearing on face, trunk and extremities, facial and acral edema with periorbital attenuation and lymphadenopathy. Nikolsky sign was negative. Laboratory tests revealed eosinophilia and atypical lymphocytes.

**Results**

DRESS syndrome was suspected, all antiobiotics were stopped and intravenous methylprednisolone was started. Fever stopped during the 2nd day of admission and edema and rash gradually receded. Reactivation of HHV-6 and EBV was not proved. She was discharged 14 days later with instructions of slow tappering of methylprednisolone.

**Conclusion**

Protracted antibiotic therapy is accompanied with increased risk of adverse drug reactions. DRESS syndrome is a rare but serious potentially dangerous hypersensitivity reaction. Early recognition, withdrawal of possible causative drugs, and adequate supportive care are mainstays of improving patient prognosis and reduce morbidities.

**Consent to publish**

Written informed consent to publish has been obtained from the parents of the patient involved in this study.

## P111

### Feasibility of FeNO measurements in young children for epidemiological study

#### Yamamoto-Hanada Kiwako*, Matsumoto Kenji, Saito Hirohisa, Ohya Yukihiro

##### Medical Support Center for Japan Environment and Children’s Study (JECS), Tokyo, Japan

**Correspondence:** Yamamoto-Hanada Kiwako - yamamoto-k@ncchd.go.jp

*Clinical and Translational Allergy* 2018, **8(Suppl 2):**P111

**Introduction**

Fractional exhaled nitric oxide concentration (FeNO) is a biomarkerto evaluate airway inflammation ofasthma noninvasively and safely. Japan Environment and Children’s Study (JECS), nationwide-birth cohort study, is planning to measure FeNO of child participants. The aim of the study is to explore the feasibility of FeNO measurement for young children by using a portable electrochemical device (NIOX VERO ^®^).

**Methods**

Measurements of FENO with NIOX VERO ^®^ were applied for children with asthma and age-matched controls from the age of three to six, who visited allergy clinic in National Center for Child Health and Development, Tokyo. All participants had no experiences for NIOX VERO ^®^ and pediatricians explained to participants how to measure FeNO. They were allowed their challenge from twice to six times if they could not achieve successful measurement once. We defined the acceptable success rate is more than 80% of children who could measure FeNO.

**Results**

Success rate of measurement with NIOX VERO ^®^ was 4/7(44.4%) among children at the age of three, 4/8 (50.0%) among children at the age of four, 6/7 (85.7%) at the age of five, and 5/5 (100%) among children at the age of six. All children commented that the measurement was enjoyable. Only one child made a success in measuring FeNO once. The other children challenged from twice to six times until they could success in measuring FeNO.

**Conclusion**

Measuring FeNO was applicable to young children at the age of five and over. Children may need training several times to measure FeNO.

## P112

### Introduction of solids in a child group with low prevalence of sensitization

#### Helen Rosenlund^1,2^*, Sara Fagerstedt ^1^, Johan Alm ^1,3^, Axel Mie ^1^

##### Karolinska Institutet, Dept of Clinical Science and Education, Stockholm, Sweden; ^2^ Dept of Clinical Nutrition and Dietetics, Div of Orthopedics, Danderyd Hospital, Stockholm, Sweden; ^3^ Sachs’ Children and Youth Hospital, Södersjukhuset, Stockholm, Sweden

**Correspondence:** Helen Rosenlund - helen.rosenlund@gmail.com

*Clinical and Translational Allergy* 2018, **8(Suppl 2):**P112

**Introduction**

Recently scientists reported that an early introduction of strong allergens, i.e. peanuts, was associated with a decreased risk of allergy development in childhood. This is contradictory to former advice. It is of interest to study how introduction of solids is performed in a child group with lower prevalence of allergic disease.

The aim of this study was therefore to investigate if the lower prevalence of sensitization among children of anthroposophic families could be explained by differences in feeding and weaning practices.

**Methods**

The prospective birth cohort ALADDIN includes 550 Swedish children from anthroposophic, partly anthroposophic and non-anthroposophic families. ALADDIN aims at elucidating the role of specific lifestyle factors that could clarify the lower allergy prevalence in anthroposophic children. Allergen-specific IgE against 7 common allergens were obtained from blood samples at ages 6, 12, 24 months, and 5 years old.

**Results**

The anthroposophic child group is breastfed for a longer period compared to the non-anthroposophic children, which confirms previous studies. At 12 months of age, 54% of the anthroposophic, and the partly anthroposophic children, were partly breastfed, compared to 20% in the non-anthroposophic group.

Ten percent of the anthroposophic children have been introduced to replacement and/or infant formulas at 6 months of age, compared to 14% among the partly anthroposophic and 26% among the non-anthroposophic children. At 12 months of age, 95% of the anthroposophic children were introduced to a diet based on organically and/or biodynamically grown foods, compared to 80% in the partly anthroposophic and 20% in the non-anthroposophic groups.

**Conclusion**

The three lifestyle groups differed in introduction of solids to their children.

## P113

### Reversion of asthmatic complications and mast cell signalling pathways in BALB/c mice model using quercetin nanocrystals

#### Kriti Gupta^1,2^*, Rinkesh Kumar Gupta^1^, Premendra Dhar Dwivedi^1^

##### ^1^CSIR-Indian Institute of Toxicology Research, Lucknow UP, India; ^2^Babu Banarasi Das University (BBDU), Lucknow UP, India

**Correspondence**: Kriti Gupta - kritigupta582@gmail.com

*Clinical and Translational Allergy* 2018, **8(Suppl 2):**P113

**Introduction**

The current study is focused on the preparation of water soluble quercetin nanocrystals that are poised to show enhanced bioavailability and therapeutic potential. We investigated well characterized nQ on OVA induced BALB/c mouse model of allergic asthma for various allergic parameters, like serum Immunoglobulin (Ig) concentration, histopathological changes of lung tissue, mediator release assay, mast cell signalling events, providing an effective strategy for treating allergic asthma [1, 2].

**Methods**

1. Preparation and Lyophilization of Quercetin Nanocrystals
2. Physical Characterization of Quercetin Nanocrystals
3. Solubility and Stability of nQ
4. In-Vivo Pharmacokinetics of nQ
5. Total Serum IgE Assay OVA Specific IgE and IgG1 Assay
6. Measurement of Allergic Mediators in the Serum like Prostaglandin D2, cysteinyl leukotrienes, mouse mast cell protease-1and mouse thymic stromal lymphopoietin levels
7. Western Blot Analysis T-bet, GATA-3, c-maf, NfAT and SOCS-3

**Results**

The nQ was found to be more stable and soluble in PBS, and sera of BALB/c mice compared to bulk quercetin. Dose dependent experiments with nQ on OVA sensitized asthma mice exhibited significant anti-asthmatic potential of nQ at much lower dose (1 mg/kg body weight) compared to bulk quercetin. The treatment of nQ remarkably resulted in reduced OVA specific immunoglobulin E (sIgE) production, anaphylaxis signs and type 1 skin test. The nQ also significantly modulated the expression of Th2 cytokines like IL-4 and IL-5, which are responsible for IgE class switching and suppressed the degranulation/secretion of different chemical mediators (PGD2, mMCPT-1 Cys-L and TSLP) from activated mast cells. The levels of Fc*ε*R1, Syk, c-Yes, PI-3, p-PI-3, PLC-*γ*2, and p-PLC-*γ*2 were found to be reduced in the OVA sensitized BALB/c mice treated with nQ compared to those treated with OVA only.

**Conclusions**

In conclusion, the prepared water-soluble quercetin nanocrystals have shown potential as promising nanovehicle with in vivo and ex vivo stability, excellent bioavailability, and therapeutic efficacy in asthma murine model. It has also been found to be effective in preventing anaphylaxis in murine model of allergic asthma. In totality, the nQ has potential to substantially reduce the symptoms of asthma and may play an important role in providing the most effective cure for asthma that is currently lacking.

**Acknowledgements**

Director CSIR-IITR is gratefully acknowledged for his keen interest in the present study. KG is thankful to DST, New Delhi, for the award of Women Scientist Fellowship. Funding from CSIR network project Nano-SHE (BSc 0112) and IUSSTF (GAP-130932) is also appreciatively acknowledged. The authors declared no conflict of interest. This is a CSIR-IITR manuscript no. 3306.

**References**

1. N. G. Sahoo, M. Kakran, L. A. Shaal, L. Li, R. H. Müller, M. Pal, and L. P. Tan, Preparation and characterization of quercetin nanocrystals. J. Pharm. Sci. 100, 2379 (2011).
2. C. Mohanty, S. Acharya, A. K. Mohanty, F. Dilnawaz, and S. K. Sahoo, Curcumin encapsulated MePEG/PCL block copolymeric micelles: A novel controlled delivery vehicle for cancer therapy. Nanomedicine (Lond) 5, 43 (2010).

## P114

### Tacrolimus as an effective and safe therapeutic alternative in vernal keratoconjunctivitis resistant to conventional treatment

#### Monica Gonzalez Medina^1^*, Cristina Blasco Valero^1^, Nieves Martín Begué^2^, Blanca Vila Indurain^1^, Teresa Garriga Baraut^1^

##### ^1^Unidad de Alergología Pediátrica, Hospital Materno Infantil, Hospital Universitario Vall d'Hebron, Barcelona, Spain; ^2^Unidad de Oftalmología Pediátrica, Hospital Materno Infantil, Hospital Universitario Vall d'Hebron, Barcelona, Spain

**Correspondence**: Monica Gonzalez Medina - monicagonzmedina@gmail.com

*Clinical and Translational Allergy* 2018, **8(Suppl 2):**P114

**Introduction**

Allergic Ocular Diseases (AODs) are chronic forms of different conditions that affect the ocular surface and can cause severe visual complications. Topical corticosteroids should be used when the cornea is involved as short pulsed therapy, given the known adverse effects. In several studies Tacrolimus (Tcr) and Cyclosporine A (Cyc)had demonstrated to be an effective treatment in vernal keratoconjunctivitis (VKC). Cyclosporine A has been widely used to treat mild-to-severe VKC, although up to 10% of patients are transiently or in some cases totally unresponsive. Topical Tcr is very effective and safe in the short term for patients suffering from severe VKC resistant to topical Cyc. However treatment with topical Tcr in pediatric population in our geographic area has been scarcely reported. The aim of our study was to evaluate the usefulness of topical ocular treatment with tacrolimus 0.03% ointment in pediatric patients affected by VKC.

**Methods**

This is a retrospective study in patients with VKC refractory to conventional treatment whom were treated with Tcr 0.03% ointment. The diagnosis of allergic conjunctivitis was based on published guidelines. The concept of refractoriness was defined as persistence of symptoms and signs despite the use of conventional treatment. The clinical ocular signs and symptoms were collected and sensitization profile to airborne allergens was also evaluated.

**Results**

Seventeen patients with VKC were included, 12 males and 5 females. The mean age of patients was 12 years old (with a mean age at onset of 6 years old). The family history of atopia was positive in 53% of patients.

The mean time that patients needed treatment with corticosteroids was 4 weeks as minimum. However patients treated with corticosteroids and Tcr concomitantly allowed to reduce significantly the lasting of corticosteroid treatment to 2, 2 weeks. In regard to adverse effects with Tcr only one patient referred burning sensation.

**Conclusion**

Low-dose topical tacrolimus 0.03% ointment is a valid therapeutic alternative in pediatric patients with refractory VKC to conventional treatment, reducing the time with corticosteroid treatment. Moreover it is well tolerated in most patients.

## P115

### Diagnosing penicillin allergy in children: results of a 5-days challenge

#### Birgitte T. Petersen*, Josefine Gradman

##### Department of Pediatrics, Regional Hospital Central Jutland, Denmark

**Correspondence**: Birgitte T. Petersen - tusgaard@dadlnet.dk

*Clinical and Translational Allergy* 2018, **8(Suppl 2):**P115

**Introduction**

Drug allergy is uncommon in children although many children are suspected to be allergic to penicillins. It is unclear whether a challenge test with a single therapeutic dose of the culprit drug is sufficient to elicit reactions in children. Purpose: To investigate whether a prolonged duration of the provocation test with penicillins elicits additional positive reactions in children.

**Methods**

In a prospective study, 104 children with a history of an allergic reaction to penicillins underwent an extended challenge. All children had negative specific IgE to penicillin G and V, ampicillin and amoxicillin (ImmunoCap, Thermo Fisher Scientific). A titrated 2-step challenge was performed with the culprit drug: 1/10 and 1/1 of a therapeutic dose with 60 min interval followed by 2 h observation at the department. In case of no reaction the challenge continued at home with three daily doses for five days. The study was performed from March 2014 to April 2017 at the Department of Pediatrics, Regional Hospital Central Jutland.

**Results**

A total of 104 children (boys 57%) aged 8 months to 14 years (mean 4.7 years) had either a history of urticaria (48.0%) maculopapular rash (44.2%) *erythema multiforme* (1.9%) angioedema (1.9%), trouble breathing (0.9%) or gastrointestinal symptoms (2.8%) related to treatment with penicillin. The culprit drug was penicillin V (47%), amoxicillin (40.3%), dicloxacillin (5.7%), mecillinam (2.9%) amoxicillin/clavulanic acid (1.9%), and ampicillin (1.0%). Seven (6.7%) children had a positive challenge. Four of the children had a positive reaction at the department within 2 h after either 1/10 (1 child) or a 1/1 dose (3 children). Two children had a cutaneous reaction at home at day 1 (more than 8 h after 1/1 therapeutic dose) and one child reacted at day 4 of the provocation test with cutaneous symptoms.

**Conclusion**

In this study 42% of the positive reactions were observed during the prolonged provocation at home. Extending the duration of the challenge seems to be useful in diagnosing penicillin allergy in children.

## P116

### Immediate allergic reaction by evacuant solution BOHM^®^

#### Mariangelica Bermudez Martinez*, Ricardo Moreno Borque, Pilar Gajate Fernandez, Maria Del Mar Rodriguez Gonzalez, Vanesa Cintas Jaramillo, Paula Sanchez Lopez, Rebeca Martin Recio Rey Juan Carlos Hospital, Madrid, Spain

**Correspondence**: Mariangelica Bermudez Martinez - mariangelicabermudezmartinez@gmail.com

*Clinical and Translational Allergy* 2018, **8(Suppl 2):**P116

**Introduction**

The evacuant solution (BOHM^®^) is a drug that has been used to clean the colon, containing Polyethylene glycol: PEG 4000, NaCO3: 420 mg, NaPO3: 120 mg, KCL: 186.25 mg, NaSO3: 1.41 g. The most frequent side effects are Abdominal pain, Diarrhea, and other abdominal symptoms.

**Case report**

We observed a 17 year’s old boy who presented immediate (3 min) urticaria, angioedem and nauseas after taking a BOHM^®^ evacuant solution prior to undergoing a colonoscopy. The patient went to the emergency room and received medical treatment consisting of: corticoids and H1 antihistamines. The patient consulted with the allergist doctor after obtaining the medical history and after providing consent, we perfomed allergy studies: skin prick test with common inhalants and with evacuant solution (BOHM^®^), the results were negative. We performed an oral challenge with evacuant solution (BOHM^®^), 10 min later, the patient presented pruritus signs in his body and urticaria in his face, neck, thorax and abdomen, without any other symptoms. Figures 1 and 2.

**Fig. 1:**
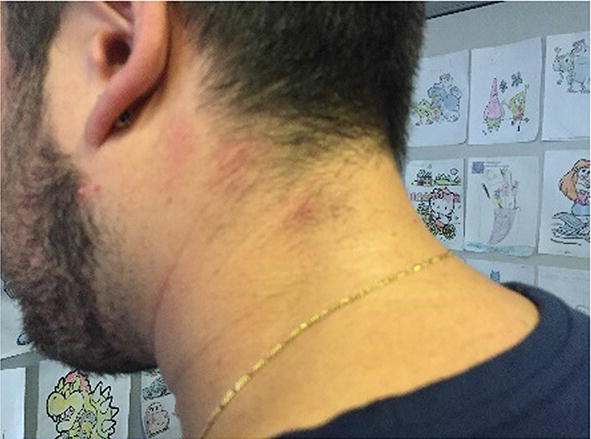
.

**Fig. 2:**
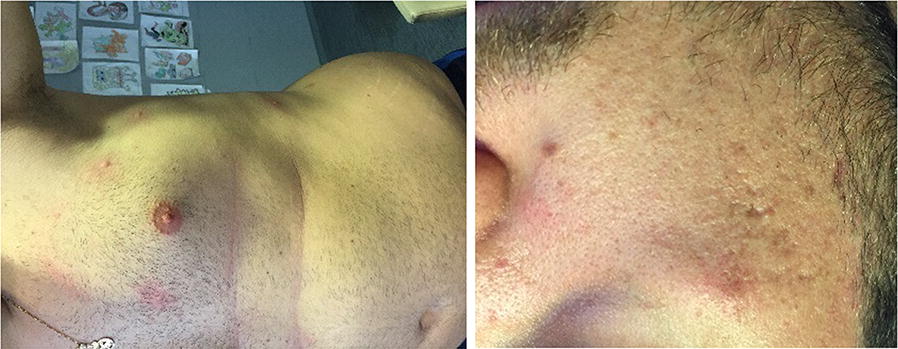
.

We performed an oral challenge with Citrafllet^®^ with negative results as well.

**Conclusion**

We described an immediate allergy reaction (type 1) caused by oral evacuant solution (PEG 400). We will think in the PEG excipient as the allergy cause in our patients.

**Consent to publish**

Informed constent for presentation and publication was obtained.

## P117

### Short Chain Fatty Acid as a dietary adjuvant in oral Peanut Immunotherapy

#### Michelle Barnes^1^, Laurence Macia^2^, Sam Mehr^1^, Peter Hsu^1,3^, Paul J. Turner^3,4^, Mingjing Hu^5^, Brigitte Nanan^5^, Charles Mackay^6^, Alice Lee^7^, Trevor Lockett^8^, Julie Clarke^9^, Ralph Nanan^5^, Dianne E. Campbell^1,3^*

##### ^1^Department of Allergy and Immunology, Children’s Hospital at Westmead, Sydney, Australia; ^2^Charles Perkins Centre, University of Sydney, Sydney, Australia; ^3^Child and Adolescent Health, University of Sydney, Sydney, Australia; ^4^Section of Pediatrics, Imperial College London, London, United Kingdom; ^5^Nepean Clinical School, University of Sydney, Sydney, Australia; ^6^Microbiology, Monash University, Melbourne, Australia; ^7^ARC Training Centre for Advanced Technologies in Food Manufacture, University of New South Wales, Sydney, Australia; ^8^CSIRO (Food and Nutritional Sciences), Sydney, Australia; ^9^CSIRO (Animal, Food and Health Sciences), Adelaide, Australia

**Correspondence**: Dianne E. Campbell - diannec3@chw.edu.au

*Clinical and Translational Allergy* 2018, **8(Suppl 2):**P117

**Introduction**

There is increasing epidemiological and in vitro evidence that dietary fibre, short chain fatty acids (SCFA)—and particularly butyrate—may have a role in modulating immune tolerance. Recent work by our study group has shown that butyrate significantly impairs allergic responses in peanut-sensitised mice via GPR109a, a G-protein-coupled receptor, with induction of regulatory T cells [1].

Peanut oral immunotherapy (OIT) has been shown to be quite successful in leading to short term desensitisation, albeit with a range of reported side effects- but longer term sustained unresponsiveness to peanut has been harder to achieve. We hypothesize that the use of oral SCFA (butyrate) during peanut OIT will induce regulatory responses leading to sustained unresponsiveness, with an improved safety profile. We are testing this in a 3-arm RCT.

**Methods**

To provide further rationale for a clinical trial we undertook a series of in vitro experiment which support the notion that butyrate (and other SCFA) induces regulatory immune responses in human T and dendritic cells.

Using PBMC from healthy and atopic donors, we demonstrated that CD3 + CD4 + CD45RA + naïve T-helper cells derived from PBMC and cultured in the presence of SCFA acquire a Treg phenotype and suppressive capacity in vitro. We also demonstrated SCFAs skew human dendritic cells towards a tolerogenic phenotype.

OPIA study: *O*ral *P*eanut *I*mmunotherapy with *A*djuvant

The OPIA study is a parallel 3-arm RCT safety and efficacy study, due to commence August 2017 (ANZCTR registration ACTRN12617000914369) in Sydney, Australia. We will recruit 160 (challenge-proven) peanut-allergic subjects aged 10–16 years. Participants will be randomised to one of 3 groups: no invention (unblinded); daily peanut OIT + daily butyrylated high amylose maize starch supplement; or daily peanut OIT + placebo (low amylose maize starch), for 12 months. Participants, families and the study team are double-blinded to the dietary supplement allocation.

**Results**

The primary outcome is tolerance to > 1.4 g peanut protein following 6 weeks OFF all study interventions, assessed at 58 weeks after randomisation. Secondary outcomes include changes in eliciting dose over study period, safety/AEs, QoL, induction of allergen specific T and B-regulatory cells, histone acetylation and DNA methylation changes, peanut IgE, IgG4 and changes in gut microbiome.

**Reference**

1. Tan J, McKenzie C, Vuillermin PJ, et al. Dietary Fiber and Bacterial SCFA Enhance Oral Tolerance and Protect against Food Allergy through Diverse Cellular Pathways. Cell Rep. 2016;15(12):2809–2824. doi: 2810.1016/j.celrep.2016.2805.2047.

## P118

### Incidence of parent reported food hypersensitivity in Greek children at 4 and 6 years of age: results from a birth cohort study in Crete

#### Evangelia Stefanaki^1^*, Aikaterini Margetaki^2^, Theano Roumeliotaki^2^, Leda Chatzi^2,3,4^

##### ^1^Department of Pediatrics, Venizelion General Hospital, Heraklion, Greece; ^2^Department of Social Medicine, Faculty of Medicine, University of Crete, Heraklion, Greece; ^3^Department of Preventive Medicine, Keck School of Medicine, University of Southern California, Los Angeles, United States of America; ^4^Department of Genetics and Cell Biology, Faculty of Health, Medicine and Life Sciences, Maastricht University, Maastricht, The Netherlands

**Correspondence**: Evangelia Stefanaki - linastef74@gmail.com

*Clinical and Translational Allergy* 2018, **8(Suppl 2):**P118

**Introduction**

Food hypersensitivity is increasing in Western world but true incidence varies between different populations and is not well documented in all countries. We aimed to evaluate the prevalence of parent-reported food hypersensitivity in a population based sample from the Rhea birth cohort study in Crete, Greece.

**Methods**

Information about potential allergic reactions to food was collected at the 4 and 6 year follow ups. Specifically, at the 4 years’ follow up, parents were asked to choose between a list of foods (fruits-vegetables, dried fruits, milk, egg white, fish, peanut, other) and a list of reactions for each food (facial edema, Quincke edema, generalized exanthema, vomit or diarrhea, dyspnea, fainting, loss of consciousness). During the 6 years’ follow up, parents were asked to choose between cow’s milk, hen’s egg, wheat, cod, soya, peanut and other food but there was no list of reactions.

**Results**

Among the 917 children with available data on food allergies at the 4 years’ follow up, 97 (10.6%) had a parent-reported “adverse food reaction, ever“and 26 (2.9%) of them had an “insisting allergic reaction to food”.Reactions were mainly triggered by tomato (1.3%), milk (1.7%), egg white (1%), fish (1%) peanut (0.3%) and treenuts (0.3%). The median (IQR) age at first reaction was 12 (8, 29) months.

Among the 623 children with available data on food allergies at the 6 years’ follow up 37 (5.9%) children had a doctor diagnosed “allergic reaction to food, ever“and 12 (1.9%) presented an “insisting allergic reaction to food”.Reactions were mainly triggered by cow’s milk (2.1%), hens egg (1.9%) peanut (0.8%), and cod (0.6%). The median (IQR) age at reaction was 12 (12, 30) months.

**Conclusions**

The recorded prevalence of parent reported allergic reactions to food in the Rhea mother–child cohort study of Greek children is relatively low compared to the ones described in Western world.

## P120

### A prospective observational study assessing the impact of bone marrow transplantation on transfer, loss or de novo acquisition of allergen-specific sensitizations

#### Markus Debiasi^1^*, Herbert Pichler^2^, Florian Klinglmüller^3^, Klara Schmidthaler^1^, Jonas Rech^1^, David Scherer^1^, Christian Lupinek^4^, Rudolf Valenta^4^, Ewa Kacinska-Pfaller^2^, Heidrun Boztug^2^, Christina Peters^2^, Cezmi A. Akdis^5^, Zsolt Szépfalusi^1^, Thomas Eiwegger^1,6,7,8^

##### ^1^Department of Pediatrics and Adolescent Medicine, Medical University of Vienna, Vienna, Austria; ^2^Department of Pediatrics and Adolescent Medicine, St. Anna Children’s Hospital, Medical University of Vienna, Vienna, Austria; ^3^Center for Medical Statistics, Informatics, and Intelligent Systems, Medical University of Vienna, Vienna, Austria; ^4^Division of Immunopathology, Department of Pathophysiology and Allergy Research, Center for Pathophysiology, Infection and Immunology, Medical University of Vienna, Vienna, Austria; ^5^Swiss Institute of Allergy and Asthma Research (SIAF), University of Zurich, Davos, Switzerland; Christine Kühne-Center for Allergy Research and Education, Davos, Switzerland; ^6^Division of Immunology and Allergy, Food allergy and Anaphylaxis Program, The Department of Pediatrics, The Hospital for Sick Children, Toronto, Canada; ^7^Research institute, The Hospital for Sick Children, Translational Medicine program, Toronto, Canada; ^8^Department of Immunology, The University of Toronto, Toronto, Canada

**Correspondence:** Markus Debiasi - markus.debiasi@meduniwien.ac.at

*Clinical and Translational Allergy* 2018, **8(Suppl 2):**P120

**Introduction**

Transfer of IgE-mediated allergies via bone marrow transplantation (BMT) has been described in some case reports and small series. Prospective studies investigating the probability of transferring, losing or *de novo* acquisition of allergic sensitization after BMT have been lacking. Understanding this process may allow a relevant risk assessment for BMT from anaphylactic donors and gain new insights in the cellular and molecular origins of allergy development and allergen-specific immune responses in the context of immune system reconstitution.

**Methods**

A single center prospective observational study to assess the probability of transfer or maintenance of allergen-specific sensitizations by BMT was conducted from 2011 to 2016 at the Medical University of Vienna. We enrolled 50 children before BMT. Allergen-specific IgE and IgG to 175 micro-arrayed allergens was measured using an allergen chip, based on ISAC technology. Sensitizations of donors and recipients were assessed pre-BMT and in recipients at 6, 12 and 24 months post-BMT. Based on a mixed effect model we computed a risk-estimation of IgE- or IgG-sensitization 2 years post-BMT as a function of donor and recipient sensitization status pre-BMT.

**Results**

At baseline, 25/50 donors and 19/50 recipients displayed allergen-specific IgE to individual components (n = 174 vs.187 individual allergen sensitizations). The majority of IgE sensitizations pre-BMT was lost 2 years post-BMT with exception of 9 individual allergen-specific responses (7 transferred from the donor, n = 4 of 32 recipients who completed the study; 2 maintained, n = 1 of 32 recipients). The donor-related sensitizations (n = 7) were transiently lost (6 months) and redeveloped until 24 months post-BMT. One sensitization was developed *de novo* (n = 1 of 32 recipients). The estimated risk of maintaining (recipient to recipient) or transferring (donor to recipient) an individual allergic sensitization 2 years post-BMT was 4% for both scenarios (p < 0.05). No reliable risk estimation was possible in case of matched sensitizations (donor and recipient sensitized to the same allergen). Donor and recipient pre-BMT allergen-specific IgG were transferred to the recipient 2 years post-BMT (probability 25% if only the donor, or 31% if only the recipient has specific IgG to the respective allergen or 80% in case of matched allergen-specific IgG). IV-Immunoglobulin substitution did not show any impact on the outcome.

**Conclusion**

This is the first prospective trial demonstrating that BMT significantly reduces the maintenance of allergic sensitization. However, there is a considerable risk in the range of 4% to transfer allergen-specific immune responses from the donor to the recipient, which should be considered for post BMT management.

**Funding**

Austrian Science Fund (FWF) SFB F4615

## P121

### Vitamin D levels and allergic airway disease at school age—results from the PAPS cohort

#### Siri Rossberg^1^*, Valentina Belzer^2^, Petra Wagner^1^, Stephanie Hofmaier^1^, Thomas Geske^3^, Kurt Zimmermann^4^, Mohammed Zaino^5^, Ulrich Wahn^1^, Eckard Hamelmann^6^, Susanne Lau^1^

##### ^1^Department for Pediatric Pneumology and Immunology Charité—Universitätsmedizin, Berlin, Germany; ^2^Klinik für Kinder- und Jugendmedizin, Helios Klinikum, Berlin, Germany; ^3^TG Medical Services, Berlin, Germany; ^4^Symbiopharm GmbH, Herborn, Germany; ^5^Biostatistics, Leipzig, Germany; ^6^Kinderzentrum Bethel, Evangelisches Krankenhaus Bielefeld, Bielefeld, Germany

**Correspondence:** Siri Rossberg - siri.rossberg@charite.de

*Clinical and Translational Allergy* 2018, **8(Suppl 2):**P121

**Introduction**

The crucial role of vitamin D and the development of allergic airway disease (asthma, allergic rhinitis) is still under debate. Repetitively severe asthma and asthma exacerbations have been found to be associated with low levels of vitamin D. We sought to investigate vitamin D levels in school age children at risk for allergic airway disease.

**Methods**

This study (PAPS) was initially started as a RCT with 606 newborns from allergic families. Since the intervention had no effect on the primary outcome atopic dermatitis, the study was continued without intervention as an observational cohort. A standardized follow-up assessment at school age (5–12y) was performed using clinical assessment by examination, sensitization to most common areo-allergens, spirometry and validated parent-reported ISAAC questions on typical symptoms and a previous doctor`s diagnosis of asthma (ever) to define current asthma and allergic rhinitis (in the last 12 months).

Serum levels of vitamin D3 were measured using the chemiluminescence assay IDS-iSYS 25 Hydroxy Vitamin D test. The cut-off for vitamin D3 deficiency was assumed at < 20 ng/l. To determine the association of vitamin D levels and the diagnosis of allergic airway disease Pearson Chi square test was used.

**Results**

Among the 403 participants at school-age, Vitamin D levels were available for 284 individuals. 48/284 had asthma and 86/284 had allergic rhinitis. Vitamin D levels ranged from 4.0 to 66.1 ng/ml. Mean levels for wintertime were slightly lower than for summertime.

25 children with asthma (18.9%) had vitamin D levels < 20 ng/l and 23 children with asthma (15.2%) > 20 ng/l. 34 children with allergic rhinitis (25.8%) had vitamin D levels below < 20 ng/l and 52 children with allergic rhinitis (34.4%) above > 20 ng/l. 92 healthy individuals (50.3%) had an insufficient vitamin D level at < 20 ng/ml and 91 children (49.7%) had a normal vitamin D level > 20 ng/ml.

**Conclusion**

No differences were observed in children with allergic airway diseases concerning vitamin D levels below and above 20 ng/l. Healthy as well as allergic participants showed vitamin D levels below 20 ng/ml. Epidemiologic studies are needed to determine normal and low levels of vitamin D as well as interventional trials to estimate the potential role of vitamin D in the development of allergic airway disease furthermore.

## P122

### Hypersensitivity to betalactamics in children: clinical profile and diagnostic investigation in an outpatient clinic in Rio de Janeiro-Brazil

#### Ekaterini Goudouris*, Camila Lira, Evandro Prado, Fernanda Pinto Mariz, Heloiza Silveira, Maria Fernanda AMA Motta

##### IPPMG - UFRJ, Rio de Janeiro, Brazil

**Correspondence:** Ekaterini Goudouris - egoudouris@gmail.com

*Clinical and Translational Allergy* 2018, **8(Suppl 2):**P122

**Introduction**

Betalactams are commonly prescribed by pediatricians for various types of infection. Most of the suspected hypersensitivity reactions are related to this class of antimicrobials. However, studies have shown that there is an overdiagnosis of hypersensitivity to these drugs. We aim to describe the clinical profile and the diagnostic investigation in children with suspected hypersensitivity to beta—lactams followed in an allergy and immunology service in Rio de Janeiro–Brazil.

**Methods**

Retrospective descriptive study based on data collection in medical records, using the ENDA questionnaire, from patients followed from March 2015 to May 2017.

**Results**

Thirty patients with suspected reaction to beta-lactams were analyzed, with a total of 35 reactions: 25—amoxicillin, 3—amoxicillin with clavulanic acid, 1—ampicillin, 5—cephalexin and 1—cefaclor. Of these patients, 13 were female and 17 were male. The age at reaction time ranged from 3 months to 10 years, with a median of 23 months. The majority of reactions were late (71.4%). Cutaneous manifestations were the most common: maculopapular rash (40%), urticaria (48.5%) and angioedema associated with rash or urticaria (20%). The diagnostic investigation included: skin prick tests (none positive) and intradermal (ID) test (66.7% positive) and, when available, specific IgE dosage (90% negative). Thirty-two oral challenge tests were carried out, discarding the diagnosis of hypersensitivity in 96.9%. The diagnosis of allergy to beta-lactams was confirmed only in three patients (10%): two by ID test and one by oral challenge.

**Conclusion**

Hypersensitivity to beta-lactams was not confirmed in most of the patients in our group, as it has been described in the literature. The diagnostic approach is complex, which contributes to the overestimation of these reactions, leading to unnecessary restrictions on patients. Proper investigation discards the diagnosis in most cases.

## P124

### Prenatal maternal stress (PNMS) and offspring’s asthma and allergy risk: a systematic review and meta-analysis

#### Audrey DunnGalvin^1^*, Catherine Flanigan^1^, Aziz Sheik^2^, Bronwyn Brew^1^, Catarina Almquist^3^, Bright Nwaru^2^

##### ^1^University College Cork, Cork, Ireland; ^2^University of Edinburgh, Edinburgh, United Kingdom; ^3^Karolinska Institute, Stockholm, Sweden

**Correspondence:** Audrey DunnGalvin - A.DunnGalvin@ucc.i.e.

*Clinical and Translational Allergy* 2018, **8(Suppl 2):**P124

**Introduction**

The continuing increase in atopic disease prevalence has given rise to a search for environmental risk factors, which have the potential for modification. Within the fetal programming framework, prenatal maternal stress (PNMS) may influence offspring’s atopic risk. We undertook a comprehensive synthesis and meta-analysis of studies on maternal prenatal stress and risk of allergy and asthma in the offspring, taking into account the type of stress, timing of exposure, and full spectrum of allergy outcomes.

**Methods**

We searched 11 electronic databases from 1960 to end 2016 and a search of the grey literature. Type of stress included mood disorders, pregnancy related anxiety, exposure to violence, bereavement and socio-economic problems. We conducted random-effects meta-analyses to quantitatively synthesize the data.

**Results**

We identified 9779 papers of which 30 (enrolling> 6 million participants) met inclusion criteria. Maternal exposure to any type of stressor was associated with an increased risk of atopic eczema/dermatitis (OR 1.34, 95% CI 1.22–1.47), wheeze (OR 1.34, 95% CI 1.16–1.54), asthma (OR 1.15, 95% CI 1.04–1.27), and allergic rhinitis (OR 1.30, 95% CI 1.04–1.62), but decreased the risk of atopic sensitization (OR 0.92, 95% CI 0.86–0.98). The majority of studies used self-report tools to measure stress. Of the 30 studies graded for quality, four of the studies were strong, 25 were moderate, and one study was weak. The results showed that PNMS (principally 3rd trimester) was associated with an increased risk of current or ever wheeze in the offspring, atopic eczema/dermatitis, and allergic rhinitis in the offspring. Findings were similar for stressor types (Mean HR 1.3, 95% CI 1.-1.50).

**Conclusion**

Maternal prenatal exposure to psychosocial stress increased the risk of adverse allergy outcomes in the offspring. This may represent a causal association or a result of residual confounding. Consensus is needed on the use of validated assessment tools. The combined use of self-report and objective measures would provide a more robust and informative understanding of the contribution of environmental and mechanistic factors.

## P125

### Results of drug hypersensitivity investigations in children referred to the Allergy Unit of Anna Meyer Children’s Hospital over the course of 16 months

#### Alessandra Piccorossi^1^*, Francesca Mori^2^, Simona Barni^2^, Elio Novembre^2^

##### ^1^Department of Pediatrics, University of L’Aquila, San Salvatore Hospital, L’Aquila, Italy; ^2^Allergy Unit, Department of Pediatrics, Anna Meyer Children’s University Hospital, Florence, Italy

**Correspondence:** Alessandra Piccorossi - alepiccorossi@gmail.com

*Clinical and Translational Allergy* 2018, **8(Suppl 2):**P125

**Introduction**

Evaluation of drug hypersensitivity in pediatric age is a common topic of debate, especially becauΑse prescribed drugs differ between children and adults and drug reactions often require a differential diagnosis with viral infections. The aim of our study was to evaluate the clinical history, the diagnostic work-up results and the sensitization profile of children referred to our Allergy Unit for suspected drug hypersensitivity reactions.

**Methods**

From January 2016 to April 2017, a group of 533 patients with history of drug reactions were retrospectively evaluated. The allergy diagnostic work-up included skin tests (ST), in vitro tests and drug provocation test (DPT).

**Results**

The age range varied between 1 and 17 years (M 294; F239). The results of the allergy work-up are summarized in Table 1.

**Conclusion**

In our study Beta-lactams (BLs) were the drugs most commonly involved in the reported reactions followed by Non Steroidal Anti-Inflammatory Drugs (NSAIDs). In the group of BLs, ST were positive in the 2.9% of patients and the diagnosis of BLs allergy was confirmed by DPT in the 4.1% of patients. In the group of NSAIDs a positive DPT with the culprit drug was obtained in the 6.9% of patients. In children, delayed severe cutaneous reactions have been most frequently observed during BLs course treatments, while patients using NSAIDs more commonly reported anaphylaxis.  

## P126

### Identification of functional peptides with tolerogenic potential in a partially hydrolysed infant formula

#### Joost W. Gouw^1±^*, Juandy Jo^2,3±^, Laura A.P.M. Meulenbroek^1,3^, Sam Heijjer^1,3^, Erica Kremer^1^, Elena Sandalova^2,3^, André Knulst^4^, Sergio Oliveira^1^, Jan Knol^1^, Johan Garssen^1,3^, Anneke Rijnierse^1^, Léon M.J. Knippels^1,3^

##### ^1^Nutricia Research, Utrecht, The Netherlands; ^2^Nutricia Research, Singapore, Singapore; ^3^Division of Pharmacology, Utrecht Institute for Pharmaceutical Sciences, Utrecht University, Utrecht, The Netherlands; ^4^Department of Dermatology and Allergology, University Medical Centre Utrecht, Utrecht, The Netherlands; ^±^These authors contributed equally to this work

**Correspondence:** Joost W. Gouw - joost.gouw@danone.com

*Clinical and Translational Allergy* 2018, **8(Suppl 2):**P126

**Introduction**

Oral tolerance is the default response of the immune system to innocuous food proteins and is characterized by regulation of local and systemic immune responses to these proteins [1]. Failure to induce oral tolerance to food proteins results in food allergy. International prevention guidelines recommend the use of partial hydrolysed cow’s milk-based infant formula in infants with increased risk of developing allergic diseases, when breastfeeding is limited or absent [2, 3]. The aim of this study was to investigate whether a specific partial hydrolysed whey-based infant formula contains unique peptides that might act as functional human T-cell epitopes to support the development of oral tolerance to whey.

**Methods**

First, a novel liquid chromatography-mass spectrometry (LC–MS) method was developed to characterise beta-lactoglobulin (BLG)-derived peptides present in a whey-based hydrolysed formula with a particular focus on AA13–48 of the mature BLG protein, a region which has previously been described to contain T-cell epitopes with tolerogenic potential [4]. Second, the same formula was subjected to the ProImmune ProPresent^®^ antigen presentation assay to identify HLA-DRB1-restricted, BLG-derived T-cell epitopes. Third, synthetic peptides identical to the BLG-derived peptides identified by LC–MS were tested on human cow’s milk-specific T-cell lines to determine T-cell recognition.

**Results**

Thirteen BLG-derived peptides of minimal 9AAs long that overlap with the region of AA13–48 of mature BLG were identified. Six of them were found across all batches analysed. It was further confirmed that these peptides were internalized, processed and presented by human dendritic cells. The identified HLA-DRB1-restricted T-cell epitopes were correlated to AA11–30 and AA23–39 of mature BLG. Importantly, the T-cell proliferation assay showed that the synthetic peptides were recognized by cow’s milk-specific T-cell lines and induced T-cell proliferation.

**Conclusion**

This study demonstrates that the tested partially hydrolysed whey-based infant formula contains functional HLA-DRB1-restricted T-cell epitopes. These functional peptides in turn can potentially support the development of oral tolerance to whey.

**References**

1. Pabst O, Mowat AM. Oral tolerance to food protein. Mucosal Immunol 2012:5:232–239.
2. Fleischer DM, Spergel JM, Assa’ad AH, Pongracic JA. Primary prevention of allergic disease through nutritional interventions. J Allergy Clin Immunol Pract 2013:1:29–36.
3. Muraro A, Halken S, Arshad SH, et al. EAACI food allergy and anaphylaxis guidelines. Primary prevention of food allergy. Allergy 2014:69:590–601.
4. Meulenbroek LA, van Esch BC, Hofman GA, et al. Oral treatment with beta-lactoglobulin peptides prevents clinical symptoms in a mouse model for cow’s milk allergy. Pediatr Allergy Immunol 2013:24:656–664.

